# Orforglipron for maintenance of body weight reduction: the double-blind, randomized phase 3b ATTAIN-MAINTAIN trial

**DOI:** 10.1038/s41591-026-04386-7

**Published:** 2026-05-13

**Authors:** Louis J. Aronne, Deborah B. Horn, Carel W. le Roux, Ariana M. Chao, Wayne Ho, Bruno Halpern, Ryan Griffin, Cathy Xie, Elisa Gomez Valderas, Clare J. Lee, Anderson Ribeiro, David M. Hyman, Leonard Glass, Neena Xavier

**Affiliations:** 1 Comprehensive Weight Control Center, Division of Endocrinology, Diabetes and Metabolism, Weill Cornell Medicine, New York, NY USA; 2 Center for Obesity Medicine and Metabolic Performance, Departments of Surgery and Internal Medicine, University of Texas McGovern Medical School, Houston, TX USA; 3 Metabolic Medicine, School of Medicine, Conway Institute, University College Dublin, Dublin, Ireland; 4 Diabetes Research Centre, Ulster University, Coleraine, UK; 5 Johns Hopkins School of Nursing, Baltimore, MD USA; 6 Keck School of Medicine of University of Southern California, Los Angeles, CA USA; 7 Obesity Center, Nove de Julho Hospital, São Paulo, Brazil; 8 Eli Lilly and Company, Indianapolis, IN USA

**Keywords:** Obesity, Obesity, Patient education

## Abstract

Incretins have improved the management of obesity and its related complications, but maintaining these health benefits requires ongoing administration, which can be challenging. Orforglipron, a once-daily oral nonpeptide glucagon-like peptide-1 (GLP-1) receptor agonist, has demonstrated weight loss efficacy, improvements in cardiometabolic risk factors, and safety generally similar to injectable GLP-1 receptor agonists. Here this double-blind, placebo-controlled trial randomized participants previously treated with tirzepatide (cohort 1: *N* = 205) or semaglutide (cohort 2: *N* = 171) during the SURMOUNT-5 study to orforglipron once daily or placebo. Cohort 1 participants who achieved body weight plateau maintained a model-based estimate (MBE) of 74.7% (s.e.m. 4.05) of body weight reduction with orforglipron compared with an MBE of 49.2% (s.e.m. 3.92) with placebo, resulting in an estimated treatment difference of MBE 25.5% (95% confidence interval 14.5 to 36.5); *P* < 0.001; treatment-regimen estimand) at week 52. Cohort 2 participants who achieved body weight plateau maintained an MBE of 79.3% (s.e.m. 4.42) of body weight reduction with orforglipron compared with an MBE of 37.6% (s.e.m. 7.46) with placebo, resulting in an estimated treatment difference of MBE 41.7 (95% confidence interval 24.4 to 59.0); *P* < 0.001; treatment-regimen estimand) at week 52. All key secondary endpoints were met. The most common adverse events were gastrointestinal effects, which were mostly mild to moderate in severity. These data demonstrate orforglipron’s potential as a globally scalable option for minimizing weight changes after injectable therapy. Trial limitations include the absence of a comparator arm involving continued use of injectable obesity-management medications and the trial’s 1-year duration. ClinicalTrials.gov registration: NCT06584916.

## Abstract (web summary)

As presented at the European Congress on Obesity, this randomized, placebo-controlled trial demonstrates that oral orforglipron, a nonpeptide GLP-1 receptor agonist, preserves weight loss and cardiometabolic benefits achieved with injectable GLP-1 receptor agonist therapies, making it a viable oral maintenance strategy.

## Main

Obesity is a chronic, relapsing and treatable multifactorial disease^1^. Although safe and effective therapies are now available, persistence with existing injectable obesity management medications (OMMs) remains a barrier to weight loss maintenance^2^. Weight regain after discontinuing weight loss interventions, regardless of modality of weight loss, has been demonstrated, underscoring the need for sustained therapy to minimize changes in body weight and retain improvements in cardiometabolic parameters after weight reduction with the ultimate goal of preserving the accompanying benefits to overall health^3,4^.

Weight regain can be associated with negative overall health consequences, such as reversal of cardiometabolic improvement, impaired physical function (for example, increased pain) and an increase in psychological burden. During the weight maintenance phase, weight regain can occur secondary to decreased energy expenditure and increased energy efficiency, which together result in a net decrease in caloric needs^5–8^. Because of the negative consequences of weight regain and weight cycling, utilizing options to minimize weight change after intentional weight loss is a vital component for successful long-term health outcomes. As more options for safe and effective OMMs become available, exploring their role in preserving weight loss and minimizing weight change will help patients to receive evidence-based care across all phases of obesity management.

Orforglipron is a once-daily, nonpeptide GLP-1 receptor agonist (RA) currently under investigation for the treatment of obesity and type 2 diabetes. The ATTAIN global phase 3 registration program demonstrated clinically significant and meaningful body weight reduction along with improvements in markers of cardiometabolic disease, such as waist circumference, blood pressure, lipids and high‑sensitivity C‑reactive protein, in people with obesity, with and without type 2 diabetes, with an efficacy and safety profile generally similar to injectable incretin-based OMMs^3,9^. As a nonpeptide oral therapy, orforglipron has the potential to eliminate some individual patient barriers to injectable therapy (for example, resistance to injection, increased challenges with traveling, cold chain distribution and so on), can be taken without food or water restrictions and, if approved, can be globally scalable to meet the need for treatments worldwide. Literature suggests that aligning with patients’ preferences of attributes in treatment modalities increases the likelihood of patients persisting on therapy^10,11^. Thus, by addressing barriers that can be alleviated by orforglipron, generating evidence of its ability to maintain body weight reduction, along with safety data when switching to oral therapy, can help advance the field of obesity medicine.

The aim of this study was to assess the efficacy and safety of orforglipron compared with placebo, in maintenance of body weight reduction after 72 weeks of tirzepatide (cohort 1) or semaglutide (cohort 2) treatment in participants with obesity or body mass index (BMI) ≥27 kg/m^2^ with obesity-related complications who had previously participated in the SURMOUNT-5 study^12^. This is the initial clinical trial investigating the switch from injectable incretin-based therapy to an oral OMM for the maintenance of body weight reduction, and as a result, the study evaluated various endpoints, acknowledging that the clinical relevance of these endpoints may not be appreciated until trial completion. This study addressed important clinical questions, including a dosing strategy to transition patients from injectable to oral therapy, tolerability during the transition, and whether switching to oral therapy helps preserve the weight reduction achieved after injectable therapy compared with discontinuing therapy.

## Results

**Fig. 1:**
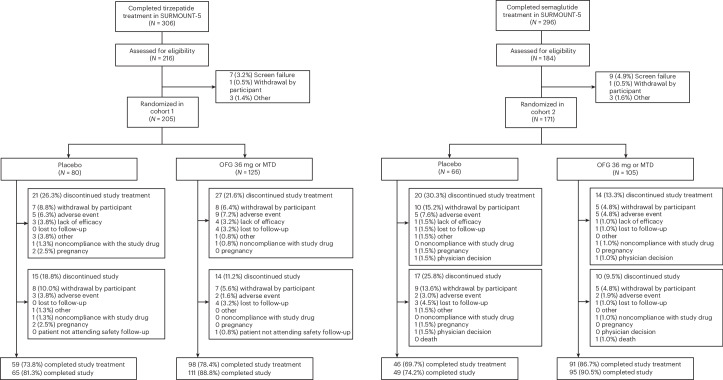
Trial disposition. CONSORT diagrams summarizing the design of the double-blind, placebo-controlled trial in which randomized participants previously treated with either tirzepatide (cohort 1: *N* = 205) or semaglutide (cohort 2: *N* = 171) during the SURMOUNT-5 study were treated with the GLP-1 receptor agonist orforglipron (OFG) once daily or with placebo.

ATTAIN-MAINTAIN screened 400 and enrolled 376 participants between 13 September 2024 and 21 November 2025, in 29 sites in the USA. A total of 205 participants in cohort 1 were randomized to treatment (*N* = 125 orforglipron 36 mg or maximum tolerated dose (MTD), *N* = 80 placebo) and 171 participants in cohort 2 were randomized to treatment (*N* = 105 orforglipron 36 mg or MTD, *N* = 66 placebo) (Fig. 1). This work reports data on the investigational orforglipron capsule formulation of 1, 3, 6, 12, 24 and 36 mg; the doses have been shown as the equivalent to tablet doses of 0.8, 2.5, 5.5, 9, 14.5 and 17.2 mg, respectively, which are approved in the USA^13^. Overall, 157 (76.6%) participants in cohort 1 and 137 (80.1%) participants in cohort 2 completed study treatment, and 176 (85.9%) completed the study in cohort 1 and 144 (84.2%) in cohort 2. Reasons for early study treatment and study discontinuations are shown in Fig. 1. Table 1 presents the demographic and baseline characteristics of the participants. In cohort 1, the mean age of the participants was 48.5 years; most were female (62.9%) and white (73.8%) and had a mean body weight of 90.1 kg. In cohort 2, the mean age was 48.6 years; most participants were female (68.4%) and white (75.9%) and had a mean body weight of 94.4 kg.

**Table 1:** Demographics and clinical characteristics of the participants at baseline

	Cohort 1 (tirzepatide group from SURMOUNT-5)			Cohort 2 (semaglutide group from SURMOUNT-5)		
	Orforglipron *N* = 125	Placebo *N* = 80	Total *N* = 205	Orforglipron *N* = 105	Placebo *N* = 66	Total *N* = 171
Age, years	49.0 (12.6)	47.7 (11.8)	48.5 (12.2)	48.6 (11.8)	48.7 (13.5)	48.6 (12.4)
Female sex, *n* (%)	79 (63.2)	50 (62.5)	129 (62.9)	73 (69.5)	44 (66.7)	117 (68.4)
Race^a^, *n* (%)						
American Indian or Alaska Native	1 (0.8)	2 (2.5)	3 (1.5)	–	–	–
Asian	1 (0.8)	4 (5.1)	5 (2.5)	1 (1.0)	2 (3.0)	3 (1.8)
Black or African American	26 (21.1)	16 (20.3)	42 (20.8)	27 (26.0)	9 (13.6)	36 (21.2)
White	94 (76.4)	55 (69.6)	149 (73.8)	74 (71.2)	55 (83.3)	129 (75.9)
Multiple	1 (0.8)	2 (2.5)	3 (1.5)	2 (1.9)	0 (0)	2 (1.2)
Duration of obesity, years	19.0 (12.6)	17.3 (9.6)	18.3 (11.5)	16.2 (10.3)	16.2 (11.4)	16.2 (10.7)
Body weight, kg	90.8 (23.5)	88.8 (21.5)	90.1 (22.7)	95.0 (25.7)	93.4 (25.6)	94.4 (25.6)
BMI, kg/m^2^	31.6 (6.8)	30.7 (6.0)	31.2 (6.5)	33.2 (7.6)	33.1 (7.5)	33.2 (7.5)
BMI category, *n* (%)						
<30	64 (51.2)	42 (52.5)	106 (51.7)	37 (35.2)	28 (42.4)	65 (38.0)
≥30 to <35	29 (23.2)	20 (25)	49 (23.9)	40 (38.1)	16 (24.2)	56 (32.7)
≥35 to <40	19 (15.2)	12 (15)	31 (15.1)	11 (10.5)	11 (16.7)	22 (12.9)
≥40	13 (10.4)	6 (7.5)	19 (9.3)	17 (16.2)	11 (16.7)	28 (16.4)
Waist circumference, cm	100.3 (16.6)	98.9 (15.3)	99.8 (16.1)	103.4 (18.7)	102.2 (18.4)	103.0 (18.6)
Blood pressure, mm Hg						
Systolic	113.5 (12.8)	116.0 (13.9)	114.5 (13.2)	117.7 (12.6)	115.8 (13.9)	117.0 (13.1)
Diastolic	74.7 (8.1)	75.3 (9.3)	74.9 (8.6)	77.6 (8.6)	75.9 (7.9)	76.9 (8.4)
Pulse, bpm	72.0 (8.5)	71.3 (10.2)	71.7 (9.2)	72.1 (8.9)	72.2 (9.4)	72.2 (9.0)
Lipid parameters, mg dl^−1^						
Triglycerides	84.6 (39.0)	95.9 (57.0)	88.9 (46.9)	95.9 (44.7)	92.1 (48.2)	94.4 (46.0)
Total cholesterol	173.0 (33.9)	180.2 (38.1)	175.8 (35.7)	181.2 (35.1)	175.1 (29.6)	178.8 (33.1)
HDL cholesterol	55.8 (13.9)	54.5 (11.7)	55.3 (13.1)	53.3 (12.2)	53.9 (13.4)	53.5 (12.7)
Non-HDL cholesterol	117.3 (30.5)	125.4 (37.6)	120.5 (33.7)	127.7 (32.2)	121.0 (28.8)	125.1 (31.0)
LDL cholesterol	100.3 (28.1)	106.5 (32.2)	102.7 (29.8)	108.7 (29.6)	102.7 (25.6)	106.4 (28.2)
VLDL cholesterol	16.9 (7.8)	19.2 (11.4)	17.8 (9.4)	19.2 (8.9)	18.4 (9.7)	18.9 (9.2)
Prediabetes, *n* (%)	6 (4.8)	6 (7.5)	12 (5.9)	13 (12.4)	13 (19.7)	26 (15.2)
HbA1c, %	5.2 (0.3)	5.1 (0.3)	5.1 (0.3)	5.2 (0.4)	5.3 (0.4)	5.2 (0.4)
HbA1c, mmol mol^−1^	32.9 (3.3)	32.3 (3.5)	32.6 (3.4)	33.3 (3.9)	33.9 (4.1)	33.6 (4.0)
eGFR^b^, ml min^−1^ per 1.73 m^2^	94.1 (19.7)	94.8 (16.2)	94.4 (18.4)	91.9 (21.0)	94.6 (18.8)	92.9 (20.2)
Fasting glucose, mg dl^−1^	82.6 (8.3)	82.5 (7.7)	82.6 (8.0)	84.0 (9.6)	83.3 (8.7)	83.7 (9.3)
Fasting insulin, pmol l^−1^	72.4 (50.5)	66.8 (42.9)	70.2 (47.7)	117.1 (138.5)	85.3 (58.4)	104.9 (115.2)
Number of obesity-related complications^c^, *n* (%)						
0	29 (23.4)	14 (17.5)	43 (21.1)	12 (11.4)	10 (15.2)	22 (12.9)
1	29 (23.4)	22 (27.5)	51 (25.0)	29 (27.6)	18 (27.3)	47 (27.5)
2	19 (15.3)	13 (16.3)	32 (15.7)	26 (24.8)	15 (22.7)	41 (24.0)
3	15 (12.1)	9 (11.3)	24 (11.8)	18 (17.1)	12 (18.2)	30 (17.5)
4	9 (7.3)	11 (13.8)	20 (9.8)	13 (12.4)	5 (7.6)	18 (10.5)
≥5	23 (18.5)	11 (13.8)	34 (16.7)	7 (6.7)	6 (9.1)	13 (7.6)

Data are mean ± s.d. or *n* (%). *N*, number of participants in the analysis population; *n*, number of participants in the specified category; bpm, beats per minute; HbA1c, glycated hemoglobin; HDL, high-density lipoprotein; LDL, low-density lipoprotein; VLDL, very-low-density lipoprotein.

^a^Race or ethnicity was reported by the participants.

^b^The value of the estimated glomerular filtration rate (eGFR) was calculated according to the serum cystatin C-based Chronic Kidney Disease Epidemiology Collaboration (CKD-EPI) equation.

^c^Obesity-related complications were assessed through a review of medical history.

### Cohort 1: tirzepatide to orforglipron (MTD) versus placebo

**Extended Data Fig. 1:**
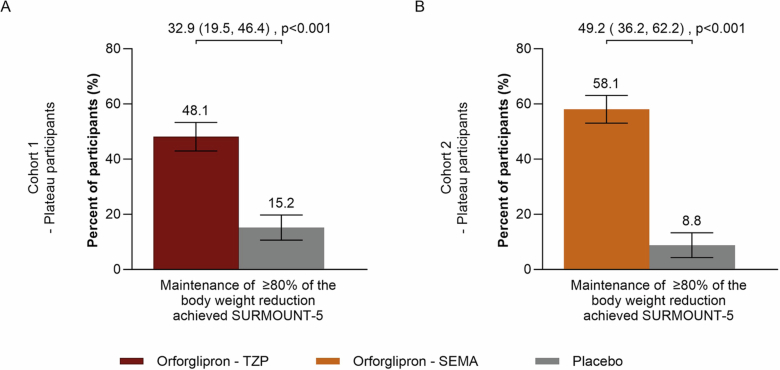
Assessment of maintenance of ≥80% body weight reduction achieved. Key secondary endpoints of assessment of maintenance of ≥80% body weight reduction achieved. Data presented as MBE (SEM). OFG vs. PBO: p-values are for risk difference vs. PBO. **A**.) Cohort 1 maintenance of ≥80% body weight reduction achieved during SURMOUNT-5. OFG N = 85; Placebo N = 57. Efficacy estimand. **B**.) Cohort 2 maintenance of ≥80% body weight reduction achieved during SURMOUNT-5. OFG N = 87; Placebo N = 43. Efficacy estimand. Abbreviations: BW = body weight; OFG = orforglipron, PBO = placebo. Source data

For the primary endpoint with the modified treatment-regimen estimand, participants in cohort 1 who achieved body weight plateau (<5% body weight change from weeks 60 to 72 in the SURMOUNT-5 study) maintained 74.7% (s.e.m. 4.05) of body weight reduction with orforglipron compared with 49.2% (s.e.m. 3.92) with placebo, with an estimated treatment difference (ETD) relative to placebo of 25.5% (95% confidence interval (CI) 14.5 to 36.5); *P* < 0.001) at week 52. Key secondary endpoints in the modified treatment-regimen estimand, in cohort 1 participants who achieved a body weight plateau, 43.7% (s.e.m. 4.98) of participants treated with orforglipron maintained ≥80% of the body weight reduction achieved during SURMOUNT-5 compared with 16.4% (s.e.m. 4.60) with placebo, with a risk difference to placebo of 27.3 (95% CI 14.1 to 40.6); *P* < 0.001 (efficacy estimand data shown in Extended Data Fig. 1a). All participants in cohort 1 maintained 74.4% (s.e.m. 3.63) of body weight reduction with orforglipron and 49.7% (s.e.m. 3.53) with placebo, with an ETD relative to placebo of 24.7 percentage points (95% CI 14.9 to 34.5; *P* < 0.001) with orforglipron treatment. All participants in cohort 1 had a mean MBE percent change from SURMOUNT-5 baseline body weight of –16.5% (s.e.m. 0.90) with orforglipron and 12.6% (s.e.m. 0.91) with placebo with an ETD relative to placebo of –3.9 percentage points (95% CI –6.1 to –1.8; *P* < 0.001).

**Fig. 2:**
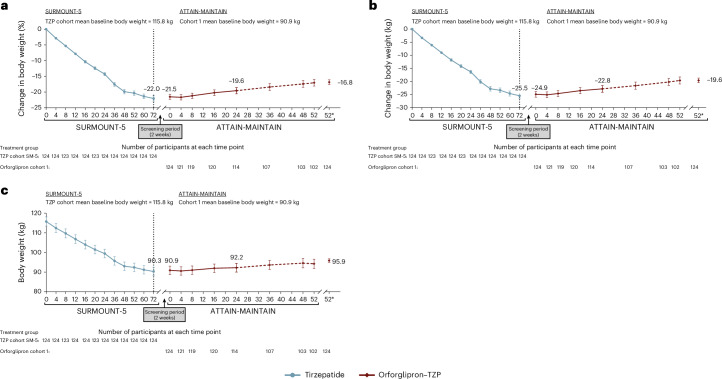
Weight change from SURMOUNT-5 to ATTAIN-MAINTAIN in cohort 1. Sensitivity analysis of weight change from SURMOUNT-5 (SM-5) to ATTAIN-MAINTAIN for cohort 1 (tirzepatide (TZP) to randomized control trial). **a**, Cohort 1: percent change in body weight from SURMOUNT-5 to ATTAIN-MAINTAIN. Observed data and efficacy estimand. **b**, Cohort 1: mean change in body weight from SURMOUNT-5 to ATTAIN-MAINTAIN. Observed data and efficacy estimand. **c**, Cohort 1: absolute body weight from SURMOUNT-5 to ATTAIN-MAINTAIN. Observed data and efficacy estimand. Data presented in line plots are observed mean (s.e.m.). *Data are MBE (s.e.m.) for the mean actual value of body weight based on MMRM analysis. Dashes beginning at week 24 include participants eligible for rescue therapy. Data in ATTAIN-MAINTAIN after rescue orforglipron were imputed with worst value observed before start of rescue. All other data are MBE (s.e.m.) of the modified intent-to-treat population. Source data

In additional sensitivity analyses based on the efficacy estimand, participants in cohort 1 randomized only to orforglipron in ATTAIN-MAINTAIN had a mean baseline body weight of 115.8 kg (s.e.m. 2.23) in SURMOUNT-5. At the start of the ATTAIN-MAINTAIN study, they had a baseline body weight of 90.9 kg (s.e.m. 2.12) with a reduction of 21.5% (s.e.m. 0.83). Participants in cohort 1 had a mean MBE body weight reduction of 16.8% (s.e.m. 0.76) (Fig. 2a) and absolute body weight reduction of 19.6 kg (s.e.m. 0.91) (Fig. 2b) from the beginning of SURMOUNT-5 to the end of ATTAIN-MAINTAIN. This represents a difference in percent weight changes of approximately 5% and an average difference in weight of approximately 5 kg from baseline. For these participants, the mean body weight (MBE) at week 52 was 95.9 kg (s.e.m. 0.91) with orforglipron (Fig. 2c). Participants in cohort 1 at 24 weeks, before rescue therapy eligibility per study protocol, had a mean MBE body weight reduction of −19.0% (s.e.m. 0.68) from the beginning of SURMOUNT-5, with a mean MBE body weight of 93.4 kg (s.e.m. 0.79). This preserved the previously achieved weight reduction with an average difference of approximately 3 kg. Among all participants in cohort 1 randomized only to orforglipron who achieved 15% or more body weight reduction in SURMOUNT-5, 63.7% (s.e.m. 5.03) maintained 15% or more body weight reduction after 52 weeks of treatment with orforglipron.

**Extended Data Fig. 2:**
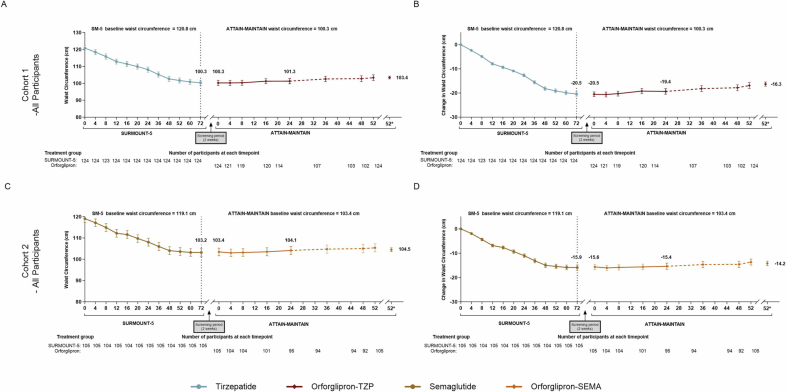
Waist circumference change from SURMOUNT-5 to ATTAIN-MAINTAIN. Sensitivity analysis of waist circumference change from SURMOUNT-5 to ATTAIN-MAINTAIN. **A**) Cohort 1: Absolute waist circumference from SM-5 to ATTAIN-MAINTAIN. Observed data and efficacy estimand. **B**) Cohort 1: Mean change in waist circumference from SM-5 to ATTAIN-MAINTAIN. Observed data and efficacy estimand. **C**) Cohort 2: Absolute waist circumference from SM-5 to ATTAIN-MAINTAIN. Observed data and efficacy estimand. **D**) Cohort 2: Mean change in waist circumference from SM-5 to ATTAIN-MAINTAIN. Observed data and efficacy estimand. Data presented in line plots are observed mean (SEM). *Data are MBE (SEM) for the mean actual value of waist circumference based on MMRM analysis. Dashes beginning at week 24 include participants eligible for rescue therapy. Data in ATTAIN-MAINTAIN after rescue orforglipron were imputed with worst value observed prior to start of rescue. All other data are MBE (SEM) of mITT population. ICE=intercurrent event; MBE=model-based estimate; mITT=modified intent-to-treat; MMRM=mixed model for repeated measures; MTD=maximum tolerated dose; OFG=orforglipron; PBO=placebo; SEM=standard error of the mean; SEMA=semaglutide, SM-5 = SURMOUNT-5, TZP=tirzepatide. Source data

Participants in cohort 1 randomized only to orforglipron in ATTAIN-MAINTAIN had a mean baseline waist circumference of 120.8 cm (s.e.m. 1.48) in SURMOUNT-5. At the start of the ATTAIN-MAINTAIN study, they had a mean waist circumference of 100.3 cm (s.e.m. 1.50) with a decrease in mean waist circumference of 20.5 cm (s.e.m. 0.95) (Extended Data Fig. 2a,b). Based on the efficacy estimand, participants in cohort 1 had a mean decrease in waist circumference of 16.3 (s.e.m. 0.86) cm from the beginning of SURMOUNT-5, resulting in an MBE waist circumference of 103.4 cm (s.e.m. 0.86) with orforglipron. This preserved their previously achieved decrease in waist circumference with an average change from randomization of approximately 3 cm (Extended Data Fig. 2a).

**Extended Data Fig. 3:**
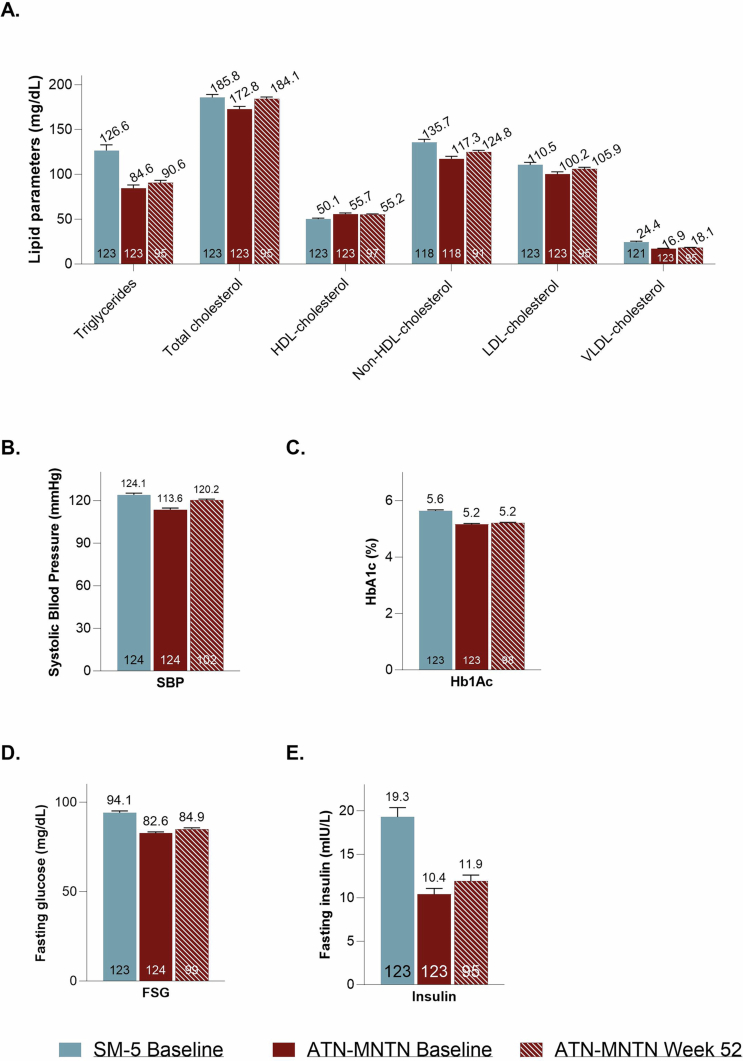
Cardiometabolic Parameter changes from SURMOUNT-5 to ATTAIN-MAINTAIN in Cohort 1 (Tirzepatide to RCT). Additional sensitivity analyses of changes in cardiometabolic parameters. Cohort 1 cardiometabolic parameters. Within bar values = number of participants. **A**.) Lipid parameters mean (SEM) and at Week 52 data MBE (SEM). **B**.) Systolic blood pressure, mean (SEM) and at Week 52 data as MBE (SEM). **C**.) HbA1c mean (SEM) and at Week 52 data MBE (SEM). **D**.) Fasting glucose mean (SEM) and at Week 52 data MBE (SEM). **E**.) Fasting insulin mean (SEM) and at Week 52 data MBE (SEM). Abbreviations: SM-5 = SURMOUNT-5; ATN-MNTN = ATTAIN-MAINTAIN; HDL=high density lipoprotein; LDL=low density lipoprotein; VLDL=very low density lipoprotein; SBP=systolic blood pressure; SEM=standard error of the mean; MBE=model based estimate. Source data

In addition, other cardiometabolic risk factors demonstrated similar preservation of reductions at the end of ATTAIN-MAINTAIN. As an example, in cohort 1, participants subsequently randomized to orforglipron had a mean baseline HbA1c of 5.6% at the beginning of SURMOUNT-5. At the beginning of ATTAIN-MAINTAIN, after weight reduction with injectables, the HbA1c improved to a mean of 5.2%. At 52 weeks, the mean HbA1c remained at 5.2% and retained the improvements in this marker after switching to oral orforglipron. Similar trends were observed in insulin levels, fasting serum glucose (FSG), markers of lipids such as triglycerides and non-HDL, and systolic blood pressure (Extended Data Fig. 3a–e).

### Cohort 2: semaglutide to orforglipron (MTD) versus placebo

For the primary endpoint with modified treatment-regimen estimand, participants in cohort 2 who achieved body weight plateau maintained 79.3% (s.e.m. 4.42) of body weight reduction with orforglipron compared with 37.6% (s.e.m. 7.46) with placebo, with an ETD relative to placebo of 41.7 (95% CI 24.4 to 59.0); *P* < 0.001) at week 52. Among key secondary endpoints in cohort 2, participants who achieved a body weight plateau, 55.0% (s.e.m. 5.08) of those treated with orforglipron maintained ≥80% of the body weight reduction achieved during SURMOUNT-5, compared with 6.9% (s.e.m. 3.77) with placebo, corresponding to a risk difference of 48.1 (95% CI 35.6 to 60.5; *P* < 0.001) (efficacy estimand data shown in Extended Data Fig. 1b). All participants in cohort 2 maintained 85.9% (s.e.m. 4.60) of body weight reduction with orforglipron and 40.2% (s.e.m. 7.87) with placebo, with an ETD relative to placebo of 45.6 percentage points (95% CI 28.3 to 63.0; *P* < 0.001) with orforglipron treatment. All participants in cohort 2 had a mean MBE percent change from SURMOUNT-5 baseline body weight of –14.9% (s.e.m. 0.94) with orforglipron and –7.9% (SE 0.81) with placebo, with an ETD relative to placebo of –7.0 percentage points (95% CI –9.1 to –5.0; *P* < 0.001).

**Fig. 3:**
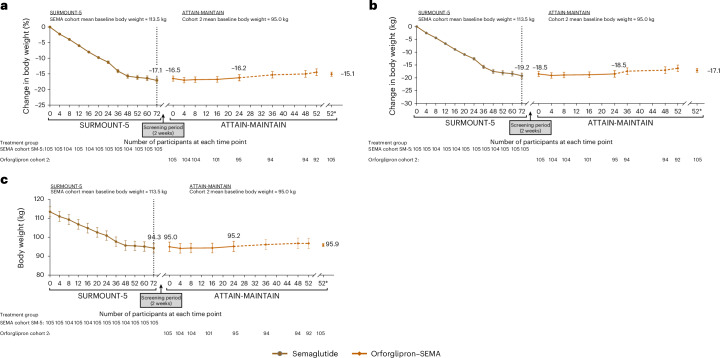
Weight change from SURMOUNT-5 to ATTAIN-MAINTAIN in cohort 2. Sensitivity analysis of weight change from SURMOUNT-5 to ATTAIN-MAINTAIN for cohort 2 (semaglutide (SEMA) to randomized control trial). **a**, Cohort 2: percent change in body weight from SURMOUNT-5 to ATTAIN-MAINTAIN. Observed data and efficacy estimand. **b**, Cohort 2: mean change in body weight from SURMOUNT-5 to ATTAIN-MAINTAIN. Observed data and efficacy estimand. **c**, Cohort 2: absolute body weight from SURMOUNT-5 to ATTAIN-MAINTAIN. Observed data and efficacy estimand. Data presented in line plots are observed mean (s.e.m.). *Data are MBE (s.e.m.) for the mean actual value of body weight based on MMRM analysis. Dashes beginning at week 24 include participants eligible for rescue therapy. Data in ATTAIN-MAINTAIN after rescue orforglipron were imputed with worst value observed before start of rescue. All other data are MBE (s.e.m.) of the modified intent-to-treat population. Source data

In additional sensitivity analyses of the efficacy estimand, participants in cohort 2 randomized only to orforglipron in ATTAIN-MAINTAIN had a mean baseline body weight of 113.5 kg (s.e.m. 2.64) in SURMOUNT-5. At the start of the ATTAIN-MAINTAIN study, they had a baseline body weight of 95.0 kg (s.e.m. 2.51) with a reduction of 16.5% (s.e.m. 0.81). Participants in cohort 2 had a mean MBE body weight reduction of 15.1% (s.e.m. 0.67) (Fig. 3a) and absolute body weight reduction of 17.1 kg (Fig. 3b) from the beginning of SURMOUNT-5 to the end of ATTAIN-MAINTAIN. This represents a difference in percent weight change of approximately 1% and an average difference in weight of approximately 1 kg from baseline. For these participants, the mean body weight (MBE) at week 52 was 95.9 kg (s.e.m. 0.76) with orforglipron (Fig. 3c). Participants in cohort 2 at 24 weeks, before rescue therapy eligibility per study protocol, had a mean MBE body weight reduction of 16.3% (s.e.m. 0.56) from the beginning of SURMOUNT-5, with a mean MBE body weight of 94.5 kg (s.e.m. 0.68). This preserved all of the previously achieved weight reduction. Among all participants in cohort 2 randomized only to orforglipron who achieved 15% or more body weight reduction in SURMOUNT-5, 69.5% (s.e.m. 5.79) maintained 15% or more body weight reduction after 52 weeks of treatment with orforglipron.

Participants in cohort 2 randomized only to orforglipron in ATTAIN-MAINTAIN had a mean baseline waist circumference of 119.1 cm (s.e.m. 1.77) in SURMOUNT-5. At the start of the ATTAIN-MAINTAIN study, they had a waist circumference of 103.4 (s.e.m. 1.83) cm with a decrease in mean waist circumference of 15.6 (s.e.m. 1.09) cm (Extended Data Fig. 2c,d). Based on the efficacy estimand, participants in cohort 2 had a mean decrease in waist circumference of 14.2 (s.e.m. 0.90) cm from the beginning of SURMOUNT-5, resulting in an MBE waist circumference of 104.5 (s.e.m. 0.90) cm with orforglipron. This preserved their previously achieved decrease in waist circumference with an average change from randomization of approximately 1 cm (Extended Data Fig. 2c).

**Extended Data Fig. 4:**
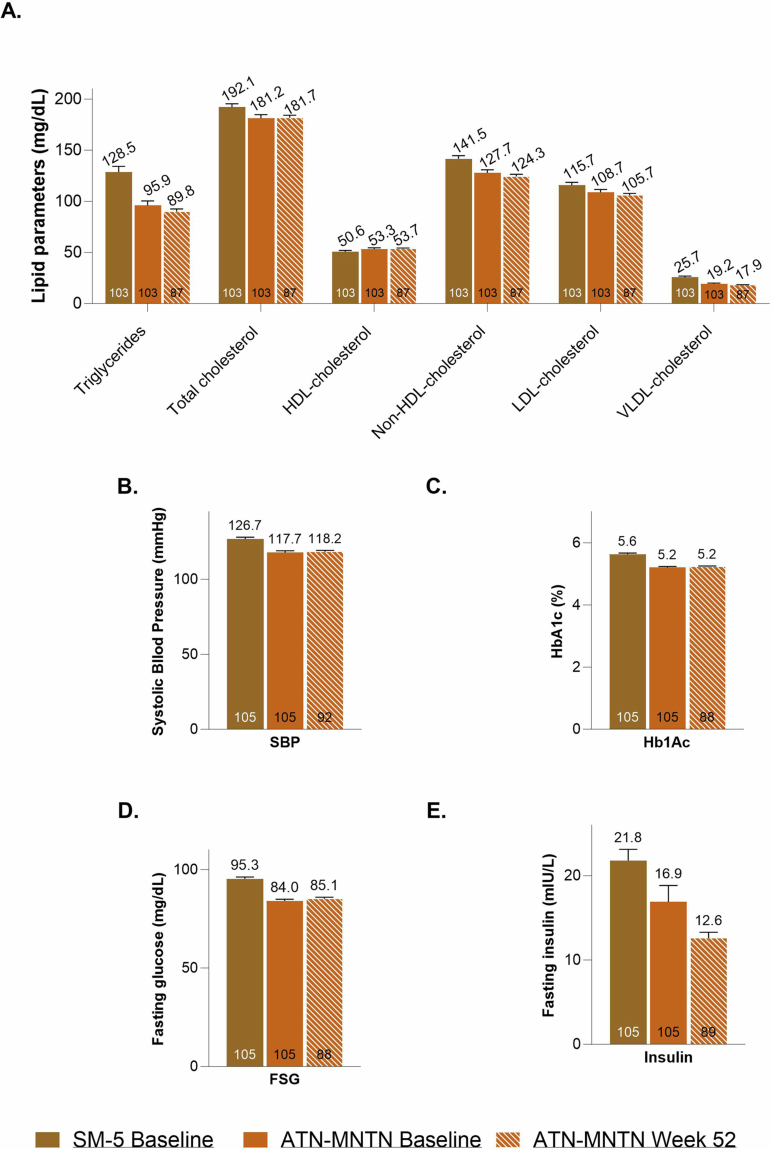
Cardiometabolic Parameter changes from SURMOUNT-5 to ATTAIN-MAINTAIN in Cohort 2 (Semaglutide to RCT). Additional sensitivity analysis of changes in cardiometabolic parameters. Cohort 2 cardiometabolic parameters. Within bar values = number of participants. **A**.) Lipid parameters mean (SEM) and at Week 52 data MBE (SEM). **B**.) Systolic blood pressure, mean (SEM) and at Week 52 data as MBE (SEM). **C**.) HbA1c mean (SEM) and at Week 52 data MBE (SEM).**D**.) Fasting glucose mean (SEM) and at Week 52 data MBE (SEM). **E**.) Fasting insulin mean (SEM) and at Week 52 data MBE (SEM). Abbreviations: SM-5 = SURMOUNT-5; ATN-MTN = ATTAIN-MAINTAIN; HDL=high density lipoprotein; LDL=low density lipoprotein; VLDL=very low density lipoprotein; SBP=systolic blood pressure; SEM=standard error of the mean; MBE=model based estimate. Source data

In addition, other cardiometabolic risk factors demonstrated similar preservation of reductions at the end of ATTAIN-MAINTAIN. As an example, in cohort 2 participants subsequently randomized to orforglipron had a mean baseline A1c of 5.6% at the beginning of SURMOUNT-5. At the beginning of ATTAIN-MAINTAIN, after weight reduction with injectables, the A1c improved to a mean of 5.2%. At 52 weeks, the mean A1c remained at 5.2% and retained the improvements in this marker after switching to oral orforglipron. Similar trends were observed in insulin levels, FSG, markers of lipids such as triglycerides and non-HDL, and systolic blood pressure (Extended Data Fig. 4a–e).

### Rescue therapy

Starting at week 24, participants on placebo who regained 50% or more of the weight loss achieved during the SURMOUNT-5 trial were initiated on orforglipron as rescue therapy. Participants began taking rescue orforglipron 1 mg orally each day, with dose escalation every 4 weeks until the MTD was achieved as determined by the interactive web response system. For participants taking orforglipron 24 mg, they were allowed to re-escalate to orforglipron 36 mg using the interactive web response system to maintain blinding. Other oral OMMs were not permitted as rescue therapy in the study. In those participants who had ≥50% weight regain in cohort 1 and cohort 2, 39 (65.0%) and 42 (64.6%) received rescue orforglipron therapy in the placebo group, respectively. In those randomized to the orforglipron group, two participants in cohort 1 and one participant in cohort 2 were escalated from a dose of 24 mg to 36 mg of orforglipron. Furthermore, at 52 weeks, only 25 participants (31.3%) from cohort 1 and 12 participants (18.2%) from cohort 2 who were originally randomized to placebo completed treatment without receiving active therapy (either as rescue or other OMMs).

### Safety

**Extended Data Fig. 5:**
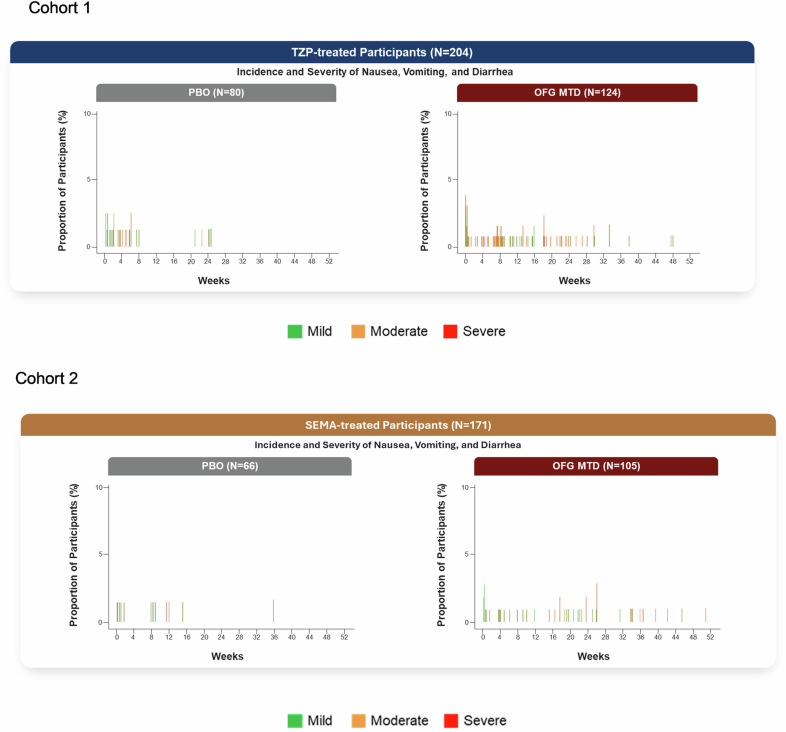
Incidence of nausea, vomiting, diarrhea, over time. Sensitivity analysis of incidence of treatment-emergent nausea, vomiting, diarrhea. Proportions are based on the number of participants at risk. Vomiting includes preferred terms of vomiting and vomiting projectile; diarrhea includes preferred terms of diarrhea and frequent bowel movements; constipation includes preferred terms of constipation, infrequent bowel movements, and feces hard. Source data

The proportion of participants reporting any adverse events (AEs) and the number of reported serious adverse events (SAEs) were similar between arms (Table 2; see additional safety data in Extended Data Tables 1 and 2). Across both the cohorts, study drug discontinuations owing to AEs were 4.8% to 7.3% with orforglipron MTD. The most frequently reported AEs with orforglipron were gastrointestinal disorders of nausea, constipation, vomiting or diarrhea. The overall incidence of GI AEs including nausea, vomiting or diarrhea in the first 4 weeks of the trial for both cohorts was 10.5% and 9.5% in the tirzepatide and semaglutide cohorts, respectively. Most GI AEs were mild to moderate in severity, and there were no dose de-escalations within the first 4 weeks when transitioning from injectable therapy directly to 12 mg of orforglipron (Extended Data Fig. 5).

**Table 2:** AEs in participants before rescue safety set

Cohort 1 (tirzepatide)			
	Orforglipron *N* = 124	Placebo *N* = 80	Total *N* = 204
Any AE emerging during treatment	85 (68.5)	47 (58.8)	132 (64.7)
Deaths^a^	0	0	0
SAEs	3 (2.4)	0	3 (1.5)
AEs leading to discontinuation of study treatment	9 (7.3)	2 (2.5)	11 (5.4)
Gastrointestinal disorders leading to discontinuation of study treatment	3 (2.4)	0	3 (1.5)
AEs that emerged during treatment and occurred in ≥5% of participants in any treatment group			
Constipation	15 (12.1)	4 (5.0)	19 (9.3)
Nausea	29 (23.4)	4 (5.0)	33 (16.2)
Diarrhea	9 (7.3)	9 (11.2)	18 (8.8)
Vomiting	14 (11.3)	1 (1.2)	15 (7.4)
Fatigue	7 (5.6)	0	7 (3.4)
Upper respiratory tract infection	8 (6.5)	4 (5.0)	12 (5.9)
Abdominal distension	7 (5.6)	5 (6.2)	12 (5.9)
Hypertension	3 (2.4)	4 (5.0)	7 (3.4)
Influenza	4 (3.2)	5 (6.2)	9 (4.4)
Flatulence	2 (1.6)	5 (6.2)	7 (3.4)
AEs of special interest emerging during treatment			
Hepatic elevations^b,e^	0	0	0
Malignancies^b^	1 (0.8)	0	1 (0.5)
Pancreatitis (adjudication-confirmed)^c^	1 (0.8)	0	1 (0.5)
MACE (adjudication-confirmed)^b^	0	0	0
Cardiac disorders^d^	0	0	0
Gastrointestinal events^b^	3 (2.4)	0	3 (1.5)
Gallbladder disease^b^	0	0	0
Acute renal events^b^	0	0	0
Major depressive disorder/suicidal ideation or behavior^b^	0	0	0
Hypersensitivity^b^	0	0	0
Other AEs emerging during treatment			
Cholelithiasis	1 (0.8)	0	1 (0.5)

Cohort 2 (semaglutide)			
	**Orforglipron** ***N*** = **105**	**Placebo** ***N*** = **66**	**Total** ***N*** = **171**
Any AE emerging during treatment	65 (61.9)	35 (53.0)	100 (58.5)
Deaths^a^	1 (1.0)	0	1 (0.6)
SAEs	1 (1.0)	1 (1.5)	2 (1.2)
AEs leading to discontinuation of study treatment	5 (4.8)	2 (3.0)	7 (4.1)
Gastrointestinal disorders leading to discontinuation of study treatment	1 (1.0)	0	1 (0.6)
AEs that emerged during treatment and occurred in ≥5% of participants in any treatment group			
Constipation	15 (14.3)	2 (3.0)	17 (9.9)
Nausea	14 (13.3)	2 (3.0)	16 (9.4)
Diarrhea	8 (7.6)	2 (3.0)	10 (5.8)
Vomiting	5 (4.8)	4 (6.1)	9 (5.3)
Upper respiratory tract infection	8 (7.6)	7 (10.6)	15 (8.8)
Headache	8 (7.6)	1 (1.5)	9 (5.3)
Increased appetite	1 (1.0)	4 (6.1)	5 (2.9)
AEs of special interest emerging during treatment			
Hepatic elevations^b,e^	0	1 (1.5)	1 (0.6)
Malignancies^b^	0	0	0
Pancreatitis (adjudication-confirmed)^c^	0	0	0
MACE (adjudication-confirmed)^b^	1 (1.0)	0	1 (0.6)
Cardiac disorders^d^	0	0	0
Gastrointestinal events^b^	2 (1.9)	1 (1.5)	3 (1.8)
Gallbladder disease^b^	0	0	0
Acute renal events^b^	0	0	0
Major depressive disorder/suicidal ideation or behavior^b^	0	0	0
Hypersensitivity^b^	0	0	0
Other AEs emerging during treatment			
Cholelithiasis	0	1 (1.5)	1 (0.6)

Data are number of participants (%). MACE, major adverse cardiovascular event.

^a^Cause of death was reported as follows: cardiovascular/stroke in cohort 2 participant randomized to orforglipron; Deaths were also counted as SAEs and discontinuation owing to AEs.

^b^Events that were classified as severe AEs or SAEs.

^c^Additional information provided in Extended Data Table 2.

^d^Events that were classified as severe or serious arrhythmias and cardiac conduction disorders.

^e^Defined as elevated transaminases, bilirubin, gamma-glutamyl transferase, AST, ALT, liver function or hepatic steatosis by study investigator. See additional AST/ALT data in Extended Data Table 3.

SAEs were reported by three participants in the orforglipron group in cohort 1 and one participant each in the orforglipron and placebo group in cohort 2, accounting for <2% of the total group (Table 2). One case of mild pancreatitis was confirmed through adjudication in the orforglipron treatment group in cohort 1. Among the participants treated with orforglipron in cohort 1, four participants had increased ALT levels greater than or equal to three times the upper limit of normal (ULN) and one participant in cohort 2 and one participant in the placebo group for both cohorts 1 and 2 experienced this elevation in ALT (Extended Data Table 3). In both cohorts 1 and 2, two participants receiving orforglipron had increased AST levels greater than or equal to three times the ULN. In the placebo group, one participant in cohort 2 experienced this elevation (Extended Data Table 3). Of the participants experiencing these aspartate aminotransferase (AST)/alanine aminotransferase (ALT) elevations, one participant in cohort 2 receiving placebo also experienced an increase of twice the ULN for total bilirubin. Overall, no liver safety signals were detected. One death was reported in the orforglipron group in cohort 2 and was not considered related to the study treatment.

## Discussion

We present a clinical trial exploring the switch from injectable to oral incretin therapy for the treatment of obesity. In ATTAIN-MAINTAIN, participants treated with orforglipron demonstrated significant and clinically meaningful maintenance of body weight reduction achieved with injectable therapy compared with placebo, with 75% and 79% in the orforglipron group versus 49% and 38% in the placebo group in cohorts 1 and 2, respectively. A key finding of this trial is that participants were better able to maintain the majority of weight loss achieved with injectable therapy when switched to orforglipron compared with placebo. As an initial trial examining switching to oral therapy for maintenance of weight reduction, and in the absence of a widely agreed upon definition or calculation to assess maintenance of weight reduction, several endpoints, including additional sensitivity analyses, were evaluated in this trial regarding maintenance and overall change. We observed that calculating maintenance based on the weight loss achieved in SURMOUNT-5 resulted in relatively high variability due to the limited sample size, which may disproportionately skew the mean and limit clarity and generalizability, particularly given that only 12 participants remained on placebo at week 52 without requiring rescue therapy or other OMMs in the semaglutide-to-placebo group. Thus, additional sensitivity analyses were conducted to provide data including absolute change in mean weight. Mean absolute change in weight may be an important complementary endpoint to facilitate a clearer and fuller understanding of maintenance for the clinical provider. Participants treated with orforglipron demonstrated average reductions in body weight from week 0 to 52 in ATTAIN-MAINTAIN of approximately 5 kg (5%) in cohort 1 and 1 kg (1%) in cohort 2.

Consistent with the orforglipron clinical trial program demonstrating an efficacy and safety profile generally similar to injectable GLP-1 RAs, orforglipron maintained nearly all the weight loss achieved on injectable semaglutide^9,14^. We hypothesized that patients would experience more weight regain on average when transitioning off high doses of the dual GIP/GLP-1 RA tirzepatide to a GLP-1 RA than when transitioning from a GLP-1 RA to a GLP-1 RA, but further exploration is necessary. A head-to-head clinical trial of injectable tirzepatide versus injectable semaglutide has already demonstrated superior weight loss with tirzepatide^12^. Even so, most participants transitioning from tirzepatide maintained the majority of their weight loss, particularly compared with placebo, and demonstrated minimal changes in waist circumference as a marker of visceral adiposity, along with sustained improvements in systolic blood pressure, lipid levels and glycemic parameters following weight reduction. For some patients, a small degree of weight regain while maintaining the majority of weight loss achieved with injectable therapy may be clinically acceptable, particularly if switching to a different therapy that has attributes that facilitate long-term persistence on therapy. The individual differences highlight the need for shared decision-making conversations across the entire obesity management journey. An additional finding is that, regardless of the initial intervention, switching to orforglipron resulted in both cohorts ending at the same body weight of 95.9 kg. This finding with orforglipron could suggest that there may be a biological component that controls the disease of obesity at this body weight. Further studies are needed to explore this potential biological control of obesity.

A common misconception among individuals with obesity, as well as some clinicians, is that OMMs can be discontinued after achieving initial weight loss. The approach of stopping medication after intentional weight loss can lead to weight cycling and loss of improvements in cardiometabolic health such as increased insulin resistance or blood pressure, as is seen when individuals stop therapy in other chronic diseases such as hypertension and dyslipidemia^3,15,16^. So far, injectable OMMs have proven to be highly efficacious and safe for use, but persistence on therapy remains challenging. This trial provides evidence of how to switch to oral OMM therapy and its ability to improve weight loss maintenance and could serve as a potential solution for persistence on therapy for those who wish to stop injectable therapy owing to patient preference, convenience, cost or cold storage requirements.

When the study was originally designed, it was uncertain whether tolerance to high doses of injectable OMMs used in SURMOUNT-5 would confer similar cross-tolerance to an oral nonpeptide agonist like orforglipron. To minimize the dose escalation period, we evaluated transitioning patients directly from injectable therapy to 12 mg of orforglipron (compared with starting doses of 1 mg evaluated in the registration program evaluating participants without recent GLP-1 RA exposure). This transition was generally well tolerated, with fewer than 5% of participants experiencing gastrointestinal AEs and no participants requiring a dose de-escalation in both cohort 1 and cohort 2 during the first 4 weeks of the trial. This tolerability profile raises the question of whether patients could transition directly from injectable incretin therapy to higher doses of orforglipron, potentially minimizing the dose-escalation period required to reach equipotent dosing. Although not explored here, this could be an area of future investigation to further optimize patient convenience and prescribing simplicity.

In our study, participants received rescue orforglipron if they experienced 50% or more weight regain while on placebo starting at 24 weeks post-randomization. The use of rescue orforglipron in this trial was a, patient-centric approach intended to mitigate both the physiological and psychological consequences of weight regain, considering the substantial weight reductions observed in SURMOUNT-5. This patient-centric approach did contribute to complexity when interpreting comparisons to placebo at the 52-week time point. In fact, as expected and demonstrated by results between weeks 24 and 52, the availability of rescue therapy largely prevented further increases in average weight across both cohorts. In both cohorts, the majority of participants who regained ≥50% of their weight reduction were from the placebo arm, which probably contributed to the high study completion rates observed in these arms (81.3% cohort 1, 74.2% cohort 2), despite the increasing availability of OMMs in the USA. This highlights the need for clinicians to educate patients on the importance of persistence on therapy in managing obesity as a chronic condition and to clarify the natural progression of the disease should therapy be halted.

Trial limitations include study sites located only in the USA and a predominantly white study population, although the study includes a more representative US population with a higher Black or African American and Hispanic or Latino population. Additional limitations were the lack of a comparator arm that included continuing injectable OMM and a trial duration of 1 year. Owing to the increasing availability of OMMs, there were participants who discontinued treatment to start available and approved medications, even before the 24 weeks when rescue therapy became an option for placebo. Trial strengths include the randomized, controlled study design, inclusion of healthy lifestyle counseling throughout the trial, and use of rescue orforglipron in case of significant (≥50%) weight regain to limit the potential harm associated with weight regain, although this does not allow for pure placebo versus treatment comparison after week 24. In our study, only 25 participants (31.3%) in cohort 1 and 12 participants (18.2%) in cohort 2 from the placebo arm remained on placebo through week 52 without requiring intervention to manage weight regain. In addition, this trial evaluates switching from injectable OMMs to oral therapy and provides evidence supporting the use of 12 mg of orforglipron as a starting dose after transitioning from injectable therapy.

Once-daily orforglipron minimized weight changes after body weight reduction achieved with tirzepatide or semaglutide for people living with obesity without type 2 diabetes. The results indicate that switching to orforglipron may be an effective approach for maintaining weight loss for those who do not continue injectable therapy.

## Methods

### Study design

The trial was conducted in compliance with the Declaration of Helsinki and the Good Clinical Practice guidelines of the International Council for Harmonization. The trial protocol was approved by the Advarra central institutional review board. A blind, independent, external committee adjudicated all deaths, pancreatic AEs and cardiovascular events. The completed trial is registered on ClinicalTrials.gov (NCT06584916).

ATTAIN-MAINTAIN consisted of two phase 3b randomized, double-blind, placebo-controlled cohorts conducted at 29 sites in the USA. The trial examined the maintenance of body weight reduction with treatment of orforglipron among participants who previously completed SURMOUNT-5 and received 72 weeks of either tirzepatide (cohort 1) or semaglutide (cohort 2). The tirzepatide and semaglutide treatment groups from the SURMOUNT-5 trial were treated independently and analyzed separately. ATTAIN-MAINTAIN builds upon the findings of the previously published SURMOUNT-5 trial^12^.

**Extended Data Fig. 6:**
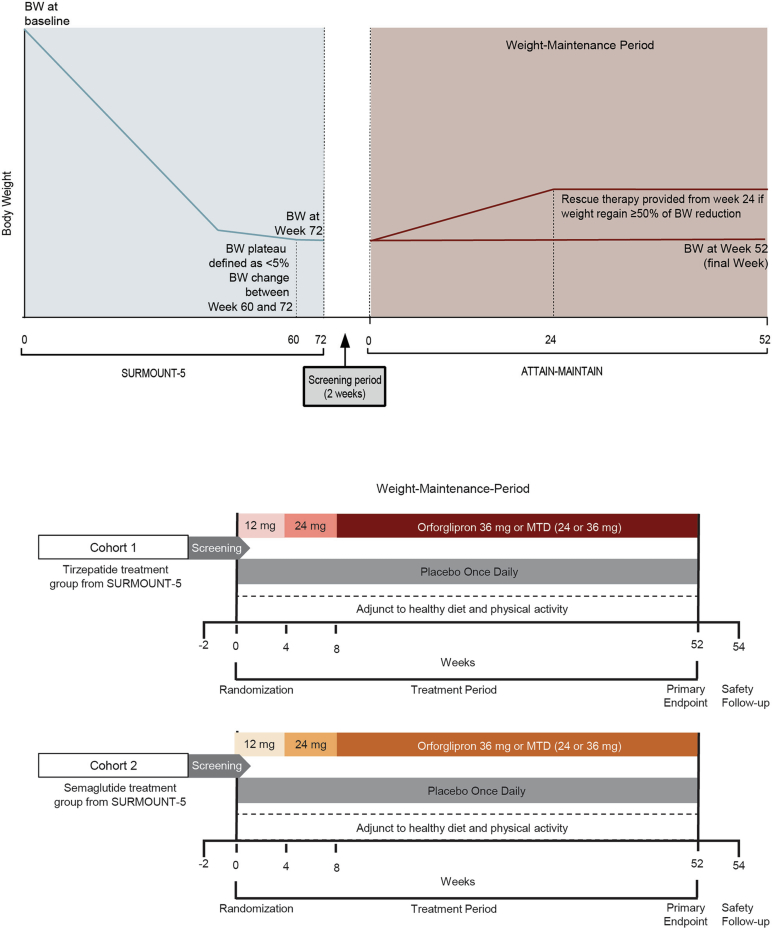
**Study design and schema**.

During SURMOUNT-5, participants were randomized to either tirzepatide MTD (10 mg or 15 mg) or semaglutide MTD (1.7 mg or 2.4 mg) with a total weight-loss period of 72 weeks^1^. After completion of SURMOUNT-5, eligible participants who provided written, informed consent for ATTAIN-MAINTAIN were randomized to either orforglipron or placebo (Extended Data Fig. 6). The study included a screening period of up to 2 weeks after completion of SURMOUNT-5, a 52-week treatment period and a 2-week safety follow-up. A list of study investigators is available in the Supplementary Information.

### Participants

Eligible adult participants completed the SURMOUNT-5 trial on study treatment and achieved a body weight reduction of at least 5% at week 72 with either tirzepatide or semaglutide. Exclusion criteria included a diagnosis of diabetes and a BMI of 22 kg/m^2^ or lower. A complete list of inclusion and exclusion criteria is provided in the Supplementary Information (Protocol Study Population 47). All participants provided signed informed consent before trial participation. Participants were screened and recruited irrespective of sex.

### Randomization and blinding

Participants were randomized in a 3:2 ratio to receive once-daily orforglipron (36 mg or MTD of 24 or 36 mg), or placebo, using a computer-generated random sequence from a Lilly interactive web response system. This work reports data on the investigational orforglipron capsule formulation of 1 mg, 3 mg, 6 mg, 12 mg, 24 mg and 36 mg; the doses have been shown as the equivalent to the tablet doses of 0.8 mg, 2.5 mg, 5.5 mg, 9 mg, 14.5 mg and 17.2 mg, respectively, which are approved in the USA^13^. Randomization was stratified by achievement of plateau at week 72 in SURMOUNT-5, sex and percent weight loss at week 72 of SURMOUNT-5 (<20%, ≥20%). Plateau was defined as a weight change of less than 5% between weeks 60 and 72 in the SURMOUNT-5 study. Study investigators, site staff, clinical monitors and participants were blinded to the study intervention until study completion.

### Study interventions

Following randomization, participants initiated blinded orforglipron 12 mg or a matching placebo once daily. The first study intervention dose was ideally administered within 14 days of the last dose of SURMOUNT-5 study drug. Participants randomized to orforglipron increased the dose every 4 weeks until reaching 36 mg or MTD. The maintenance dose of the study intervention was continued for the remainder of the 52-week study. Starting at week 24, participants on placebo who regained 50% or more of the weight loss achieved during the SURMOUNT-5 trial were initiated on orforglipron as rescue therapy. No restrictions were placed on the time of day or food and water intake when taking the study intervention; however, participants were encouraged to take the study intervention at the same time each day. All participants, regardless of treatment assignment, received lifestyle counseling consistent with current guidelines for weight management of a healthy diet and physical activity.

### Outcomes

For both cohorts, the primary endpoint was the percent maintenance of body weight reduction in SURMOUNT-5 for participants who achieved a body weight plateau. For each participant, this endpoint is defined as the ratio between the total weight loss achieved between the start of SURMOUNT-5 and the end of ATTAIN-MAINTAIN (week 52) over the total weight loss achieved between the start of SURMOUNT-5 and the baseline of ATTAIN-MAINTAIN (week 0). The key secondary endpoint in plateau participants included the assessment (yes/no) of maintaining at least 80% of body weight reduction. For all participants, the key secondary endpoints included the percent change in body weight from SURMOUNT-5 baseline and percent maintenance of body weight reduction. Additional secondary endpoints include evaluation of maintenance of at least 15% body weight reduction from SURMOUNT-5 baseline for participants who had already lost at least 15% body weight reduction after 72 weeks of tirzepatide or semaglutide treatment, change (in kg) and percent change in body weight from baseline to week 52, and change in waist circumference (in cm) from baseline to week 52. Safety endpoints include the number and incidence of SAEs, treatment-emergent AEs and study intervention discontinuation owing to AEs. Exploratory endpoints included the use of rescue orforglipron for weight regain of 50% or greater of the body weight reduction achieved during SURMOUNT-5, changes in patient-reported outcomes and changes in cardiometabolic parameters. A detailed discussion of retention of improvements in cardiometabolic parameters will be presented in future publications.

### Statistical analysis

#### Sample size

We estimated that a sample size of 150 participants was required for each cohort to ensure that 118 participants reached a plateau in body weight. This sample size provides approximately 90% power to detect a 10% treatment difference for the primary endpoint. This calculation assumed a 20% discontinuation rate and a common standard deviation of 14%, utilizing a two-group *t*-test with a 5% two-sided significance level.

#### Statistical methods

The primary hypothesis was that orforglipron 36 mg or MTD was superior to placebo at week 52 for mean percent maintenance of body weight reduction in participants who had reached a body weight plateau in SURMOUNT-5.

Primary and key secondary efficacy endpoints were assessed using either a modified intent-to-treat population who received at least one dose of orforglipron or placebo, excluding participants who were inadvertently enrolled, or a subset of participants who achieved body weight plateau. Two estimands were used in this study: the modified treatment-regimen estimand, considered primary, and the efficacy estimand, considered supportive. All results in this Article are based on the modified treatment-regimen estimand, unless otherwise specified. The modified treatment-regimen estimand evaluated the treatment effect regardless of treatment discontinuation or initiation of prohibited medications. This estimand also assumes that participants who had bariatric surgery or another weight loss procedure or took rescue orforglipron would not have received any additional improvement from their randomized study treatment. For the modified treatment-regimen estimand, the analysis of covariance model was used to analyze continuous measurements at week 52. This analysis adjusted for baseline value, stratification factors (plateau at week 72 in SURMOUNT-5, sex and percent weight loss at week 72 of SURMOUNT-5 (<20%, ≥20%)) and interactions of treatment-by-baseline and treatment-by-stratification factors, incorporating imputed data for missing values at baseline and missing endpoints at week 52. Binary endpoints, such as reaching at least 80% maintenance of body weight reduction at week 52, were analyzed using a logistic regression model, with treatment, stratification factors and continuous baseline value, and interactions of treatment-by-baseline and treatment-by-stratification factors as covariates. The efficacy estimand evaluated the treatment effect assuming that participants had stayed on treatment, had not taken prohibited medications, had not had bariatric surgery or any other weight management procedures and assuming that participants who took rescue orforglipron would not have received any additional improvement from their randomized study treatment. For the efficacy estimand and for continuous endpoints, a maximum-likelihood-based mixed model for repeated measures (MMRM) was used with adjustment for baseline value and stratification factors, considering three-way interactions between treatments, visits and baseline value (or stratification factors). Additional details on statistical methodology can be found in the statistical analysis plan in the Supplementary Information. For the primary and key secondary end points, the overall type I error rate was controlled at a two-sided alpha level of 0.05 within each estimand by means of a graphical testing procedure for each cohort separately.

Safety endpoints were evaluated using the Safety Analysis Set before rescue (SAS-Before Rescue), which included data from all participants who were randomly assigned to the study and had received at least one dose of the study intervention. Data obtained after rescue were excluded. In addition, selected safety endpoints will be evaluated using the Safety Analysis Set Orforglipron (SAS-OFG), which included data after the first dose of orforglipron for participants randomized to orforglipron and data after initiation of rescue orforglipron for participants randomized to placebo. Safety assessments included AEs and SAEs reported throughout the study. The frequency and proportion of participants experiencing AEs and SAEs were summarized descriptively. Categorical comparisons were made using Fisher’s exact test, and risk differences with 95% CIs were provided.

Statistical analyses were computed using R version 4.4.2. Additional statistical methods are available in the Supplementary Information (statistical analysis plan).

### Reporting summary

Further information on research design is available in the Nature Portfolio Reporting Summary linked to this article.

## Online content

Any methods, additional references, Nature Portfolio reporting summaries, source data, extended data, supplementary information, acknowledgements, peer review information; details of author contributions and competing interests; and statements of data and code availability are available at 10.1038/s41591-026-04386-7.

## Supplementary information

Supplementary Information Supplementary Table 1 (list of investigators), Study Protocol 1 and Statistical Analysis Plan 1.

Reporting Summary

## Source data

Source Data Fig. 2 Sensitivity analysis.

Source Data Fig. 3 Sensitivity analysis.

Source Data Extended Data Fig./Table 1 MMRM.

Source Data Extended Data Fig./Table 2 Sensitivity analysis.

Source Data Extended Data Fig./Table 3 Sensitivity analysis.

Source Data Extended Data Fig./Table 4 Sensitivity analysis.

Source Data Extended Data Fig./Table 5 Sensitivity analysis.

## Figures and Tables

**Extended Data Table 1:**
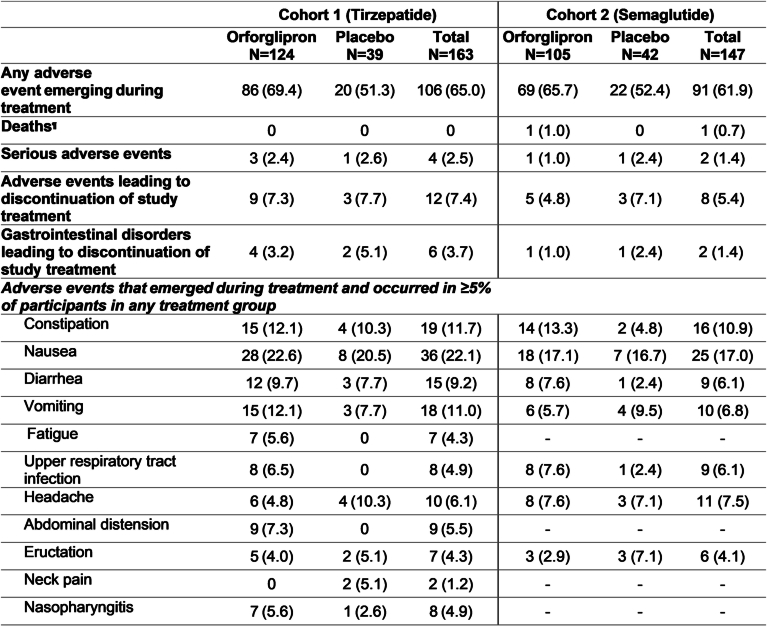
Adverse events (safety analysis set)

Data are number of participants (%).

¶ Cause of death was reported as follows: cardiovascular/stroke; Deaths were also counted as serious adverse events and discontinuation due to adverse events.

**Extended Data Table 2:**
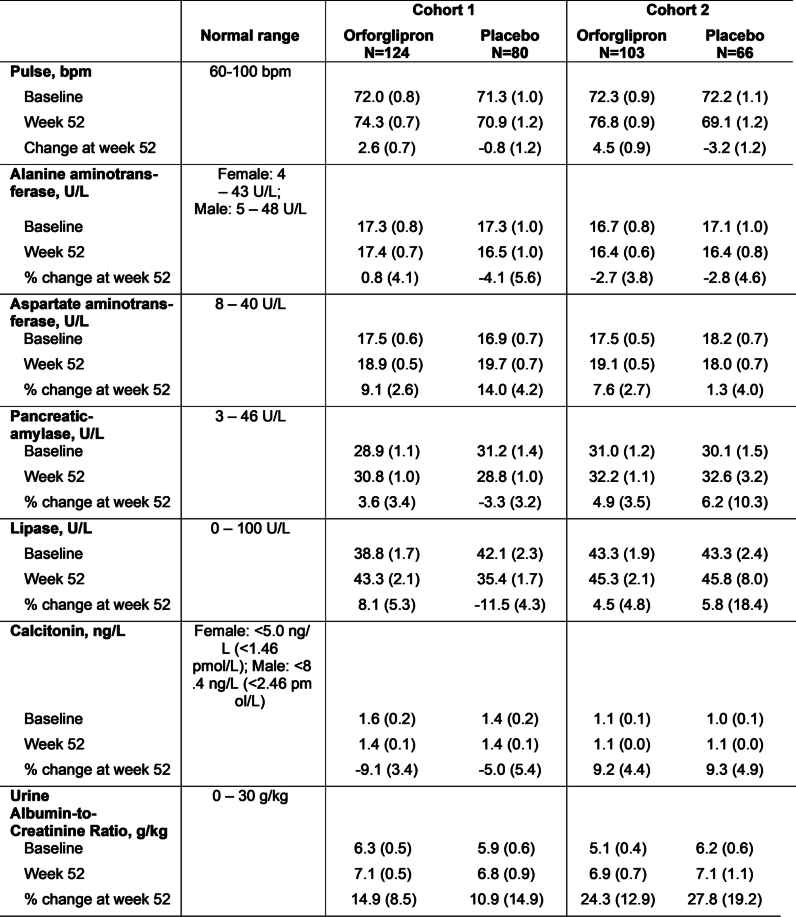
Additional safety measures

Data are MBE (SE). Abbreviations include MBE = Model-based estimate

**Extended Data Table 3:**
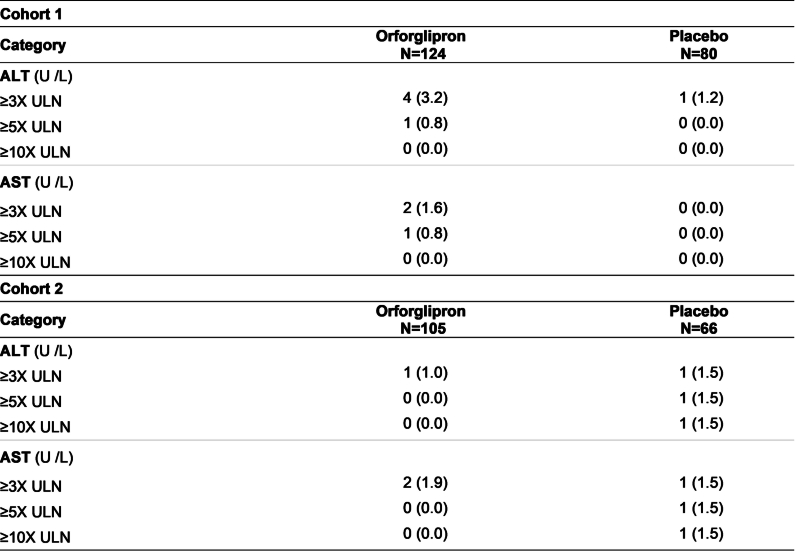
Summary of participants with maximum postbaseline ALT or AST ≥ 3X, ≥ 5X, and ≥ 10X upper limit of normal

Data presented as number of participants, n (%). N = number of participants with at least one post-baseline measurement for the specified parameters including results obtained from both central and local labs.

## Data Availability

Lilly provides access to all individual participant data collected during the trial, after anonymization, with the exception of pharmacokinetic or genetic data. Data are available to request 6 months after the indication studied has been approved in the USA and European Union and after primary publication acceptance, whichever is later. No expiration date of data requests is currently set once data are made available. Access is provided after a proposal has been approved by an independent review committee identified for this purpose and after receipt of a signed data sharing agreement. Data and documents, including the study protocol, statistical analysis plan, clinical study report, blank or annotated case report forms, will be provided in a secure data sharing environment. For details on submitting a request, see the instructions provided at www.vivli.org. Source data are provided with this paper.
