# Supplementary material for: Orforglipron for maintenance of body weight reduction: the double-blind, randomized phase 3b ATTAIN-MAINTAIN trial
Source: Nat Med. 2026 May 13;32(7):2679–87. doi: 10.1038/s41591-026-04386-7 (PMC13375559; doi:10.1038/s41591-026-04386-7)
Supplement: Supplementary file 1 — Supplementary Table 1 (list of investigators), Study Protocol 1 and Statistical Analysis Plan 1. [file 41591_2026_4386_MOESM1_ESM.pdf]

nature medicine

Supplementary information https://doi.org/10.1038/s41591-026-04386-7

Orforglipron for maintenance of body weight reduction: the double-blind, randomized phase 3b ATTAIN-MAINTAIN trial

In the format provided by the authors and unedited

CONTENTS

LIST OF INVESTIGATORS
STUDY PROTOCOL
STATISTICAL ANALYSIS PLAN

LIST OF INVESTIGATORS

Name	Institution
**United States of America**	
Donald Franklin Gardner	Biopharma Informatic, LLC
Shannon Bauman	Iowa Diabetes and Endocrinology Research Center
Timothy Smith	StudyMetrix Research-Research
Lisa Connery	Alliance for Multispecialty Research, LLC
Deborah Horn	The University of Texas Health Science Center at Houston-General Surgery MIST Division
Harold Bays	L-MARC Research Center
Alpana Shukla	Weill Cornell Medical College-Comprehensive Weight Control Center
Lazaro Nunez	New Horizon Research Center
Sumana Gangi	Southern Endocrinology Associates-Southern Endocrinology Associates PA
Kevin Prier	Rocky Mountain Clinical Research, LLC
Hanid Audish	Encompass Clinical Research
Demetri Marshall	Prime Health and Wellness/SKYCRNG
Jordan Vaughn	Cahaba Research, Inc.
Peter Bressler	North Texas Endocrine Center-Research Department
Betsy Palal	Palm Research Center Sunset-Research
Samer Nakhle	Palm Research Center Tenaya-Research
Obadias Marquez	WR-Clinsearch, LLC
Michael Vaughn	Cahaba Research-Pelham
Wayne Ho	Southern California Clinical Research
Joanna Van	University Clinical Investigators, Inc. - Diabetes Research Center
Alexander Murray	PharmQuest Life Sciences, LLC
Neil Fraser	Arcturus Healthcare, PLC, Troy Internal Medicine Research Division
Ronald Mayfield	Tribe Clinical Research, LLC
Gustavo Albizu Angulo	Wellness clinical Research Vega Baja
Prashant Koshy	PlanIt Research, PLLC
Michael Lillestol	Lillestol Research, LLC

Julio Rosenstock	Velocity Clinical Research, Dallas
Dwight Blake	Rophe Adult and Pediatric Medicine/SKYCRNG
Jose Santiago	Puerto Rico Medical Research

Title Page

Confidential Information

The information contained in this document is confidential and is intended for the use of clinical investigators. It is the property of Eli Lilly and Company or its subsidiaries and should not be copied by or distributed to persons not involved in the clinical investigation of orforglipron (LY3502970), unless such persons are bound by a confidentiality agreement with Eli Lilly and Company or its subsidiaries.

Note to Regulatory Authorities: This document may contain protected personal data and/or commercially confidential information exempt from public disclosure. Eli Lilly and Company requests consultation regarding release/redaction prior to any public release. In the United States, this document is subject to Freedom of Information Act (FOIA) Exemption 4 and may not be reproduced or otherwise disseminated without the written approval of Eli Lilly and Company or its subsidiaries.

Protocol Title:

A Phase 3b, Randomized, Double-Blind, Placebo-Controlled Study to Evaluate the Efficacy and Safety of Orforglipron Once Daily Versus Placebo for Maintenance of Body Weight Reduction in Participants Who Have Obesity or Overweight with Weight-Related Comorbidities (ATTAIN- MAINTAIN)

Protocol Number: J2A-MC-GZPN

Amendment Number: a

Compound: Orforglipron (LY3502970)

Brief Title:

Efficacy and Safety of Orforglipron Compared with Placebo for Maintenance of Body Weight Reduction in Participants with Obesity or Overweight with Weight-Related Comorbidities (ATTAIN-MAINTAIN)

Study Phase: 3b

Acronym: ATTAIN-MAINTAIN

Sponsor Name: Eli Lilly and Company

Legal Registered Address: Indianapolis, Indiana, USA 46285

Regulatory Agency Identifier Number:

IND: 156143

Approval Date: Protocol Amendment (a) Electronically Signed and Approved by Lilly on date provided below.

Document ID: VV-CLIN-151791

Medical monitor name and contact information will be provided separately.

Protocol Amendment Summary of Changes Table

DOCUMENT HISTORY	
**Document**	**Date**
Original Protocol	27-May-2024

Amendment (a)

Overall rationale for the amendment

The overall rationale for the changes implemented in the amendment is to

- add 2 key secondary endpoints for participants treated with tirzepatide and for those treated with semaglutide in SURMOUNT-5 study to evaluate percent maintenance of BW reduction achieved during the 72 weeks of tirzepatide or semaglutide treatment in all participants and assessment (yes/no) of maintaining ≥80% of the BW reduction achieved during 72 weeks of tirzepatide or semaglutide treatment in participants who have reached a BW plateau. This is to strengthen the clinical evidence of efficacy and maximize the scientific value of the study.
- update primary estimands section to indicate that 2 estimands are planned for the study. The modified treatment-regimen estimand will be considered the primary estimand and the efficacy estimand will be considered supportive. Removed the reference to secondary estimand previously intended to be evaluated with the modified treatment-regimen estimand.
- remove the 0% and 100% capping from primary endpoint calculation.

Additional changes are included in the table. Minor editorial or formatting changes are not included in this table.

Approved on 10 Nov 2025 GMT

Section # and Name	Description of Change	Brief Rationale
Section 1.1. Synopsis and Section 3.1. Participants Treated with Tirzepatide in SURMOUNT-5 Study<br><br>Section 1.1. Synopsis and Section 3.2. Participants Treated with Semaglutide in SURMOUNT-5 Study<br><br>Section 8.1.2. Secondary Efficacy Assessments	Added 2 key secondary objectives and corresponding endpoints for participants treated with tirzepatide and for those treated with semaglutide in SURMOUNT-5 study:<br>• to demonstrate that orforglipron 36 mg or MTD (24 mg or 36 mg) is superior to placebo at Week 52 for the mean percent maintenance of BW reduction achieved with 72 weeks of treatment with tirzepatide or semaglutide in all participants. Endpoint will evaluate percent maintenance of BW reduction achieved during the 72 weeks of tirzepatide or semaglutide treatment.<br>• to demonstrate that orforglipron 36 mg or MTD (24 mg or 36 mg) is superior to placebo at Week 52 for maintenance of BW reduction achieved with 72 weeks treatment of tirzepatide or semaglutide in participants who have reached a BW plateau. Endpoint will evaluate assessment (yes/no) of maintaining ≥80% of the BW reduction achieved during 72 weeks of tirzepatide or semaglutide treatment.	To strengthen the clinical evidence of efficacy and maximize the scientific value of the study
Section 1.1. Synopsis and Section 3.3. Primary Estimand	• Added a new heading to specify ‘Primary Estimands’.<br>• Primary estimand is updated to indicate that 2 estimands are planned for the study: the modified treatment-regimen estimand, considered as primary and the efficacy estimand, considered as supportive.<br>• The order has been switched to present modified treatment-regimen estimand followed by efficacy estimand.<br>• Removed the reference to secondary estimand previously intended to be evaluated with the modified treatment-regimen estimand.	To address regulatory feedback and consistent guidance to indicate that the modified treatment-regimen estimand should serve as the primary estimand.

Section # and Name	Description of Change	Brief Rationale
Section 1.3. Schedule of Activities (SoA)	The timepoints for the following laboratory tests and sample collections assessments have been added:<br>• Cystatin C laboratory draw at Visit 801.<br>• eGFR calculation at Visit 2	Clarification of the timepoints in the SoA<br>• To calculate eGFR at Visit 801<br>• To calculate eGFR at Visit 2 as the appropriate laboratory samples are already being collected (that is, creatinine and cystatin-c)
Section 1.3. Schedule of Activities (SoA)	The timepoints for the following randomization and dosing related activities have been added:<br>• "Register visit with IWRS" at Visits 1, 9, and 13.<br>• "Dispense study intervention via IWRS" at Visits 5, 7, 9, 10, 12 and 13<br>• "Assess Study Intervention Compliance" at Visits 9, and 13.	Clarification of the timepoints in the SoA<br>All visits should be registered in IWRS.<br>To be consistent with "Register visit with IWRS" assessment timepoints.<br>To be consistent with Section 6.5. Study Intervention Compliance that states "participant compliance with study intervention will be assessed at each visit."
Section 3.1. Participants Treated with Tirzepatide in SURMOUNT-5 Study<br>Section 3.2. Participants Treated with Semaglutide in SURMOUNT-5 Study	The additional secondary endpoint on assessment (yes/no) of maintaining ≥80% of the BW reduction has been removed and added as a key secondary endpoint.	To strengthen the clinical evidence of efficacy to maximize the scientific value of the study
Section 9.1. Statistical Hypotheses	The null hypothesis corresponding to the primary objective has been revised to specify participants who have reached a BW plateau, and the null hypotheses corresponding to the key secondary objectives have been updated to reflect the inclusion of 2 new key secondary endpoints.	To strengthen the clinical evidence of efficacy and maximize the scientific value of the study
Section 9.1.1 Multiplicity Adjustment	Revised multiplicity adjusted analyses to control the overall family-wise error rate and a graphical testing scheme will be used for the key secondary endpoints.	To clarify that each treatment group will have its own alpha level (0.05).

Section # and Name	Description of Change	Brief Rationale
Section 9.3.2. Primary Endpoint(s)/Estimand(s) Analysis	Removed the 0% and 100% capping previously included in the calculation of percent maintenance of BW reduction from SURMOUNT-5 study	To preserve the distribution of the primary endpoint as it is a continuous endpoint.
9.3.3. Secondary Endpoints Analysis	Added the analysis for the 2 new key secondary endpoints in alignment with Section 3.	To align the analysis methods with the 2 new key secondary endpoints

Table of Contents

Protocol Amendment Summary of Changes Table 3

Table of Contents 7

1. Protocol Summary 11
1.1. Synopsis 11
1.2. Schema 17
1.3. Schedule of Activities (SoA) 18

2. Introduction 32
2.1. Study Rationale 32
2.2. Background 32
2.3. Benefit/Risk Assessment 34
2.3.1. Risk Assessment 34
2.3.2. Benefit Assessment 34
2.3.3. Overall Benefit Risk Conclusion 34

3. Objectives, Endpoints, and Estimands 36
3.1. Participants Treated with Tirzepatide in SURMOUNT-5 Study 36
3.2. Participants Treated with Semaglutide in SURMOUNT-5 Study 39
3.3. Estimands 41

4. Study Design 44
4.1. Overall Design 44
4.2. Scientific Rationale for Study Design 44
4.3. Justification for Dose 45
4.4. End of Study Definition 46

5. Study Population 47
5.1. Inclusion Criteria 47
5.2. Exclusion Criteria 47
5.3. Lifestyle Considerations 51
5.3.1. Meals and Dietary Restrictions 52
5.3.2. Monitoring Nutritional Needs 53
5.3.3. Healthy Physical Activity 53
5.3.4. Activity Before Blood Collections 53
5.3.5. Blood Donation 53
5.3.6. Diabetes Education 53
5.4. Screen Failures 53
5.5. Criteria for Temporarily Delaying Enrollment of a Participant 54

6. Study Intervention(s) and Concomitant Therapy 55
6.1. Study Intervention(s) Administered 55
6.2. Preparation, Handling, Storage, and Accountability 56
6.3. Assignment to Study Intervention 56
6.4. Blinding 56
6.5. Study Intervention Compliance 57
6.6. Dose Modification 57
6.6.1. Management of Gastrointestinal Symptoms 58

6.7. Continued Access to Study Intervention after the End of the Study 59
6.8. Treatment of Overdose 60
6.9. Prior and Concomitant Therapy 60
6.9.1. Symptomatic Medication for Gastrointestinal Symptoms 61
6.9.2. Initiation of Antihyperglycemic Medications 61
6.9.3. Prohibited or Restricted Use Medications 61
6.9.4. Prohibited Surgical Treatments or Procedures for Weight Management 62
6.9.5. Rescue Medicine 62

7. Discontinuation of Study Intervention and Participant Discontinuation/Withdrawal 64
7.1. Discontinuation of Study Intervention 64
7.1.1. Hepatic Criteria for Study Intervention Interruption or Discontinuation 65
7.1.2. Temporary Discontinuation 65
7.2. Participant Discontinuation/Withdrawal from the Study 66
7.3. Lost to Follow-up 67

8. Study Assessments and Procedures 68
8.1. Efficacy Assessments 68
8.1.1. Primary Efficacy Assessments 68
8.1.2. Secondary Efficacy Assessments 68
8.1.3. Exploratory Efficacy Assessments 69
8.1.4. Patient-Reported Outcomes 69
8.2. Safety Assessments 71
8.2.1. Physical Examinations 71
8.2.2. Vital Signs 71
8.2.3. Electrocardiograms 72
8.2.4. Clinical Safety Laboratory Tests 72
8.2.5. Pregnancy Testing 73
8.2.6. Hepatic Safety Monitoring 73
8.2.7. Hypersensitivity Reactions 76
8.2.8. Suicidal Ideation and Behavior Risk Monitoring 77
8.2.9. Depression Monitoring 77
8.3. Adverse Events, Serious Adverse Events, and Product Complaints 78
8.3.1. Timing and Mechanism for Collecting Events 78
8.3.2. Collection of Pregnancy Information 80
8.3.3. Adverse Events of Special Interest and Other Safety Topics 81
8.4. Pharmacokinetics 87
8.5. Pharmacodynamics 87
8.6. Genetics 87
8.7. Biomarkers 87
8.8. Immunogenicity Assessments 87
8.9. Medical Resource Utilization and Health Economics 87

9. Statistical Considerations 88
9.1. Statistical Hypotheses 88
9.1.1. Multiplicity Adjustment 88
9.2. Analyses Sets 89
9.3. Statistical Analyses 90
9.3.1. General Considerations 90
9.3.2. Primary Endpoint/Estimands Analysis 91
9.3.3. Secondary Endpoints/Estimands Analysis 92
9.3.4. Exploratory Endpoint Analysis 93
9.3.5. Safety Analyses 93
9.3.6. Other Analyses 94
9.4. Interim Analysis 95
9.5. Sample Size Determination 95

10. Supporting Documentation and Operational Considerations 96
10.1. Appendix 1: Regulatory, Ethical, and Study Oversight Considerations 96
10.1.1. Regulatory and Ethical Considerations 96
10.1.2. Financial Disclosure 97
10.1.3. Informed Consent Process 97
10.1.4. Data Protection 97
10.1.5. Committees Structure 98
10.1.6. Dissemination of Clinical Study Data 98
10.1.7. Data Quality Assurance 99
10.1.8. Source Documents 100
10.1.9. Study and Site Start and Closure 101
10.1.10. Publication Policy 101
10.1.11. Investigator Information 101
10.1.12. Sample Retention 102
10.2. Appendix 2: Clinical Laboratory Tests 103
10.2.1. Laboratory Samples to be Obtained at the Time of a Systemic Hypersensitivity Event 106
10.3. Appendix 3: Adverse Events and Serious Adverse Events: Definitions and Procedures for Recording, Evaluating, Follow-up, and Reporting 107
10.3.1. Definition of AE 107
10.3.2. Definition of SAE 108
10.3.3. Definition of Product Complaints 109
10.3.4. Recording and Follow-Up of AE and/or SAE and Product Complaints 109
10.3.5. Reporting of SAEs 111
10.3.6. Regulatory Reporting Requirements 111
10.4. Appendix 4: Contraceptive and Barrier Guidance 113
10.4.1. Definitions 113
10.4.2. Contraception Guidance 114
10.5. Appendix 5: Liver Safety: Suggested Actions and Follow-up Assessments 117

10.6. Appendix 6: Measurement of Height, Weight, Waist, and Hip Circumference 119
10.7. Appendix 7: Prohibited Medications or Medications with Special Use Restrictions 121
10.7.1. Excluded/Prohibited or Restricted Use Medications 121
10.7.2. Medications with Special Use Restrictions....124
10.8. Appendix 8: Definition and Management of Diabetes 126
10.9. Appendix 9: Provisions for Changes in Study Conduct During Exceptional Circumstances....128
10.10. Appendix 10: Abbreviations and Definitions 132

11. References....137

1. Protocol Summary

1.1. Synopsis

Protocol Title:

A Phase 3b, Randomized, Double-Blind, Placebo-Controlled Study to Evaluate the Efficacy and Safety of Orforglipron Once Daily Versus Placebo for Maintenance of Body Weight Reduction in Participants Who Have Obesity or Overweight with Weight-Related Comorbidities (ATTAIN-MAINTAIN)

Brief Title:

Efficacy and Safety of Orforglipron Compared with Placebo for Maintenance of Body Weight Reduction in Participants with Obesity or Overweight with Weight-Related Comorbidities (ATTAIN-MAINTAIN)

Regulatory Agency Identifier Number:

IND: 156143

Rationale:

The persistence of pharmacologic therapies poses a significant challenge across all chronic diseases. In the context of obesity care, discontinuation of anti-obesity medication (AOM) is pervasive, particularly when patients encounter a weight plateau after an initial reduction. Early discontinuation of AOM has been associated with weight regain. Obesity management involves diverse patient factors, including variable obesity phenotypes and biopsychosocial, which may impact response and adherence to pharmacotherapy. Given this complexity, it is essential to explore different strategies for sustaining weight reduction long term. One potential strategy involves transitioning from an injectable AOM to an oral AOM for maintenance of weight reduction.

Study J2A-MC-GZPN (GZPN) aims to investigate whether switching from once-weekly injectable tirzepatide or semaglutide (maximum tolerated dose) to daily oral orforglipron 36 mg or maximum tolerated dose (24 mg or 36 mg) in participants with obesity or overweight with comorbidities (without T2D) can lead to superior long-term maintenance of body weight reduction compared to switching to placebo. Participants must have achieved clinically meaningful body weight reduction (≥5%) to be eligible for this study.

Objectives, Endpoints, and Estimands:

Participants treated with tirzepatide in SURMOUNT-5 Study

Objectives	Endpoints
**Primary**	
To demonstrate that orforglipron 36 mg or MTD (24 mg or 36 mg) is superior to placebo at Week 52 for the mean percent maintenance of BW reduction achieved with 72 weeks of treatment with tirzepatide 15 mg or MTD (10 mg or 15 mg) in participants who have reached a BW plateau[a]	Percent maintenance of BW reduction achieved during the 72 weeks of tirzepatide treatment
**Key Secondary**	
To demonstrate that orforglipron 36 mg or MTD (24 mg or 36 mg) is superior to placebo at Week 52 for the mean percent change in BW from SURMOUNT-5 baseline in all participants	Percent change in BW from SURMOUNT-5 baseline prior to the initiation of tirzepatide treatment
To demonstrate that orforglipron 36 mg or MTD (24 mg or 36 mg) is superior to placebo at Week 52 for the mean percent maintenance of BW reduction achieved with 72 weeks of treatment with tirzepatide 15 mg or MTD (10 mg or 15 mg) in all participants	Percent maintenance of BW reduction achieved during the 72 weeks of tirzepatide treatment
To demonstrate that orforglipron 36 mg or MTD (24 mg or 36 mg) is superior to placebo at Week 52 for maintenance of BW reduction achieved with 72 weeks treatment of tirzepatide 15 mg or MTD (10 mg or 15 mg) in participants who have reached a BW plateau[a]	Assessment (yes/no) of maintaining ≥80% of the BW reduction achieved during 72 weeks of tirzepatide treatment

Abbreviations: BW = body weight; MTD = maximum tolerated dose; SURMOUNT-5 = Study I8F-MC-GPHJ (GPHJ).

a BW plateau is defined as <5% BW change between Visit 17 (Week 60) and Visit 20 (Week 72) of SURMOUNT-5 study.

Participants treated with semaglutide in SURMOUNT-5 Study

Objectives	Endpoints
**Primary**	
To demonstrate that orforglipron 36 mg or MTD (24 mg or 36 mg) is superior to placebo at Week 52 for the mean percent maintenance of BW reduction achieved with 72 weeks of treatment with semaglutide 2.4 mg or MTD (1.7 mg or 2.4 mg) in participants who have reached a BW plateau[a]	Percent maintenance of BW reduction achieved during the 72 weeks of semaglutide treatment

Objectives	Endpoints
**Key Secondary**	
To demonstrate that orforglipron 36 mg or MTD (24 mg or 36 mg) is superior to placebo at Week 52 for the mean percent change in BW from SURMOUNT-5 baseline in all participants	Percent change in BW from SURMOUNT-5 baseline prior to the initiation of semaglutide treatment
To demonstrate that orforglipron 36 mg or MTD (24 mg or 36 mg) is superior to placebo at Week 52 for the mean percent maintenance of BW reduction achieved with 72 weeks of treatment with semaglutide 2.4 mg or MTD (1.7 mg or 2.4 mg) in all participants	Percent maintenance of BW reduction achieved during the 72 weeks of semaglutide treatment
To demonstrate that orforglipron 36 mg or MTD (24 mg or 36 mg) is superior to placebo at Week 52 for maintenance of BW reduction achieved with 72 weeks of treatment with semaglutide 2.4 mg or MTD (1.7 mg or 2.4 mg) in participants who have reached a BW plateau[a]	Assessment (yes/no) of maintaining ≥80% of the BW reduction achieved during 72 weeks of semaglutide treatment

Abbreviations: BW = body weight; MTD = maximum tolerated dose; SURMOUNT-5 = Study I8F-MC-GPHJ (GPHJ).

[a] BW plateau is defined as <5% BW change between Visit 17 (Week 60) and Visit 20 (Week 72) of SURMOUNT-5 study.

Estimands

There will be 2 estimands planned in the study, modified treatment-regimen estimand and efficacy estimand. Unless otherwise specified or requested by a regulatory agency, modified treatment-regimen estimand will be the primary estimand, with the efficacy estimand considered supportive. Both the modified treatment-regimen and efficacy estimands will be evaluated for the primary and the key secondary objectives.

Modified treatment-regimen estimand

For each treatment in the SURMOUNT-5 study (tirzepatide or semaglutide), the clinical question of interest is:

What is the treatment difference at Week 52 for orforglipron versus placebo in mean percent maintenance of BW reduction achieved during 72 weeks of treatment with tirzepatide or semaglutide, respectively, as an adjunct to a reduced-calorie diet and increased physical activity in participants with obesity or overweight with at least 1 weight-related comorbid condition, regardless of treatment discontinuation for any reasons and regardless of initiation of other AOMs, GLP-1 RAs, GIP/GLP-1 RAs, or DPP-4 inhibitors. This estimand also assumes that participants who had bariatric surgery or another weight-loss procedure or took rescue orforglipron would not have received any additional improvement from their randomized study treatment?

Rationale for the modified treatment-regimen estimand

This estimand aims at reflecting how participants with obesity or overweight with at least 1 weight-related comorbid condition are treated in clinical practice and takes into account both tolerability and efficacy.

Efficacy estimand

For each treatment in the SURMOUNT-5 study: (tirzepatide or semaglutide), the clinical question of interest is:

What is the treatment difference at Week 52 for orforglipron versus placebo in mean percent maintenance of body weight (BW) reduction achieved during 72 weeks of treatment with tirzepatide or semaglutide, respectively, as an adjunct to a reduced-calorie diet and increased physical activity in participants with obesity or overweight with at least 1 weight-related comorbid condition, assuming that participants had stayed on treatment, had not taken other AOMs, GLP-1 RAs, GIP/GLP-1 RAs, or DPP-4 inhibitors, had not had bariatric surgery or other weight management procedures, and assuming that participants who took rescue orforglipron would not have received any additional improvement from their randomized study treatment?

Rationale for the efficacy estimand

This estimand focuses on the treatment effect if participants who underwent randomization continued to receive the study treatment without taking other AOMs, GLP-1 RAs, GIP/GLP-1 RA, or DPP-4 inhibitors, bariatric surgery or weight-loss procedures, or rescue orforglipron.

Overall Design:

Study GZPN is a Phase 3b, multicenter, randomized, double-blind, placebo-controlled study.

Study participants will be randomly assigned in a 3:2 ratio (orforglipron 36 mg or MTD [24 mg or 36 mg] and placebo).

This study will consist of 3 periods:

- screening and lead-in period: up to 2 weeks
- treatment period: 52 weeks, including dose escalation and maintenance dose, and
- safety follow-up period: 2 weeks.

Brief Summary:

The purpose of this study is to measure efficacy and safety of orforglipron compared with placebo, in achieving maintenance of body weight reduction from the 72 weeks of tirzepatide or semaglutide treatment in participants who have obesity or overweight with weight-related coexisting conditions and previously participated in SURMOUNT-5 study.

Study details include:

- The study duration will be up to 54 weeks.
- The treatment duration will be up to 52 weeks.
- The visit frequency will be every 4 weeks.

Study Population:

In general, an individual may take part in this study if they

- completed SURMOUNT-5 study on study treatment, and completed Visits 2, 17, and 20 (Weeks 0, 60, 72 respectively) of SURMOUNT-5 study.
- have lost ≥5% of BW during 72-week treatment with tirzepatide or semaglutide.
- are reliable and willing to make themselves available and follow study procedures for the duration of the study.
- are assigned male at birth/assigned female at birth, and their use of contraception should be in accordance with the local regulations for participation in clinical trials.
- are capable of giving signed informed consent.

Number of Participants:

For each treatment group in the SURMOUNT-5 study, a sample size of 150 participants will be randomly assigned in a 3:2 ratio to receive orforglipron 36 mg or MTD (24 mg or 36 mg), or placebo, respectively.

Intervention Groups and Duration:

This table lists the interventions used in this clinical study.

**Intervention Name**	Orforglipron	Placebo
**Dosage Level(s)**	1 mg, 3 mg, 6 mg, 12 mg, 24 mg, and 36 mg capsules	Capsule of orforglipron placebo to match
**Route of Administration**	Oral QD	Oral QD

Abbreviation: QD = once daily

All participants will initiate treatment with a 12 mg QD dose of orforglipron or matching placebo and increase dose every 4 weeks until the randomized maintenance dose of 36 mg or MTD (24 mg or 36 mg) is reached.

Ethical Considerations of Benefit/Risk:

The safety and efficacy profile seen to date for orforglipron supports the overall benefit/risk for participants in this study. The anticipated risks are those associated with known effects of medications having similar mechanism of action, namely gastrointestinal tolerability, and increased heart rate. These risks can be monitored, are usually mild to moderate in severity, reversible, and readily manageable. To date there are no recognized AEs from orforglipron other than those related to medications of similar mechanism of action.

The potential risks based on the knowledge from the medications of similar mechanism of action are considered to be acceptable in the context of the potential benefits anticipated from treatment with orforglipron in adult participants with obesity or overweight.

Data Monitoring Committee: No

1.2. Schema

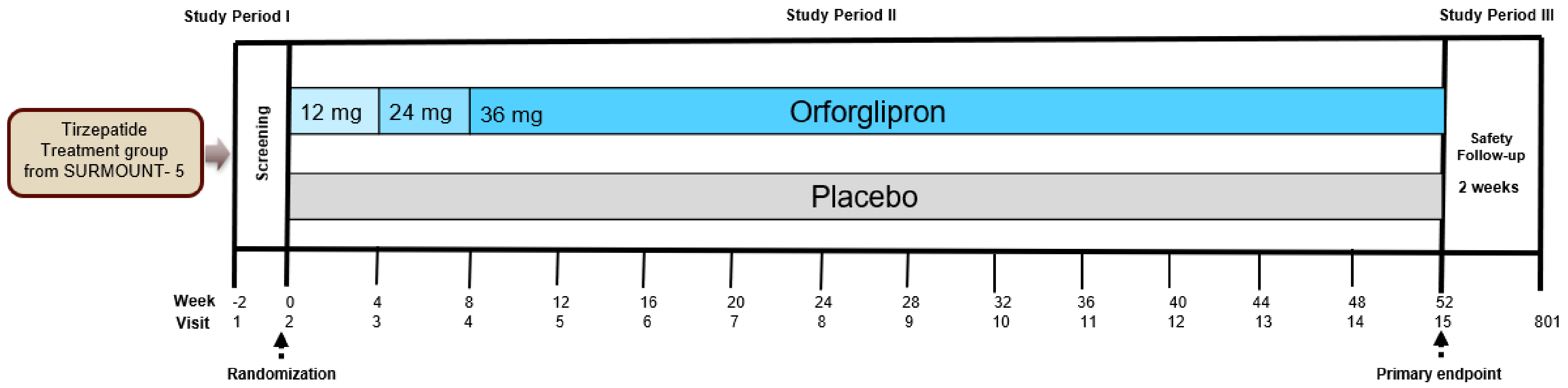

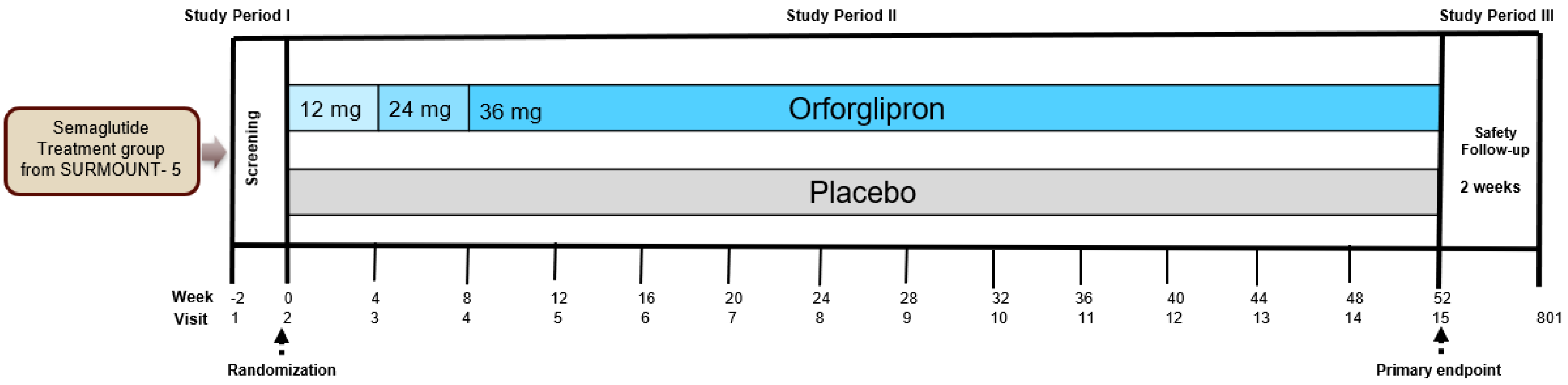

Abbreviation: SURMOUNT-5 = Study I8F-MC-GPHJ (GPHJ).

1.3. Schedule of Activities (SoA)

The SoA should be followed for all participants enrolled in Study J2A-MC-GZPN (GZPN). However, for those participants whose participation in this study is affected by exceptional circumstances, such as pandemics, or natural disasters, please refer to Section 10.9 for additional guidance.

Screening

The screening visit should ideally occur on the same day as the final visit of SURMOUNT-5 study (Study I8F-MC-GPHJ [GPHJ]). All screening activities should take place within the 2-week period. The randomization visit (Visit 2) should occur no later than 14 days after the last dose in SURMOUNT-5 study.

Fasting visits

Study participants should be reminded to report for fasting visits before taking study intervention(s) in a fasting condition, after a period of approximately 8 hours without eating or drinking (except water).

- If a participant attends these visits in a nonfasting state, body weight measurement and samples for laboratory testing should not be collected and the participant should be asked to return to the site in a fasting state as soon as possible; all other procedures scheduled at the visit may be performed.
- All procedures, especially vital signs, laboratory procedures and ECGs, should be completed prior to the participant taking study intervention on the days of office visits.

Early discontinuation (ED)

Participants who are unable or unwilling to continue the study treatment period for any reason will perform an ED of treatment visit. Procedures should be completed according to the SoA. PRO questionnaires should be administered as early as possible in the visit. Administration of Mental Health Questionnaires should be after the assessment for AEs. If the participant is discontinuing during an unscheduled visit or a scheduled visit, that visit should be performed as the ED visit.

Safety follow-up

All participants are required to complete a safety follow-up visit (Visit 801) according to the SoA. Participants discontinuing the study early and performing an ED visit will also be asked to perform the safety follow-up visit.

Telehealth visits

Telehealth visits may be by telephone or other technology. In the event a visit designated as telehealth in the SoA is preferred to be conducted as an office visit (for example, to modify study intervention dose level, AE follow-up), an exception may be granted after consultation with the sponsor-designated medical monitor.

	**Period I - Screening**	**Period II – Double-Blinded Weight Maintenance Period**															**Period III - Safety Follow-up**	**Comments**
**Visit number**	1	2	3	4	5	6	7	8	9	10	11	12	13	14	15	ED	801	
**Weeks from first dose**	-2	0	4	8	12	16	20	24	28	32	36	40	44	48	52			Visit 1 should ideally occur on the same day as the final visit of the SURMOUNT-5study. Visit 2 should occur no later than 14 days after the last dose in SURMOUNT-5 study.
**Visit interval tolerance (days)**	-14 to -7		±3	±3	±3	±3	±3	±3	±3	±3	±3	±3	±3	±3	±7		±7	
**Fasting visit**	X	X	X	X		X		X			X			X	X	X	X	
**Telehealth visit**					X		X		X	X		X	X					Telehealth visits may be converted to in-clinic visits if clinically indicated.
**Consent and demographics**																		
Informed consent	X																	Must be signed before any protocol-specific tests or procedures are performed. Refer to Section 10.1.3 for additional details.
Inclusion and exclusion criteria, review and confirm	X	X																Confirm prior to randomization and administration of first dose of study intervention.
Demographics	X																	Includes ethnicity (where permissible), year of birth, sex assigned at birth, and race.
Preexisting conditions and medical history, including relevant surgical history	X																	Collect all conditions ongoing and relevant past surgical and medical history.
Prespecified medical history	X																	Should include, but not limited to, collecting diagnosis of obesity and obesity related health problems.
Prior treatments for indication	X																	Include medications used for obesity or overweight.

	Period I - Screening	Period II – Double-Blinded Weight Maintenance Period															Period III - Safety Follow-up	Comments
**Visit number**	1	2	3	4	5	6	7	8	9	10	11	12	13	14	15	ED	801	
**Weeks from first dose**	-2	0	4	8	12	16	20	24	28	32	36	40	44	48	52			Visit 1 should ideally occur on the same day as the final visit of the SURMOUNT-5study. Visit 2 should occur no later than 14 days after the last dose in SURMOUNT-5 study.
**Visit interval tolerance (days)**	-14 to -7		±3	±3	±3	±3	±3	±3	±3	±3	±3	±3	±3	±3	±7		±7	
**Fasting visit**	X	X	X	X		X		X			X			X	X	X	X	
**Telehealth visit**					X		X		X	X		X	X					Telehealth visits may be converted to in-clinic visits if clinically indicated.
Substance use (alcohol, tobacco use)	X																	
Concomitant medications	X	X	X	X	X	X	X	X	X	X	X	X	X	X	X	X	X	Medications that lower pH should be dosed 2 hours from study intervention. Refer to Section 6.9.<br>For prohibited and restricted use medications, refer to Section 6.9.3.
AEs	X	X	X	X	X	X	X	X	X	X	X	X	X	X	X	X	X	Any events that occur after signing the informed consent are considered AEs as defined in Section 10.3. Additional data are collected for certain AEs.<br>Hypoglycemia should be reported as AE, refer to Section 8.3.3.4.
Evaluation of antihypertensive or lipid-lowering treatment								X							X	X		For participants receiving antihypertensive or lipid-lowering treatment, the investigator should evaluate changes in the participant's treatment intensity within each therapeutic area. The evaluation should be based on whether an overall change from randomization until the time of evaluation has occurred.

	Period I - Screening	Period II – Double-Blinded Weight Maintenance Period															Period III - Safety Follow-up	Comments
**Visit number**	1	2	3	4	5	6	7	8	9	10	11	12	13	14	15	ED	801	
**Weeks from first dose**	-2	0	4	8	12	16	20	24	28	32	36	40	44	48	52			Visit 1 should ideally occur on the same day as the final visit of the SURMOUNT-5study. Visit 2 should occur no later than 14 days after the last dose in SURMOUNT-5 study.
**Visit interval tolerance (days)**	-14 to -7		±3	±3	±3	±3	±3	±3	±3	±3	±3	±3	±3	±3	±7		±7	
**Fasting visit**	X	X	X	X		X		X			X			X	X	X	X	
**Telehealth visit**					X		X		X	X		X	X					Telehealth visits may be converted to in-clinic visits if clinically indicated.
**Physical evaluation**																		
Height	X																	Refer to Section 10.6.
Weight	X	X	X	X		X		X			X			X	X	X	X	For Visit 1: If weight is collected in the final visit of the SURMOUNT-5 study, and the visit occurs on the same day as the screening of this study, record that weight in the CRF. Weight must be measured in the fasting state. If the participant is not fasting, the participant should return at a later date within the visit interval tolerance in the fasting state. Refer to Section 10.6.
Waist circumference	X	X	X	X		X		X			X			X	X	X	X	For Visit 1: If waist circumference is collected in the final visit of the SURMOUNT-5 study, and the visit occurs on the same day as the screening of this study, record that measurement in the CRF. Refer to Section 10.6.
Hip circumference	X	X	X	X		X		X			X			X	X	X	X	Refer to Section 10.6.
Physical examination	X																	Excludes pelvic, rectal, and breast examinations unless clinically indicated.

	**Period I - Screening**	**Period II – Double-Blinded Weight Maintenance Period**															**Period III - Safety Follow-up**	**Comments**
**Visit number**	1	2	3	4	5	6	7	8	9	10	11	12	13	14	15	ED	801	
**Weeks from first dose**	-2	0	4	8	12	16	20	24	28	32	36	40	44	48	52			Visit 1 should ideally occur on the same day as the final visit of the SURMOUNT-5study. Visit 2 should occur no later than 14 days after the last dose in SURMOUNT-5 study.
**Visit interval tolerance (days)**	-14 to -7		±3	±3	±3	±3	±3	±3	±3	±3	±3	±3	±3	±3	±7		±7	
**Fasting visit**	X	X	X	X		X		X			X			X	X	X	X	
**Telehealth visit**					X		X		X	X		X	X					Telehealth visits may be converted to in-clinic visits if clinically indicated.
Symptom-directed physical assessment		X	X	X		X		X			X			X	X	X	X	Will be conducted at the discretion of the PI or qualified personnel per local regulations, as indicated based on participant status and standard of care. Qualified personnel per local regulations will perform the examination. Excluding screening visit as it includes a physical examination.
Vital signs (triplicate)	X	X	X	X		X		X			X			X	X	X	X	Measure after participant has been sitting at least 5 minutes and before obtaining an ECG tracing and before collection of blood samples for laboratory testing. Refer to Section 8.2.2. Measure recordings at least 1 minute apart.

	**Period I - Screening**	**Period II – Double-Blinded Weight Maintenance Period**															**Period III - Safety Follow-up**	**Comments**
**Visit number**	1	2	3	4	5	6	7	8	9	10	11	12	13	14	15	ED	801	
**Weeks from first dose**	-2	0	4	8	12	16	20	24	28	32	36	40	44	48	52			Visit 1 should ideally occur on the same day as the final visit of the SURMOUNT-5study. Visit 2 should occur no later than 14 days after the last dose in SURMOUNT-5 study.
**Visit interval tolerance (days)**	-14 to -7		±3	±3	±3	±3	±3	±3	±3	±3	±3	±3	±3	±3	±7		±7	
**Fasting visit**	X	X	X	X		X		X			X			X	X	X	X	
**Telehealth visit**					X		X		X	X		X	X					Telehealth visits may be converted to in-clinic visits if clinically indicated.
12-lead ECG (single, local)		X						X							X	X	X	Perform prior to collection of blood samples for laboratory testing. Participants should be supine for approximately 5 to 10 minutes before ECG collection and remain supine but awake during the ECG collection. ECGs may be repeated at the investigator's discretion at any visit. Refer to Section 8.2.3.
Individual AFAB reproductive status: menopausal status	X																	For Visit 1: If individual AFAB reproductive status: menopausal status is collected in the final visit of the SURMOUNT-5 study, and the visit occurs on the same day as the screening of this protocol, record in the CRF.
First day of last menstrual cycle in IOCBP with menstrual cycles	X	X	X	X	X	X	X	X	X	X	X	X	X	X	X	X		For Visit 1: If first day of the last menstrual cycle in IOCBP with menstrual cycles are collected in the final visit of the SURMOUNT-5 study, and the visit occurs on the same day as the screening of this protocol, record in the CRF.
**Patient-reported outcomes**																		Complete before any clinician-administered assessments.
SF-36 v2, acute form		X						X							X	X		Refer to Section 8.1.4.1.

	Period I - Screening	Period II – Double-Blinded Weight Maintenance Period															Period III - Safety Follow-up	Comments
**Visit number**	1	2	3	4	5	6	7	8	9	10	11	12	13	14	15	ED	801	
**Weeks from first dose**	-2	0	4	8	12	16	20	24	28	32	36	40	44	48	52			Visit 1 should ideally occur on the same day as the final visit of the SURMOUNT-5study. Visit 2 should occur no later than 14 days after the last dose in SURMOUNT-5 study.
**Visit interval tolerance (days)**	-14 to -7		±3	±3	±3	±3	±3	±3	±3	±3	±3	±3	±3	±3	±7		±7	
**Fasting visit**	X	X	X	X		X		X			X			X	X	X	X	
**Telehealth visit**					X		X		X	X		X	X					Telehealth visits may be converted to in-clinic visits if clinically indicated.
CoEQ		X		X				X			X				X	X		Refer to Section 8.1.4.2.
PGIS-Physical Function Weight		X						X							X	X		Refer to Section 8.1.4.3.
PGIS-Food Craving		X		X				X			X				X	X		Refer to Section 8.1.4.4.
PGIC-Physical Function Weight								X							X	X		Refer to Section 8.1.4.5.
PGIC-Food Craving				X				X			X				X	X		Refer to Section 8.1.4.6.
rPDQS		X		X				X			X				X	X		Refer to Section 8.1.4.7.
PHQ-9	X	X	X	X		X		X			X			X	X	X	X	For Visit 1: If PHQ-9 is collected in the final visit of the SURMOUNT-5 study, and the visit occurs on the same day as the screening of this protocol, record in the CRF. Refer to Section 8.2.9.
**Clinician administered questionnaires** *Collect after the assessment of AEs*																		
C-SSRS Screening/Baseline	X																	Authorized study personnel to collect via a paper source document and to transcribe into the EDC system. Refer to Section 8.2.8.

	Period I - Screening	Period II – Double-Blinded Weight Maintenance Period															Period III - Safety Follow-up	Comments
**Visit number**	1	2	3	4	5	6	7	8	9	10	11	12	13	14	15	ED	801	
**Weeks from first dose**	-2	0	4	8	12	16	20	24	28	32	36	40	44	48	52			Visit 1 should ideally occur on the same day as the final visit of the SURMOUNT-5study. Visit 2 should occur no later than 14 days after the last dose in SURMOUNT-5 study.
**Visit interval tolerance (days)**	-14 to -7		±3	±3	±3	±3	±3	±3	±3	±3	±3	±3	±3	±3	±7		±7	
**Fasting visit**	X	X	X	X		X		X			X			X	X	X	X	
**Telehealth visit**					X		X		X	X		X	X					Telehealth visits may be converted to in-clinic visits if clinically indicated.
C-SSRS Since Last Assessed		X	X	X		X		X			X			X	X	X	X	Authorized study personnel to collect via a paper source document and to transcribe into the EDC system. Refer to Section 8.2.8.
**Participant education**																		
Dispense diary		X																Dosing diary dispensed at randomization.
Diary review (dosing)		X	X	X	X	X	X	X	X	X	X	X	X	X	X	X	X	
Diary return																X	X	
Lifestyle (diet and exercise goals) program instruction		X	X	X		X		X			X			X	X	X		
Review diet and exercise goals			X	X	X	X	X	X		X	X	X		X	X	X		
Review of contraceptive measures as applicable		X	X	X		X		X			X			X	X			

	**Period I - Screening**	**Period II – Double-Blinded Weight Maintenance Period**															**Period III - Safety Follow-up**	**Comments**
**Visit number**	1	2	3	4	5	6	7	8	9	10	11	12	13	14	15	ED	801	
**Weeks from first dose**	-2	0	4	8	12	16	20	24	28	32	36	40	44	48	52			Visit 1 should ideally occur on the same day as the final visit of the SURMOUNT-5study. Visit 2 should occur no later than 14 days after the last dose in SURMOUNT-5 study.
**Visit interval tolerance (days)**	-14 to -7		±3	±3	±3	±3	±3	±3	±3	±3	±3	±3	±3	±3	±7		±7	
**Fasting visit**	X	X	X	X		X		X			X			X	X	X	X	
**Telehealth visit**					X		X		X	X		X	X					Telehealth visits may be converted to in-clinic visits if clinically indicated.
**Laboratory tests and sample collections**																		
HbA1c	X			X				X			X			X	X	X	X	For Visit 1: If HbA1c is collected in the final visit of the SURMOUNT-5 study, it does not need to be recollected at the screening for this study.
Hematology	X			X		X					X				X	X	X	For Visit 1: If Hematology is collected in the final visit of the SURMOUNT-5 study, it does not need to be recollected at the screening for this study.
Clinical chemistry	X	X	X	X		X		X			X			X	X	X	X	For Visit 1: If Clinical chemistry is collected in the final visit of the SURMOUNT-5 study, it does not need to be recollected at the screening for this study. If, at Visit 801, hepatic laboratory values are elevated participants with normal baseline: ALT or AST ≥3x ULN; participants with elevated baseline: ≥2x baseline, then repeat 2 weeks later.
HBV screening	X																	Confirmation by HBV DNA will be performed if positive for HBcAb.
HCV screening	X																	Confirmation by HCV RNA will be performed if positive for HCV antibody.

	Period I - Screening	Period II – Double-Blinded Weight Maintenance Period															Period III - Safety Follow-up	Comments
**Visit number**	1	2	3	4	5	6	7	8	9	10	11	12	13	14	15	ED	801	
**Weeks from first dose**	-2	0	4	8	12	16	20	24	28	32	36	40	44	48	52			Visit 1 should ideally occur on the same day as the final visit of the SURMOUNT-5study. Visit 2 should occur no later than 14 days after the last dose in SURMOUNT-5 study.
**Visit interval tolerance (days)**	-14 to -7		±3	±3	±3	±3	±3	±3	±3	±3	±3	±3	±3	±3	±7		±7	
**Fasting visit**	X	X	X	X		X		X			X			X	X	X	X	
**Telehealth visit**					X		X		X	X		X	X					Telehealth visits may be converted to in-clinic visits if clinically indicated.
Lipid panel		X		X				X							X	X	X	
Serum pregnancy	X																	Collect for IOCBP. Refer to Section 10.4.
Urine pregnancy (local)		X		X				X			X			X	X	X		The result must be available before the first dose of study intervention for IOCBP. Perform additional pregnancy tests at any time during the trial if a menstrual period is missed, there is clinical suspicion of pregnancy, or as required by local law or regulation. If the urine pregnancy test is inconclusive at any visit, an additional serum pregnancy test should be collected.
Follicle-stimulating hormone (FSH)	X																	If participant has diagnosis of menopausal status, this test is not needed. Perform as needed to confirm postmenopausal status. Refer to Section 10.4.1.
Insulin		X		X				X			X			X	X	X		
C-peptide		X		X				X			X			X	X	X		
Cystatin C	X	X		X				X			X			X	X	X	X	For Visit 1: If cystatin C is collected in the final visit of the SURMOUNT-5 study, it does not need to be recollected at the screening for this study.

	Period I - Screening	Period II – Double-Blinded Weight Maintenance Period															Period III - Safety Follow-up	Comments
**Visit number**	1	2	3	4	5	6	7	8	9	10	11	12	13	14	15	ED	801	
**Weeks from first dose**	-2	0	4	8	12	16	20	24	28	32	36	40	44	48	52			Visit 1 should ideally occur on the same day as the final visit of the SURMOUNT-5study. Visit 2 should occur no later than 14 days after the last dose in SURMOUNT-5 study.
**Visit interval tolerance (days)**	-14 to -7		±3	±3	±3	±3	±3	±3	±3	±3	±3	±3	±3	±3	±7		±7	
**Fasting visit**	X	X	X	X		X		X			X			X	X	X	X	
**Telehealth visit**					X		X		X	X		X	X					Telehealth visits may be converted to in-clinic visits if clinically indicated.
Calcitonin	X							X							X	X	X	For Visit 1: If calcitonin is collected in the final visit of the SURMOUNT-5 study, it does not need to be recollected at the screening for this study.
Pancreatic amylase	X							X							X	X	X	For Visit 1: If pancreatic amylase is collected in the final visit of the SURMOUNT-5 study, it does not need to be recollected at the screening for this study.
Lipase	X							X							X	X	X	For Visit 1: If lipase is collected in the final visit of the SURMOUNT-5 study, it does not need to be recollected at the screening for this study.
eGFR	X	X		X				X			X			X	X	X	X	For Visit 1, eGFR (calculated using CKD-EPI cystatin C method) will be calculated based on laboratory results collected at the final visit of the SURMOUNT-5 study.
Urine albumin/creatinine ratio		X		X				X			X				X	X	X	
hsCRP		X						X							X	X	X	

	Period I - Screening	Period II – Double-Blinded Weight Maintenance Period															Period III - Safety Follow-up	Comments
**Visit number**	1	2	3	4	5	6	7	8	9	10	11	12	13	14	15	ED	801	
**Weeks from first dose**	-2	0	4	8	12	16	20	24	28	32	36	40	44	48	52			Visit 1 should ideally occur on the same day as the final visit of the SURMOUNT-5study. Visit 2 should occur no later than 14 days after the last dose in SURMOUNT-5 study.
**Visit interval tolerance (days)**	-14 to -7		±3	±3	±3	±3	±3	±3	±3	±3	±3	±3	±3	±3	±7		±7	
**Fasting visit**	X	X	X	X		X		X			X			X	X	X	X	
**Telehealth visit**					X		X		X	X		X	X					Telehealth visits may be converted to in-clinic visits if clinically indicated.
**Stored samples**																		
Exploratory biomarker samples		X		X				X							X	X	X	
**Randomization and dosing related activities**																		
Register visit with IWRS	X	X	X	X	X	X	X	X	X	X	X	X	X	X	X	X	X	
Randomization via IWRS		X																
Dispense study intervention via IWRS		X	X	X	X	X	X	X	X	X	X	X	X	X				
Participant returns unused study intervention			X	X		X		X			X			X	X	X		
Assess study intervention compliance			X	X	X	X	X	X	X	X	X	X	X	X	X	X		

	**Period I - Screening**	**Period II – Double-Blinded Weight Maintenance Period**															**Period III - Safety Follow-up**	**Comments**
**Visit number**	1	2	3	4	5	6	7	8	9	10	11	12	13	14	15	ED	801	
**Weeks from first dose**	-2	0	4	8	12	16	20	24	28	32	36	40	44	48	52			Visit 1 should ideally occur on the same day as the final visit of the SURMOUNT-5study. Visit 2 should occur no later than 14 days after the last dose in SURMOUNT-5 study.
**Visit interval tolerance (days)**	-14 to -7		±3	±3	±3	±3	±3	±3	±3	±3	±3	±3	±3	±3	±7		±7	
**Fasting visit**	X	X	X	X		X		X			X			X	X	X	X	
**Telehealth visit**					X		X		X	X		X	X					Telehealth visits may be converted to in-clinic visits if clinically indicated.
Assess eligibility for rescue orforglipron								X			X			X				Refer to Section 6.9.5.

Abbreviations: AE = adverse event; AFAB = assigned female at birth; ALT = alanine aminotransferase; AST = aspartate aminotransferase; CKD-EPI = Chronic Kidney Disease-Epidemiology; CoEQ = Control of Eating Questionnaire-NRS; CRF = case report form; C-SSRS = Columbia-Suicide Severity Rating Scale; ECG = electrocardiogram; eGFR = estimated glomerular filtration rate; HbA1c = Hemoglobin A1c; HBcAb = Hepatitis B core antibody; HBV = Hepatitis B Virus; HCV = Hepatitis C Virus IOCBP= individual of child bearing potential; IWRS = Interactive Web-Response System; PI = principal investigator; PGIC = Patient Global Impression of Change; PGIS = Patient Global Impression of Severity; PHQ-9 = Patient Health Questionnaire-9; rPDQS = rapid Prime Diet Quality Score; SF-36 v2 = Short Form-36 Version 2 Health Survey acute form; SURMOUNT-5 = Study I8F-MC-GPHJ (GPHJ); ULN = upper limit of normal.

2. Introduction

GLP-1 receptor agonism is an established therapeutic mechanism for glycemic control in T2D, as well as weight management in individuals with obesity or overweight. Unlike injectable or orally available peptide GLP-1 RAs approved by regulatory authorities to date, orforglipron is an oral, non-peptide, small-molecule GLP-1 RA. Orforglipron is being developed for the treatment of T2D and for chronic weight management in individuals with overweight or obesity.

2.1. Study Rationale

The persistence of pharmacologic therapies poses a significant challenge across all chronic diseases. In the context of obesity care, discontinuation of AOM is pervasive, particularly when patients encounter a weight plateau after an initial reduction. Early discontinuation of AOM has been associated with weight regain. Obesity management involves diverse patient factors, including variable obesity phenotypes and biopsychosocial, which may impact response and adherence to pharmacotherapy. Given this complexity, it is essential to explore different strategies for sustaining weight reduction long term. One potential strategy involves transitioning from an injectable AOM to an oral AOM for maintenance of weight reduction.

Study GZPN aims to investigate whether switching from once-weekly injectable tirzepatide or semaglutide (maximum tolerated dose) to daily oral orforglipron 36 mg or maximum tolerated dose (24 mg or 36 mg) in participants with obesity or overweight with comorbidities (without T2D) can lead to superior long-term maintenance of body weight reduction compared to switching to placebo. Participants must have achieved clinically meaningful body weight reduction (≥5%) to be eligible for this study.

2.2. Background

Obesity or overweight

Obesity is a chronic disease associated with multiple complications such as T2D, CV disease, obstructive sleep apnea, osteoarthritis, increased risk for some cancers and increased risk for premature death (Allison et al. 2008; AMA 2013; CSAPH 2013). There is strong and consistent evidence that obesity management is beneficial in the treatment of T2D and weight-related comorbidities (ADA-EASD 2022). Lifestyle changes that result in modest and sustained weight loss produce clinically meaningful reductions in BG, HbA1c, and triglycerides (Look AHEAD Research Group et al. 2013). Greater weight reduction produces even greater metabolic benefits, including reductions in blood pressure, improvements in low-density lipoprotein and high-density lipoprotein cholesterol, and reductions in the need for medications to control BG, blood pressure, and lipids, and may even result in achievement of glycemic goals in the absence of glucose-lowering agent use in some patients (UKPDS Group 1990; Pastors et al. 2002; Wing et al. 2011; Rothberg et al. 2017; ADA 2023).

GLP-1 is secreted after meal ingestion and mediates the incretin effect, beta-cell neogenesis and proliferation, and protects beta cells from apoptosis. It also exerts actions on alpha cells, modifying glucagon secretion (Skow et al. 2016). Based on these properties, several GLP-1

receptor agonists have been approved for pharmacological treatment of T2D (Tomlinson et al. 2016).

In addition to its pancreatic effects, GLP-1 receptor activation decreases gut motility, slows gastric emptying, and promotes satiety (presumably through a combination of GLP-1 receptor activation in the central and peripheral nervous system), thereby regulating food intake and body weight (Baggio and Drucker 2007). With the advent of injectable incretin-based therapies, safe, highly efficacious, and well-tolerated medications are increasingly available. Incretin-based therapies have been able to overcome the efficacy and safety issues that have challenged this therapeutic space for decades. Specifically, semaglutide and liraglutide (Wegovy® package insert, 2023 and Saxenda® package insert, 2023), both injectable peptide GLP-1 receptor agonists, have been shown to be safe and effective for the treatment of obesity and overweight and have established CV safety. More recently, GIP/GLP-1 receptor agonist tirzepatide (Zepbound package insert, 2023) was approved for chronic weight management.

Results of clinical trials suggest that clinically meaningful reductions in body weight are expected for those patients that take and remain on treatment with incretin-based AOMs (Garvey et al. 2022; Aronne et al. 2024). However, in the real-world clinical setting, long-term persistence on AOM remains challenging for multifactorial reasons including cost, supply/access, and patient or provider preference. Trials such as STEP-1 extension study, STEP-4 and SURMOUNT-4 have demonstrated that there is a strong likelihood of weight regain when discontinuing therapy (Rubino et al. 2021; Wilding et al. 2022; Aronne et al. 2024). Therefore, multiple strategies are necessary, including switching from an injectable AOM to oral AOM, to provide individualized treatment options for patients to maintain the weight reduction long-term.

This Phase 3b study will evaluate the efficacy and safety of once daily orforglipron 36 mg or maximum tolerated dose of orforglipron (24 mg or 36 mg) compared with placebo for the maintenance of body weight reduction in participants who have achieved body weight reduction after 72 weeks of treatment with either tirzepatide 10 mg or 15 mg or semaglutide 1.7 mg or 2.4 mg.

Clinical data for orforglipron

A detailed description of the chemistry, pharmacology, efficacy, and safety of orforglipron is provided in the IB.

The clinical pharmacology, PK, and PD of orforglipron were initially studied in 2 Phase 1 Studies, J2A-MC-GZGA (GZGA) in healthy volunteers and J2A-MC-GZGC (GZGC) in patients with T2D. Results from these studies demonstrated a PK profile appropriate for once daily oral dosing that can be administered without limitations pertaining to food or water intake or time of day.

Two Phase 2 studies have evaluated the safety and efficacy of orforglipron: Study J2A-MC-GZGE (GZGE) in participants with T2D and Study J2A-MC-GZGI (GZGI) in participants with obesity or overweight and at least 1 weight-related comorbidity.

Study GZGI was a Phase 2, randomized, double-blind, parallel, placebo-controlled 36-week study (Wharton et al. 2023). The objective of the study was to investigate efficacy and safety of 4 dose levels (12 mg, 24 mg, 36 mg, and 45 mg) QD orforglipron compared with QD placebo in

participants who have obesity or overweight with weight-related complication at 26 weeks (primary endpoint) and 36 weeks (secondary endpoint).

Key efficacy and safety data at 36-weeks

- Change from baseline in percent body weight reduction at Week 36 ranged from –9.4% (12 mg) to –14.7% (45 mg) compared with placebo –2.3%.
- At Week 26, up to 90% of participants lost ≥5% and 71% lost ≥10% of body weight, with continued weight loss evident for participants completing Week 36.
- The overall safety profile of orforglipron was consistent with that established for the GLP-1 RA class, with most common TEAEs being GI related (nausea, vomiting, diarrhea, and constipation).
- The majority of the GI AEs were mild to moderate in severity and occurred mostly during dose escalation period.

Data from Phase 2 studies supported further clinical development of orforglipron.

2.3. Benefit/Risk Assessment

Detailed information about the known and expected benefits and risks and reasonably expected AEs of orforglipron may be found in the IB.

2.3.1. Risk Assessment

Study intervention

The potential risks associated with orforglipron are similar to those of marketed GLP-1 receptor agonists. The most commonly reported TEAEs observed in the orforglipron clinical studies, in healthy participants or participants with obesity or overweight with weight-related complications or T2D are GI effects, including nausea, vomiting, diarrhea, and constipation. Most were mild to moderate in severity and tended to occur during the dose escalation period.

Refer to Section 6.2 of the IB for detailed description of potential risks for orforglipron.

Management of risks

Sections 5.1, 5.2, and 8.2 address mitigation, management, and monitoring of the known potential risks associated with orforglipron.

2.3.2. Benefit Assessment

The known pharmacology of GLP-1 receptor agonism and Phase 2 studies of orforglipron support an expectation of such benefits as body weight reduction with orforglipron. Improvements in some cardiometabolic risk factors, including blood pressure and serum lipids, may also be expected.

Participants may also benefit from receiving personal health information, routine safety assessments, lifestyle management counseling, and frequent engagement with health care providers during the study, which provide opportunities for coaching and support.

2.3.3. Overall Benefit Risk Conclusion

The safety and efficacy profile seen to date for orforglipron supports the overall benefit/risk for participants in this study. The anticipated risks are those associated with known pharmacologic

effects of GLP-1 RAs, namely GI tolerability, and increased heart rate. These risks can be monitored, are usually mild to moderate in severity, reversible, and readily manageable. To date there are no recognized AEs from orforglipron other than those related to GLP-1 receptor agonism.

The potential risks based on the knowledge for the GLP-1 RA class are considered to be acceptable in the context of the potential benefits anticipated from treatment with orforglipron in adult participants with obesity or overweight.

3. Objectives, Endpoints, and Estimands

3.1. Participants Treated with Tirzepatide in SURMOUNT-5 Study

Objectives	Endpoints
**Primary**	
To demonstrate that orforglipron 36 mg or MTD (24 mg or 36 mg) is superior to placebo at Week 52 for the mean percent maintenance of BW reduction achieved with 72 weeks of treatment with tirzepatide 15 mg or MTD (10 mg or 15 mg) in participants who have reached a BW plateau[a]	Percent maintenance of BW reduction achieved during the 72 weeks of tirzepatide treatment
**Key Secondary**	
To demonstrate that orforglipron 36 mg or MTD (24 mg or 36 mg) is superior to placebo at Week 52 for the mean percent change in BW from SURMOUNT-5 baseline in all participants	Percent change in BW from SURMOUNT-5 baseline prior to the initiation of tirzepatide treatment
To demonstrate that orforglipron 36 mg or MTD (24 mg or 36 mg) is superior to placebo at Week 52 for the mean percent maintenance of BW reduction achieved with 72 weeks of treatment with tirzepatide 15 mg or MTD (10 mg or 15 mg) in all participants	Percent maintenance of BW reduction achieved during the 72 weeks of tirzepatide treatment
To demonstrate that orforglipron 36 mg or MTD (24 mg or 36 mg) is superior to placebo at Week 52 for maintenance of BW reduction achieved with 72 weeks treatment of tirzepatide 15 mg or MTD (10 mg or 15 mg) in participants who have reached a BW plateau[a]	Assessment (yes/no) of maintaining ≥80% of the BW reduction achieved during 72 weeks of tirzepatide treatment
**Additional Secondary**	
To demonstrate that orforglipron 36 mg or MTD (24 mg or 36 mg) is superior to placebo at Week 52 for maintenance of BW reduction achieved with 72 weeks treatment of tirzepatide 15 mg or MTD (10 mg or 15 mg) in participants who have reached a BW plateau[a]	Assessment (yes/no) of maintaining ≥15% BW reduction from SURMOUNT-5 baseline for participants who have already lost ≥15% BW after 72-week of tirzepatide treatment
To compare orforglipron 36 mg or MTD (24 mg or 36 mg) to placebo at Week 52 in participants who have reached a BW plateau[a] for BW endpoints	• From randomization to Week 52<br>o change in BW (kg)<br>o percent change in BW<br>o change in waist circumference (cm)
To compare orforglipron 36 mg or MTD (24 mg or 36 mg) to placebo at Week 24 in participants prior to receiving rescue orforglipron who have reached a BW plateau[a] for mean percent maintenance of BW reduction achieved during the 72 weeks of tirzepatide treatment	Percent maintenance of BW reduction achieved during the 72 weeks of tirzepatide treatment

Objectives	Endpoints
To describe the safety of orforglipron 36 mg or MTD (24 mg or 36 mg) in all participants enrolled in the study	• Summary of safety data, including number and incidence of<br>o SAEs<br>o TEAEs<br>o discontinuations due to AEs
**Exploratory**	
To evaluate orforglipron 36 mg and/or MTD (24 mg or 36 mg) to placebo at Week 52 in participants who have reached a BW plateau[a] before randomization for change in:	Change from before randomization at Week 52
• lipid parameters	• total cholesterol<br>• HDL-cholesterol<br>• LDL-cholesterol<br>• VLDL-cholesterol<br>• non-HDL cholesterol<br>• triglycerides
• blood pressure parameters	systolic blood pressure (mmHg).
• glycemic parameters	• fasting glucose (mg/dL)<br>• fasting insulin, and<br>• HbA1c (%)
• rescue AOM use	Use of rescue orforglipron for weight regain ≥50% of the BW reduction achieved with tirzepatide treatment
• BW endpoints	• Change in BMI (kg/m^2)<br>• Assessment (yes/no) of maintenance of BW reduction ≥20% BW reduction from SURMOUNT-5 baseline for participants who have already lost ≥20% with tirzepatide treatment
• patient-reported outcomes	• SF-36v2 acute form domain and summary scores<br>• CoEQ scores<br>• Proportion of participants with improved categorical shift in:<br>• PGIS-Physical Function Weight<br>• PGIS-Food Craving<br>• Proportion of participants with improvements in:<br>• PGIC-Physical Function Weight<br>• PGIC-Food Craving<br>• rPDQS score

Abbreviations: AE = adverse event; BMI = body mass index; BW = body weight; CoEQ = Control of Eating Questionnaire-NRS; HbA1c = hemoglobin A1c; HDL = high-density lipoprotein; LDL = low-density lipoprotein; MTD = maximum tolerated dose; PGIC = Patient Global Impression of Change; PGIS = Patient Global Impression of Severity; rPDQS = rapid Prime Diet Quality Score; SAE = serious adverse event; SF-36v2 = Short Form-36

version 2 Health Survey acute form; SURMOUNT-5 = Study I8F-MC-GPHJ (GPHJ); TEAE = treatment-emergent adverse event; VLDL = very-low-density lipoprotein.

[a] BW plateau is defined as <5% BW change between Visit 17 (Week 60) and Visit 20 (Week 72) of SURMOUNT-5 study.

Approved on 10 Nov 2025 GMT

3.2. Participants Treated with Semaglutide in SURMOUNT-5 Study

Objectives	Endpoints
**Primary**	
To demonstrate that orforglipron 36 mg or MTD (24 mg or 36 mg) is superior to placebo at Week 52 for the mean percent maintenance of BW reduction achieved with 72 weeks of treatment with semaglutide 2.4 mg or MTD (1.7 mg or 2.4 mg) in participants who have reached a BW plateau[a]	Percent maintenance of BW reduction achieved during the 72 weeks of semaglutide treatment
**Key Secondary**	
To demonstrate that orforglipron 36 mg or MTD (24 mg or 36 mg) is superior to placebo at Week 52 for the mean percent change in BW from SURMOUNT-5 baseline in all participants	Percent change in BW from SURMOUNT-5 baseline prior to the initiation of semaglutide treatment
To demonstrate that orforglipron 36 mg or MTD (24 mg or 36 mg) is superior to placebo at Week 52 for the mean percent maintenance of BW reduction achieved with 72 weeks of treatment with semaglutide 2.4 mg or MTD (1.7 mg or 2.4 mg) in all participants	Percent maintenance of BW reduction achieved during the 72 weeks of semaglutide treatment
To demonstrate that orforglipron 36 mg or MTD (24 mg or 36 mg) is superior to placebo at Week 52 for maintenance of BW reduction achieved with 72 weeks of treatment with semaglutide 2.4 mg or MTD (1.7 mg or 2.4 mg) in participants who have reached a BW plateau[a]	Assessment (yes/no) of maintaining ≥80% of the BW reduction achieved during 72 weeks of semaglutide treatment
**Additional Secondary**	
To demonstrate that orforglipron 36 mg or MTD (24 mg or 36 mg) is superior to placebo at Week 52 for maintenance of BW reduction achieved with 72 weeks treatment of semaglutide 2.4 mg or MTD (1.7 mg or 2.4 mg) in participants who have reached a BW plateau[a]	Assessment (yes/no) of maintaining ≥15% BW reduction from SURMOUNT-5 baseline for participants who have already lost ≥15% BW after 72 weeks of semaglutide treatment
To compare orforglipron 36 mg or MTD (24 mg or 36 mg) to placebo at Week 52 in participants who have reached a BW plateau[a] for BW endpoints	• From randomization to Week 52:<br>o change in BW (kg)<br>o percent change in BW<br>o change in waist circumference (cm)
To compare orforglipron 36 mg or MTD (24 mg or 36 mg) to placebo at Week 24 in participants prior to receiving rescue orforglipron who have reached a BW plateau[a] for mean percent maintenance of BW reduction achieved during the 72 weeks of semaglutide treatment	Percent maintenance of BW reduction achieved during the 72 weeks of semaglutide treatment

Objectives	Endpoints
To describe the safety of orforglipron 36 mg or MTD (24 mg or 36 mg) in all participants enrolled in the study	• Summary of safety data, including number and incidence of<br>o SAEs<br>o TEAEs<br>o discontinuations due to AEs
**Exploratory**	
To compare orforglipron 36 mg and/or MTD (24 mg or 36 mg) to placebo at Week 52 in participants who have reached a BW plateau[a] before randomization for change in:	Change from before randomization at Week 52
• lipid parameters	• total cholesterol<br>• HDL-cholesterol<br>• LDL-cholesterol<br>• VLDL-cholesterol<br>• non-HDL cholesterol<br>• triglycerides
• blood pressure parameters	systolic blood pressure (mmHg)
• glycemic parameters	• fasting glucose (mg/dL)<br>• fasting insulin<br>• HbA1c (%)
• rescue AOM use	Use of rescue orforglipron for weight regain ≥50% of the BW reduction achieved with semaglutide treatment.
• BW endpoints	• Change in BMI (kg/m^2).<br>• Assessment (yes/no) of maintenance of BW reduction ≥20% BW reduction from SURMOUNT-5 baseline for participants who have already lost ≥20% with semaglutide treatment
• patient-reported outcomes	• SF-36v2 acute form domain and summary scores<br>• CoEQ scores<br>• Proportion of participants with improved categorical shift in:<br>• PGIS-Physical Function Weight<br>• PGIS-Food Craving<br>• Proportion of participants with improvements in:<br>• PGIC-Physical Function Weight<br>• PGIC-Food Craving<br>• rPDQS score

Abbreviations: AE = adverse event; BMI = body mass index; BW = body weight; CoEQ = Control of Eating Questionnaire-NRS; HbA1c = hemoglobin A1c; HDL = high-density lipoprotein; LDL = low-density lipoprotein; MTD = maximum tolerated dose; PGIC = Patient Global Impression of Change; PGIS = Patient Global Impression

of Severity; rPDQS = rapid Prime Diet Quality Score; SAE = serious adverse event; SF-36v2 = Short Form-36 version 2 Health Survey acute form; SURMOUNT-5 = Study I8F-MC-GPHJ (GPHJ); TEAE = treatment-emergent adverse event; VLDL = very-low-density lipoprotein.

[a] BW plateau is defined as <5% BW change between Visit 17 (Week 60) and Visit 20 (Week 72) of SURMOUNT-5 study.

3.3. Estimands

There will be 2 estimands planned in the study, modified treatment-regimen estimand and efficacy estimand. Unless otherwise specified or requested by a regulatory agency, modified treatment-regimen estimand will be the primary estimand, with the efficacy estimand considered supportive. Both the modified treatment-regimen and efficacy estimands will be evaluated for the primary and the key secondary objectives.

Modified treatment-regimen estimand

For each treatment in the SURMOUNT-5 study (tirzepatide or semaglutide), the clinical question of interest is:

What is the treatment difference at Week 52 for orforglipron versus placebo in mean percent maintenance of BW reduction achieved during 72 weeks of treatment with tirzepatide or semaglutide, respectively, as an adjunct to a reduced-calorie diet and increased physical activity in participants with obesity or overweight with at least 1 weight-related comorbid condition, regardless of treatment discontinuation for any reasons and regardless of initiation of other AOMs, GLP-1 RAs, GIP/GLP-1 RAs, or DPP-4 inhibitors. This estimand also assumes that participants who had bariatric surgery or another weight-loss procedure or took rescue orforglipron would not have received any additional improvement from their randomized study treatment?

Rationale for the modified treatment-regimen estimand

This estimand aims at reflecting how participants with obesity or overweight with at least 1 weight-related comorbid condition are treated in clinical practice and takes into account both tolerability and efficacy.

Modified treatment-regimen estimand attributes

- **Population** - Participants who meet the eligibility criteria. Further details can be found in Section 5.
- **Endpoints** - Mean percent maintenance of BW reduction achieved during the 72 weeks of treatment with tirzepatide or semaglutide, respectively.
- **Treatment condition -** The randomized treatment as an adjunct to a reduced-calorie diet and increased physical activity regardless of adherence to treatment with or without other AOMs, GLP-1 RAs, GIP/GLP-1 RA, or DPP-4 inhibitors. Further details on study treatment and concomitant therapy can be found in Section 6.
- **Intercurrent events** - "treatment discontinuation for any reason" and "initiation of other AOMs, GLP-1 RAs, GIP/GLP-1 RA, or DPP-4 inhibitor" are addressed by the treatment condition. Bariatric surgery or other weight-loss procedures or rescue orforglipron will be

addressed by the hypothetical strategy. It will be assumed that participants who undergo bariatric surgery, weight-loss procedures, or rescue orforglipron would not have received any additional improvement from their randomized treatment.

- **Population-level summary and treatment effect of interest** - Difference in the mean of the endpoint between treatment conditions at Week 52.

Efficacy estimand

For each treatment in the SURMOUNT-5 study (tirzepatide or semaglutide), the clinical question of interest is:

What is the treatment difference at Week 52 for orforglipron versus placebo in mean percent maintenance of BW reduction achieved during 72 weeks of treatment with tirzepatide or semaglutide, respectively, as an adjunct to a reduced-calorie diet and increased physical activity in participants with obesity or overweight with at least 1 weight-related comorbid condition, assuming that participants had stayed on treatment, had not taken other AOMs, GLP-1 RAs, GIP/GLP-1 RAs, or DPP-4 inhibitors, had not had bariatric surgery or other weight management procedures, and assuming that participants who took rescue orforglipron would not have received any additional improvement from their randomized study treatment?

Rationale for the efficacy estimand

This estimand focuses on the treatment effect if participants who underwent randomization continued to receive the study treatment without taking other AOMs, GLP-1 RAs, GIP/GLP-1 RA, or DPP-4 inhibitors, bariatric surgery or weight-loss procedures, or rescue orforglipron.

Efficacy estimand attributes

- **Population –** Participants who meet the eligibility criteria. Further details can be found in Section 5.
- **Endpoints** – Mean percent maintenance of BW reduction achieved during the 72 weeks of treatment with tirzepatide or semaglutide, respectively.
- **Treatment condition –** The randomized treatment as an adjunct to a reduced-calorie diet and increased physical activity. Further details on study treatment can be found in Section 6.
- **Intercurrent events –** "Treatment discontinuation for any reason," "Initiation of other AOMs, GLP-1 RAs, GIP/GLP-1 RAs or DPP-4 inhibitors," "having bariatric surgery or other weight management procedures," or "taking rescue *orforglipron*" will be addressed using the hypothetical strategy:
    - had participants stayed on treatment
    - had participants not taken other AOMs, GLP-1 RAs, GIP/GLP-1 RAs, or DPP-4 inhibitors and had not had bariatric surgery or other weight-loss procedure, and
    - assuming that participants who took rescue orforglipron would not have received any additional improvement from their randomized treatment.

- **Population-level summary and treatment effect of interest** – Difference in mean of the endpoint between treatment conditions at Week 52.

4. Study Design

4.1. Overall Design

Study GZPN is a Phase 3b, randomized, double-blind, placebo-controlled study designed to evaluate the efficacy and safety of orforglipron 36 mg or MTD (24 mg or 36 mg) administered QD compared with placebo, in achieving maintenance of BW reduction from the 72 weeks of tirzepatide or semaglutide treatment in participants who have obesity or overweight with weight-related comorbidities and previously participated in SURMOUNT-5 study.

This study will consist of 3 periods:

- screening and lead-in period: up to 2 weeks
- treatment period: 52 weeks, including dose escalation and maintenance dose, and
- safety follow-up period: 2 weeks.

Incident diabetes during the treatment period

Participants will be monitored throughout the study for incident diabetes. For the definition of incident diabetes, confirmation of diabetes diagnosis, recording of incident diabetes events, and management of incident diabetes, refer to Section 10.8. The investigator must report these events as an AE as described in Section 8.3.1. Participants with incident diabetes during the study will continue participation in the study with study intervention unless discontinuation criteria are met (Section 7).

Refer to the SoA (Section 1.3) for visit details.

For the randomization ratio and stratification factors, refer Section 6.3.

4.2. Scientific Rationale for Study Design

Choice of primary endpoint

Study GZPN aims to evaluate the orforglipron 36 mg dose or MTD (24 mg or 36 mg) for chronic maintenance of BW reduction achieved with injectable AOM, specifically tirzepatide or semaglutide, by comparing orforglipron versus placebo. Current data suggest the loss of health benefits if AOM is discontinued after achieving BW reduction and significant weight regain is allowed to occur without further weight management intervention (Wadden et al. 2013; Rubino et al. 2021; Wilding et al. 2022). While it is unknown whether the weight reduction achieved with injectable AOM could be maintained long-term by switching to an oral AOM, such strategy may be valuable and needed to provide options for participants to manage and tailor chronic AOM treatment.

Data generated from this study may inform and guide clinicians and participants on long-term AOM use, specifically switching from injectable AOM to oral AOM, to maintain BW reduction and explore durability of related health benefits associated with chronic maintenance of BW reduction.

Treatment with AOM usually leads to initial BW reductions that level out or “plateau.” Weight plateau is likely due to metabolic adaptation rather than poor response or resistance to AOM

(Garvey et al. 2016; Ard et al. 2021). There is no established definition of BW plateau, whereas clinically meaningful BW reduction is considered 5% or greater (FDA 2007; Garvey et al. 2016). On the other hand, 1% to 2% change in BW is considered within normal physiological fluctuation (Stevens et al. 2006). Therefore, less than 5% BW change may be considered a reasonable cutoff to define a stable BW following an initial BW reduction.

While there is no clear consensus on the definition of weight maintenance, data from Look AHEAD demonstrated that those who maintained at least 75% of the initial BW reduction had the most cardiometabolic benefit (Berger et al. 2019). Similarly, a study in a bariatric surgery cohort found that 20% or greater weight regain correlated with cardiometabolic risk factors (King 2018). Based on these data, maintaining 75% to 80% of initial BW reduction regardless of therapeutic modality appears clinically beneficial.

Choice of comparator

A placebo comparator was selected for this trial in accordance with regulatory guidance (FDA 2007; EMA 2016). All participants, regardless of treatment assignment, will receive lifestyle modification counseling consistent with current guidelines for weight management (Jensen et al. 2014). Specifically, participants will consult with a dietician, or equivalent qualified delegate, throughout the study to focus on a healthy diet and physical activity. Additionally, all participants will be provided with rescue orforglipron if exceeding the weight regain threshold set in this study (Garvey et al. 2016) (refer Section 6.9.5).

Study duration

The planned duration of treatment for the primary endpoint is at 52 weeks. This duration is considered appropriate to assess the full effects and benefit/risk of orforglipron compared with placebo on body weight and is consistent with regulatory guidelines (FDA 2007; EMA 2016).

Concomitant medications

To minimize the potential confounding effect of changes to concomitant medications, participants will be permitted to use concomitant medications that do not interfere with the assessment of efficacy or safety characteristics of the study intervention (refer Sections 6.9 and 10.8).

Collection of race and ethnicity data

In this study, collection of demographic information includes race and ethnicity. The scientific rationale is based on the need to assess variable response in safety and/or efficacy based on race or ethnicity. This question can be answered only if all the relevant data are collected.

4.3. Justification for Dose

The orforglipron daily dose of 36 mg or MTD (24 mg or 36 mg) QD administered orally will be evaluated in this study. The 36 mg dose was selected as the highest dose based upon the greater numerical placebo-adjusted observed body weight change in Study GZGI of -10.2% (95% CI -12.4% to -8.0%) compared to the 12 mg dose. At the higher maintenance dose of 45 mg, there was no clinically meaningful improvement in weight reduction or HbA1c reduction beyond that observed at 36 mg.

The dose escalation method was selected to optimize GI tolerability based on assessment of different starting doses and escalation intervals used in the Phase 1 and 2 studies. Additionally, given that the study participants completed 72-week treatment with tirzepatide or semaglutide prior to enrollment, a higher starting dose of orforglipron 12 mg should be reasonably tolerated in this group while avoiding any potential weight regain that may occur by starting at a lower starting dose of orforglipron. The totality of these results suggested a lower starting dose of 12 mg for 4 weeks prior to escalating to 24 mg for 4 weeks, and then escalating to 36 mg if tolerated (Section 6.1).

4.4. End of Study Definition

The end of the study is defined as the date of the last visit of the last participant in the study or last scheduled procedure shown in the SoA for the last participant.

A participant is considered to have completed the study if the participant has completed all periods of the study including the last visit or the last scheduled procedure shown in the SoA.

5. Study Population

Participant eligibility for enrollment in the study is based on the criteria listed in this section. The inclusion and exclusion criteria used to determine eligibility should only apply at screening or other specified visits, and not continuously throughout the study.

Prospective approval of protocol deviations to recruitment and enrollment criteria, also known as protocol waivers or exemptions, is not permitted.

5.1. Inclusion Criteria

Participants are eligible to be included in the study only if all of the following criteria apply:

Type of participant and disease characteristics

1. Completed SURMOUNT-5 study on study treatment, and completed Visits 2, 17, and 20 (Weeks 0, 60, and 72 respectively) of SURMOUNT-5 study.
2. Have lost ≥5% of BW during 72-week treatment with tirzepatide or semaglutide in SURMOUNT 5 study.

Sex assigned at birth and contraceptive/barrier requirements

3. Assigned male at birth/assigned female at birth

   Contraceptive use by participants should be consistent with local regulations regarding the methods of contraception for those participating in clinical studies. For the contraception requirements of this protocol, refer Section 10.4.

Study procedures

4. Are reliable and willing to make themselves available for the duration of the study and are willing and able to follow study procedures for the duration of the study.

Informed consent

5. Are capable of giving signed informed consent as described in Section 10.1, which includes compliance with the requirements and restrictions listed in the ICF and in this protocol.

5.2. Exclusion Criteria

Participants are excluded from the study if any of the following criteria apply:

Medical conditions

Diabetes related

6. Have T1D, T2D, or any other types of diabetes, history of ketoacidosis, or hyperosmolar state/coma.
   Note: Participants with a history of gestational diabetes are eligible to participate in this trial.
7. Have at least 1 laboratory value suggestive of diabetes during screening.

- HbA1c ≥6.5% (≥48 mmol/mol), or
- FSG ≥126 mg/dL (≥7.0 mmol/L) (refer Section 10.8).

Obesity related

8. Have a BMI of 22 kg/m^2 or lower.
9. Prior or planned surgical treatment for obesity.

   Note: The following are allowed if performed >1 year before Visit 1:
   - Liposuction
   - Abdominoplasty, or
   - Cryolipolysis.
10. Have a prior or planned endoscopic (for example, mucosal ablation or gastric artery embolization) and/or device-based (for example, intragastric balloon, or duodenal-jejunal endoluminal liner) therapy for obesity.

    Note: Prior device-based therapy is acceptable if device removal was more than 180 days prior to Visit 1.
11. Have obesity induced by other endocrinologic disorders, for example Cushing syndrome or diagnosed monogenetic or syndromic forms of obesity, such as melanocortin 4 receptor deficiency or Prader-Willi syndrome.

Renal

12. Have an eGFR <15 mL/min/1.73 m^2, calculated by Chronic Kidney Disease-Epidemiology cystatin C equation, as determined by central laboratory at Visit 1.

Autoimmune

13. Have evidence of significant, active autoimmune abnormality, for example, lupus, rheumatoid arthritis, that, in the opinion of the investigator, is likely to require concurrent treatment with systemic glucocorticoids during the course of the study.

Cardiovascular

14. Have had any of the following CV conditions anytime between SURMOUNT-5 study participation and this screening visit
    - acute myocardial infarction
    - cerebrovascular accident (stroke)
    - coronary artery revascularization
    - unstable angina, or
    - hospitalization due to congestive heart failure.
15. Have New York Heart Association Functional Classification IV congestive heart failure.

Hepatic

16. Have acute or chronic hepatitis including a history of autoimmune hepatitis, signs and symptoms of any other liver disease other than nonalcoholic fatty liver disease, or any of the following, as determined by the central laboratory at Visit 1:
    - ALT or AST level ≥3.0x the ULN for the reference range
    - ALP level ≥1.5x the ULN for the reference range
    - TBL level ≥1.5x the ULN for the reference range, except for cases of known Gilbert's syndrome
    - Hepatitis B infection, defined as:
        - positive hepatitis B core antibody (HBcAb) and positive hepatitis B virus (HBV) DNA, or
        - positive hepatitis B surface antigen
    - Positive Hepatitis C antibody and positive hepatitis C virus (HCV) RNA.

    Note: Participants with nonalcoholic fatty liver disease (now known as metabolic associated steatotic liver disease) are eligible to participate in this study if their ALT level is <3.0x the ULN for the reference range.

Endocrine

17. Evidence of significant uncontrolled endocrine abnormality, for example, thyrotoxicosis or adrenal crises, in the opinion of the investigator.
18. Have family (first-degree relative) or personal history of MTC or MEN2 syndrome.
19. Have a calcitonin level determined by the central laboratory at Visit 1 of
    - ≥20 ng/L, if eGFR ≥60 mL/min/1.73 m^2, or
    - ≥35 ng/L, if eGFR <60 mL/min/1.73 m^2.

Malignancy

20. History of an active or untreated malignancy or are in remission from a clinically significant malignancy.

    Exceptions: basal or squamous cell skin cancer, in situ carcinomas of the cervix or in situ or Grade 1 prostate cancer (for example, Gleason 6 or lower).

Hematology

21. Have any hematological condition that may interfere with HbA1c measurement, for example, hemolytic anemias, sickle cell disease.

Psychobehavioral

22. Have a history of active or unstable major depressive disorder or other severe psychiatric disorder, such as schizophrenia, bipolar disorder, or other serious mood or anxiety disorder, within the last 2 years.

    or

In the investigator's opinion, have any significant mental health disorder that may put the individual at higher risk of study participation.

Note: In the investigator's opinion, individuals whose disease state is considered stable for the past 2 years and expected to remain stable throughout the course of the study may be considered for inclusion if they do not meet exclusion criterion #36 regarding weight gain-promoting concomitant medications.

23. Have a PHQ-9 score of 15 or more at Visit 1 or Visit 2.
24. Are, in the judgment of the investigator, actively suicidal and therefore deemed to be at significant risk for suicide.
25. Have answered "yes" to either Question 4 or Question 5 on the "Suicidal Ideation" portion of the C-SSRS **and** the ideation occurred within the past month prior to Visit 1 or Visit 2.

    or

    Have answered "yes" to any of the suicide-related behaviors on the "Suicidal Behavior" portion of the C-SSRS, **and** the behavior occurred within the past month prior to Visit 1 or Visit 2.

General

26. Have any condition, unwillingness, or inability, not covered by any of the other exclusion criteria, which in the investigator's opinion, might jeopardize the participant's safety (for example, hypersensitivity or contraindication) or compliance with the protocol (for example, recreational drug use or alcohol abuse).
27. At the time of screening have a planned surgery (except for minor surgical procedures) to occur during the course of the study.
28. Had chronic or acute pancreatitis any time prior to Visit 1.
29. Have had a transplanted organ or awaiting an organ transplant.

    Exception: corneal transplants (keratoplasty).

30. Have a history of use of marijuana or tetrahydrocannabinol-containing products within 3 months of enrollment or unwillingness to abstain from marijuana or tetrahydrocannabinol-containing products use during the trial.

    Note: If a participant has used cannabidiol oil during the past 3 months but agrees to refrain from use for the duration of the study, the participant can be enrolled.

31. Uncontrolled hypertension (systolic ≥160 mmHg and or diastolic ≥100 mmHg).
32. History of any other condition that in the opinion of the investigator may preclude the participant from following and completing the study.

Prior/concomitant therapy

33. Are receiving metformin or any other glucose-lowering medication, regardless of the indication for use, within 90 days prior to Visit 1, or between Visit 1 and Visit 2 (refer Section 10.7.1.3 for more details).
34. Are receiving chronic (>14 days) systemic glucocorticoid therapy (excluding topical, intra-ocular, intranasal, inhaled, or intra-articular preparations) or have received such therapy within 90 days prior to Visit 1, or between Visit 1 and Visit 2.
35. Have used any anti-obesity medication (except for use of injectable tirzepatide or semaglutide treatment provided by the SURMOUNT-5 study), anytime between SURMOUNT-5 study participation and this screening visit (refer Section 10.7.1.1 for more details).
36. Have initiated treatment with or changed dose of medications that may cause significant weight gain, including but not limited to tricyclic antidepressants, atypical antipsychotics, and mood stabilizers (refer Section 10.7.1.2 for more details), within 12 months prior to Visit 1.
37. Have started implantable or injectable contraceptives within 18 months prior to Visit 1.
    Note: Intrauterine devices are acceptable.
38. Are receiving strong CYP3A inhibitors or CYP3A inducers, strong OATP inhibitors, or drugs that are sensitive P-gp/BCRP substrates with narrow therapeutic index (refer Section 10.7.1.4 for more details).

    Note: To be eligible for screening into this study, these drugs need to be washed out for at least 2 weeks prior to Visit 2 and the participant should be on a stable dose of alternative medications for at least 2 weeks prior to Visit 2.
39. Have known allergies or intolerance to GLP-1 receptor agonist or GIP/GLP-1 receptor agonist.

Other exclusion criteria

40. Are pregnant, intending to be pregnant, or intending to breastfeed.
41. Are investigator site personnel directly affiliated with this study and/or their immediate family. Immediate family is defined as a spouse, legal partner, parent, child, or sibling, whether biological or legally adopted.
42. Are Lilly employees or are employees of any third party involved in the study who require exclusion of their employees.

5.3. Lifestyle Considerations

Per the SoA (Section 1.3), participants will consult a dietitian, or equivalent qualified delegate, according to local standards, to receive lifestyle management counseling at Weeks 0, 4 and 8 during dose escalation period and then at Weeks 16, 24, 36, 48 and 52 during maintenance dose period.

Healthy diet and physical activity goals established during the lifestyle consultation and the importance of adherence to the lifestyle component of the study will be reinforced at each visit by study staff.

5.3.1. Meals and Dietary Restrictions

At Visit 2 and subsequent visits, study participants will receive diet counseling by a dietitian/nutritionist, or equivalent qualified delegate, according to local standard. Dietary counseling will consist of advice on healthy food choices and focus on calorie restriction using a hypocaloric diet with macronutrient composition of

- maximum 30% of energy from fat
- approximately 20% of energy from protein
- approximately 50% of energy from carbohydrates, and
- an energy deficit of approximately 500 kcal/day compared to the participant's estimated total energy expenditure.

To encourage adherence, it is recommended that a 3-day diet and exercise log be completed prior to each counseling visit. During each visit, the participant's diet is reviewed, and advice to maximize adherence is provided if needed.

The hypocaloric diet is continued after randomization and throughout the treatment period. If a BMI ≤22 kg/m^2 is reached, the recommended energy intake should be recalculated with no kcal deficit for the remainder of the trial.

Additionally, if a BMI ≤22 kg/m^2 is reached in participants receiving study intervention, the site physician should contact Lilly medical monitor for consideration of a single dose reduction for remainder of trial (for example, 36 mg to 24 mg or 24 mg to 12 mg of orforglipron).

Total energy expenditure is calculated by multiplying the estimated basal metabolic rate (BMR) (see table below) with a Physical Activity Level value of 1.3 (FAO/WHO/UNU 2004), which reflects an inactive lifestyle. This calculation provides a conservative estimate of caloric requirements:

Total Energy Expenditure (kcal/day) = basal metabolic rate (BMR) X 1.3

Equations for estimating BMR in kcal/day[a]

Sex	Age	BMR (kcal/day)
Men	18-30 years	15.057 × actual weight in kg + 692.2
	31-60 years	11.472 × actual weight in kg + 873.1
	>60 years	11.711 × actual weight in kg + 587.7
Women	18-30 years	14.818 × actual weight in kg + 486.6
	31-60 years	8.126 × actual weight in kg + 845.6
	>60 years	9.082 × actual weight in kg + 658.5

Abbreviations: BMR = basal metabolic rate; WHO = World Health Organization.
[a] Revised WHO equations (adapted from: FAO/WHO/UNU 2004).

5.3.2. Monitoring Nutritional Needs

Similar to other GLP-1 receptor agonists, orforglipron acts, in part, by reducing appetite leading to a reduction in food intake. There is potential that a small portion of participants will have significantly reduced caloric intake.

Medical staff should consider clinical monitoring of the participant's nutritional and hydration status if there is report of significantly reduced caloric intake (for example, below 800-1200 kcal). By recognizing early signs of poor intake and dehydration, preventive actions can be taken to reduce the risk of potential complications.

5.3.3. Healthy Physical Activity

At Visit 2 and all subsequent visits, participants will be advised regarding achieving a healthy physical activity level (at least 150 minutes per week, as tolerated).

Counseling in lifestyle modification should be completed according to the SoA and must be documented in the participant's medical record. Adherence to the healthy diet and physical activity will be assessed at each study visit.

To encourage adherence to lifestyle modification, it is recommended that a 3-day diet and physical activity log be completed prior to each counseling visit.

5.3.4. Activity Before Blood Collections

Participants will abstain from strenuous physical activity for 8 hours before each blood collection for clinical laboratory tests.

5.3.5. Blood Donation

Study participants should be instructed not to donate blood or blood products during the study and for 2 weeks following the study.

5.3.6. Diabetes Education

Diabetes education, as well as a glucometer, will be provided to study participants who develop T2D (refer to Section 10.8) during the study. Education will be performed by personnel who are qualified to educate participants on symptoms and management of hyperglycemia and hypoglycemia, SMBG, and diabetes management, according to American Diabetes Association Standards of Medical Care in Diabetes (ADA 2023) or local standards.

Refer to Section 8.3.3.4 for management of hypoglycemia risk.

5.4. Screen Failures

A screen failure occurs when a participant who consents to participate in the clinical study is not subsequently assigned to study intervention. A minimal set of screen failure information is required to ensure transparent reporting of screen failure participants to meet the CONSORT publishing requirements and to respond to queries from regulatory authorities. Minimal information includes demography, screen failure details, and any SAE.

Individuals who do not meet the criteria for participation in this study (screen failure) will not be rescreened.

5.5. Criteria for Temporarily Delaying Enrollment of a Participant

Not applicable.

6. Study Intervention(s) and Concomitant Therapy

Study intervention is defined as any medicinal product(s) or medical device(s) intended to be administered to or used by a study participant according to the study protocol.

6.1. Study Intervention(s) Administered

This table lists the interventions used in this clinical study.

**Intervention Name**	Orforglipron	Placebo
**Dosage Level(s)**	1 mg, 3 mg, 6 mg, 12 mg, 24 mg, and 36 mg capsules	Capsule of orforglipron placebo to match
**Route of Administration**	Oral QD	Oral QD

Abbreviation: QD = once daily

Dose escalation

All participants will initiate treatment with a 12 mg QD dose of orforglipron or matching placebo and increase dose every 4 weeks until the randomized maintenance dose of 36 mg or MTD (24 mg or 36 mg) is reached as outlined in the below figure.

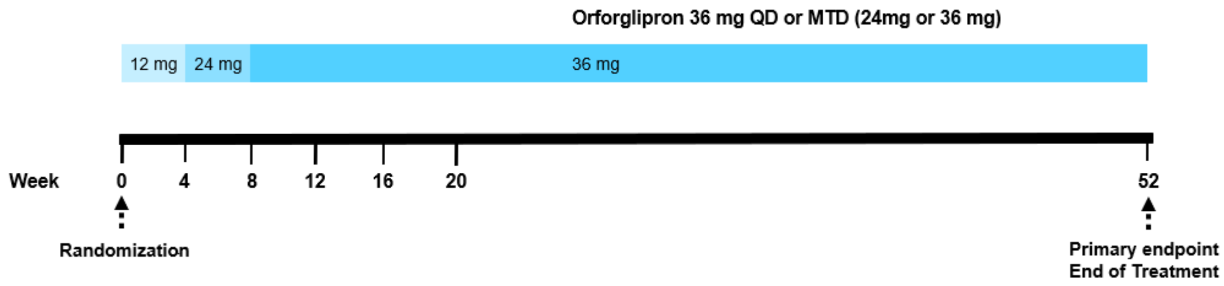

Abbreviation: MTD = maximum tolerated dose; QD = once daily.

Note: Orforglipron dose increases every 4 weeks in a blinded fashion until the randomized maintenance dose (36 mg) or MTD (24 mg or 36 mg) is reached.

The maintenance dose of orforglipron 36 mg or MTD (24 mg or 36 mg) or placebo will be continued for the remainder of the study (52 weeks from randomization). In participants who experience intolerable GI symptoms, the dose can be modified as described in Section 6.6.

Study intervention is administered orally once daily. In general, there are no restrictions on the time of day each dose is taken, but it is recommended to take the dose at approximately the same time each day. The participant will record the actual date and time of all dose administrations in a diary.

Participants should administer their first dose of study intervention at the end of Visit 2 prior to leaving the study site, after other study procedures and randomization are completed.

Packaging and labeling

Study interventions will be supplied by the sponsor or its designee in accordance with current Good Manufacturing Practice. Study interventions will be labeled as appropriate for country requirements.

6.2. Preparation, Handling, Storage, and Accountability

The investigator or designee must confirm appropriate storage conditions have been maintained during transit for all study intervention received and any discrepancies are reported and resolved before use of the study intervention.

Only participants enrolled in the study may receive study intervention. Only authorized study personnel may supply, prepare, or administer study intervention.

All study intervention must be stored in a secure, environmentally controlled, and monitored (manual or automated) area in accordance with the labeled storage conditions with access limited to the investigator and authorized study personnel.

The investigator or authorized study personnel are responsible for study intervention accountability, reconciliation, and record maintenance, that is, receipt, reconciliation, and final disposition records.

Further guidance and information for the final disposition of unused study interventions are provided in the study training materials.

6.3. Assignment to Study Intervention

Participants who meet all criteria for enrollment will be randomly assigned to study intervention using an IWRS.

Study intervention will be dispensed at the study visits summarized in the SoA. Returned study intervention should not be re-dispensed to the participants.

Participants will be randomly assigned in a 3:2 ratio to receive a daily dose of orforglipron 36 mg or MTD (24 mg or 36 mg), or placebo. All doses of study intervention capsules appear the same. Furthermore, placebo capsules look like study intervention capsules to maintain blinding.

For each treatment group in the SURMOUNT-5 study (tirzepatide or semaglutide), the randomization will be stratified by

- achieving plateau at Week 72 of SURMOUNT-5 study (yes/no)
- sex (individual AFAB, individual AMAB), and
- percent weight loss at Week 72 of SURMOUNT-5 study (<20% versus ≥20%).

Participants are considered to have achieved plateau if percent change in weight from Week 60 to Week 72 from SURMOUNT-5 study is less than 5%.

6.4. Blinding

This is a double-blind study. Investigators, site staff, clinical monitors, and participants will remain blinded to study intervention until the study is complete.

To maintain the blind, a minimum number of Lilly personnel will see the randomization table and treatment assignments before the study is complete.

If an investigator, site personnel performing assessments, or participant is unblinded, the participant must be permanently discontinued from study intervention, but should be continued in the study to be evaluated for efficacy and safety endpoints and monitored for all visits and testing. If a participant's intervention assignment is unblinded, the sponsor must be notified within 24 hours of this occurrence. The date and reason that the blind was broken must be recorded in the source documentation.

Emergency unblinding

In case of an emergency, the investigator has the sole responsibility for determining if unblinding of a participant's treatment assignment is warranted for medical management of the event. The participant's safety must always be the first consideration in making such a determination. If a participant's treatment assignment is unblinded, Lilly must be notified immediately.

If the investigator decides that unblinding is warranted, it is the responsibility of the investigator to promptly document the decision and rationale and notify Lilly as soon as possible.

Emergency unblinding may be performed through the IWRS. This option may be used ONLY if the participant's well-being requires knowledge of the participant's treatment assignment. All unblinding events are recorded and reported by the IWRS.

6.5. Study Intervention Compliance

Participant compliance with study intervention will be assessed at each visit. Compliance will be assessed by direct questioning and counting returned capsules and documented in the source documents. Participants will be instructed to return any unused study intervention capsules at the times specified in the SoA.

Further guidance and information for the final disposition of unused study interventions is provided in the pharmacy manual.

Treatment compliance for each visit interval is defined as taking at least 75% of the required doses of study intervention. Similarly, a participant will be considered significantly noncompliant if the participant is judged by the investigator to have intentionally or repeatedly taken more than the prescribed amount of medication (more than 125%).

In addition to the assessment of a participant's compliance with the study intervention administration, other aspects of compliance with the study will be assessed at each visit based on the participant's adherence to the visit schedule, completion of study diaries, and any other parameters the investigator considers necessary.

Participants considered to be nonadherent with their medication and/or the study procedures will receive additional training and instruction, as required, and will be reminded of the importance of complying with the protocol.

6.6. Dose Modification

The dose escalation period of the study occurs over the first 12 weeks to allow escalation to the maximum dose of 36 mg for those randomized to this treatment arm. All participants will

undergo each dose escalation step regardless of randomized treatment assignment. These dose escalation steps will be handled in a blinded fashion using the IWRS. During the dose escalation period, the investigator should make every effort to proceed through each dose escalation step per the study schedule for participants to achieve their randomized dose. Participants should continue on this dose for the duration of the treatment period.

Study intervention dose reduction during the entire course of the study is only permitted for management of intolerable GI symptoms (refer Section 6.6.1).

6.6.1. Management of Gastrointestinal Symptoms

All efforts should be made to prevent permanent discontinuation of study intervention. For participants who report intolerable GI symptoms during the study, the investigator should implement the following steps:

1	Advise participants to eat smaller meals, for example, splitting 3 daily meals into 4 or more smaller meals, and to stop eating when they feel full.
2	Continue #1, and prescribe symptomatic medication, for example, antiemetic or antidiarrheal medication, per local country availability and individual participant needs. Use of symptomatic medication should be captured as concomitant medication in the CRF.
3	Continue #1 and #2 and consider temporarily interrupting study intervention: omit up to 2 consecutive daily doses. Refer to Section 7.1.2 for information on temporary study intervention interruption.<br>After the interruption, the investigator should advise the participant to resume study intervention at the same dose level, with the participant taking medication to alleviate their GI symptoms.

If GI symptoms become tolerable or resolve with the above measures, the participant should continue study intervention at the same dose level until the next scheduled dose escalation step (if during the dose escalation period) or continue for the duration of the study if the participant has completed the final dose escalation step.

If intolerable GI symptoms persist, despite the above measures, the investigator should contact the sponsor to consider de-escalating to the next lower dose level for at least 4 weeks in a blinded fashion. De-escalation may be performed during an unscheduled dispensing visit.

The below table provides guidance for de-escalating the dose.

If a participant is currently taking…	Then de-escalate the dose for at least 4 weeks to…
6 mg/placebo	discontinue the study intervention and continue in the study
12 mg/placebo	6 mg/placebo
24 mg/placebo	12 mg/placebo
36 mg/placebo	24 mg/placebo

If GI symptoms become tolerable or resolve with dose de-escalation, the investigator should initiate re-escalation after at least 4 weeks have passed and only at a scheduled visit. For this purpose, if necessary, a telehealth visit may be performed as an office visit.

If the re-escalation attempt is tolerated, then the participant should continue to complete the remaining dose escalation steps to achieve the randomized maintenance dose per Section 6.1.

If the re-escalation attempt is not tolerated or if intolerable GI symptoms recur at any subsequent time point following the re-escalation, then depending on which dose level is achieved, the participant should undergo a final dose de-escalation to the next lower dose level or discontinue study intervention.

The below table provides guidance if the re-escalation attempt was not tolerated.

If the re-escalation attempt is not tolerated to reach this dose…	then the dose will be de-escalated for the remainder of the study to…
24 mg/placebo (by Week 24)	discontinue the study intervention and continue in the study
36 mg/placebo	24 mg/placebo

Please note the following

- only 2 re-escalation attempts are permitted during the entire course of the study to achieve a maximum dose of 36 mg, and
- only 1 de-escalation is permitted after re-escalation at each dose.

If intolerable GI symptoms persist despite symptomatic treatment, temporary drug interruption, and resumption of study intervention after the final dose de-escalation, the participant should be permanently discontinued from the study intervention. All participants who permanently discontinue study intervention should be encouraged to continue to attend all scheduled study visits.

All dose adjustments, for example, dose de-escalation or re-escalation, aside from planned dose escalation steps per Section 6.1 are to be recorded in the CRF.

For any participant receiving study intervention who has reached a BMI ≤22 kg/m^2, a single dose reduction may be considered. Refer Section 5.3.

6.7. Continued Access to Study Intervention after the End of the Study

Study intervention will not be made available to participants after conclusion of the study.

6.8. Treatment of Overdose

As any dose of orforglipron greater than 100 mg within a 24-hour time period will be considered a potential overdose, and considering the maximum dose any participant may receive during the study treatment period is 36 mg, for this blinded study, any dose of study intervention ≥3 capsules within a 24-hour time period will be considered a potential overdose and should be reported per criteria described in Section 10.3.1.

In the event of an overdose, the investigator should

- initiate supportive treatment according to the participant's clinical signs and symptoms
- contact the medical monitor immediately
- evaluate the participant to determine, in consultation with the medical monitor, whether study intervention should be interrupted or whether the dose should be reduced
- closely monitor the participant for any AE/SAE and laboratory abnormalities as medically appropriate until, for example, study intervention no longer has a clinical effect or can no longer be detected systemically (at least 7 days).

6.9. Prior and Concomitant Therapy

Prior therapies of interest, including, medications used for obesity or overweight, will be collected per the SoA (Section 1.3). Prior therapies are only those therapies that were received before enrollment in the study, that is, the stop date is prior to Visit 1.

Participants must consult with the investigator or a designated site staff member if they are prescribed any new medications during the study. If this is not possible due to treatment of medical emergencies, the participant will inform the investigator or a designated site staff member as soon as possible.

Any medication or vaccine, including over-the-counter or prescription medicines, vitamins, and/or herbal supplements that the participant is receiving at the time of enrollment or receives during the study must be recorded along with the

- reason for use
- dates of administration including start and end dates, and
- dosage information including dose and frequency for concomitant therapy of special interest.

In this study population, it is likely that many study participants will be taking medications to treat weight-related comorbidities, including hypertension and dyslipidemia. Since weight reduction is expected to improve these comorbidities, study participants may require a dose reduction or complete withdrawal of certain concomitant medications. Investigators should closely monitor the need for adjustment of concomitant medications throughout the study, especially antihypertensives, as incretin-based therapies and weight reduction are commonly associated with a reduction in blood pressure.

During the study, a medication (treatment) intensity CRF pertaining to concomitant medications taken for weight-related comorbidities, including hypertension and dyslipidemia, will be collected at intervals specified in the SoA to understand the investigator's assessment of changes in treatment intensity for these conditions, for example, changes in dosage or number of medications administered; however, any changes should also be captured in the concomitant medication CRF.

The medical monitor should be contacted if there are any questions regarding concomitant or prior therapy.

Drugs that may be affected by an increase in gastric pH should be separated from study intervention administration by at least 2 hours. Examples (not exhaustive) relevant to the study population include

- simvastatin, levothyroxine, ferrous sulfate, bisphosphonates, and
- other narrow therapeutic index substrates with potential pH-dependent solubility or stability (further examples provided in Section 10.7.2.1).

Initial doses of study intervention may delay gastric emptying and have the potential to transiently increase the rate of absorption of concomitantly administered oral medicinal products. In participants receiving oral medicinal products that have rapid GI absorption, the dose of study intervention should be separated by 2 to 4 hours.

6.9.1. Symptomatic Medication for Gastrointestinal Symptoms

The investigator may prescribe symptomatic medication for management of GI symptoms as needed during the study; for example, antiemetic or antidiarrheal medication, per local country availability and standard of care (refer Section 6.6.1). Record the use of symptomatic medication as concomitant medication in the CRF.

6.9.2. Initiation of Antihyperglycemic Medications

Participants who develop diabetes (Section 10.8) during the study may initiate medication for glucose control, with the exception of DPP-4 inhibitors or GLP-1 receptor agonists or other incretin-based therapies, for example, tirzepatide (Section 10.7.1.3). Initiation of metformin or SGLT-2i for the treatment of diabetes is permitted but should not be initiated for the treatment of other conditions during the study, for example, metformin for polycystic ovary syndrome or diabetes prevention; SGLT-2i for heart failure.

6.9.3. Prohibited or Restricted Use Medications

The following medications are prohibited throughout the study:

- GLP-1 receptor agonists or other incretin-based therapies (for example, tirzepatide, semaglutide), and DPP-4 inhibitors (Section 10.7.1.3).
- Those intended to promote weight management, including prescribed, over-the-counter, or alternative remedies. Examples are provided in Section 10.7.1.1).
- Strong CYP3A inducers or inhibitors, drugs that are sensitive P-gp/BCRP substrates with a narrow therapeutic index, or strong inhibitors of OATPs. Examples of these classifications of medications are provided in Section 10.7.1.4. Note that in some

circumstances a strong CYP3A inhibitor may be used for a short duration of time (for example, for the treatment of a viral infection), if necessary. If this need arises, participants should undergo a temporary study intervention interruption and not re-initiate study intervention until at least 14 days after the end date of the strong CYP3A inhibitor. Refer to Section 7.1.2 for guidance.

The following medications are restricted (strongly discouraged) for initiation during the study, and alternative medications should be considered whenever possible:

- Moderate CYP3A inducers or inhibitors. Examples are provided in Section 10.7.2.1.
- Weight gain medications. Examples are provided in Section 10.7.1.2.

6.9.4. Prohibited Surgical Treatments or Procedures for Weight Management

Any planned elective major surgery during the study should be discussed with the sponsor's designated medical monitor.

Surgical treatments, endoscopic therapy, and/or device-based therapy for weight management are not permitted during the study.

6.9.5. Rescue Medicine

Starting at Week 24, if a participant receiving study intervention exceeds the threshold of weight regain ≥50% from the weight reduction achieved between Week 0 from SURMOUNT-5 study and Week 0 of this study, the participant should start orforglipron or increase the dose of orforglipron depending on the randomization assignment. The BW measurement used to determine rescue treatment eligibility must be obtained from in-clinic fasting visit measurement.

The study site will dispense the rescue orforglipron that will be provided by the sponsor. Participants who receive rescue orforglipron will come in monthly to complete the dose titration in a blinded fashion. De-escalation is not allowed during the use of rescue orforglipron and for any GI AE or temporary dose interruptions, refer Section 7.1.2.

Condition for rescue treatment

The criteria for rescue treatment will be based on the degree of weight regain determined based on the BW measurement obtained during in-clinic visits in this study. The need for rescue will be assessed by the investigator based on the protocol criteria. When the rescue criteria is met, orforglipron for rescue will be dispensed via IWRS in a blinded fashion.

**If a participant is randomized to the following arm and has had weight regain ≥50% at/after Week 24 while receiving study intervention …**	**Then …**
Placebo	start orforglipron at 1 mg QD and escalate every 4 weeks to MTD per IWRS
orforglipron 36 mg or MTD (24 mg or 36 mg)	escalate to 36 mg QD. If already at that dose, continue 36 mg QD

Abbreviations: MTD = maximum tolerated dose; QD= once daily.

Dose Escalation for Rescue Treatment

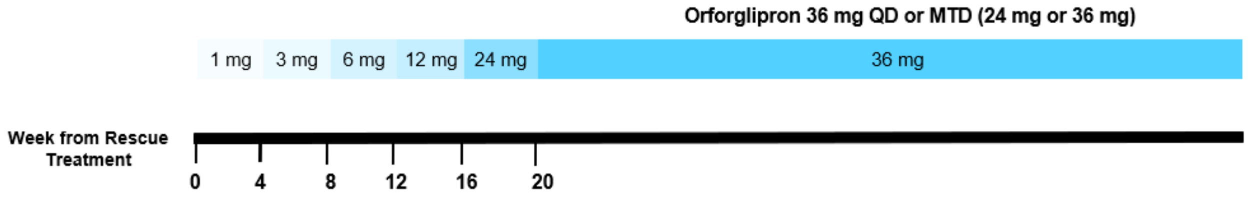

Abbreviations: MTD = maximum tolerated dose; QD = once daily.

Note: "**Week 0**" can occur at any time at or after Week 24 the participant reaches the threshold requiring rescue (that is, 50% weight regain).

Prohibited rescue treatments

No other AOMs besides orforglipron are allowed as rescue therapy in this study.

7. Discontinuation of Study Intervention and Participant Discontinuation/Withdrawal

Discontinuation of specific sites or of the study as a whole are handled as part of Section 10.1.

7.1. Discontinuation of Study Intervention

If a participant permanently discontinues study intervention for any reason, except pregnancy (Section 7.2), the participant should be encouraged to remain in the study and adhere to the study schedule until the final visit. If the participant is unwilling or unable to return for all applicable study visits, the site should attempt to collect as much follow-up information as possible, especially data collection pertaining to primary and key secondary efficacy endpoints at the final visit of the treatment period

A participant should be permanently discontinued from study intervention if

- the participant becomes pregnant during the study
- the participant is diagnosed with acute or chronic pancreatitis confirmed by adjudication. Refer Section 8.3.3.7
- the participant is diagnosed with MTC or MEN2 syndrome
- the participant develops significant elevation of serum calcitonin. Refer Section 8.3.3.8
- the participant is diagnosed with an active or untreated malignancy (other than basal or squamous cell skin cancer, in situ carcinomas of the cervix, or in situ prostate cancer [for example, Gleason 6 or lower])
- the participant is diagnosed with T1D
- the participant initiates any other GLP-1 receptor agonist, GIP/GLP-1 receptor agonist, or DPP-4 inhibitor, if the participant will not or cannot discontinue them
- if an investigator, site personnel performing assessments, or participant is unblinded
- the participant requests to discontinue study intervention
- the participant develops any other TEAE, SAE, or clinically significant laboratory value for which the investigator believes that permanent study intervention discontinuation is the appropriate measure to be taken

Other possible reasons which may lead to permanent discontinuation of study intervention:

- the participant has intolerable GI symptoms despite management as described in Section 6.6.1.
- BMI ≤18.5 kg/m^2 is reached at any time during the treatment period

  Note: The investigator should contact the sponsor’s designated medical monitor to discuss whether it is medically appropriate for the participant to continue study intervention.
- initiation of other weight management medications (Section 10.7.1.1) or if the participant has bariatric surgery or BW reduction procedures
- systemic hypersensitivity reaction (Section 8.2.7)

    - If the investigator determines that a systemic hypersensitivity reaction has occurred related to study intervention administration, the participant may be permanently discontinued from the study intervention, and the sponsor's designated medical monitor should be notified. If the investigator is uncertain about whether a systemic hypersensitivity reaction has occurred and whether discontinuation of study intervention is warranted, the investigator may consult the sponsor.
- PHQ-9 score ≥15
- C-SSRS
    - answered "yes" to Question 4 or Question 5 on the "Suicidal Ideation" portion of the C-SSRS, **or**
    - answered "yes" to any of the suicide-related behaviors on the "Suicidal Behavior" portion of the C-SSRS.

  A psychiatrist or appropriately trained professional may assist in the decision to discontinue the participant.

7.1.1. Hepatic Criteria for Study Intervention Interruption or Discontinuation

Refer to Section 8.2.6.3 for hepatic criteria for study intervention interruption or discontinuation.

7.1.2. Temporary Discontinuation

All efforts should be made to keep participants on study intervention at the randomized dose level and with minimal dose interruptions throughout the study.

Due to the short half-life of orforglipron, for any dose interruptions >2 days it is recommended that the investigator consult with the sponsor's medical monitor. Every effort should be made by the investigator to restart study intervention after any temporary interruption as soon as it is safe to do so, according to the guidance provided in the table below. Distribution of study intervention at the correct dose will be per IWRS instructions.

**If study intervention interruption is…**	**then…**
2 consecutive doses or less	participant resumes the study intervention at the last administered dose level. If the dose interruption occurred immediately prior to a scheduled dose escalation visit, the participant should proceed with the next escalation.
3-6 consecutive doses	participant resumes the study intervention at the last administered dose level, unless doing so results in intolerable GI symptoms. Refer Section 6.6.1 for management of GI symptoms. If the dose interruption occurred immediately prior to a scheduled dose escalation visit, the participant should proceed with the next escalation.

If study intervention interruption is…	then…
7 or more consecutive doses	participant repeats a dose escalation, per Section 6.9.5, starting from 3 mg/placebo to previously attained dose level, unless the interruption occurs within the first 4 weeks, in which case the last administered dose level should be resumed.<br><br>**Note** – Participants that restart at 3 mg are allowed to follow the Section 6.9.5 escalation schedule and dose modification rules to reach orforglipron doses of 24 or 36 mg and remain in study on treatment.
due to an AE (**including recurrent GI symptoms**), a clinically significant laboratory value, or a participant's personal circumstances[a]	the event is to be documented and followed according to the procedures in Section 8.3.
due to intolerable persistent GI AE	participant should be treated as suggested in Section 6.6.1.

a Travel, hospitalizations, or planned or unplanned procedures.

The data related to temporary interruption of study intervention will be documented in source documents and entered on the CRF.

7.2. Participant Discontinuation/Withdrawal from the Study

Discontinuation is expected to be uncommon. To minimize the amount of missing data and to enable assessment of study objectives as planned in the study protocol, every attempt will be made to keep participants in the study regardless of study intervention use.

Participants will be discontinued from the study if the participant becomes pregnant.

A participant may withdraw from the study

- at any time at the participant's own request for any reason or without providing any reason
- at the request of the participant's designee, for example, parents or legal guardian
- at the discretion of the investigator for safety, behavioral, compliance, or administrative reasons, or
- if enrolled in any other clinical study involving an investigational product, or enrolled in any other type of medical research judged not to be scientifically or medically compatible with this study.

At the time of discontinuing from the study, if possible, the participant will complete procedures for an ED visit and safety follow-up, as shown in the SoA. If the participant has not already

discontinued the study intervention, the participant will be permanently discontinued from the study intervention at the time of the decision to discontinue the study.

If the participant withdraws consent for disclosure of future information, the sponsor may retain and continue to use any data collected before such a withdrawal of consent. If a participant withdraws from the study, the participant may request destruction of any samples taken and not tested, and the investigator must document this in the site study records.

7.3. Lost to Follow-up

A participant will be considered lost to follow-up if they repeatedly fail to return for scheduled visits and are unable to be contacted by the study site. Site personnel or designee are expected to make diligent attempts to contact participants who fail to return for a scheduled visit or were otherwise unable to be followed up by the site.

8. Study Assessments and Procedures

Study procedures and their timing are summarized in the SoA.

Immediate safety concerns should be discussed with the sponsor immediately upon occurrence or awareness to determine if the participant should continue or discontinue study intervention.

Adherence to the study design requirements, including those specified in the SoA, is essential and required for study conduct.

All screening evaluations must be completed and reviewed to confirm that potential participants meet all eligibility criteria. The investigator will maintain a screening log to record details of all participants screened and to confirm eligibility or record reasons for screening failure, as applicable.

8.1. Efficacy Assessments

Refer Section 3 for specific efficacy endpoints.

Patient-reported outcome measures are described in Section 8.1.4.

Safety-related measures are described in Sections 8.2 and 8.3.

The independent CEC adjudicating events is described in Section 10.1.5.

8.1.1. Primary Efficacy Assessments

The primary efficacy measure is percent maintenance of BW reduction achieved during the 72-week treatment period with tirzepatide or semaglutide from SURMOUNT-5 study.

BW measurements will be collected at specific clinic visits as summarized in the SoA. Methods for measuring BW are described in Section 10.6.

8.1.2. Secondary Efficacy Assessments

The key secondary assessments include:

In all participants

- the percent change in BW from SURMOUNT-5 baseline prior to the initiation of treatment with tirzepatide or semaglutide, and
- percent maintenance of BW reduction achieved during the 72 weeks of tirzepatide or semaglutide treatment

In participants who have reached a BW plateau

- assessment (yes/no) of maintaining ≥80% of the BW reduction achieved during 72 weeks of tirzepatide or semaglutide treatment.

Additional secondary assessments include assessments of maintaining BW reduction, change in BW, percent change in BW, change in waist circumference and percent maintenance of BW reduction achieved during the 72-week treatment period with tirzepatide or semaglutide from SURMOUNT-5 study.

8.1.3. Exploratory Efficacy Assessments

The following exploratory efficacy measures will be collected at the times shown in the SoA:

- Body weight (kg) (refer Section 10.6)
- Waist circumference (refer Section 10.6)
- Lipid parameters (refer Section 3)
- Blood pressure (refer Section 3)
- Glycemic control (refer Section 3)
- Patient-reported outcomes (refer Section 8.1.4)

8.1.4. Patient-Reported Outcomes

8.1.4.1. Short Form-36 Version 2 Health Survey acute form (SF-36 v2, Acute)

The SF-36v2 will be included to assess health-related quality of life. The SF-36v2 acute form, 1-week recall version is a 36-item generic, participant-completed measure designed to assess the following 8 domains:

- physical functioning
- role-physical
- bodily pain
- general health
- vitality
- social functioning
- role-emotional, and
- mental health.

The physical functioning domain assesses limitations due to health "now" while the remaining domains assess functioning "in the past week". Each domain is scored individually and information from these 8 domains is further aggregated into 2 health component summary scores: Physical Component Summary and Mental Component Summary. Items are answered on Likert scales of varying lengths (3-point, 5-point, or 6-point scales). Scoring of each domain and both summary scores are norm based and presented in the form of T-scores, with a mean of 50 and SD of 10; higher scores indicate better levels of function and/or better health (Maruish 2011).

8.1.4.2. Control of Eating Questionnaire

The CoEQ (Dalton et al. 2015) is a 19-item, participant-completed questionnaire that assesses the intensity of food cravings, food types craved, appetite, and mood over the past 7 days. The 11-point numeric rating scale ranges from 0 (not at all) to 10 (extremely) for each of the 19 items.

Seventeen of the 19 items are grouped into the 4 domains of

- craving control
- positive mood
- craving for savory foods, and
- craving for sweet foods.

The remaining 2 items, which assess the degree of hunger and fullness, are scored individually.

Higher scores represent higher levels of the concept measured in each domain or individual item (for the 2 standalone items).

8.1.4.3. Patient Global Impression of Severity for Physical Function Weight (PGIS-Physical Function Weight)

The PGIS-Physical Function due to Weight scale is designed to assess the participants' overall perception of their condition. This is a single global item that asks participants to rate how their weight limited their ability to perform physical activities in the past 7 days on a 5-point scale ranging from "not at all limited" to "extremely limited".

8.1.4.4. Patient Global Impression of Severity for Food Craving (PGIS-Food Craving)

The PGIS Food Craving item is a patient-reported, single-item measure that assesses participants' overall perception of their food craving in the past 7 days on a 5-point scale:

- much higher than usual
- somewhat higher than usual
- same as usual
- somewhat lower than usual, and
- much lower than usual.

8.1.4.5. Patient Global Impression of Change for Physical Function Weight (PGIC-Physical Function Weight)

The PGIC-Physical Function due to Weight scale is designed to assess the participants' overall perception of the efficacy of treatment. This is a single global item that asks participants to rate the overall change in their ability to perform physical activities due to their weight since starting the study medication. The responses are based on a 5-point scale ranging from "much better" to "much worse".

8.1.4.6. Patient Global Impression of Change for Food Craving (PGIC-Food Craving)

PGIC Food Craving item is a patient-reported, single-item measure that assesses participants' change in their food craving since they started taking the study medication. The item is rated on a 5-point scale:

- increased a lot
- increased a little
- no change
- decreased a little, and
- decreased a lot.

8.1.4.7. Rapid Prime Diet Quality Score (rPDQS)

The rPDQS is a brief diet quality screener that identifies clinically relevant patterns of food intake. The rPDQS starts with the following sentence: "Thinking back over the past month, how often have you eaten each of the following foods?" This question is followed by 6 food group

items with protective health associations (for example, fish, whole grains, beans, vegetables, fruits, and peanut butter/nuts) and 7 food group items with adverse health associations (for example, processed meats, beef/pork/lamb, full-fat dairy, fast food, sugary beverages, white bread, pasta, sweets, and desserts). The responses to these 13 items are included in a dietary score.

The rPDQS questions are ordered with

1. protein sources (for example, meat, fish, and dairy),
2. carbohydrate sources (for example, fast food, sugary drinks, breads, sweets, fruits, and vegetables), and
3. fat sources (for example, nuts).

For each question, respondents select from 5 possible answers ranging from “less than once per week” to “twice per day or more”. The scoring approach allocates 0 to 4 points for consumption of each of the 6 food groups whose intake is encouraged (with a reverse scoring for the 7 food groups to limit) based on the original frequency of consumption (range, 0 to 52; higher score being healthier). The total rPDQS strongly correlated with the Healthy Eating Index (HEI)- 2015 diet quality metric estimated from 24-hour diet recalls, suggesting that the screener provides an informative assessment of an individual’s dietary intake (Kronsteiner-Gicevic et al. 2023).

8.2. Safety Assessments

Planned time points for all safety assessments are provided in the SoA.

8.2.1. Physical Examinations

A complete physical examination will include, at a minimum, assessments of the CV, respiratory, GI, and neurological systems.

Height, weight, waist and hip circumference and vital signs will also be measured and recorded. Refer Section 10.6 for further details.

Investigators should pay special attention to clinical signs related to previous serious illnesses.

8.2.2. Vital Signs

For each participant, vital sign measurements should be conducted according to Section 1.3. If warranted, additional vital signs may be measured.

Vital sign measurements (blood pressure and heart rate)

- measure vital signs before obtaining an ECG tracing and before collection of blood samples for laboratory testing
- have the participant sit quietly for about 5 minutes before vital signs measurements are taken
- for each parameter, take 3 measurements from the same arm, preferably the nondominant arm
- measure the recordings at least 1 minute apart
- BP must be taken with an automated BP instrument

- heart rate is measured by pulse

  Note: In the event pulse measurement cannot be taken via an automated BP instrument, the preferred location for measurement of pulse is the radial artery.
- if BP and pulse measurements are taken separately, pulse should be taken prior to BP.

Each measurement of sitting pulse and BP needs to be recorded in the CRF.

8.2.3. Electrocardiograms

Single 12-lead ECG will be obtained as outlined in the SoA (refer Section 1.3).

ECGs must be recorded before collecting any blood samples. Participants must be supine for at least 5 to 10 minutes before ECG collection, and remain supine and awake, during ECG collection. Electrocardiograms may be obtained at additional times, when deemed clinically necessary. All ECGs recorded should be stored at the investigational site.

ECGs will be interpreted by the investigator (a physician or qualified designee) at the site as soon after the time of ECG collection as practical, to determine whether the participant meets entry criteria or for immediate participant management, should any clinically relevant findings be identified.

If a clinically significant finding is identified after enrollment, the investigator will determine if the participant can continue in the study. The investigator, or qualified designee, is responsible for determining if any change in participant management is needed and must document their review of the ECG printed at the time of collection. Any new clinically relevant finding that occur after the participant receives the first dose of study intervention should be reported as an AE via the CRF.

8.2.4. Clinical Safety Laboratory Tests

Refer Section 10.2 for the list of clinical laboratory tests to be performed and the SoA for the timing and frequency.

The investigator must review the laboratory results, document this review, and report any clinically relevant changes occurring during the study as an AE. The laboratory results must be retained with source documents unless a Source Document Agreement or comparable document cites an electronic location that accommodates the expected retention duration. Clinically significant abnormal laboratory findings are those which are not associated with the underlying disease, unless judged by the investigator to be more severe than expected for the participant’s condition.

All laboratory tests with values considered clinically significantly abnormal during participation in the study after the last dose of study intervention should be repeated until the values return to normal or baseline or are no longer considered clinically significant by the investigator or medical monitor.

If such values do not return to normal/baseline within a period of time judged reasonable by the investigator, the etiology should be identified and the sponsor notified.

All protocol-required laboratory assessments, as defined in Section 10.2, must be conducted in accordance with the SoA, standard collection requirements, and laboratory manual.

If laboratory values from non-protocol specified laboratory assessments performed at an investigator-designated local laboratory require a change in participant management or are considered clinically significant by the investigator, for example, SAE or AE or dose modification, then report the information as an AE.

8.2.5. Pregnancy Testing

On-site pregnancy testing will occur as outlined in the SoA.

Individuals of childbearing potential will be supplied with home testing kits to perform additional pregnancy tests at any time during the study. Participants should notify the investigator as soon as possible if they test positive for pregnancy.

Participants who become pregnant during the study should be permanently discontinued from study intervention (Section 7.1) and from the study (Section 7.2).

Details of all pregnancies in AFAB participants and, if indicated, pregnancy in partners of participants will be collected as outlined in Sections 8.3.1 and 8.3.2.

8.2.6. Hepatic Safety Monitoring

The following tables summarize actions to take based on abnormal hepatic laboratory or clinical changes.

Participants with normal or near normal baseline (ALT, AST, or ALP <1.5x ULN)

If this laboratory value is observed…	Then…		
	Initiate or continue close hepatic monitoring	Initiate comprehensive evaluation	Interrupt or discontinue study intervention
ALT or AST ≥3x ULN	X		
ALP ≥2x ULN	X		
TBL ≥2x ULN[b]	X		
ALT or AST ≥5x ULN	X	X	
ALP ≥2.5x ULN	X	X	
ALT or AST ≥3x ULN with hepatic signs or symptoms[a]	X	X	X
ALT or AST ≥5x ULN for more than 2 weeks	X	X	X
ALT or AST ≥8x ULN	X	X	X
ALT or AST ≥3x ULN and TBL ≥2x ULN[b] or INR ≥1.5	X	X	X
ALP ≥3x ULN	X	X	X
ALP ≥2.5x ULN and TBL ≥2x ULN[b]	X	X	X
ALP ≥2.5x ULN with hepatic signs or symptoms[a]	X	X	X

[a] Examples of hepatic signs or symptoms: severe fatigue, nausea, vomiting, right upper quadrant abdominal pain, fever, rash, and/or eosinophilia >5%.

[b] In participants with Gilbert's syndrome, the threshold for TBL may be higher.

Participants with elevated baseline (ALT, AST, or ALP ≥1.5x ULN)

If this laboratory value is observed…	Then…		
	Initiate or continue close hepatic monitoring	Initiate comprehensive evaluation	Interrupt or discontinue study intervention
ALT or AST ≥2x baseline	X		
ALP ≥2x baseline	X		
TBL ≥2x ULN[b]	X		
ALT or AST ≥3x baseline or ≥250 U/L (whichever occurs first)	X	X	
ALP ≥2.5x baseline	X	X	
ALT or AST ≥2x baseline or ≥250 U/L (whichever occurs first) with hepatic signs or symptoms[a]	X	X	X
ALT or AST ≥3x baseline or ≥250 U/L (whichever occurs first) for more than 2 weeks	X	X	X
ALT or AST ≥4x baseline or ≥400 U/L (whichever occurs first)	X	X	X
ALT or AST ≥2x baseline or ≥250 U/L (whichever occurs first) and TBL ≥2x ULN[b] or INR ≥1.5	X	X	X
ALP ≥3x baseline	X	X	X
ALP ≥2.5x baseline and TBL ≥2x ULN[b]	X	X	X
ALP ≥2.5x baseline with hepatic signs or symptoms[a]	X	X	X

[a] Examples of hepatic signs or symptoms: severe fatigue, nausea, vomiting, right upper quadrant abdominal pain, fever, rash, and/or eosinophilia >5%.

[b] In participants with Gilbert's syndrome, the threshold for TBL may be higher.

8.2.6.1. Close Hepatic Monitoring

If a participant develops any one of these changes, initiate close hepatic monitoring:

Participants with normal or near normal baseline (ALT, AST, or ALP<1.5x ULN)	Participants with elevated baseline (ALT, AST, or ALP≥ 1.5x ULN)
ALT or AST ≥3x ULN **or**	ALT or AST ≥2x baseline
ALP ≥2x ULN **or**	ALP ≥2x baseline
TBL ≥2x ULN[b]	TBL ≥2x ULN[b]

[b] In participants with Gilbert's syndrome, the threshold for TBL may be higher.

Close hepatic monitoring should include these actions:

- Laboratory tests (Section 10.2), including ALT, AST, ALP, TBL, D. Bil, GGT, CK, and CBC with differential, should be checked within 48 to 72 hours of the detection of elevated liver tests to confirm the abnormality and to determine if it is increasing or decreasing.
- If the abnormality persists, clinical and laboratory monitoring should continue at a frequency of 2 to 3 times weekly until levels normalize or return to approximate baseline values.

- In addition to laboratory tests, basic evaluation for possible causes of abnormal liver tests should be initiated by the investigator in consultation with the Lilly-designated medical monitor. At a minimum, this evaluation should include physical examination and a thorough medical history, including current symptoms, recent illnesses (for example, heart failure, systemic infection, hypotension, or seizures), recent travel, concomitant medications (including over-the-counter), herbal and dietary supplements, history of alcohol drinking and other substance abuse.

8.2.6.2. Comprehensive Hepatic Evaluation

If a participant develops any 1 of the following laboratory or clinical changes, initiate a comprehensive hepatic evaluation:

**Participants with normal or near normal baseline (ALT, AST, or ALP <1.5x ULN)**	**Participants with elevated baseline (ALT, AST, or ALP ≥1.5x ULN)**
ALT or AST ≥5x ULN **or**	ALT or AST ≥3x baseline or ≥250 U/L (whichever occurs first) **or**
ALP ≥2.5x ULN **or**	ALP ≥2.5x baseline **or**
ALT or AST ≥3x ULN with hepatic signs or symptoms[a] **or**	ALT or AST ≥2x baseline or ≥250 U/L (whichever occurs first) with hepatic signs or symptoms[a] **or**
ALT or AST ≥3x ULN and TBL ≥2x ULN[b] or INR ≥1.5	ALT or AST ≥2x baseline or ≥250 U/L (whichever occurs first) and TBL ≥2x ULN[b] or INR ≥1.5

a Examples of hepatic signs or symptoms: severe fatigue, nausea, vomiting, right upper quadrant abdominal pain, fever, rash, and/or eosinophilia >5%.

b In participants with Gilbert's syndrome, the threshold for TBL may be higher.

Comprehensive hepatic evaluation should include these actions:

- At a minimum, comprehensive hepatic evaluation should include physical examination and a thorough medical history, as outlined above, as well as tests for PT-INR; tests for viral hepatitis A, B, C, and E; tests for autoimmune hepatitis; and an abdominal imaging study (for example, ultrasound or CT scan).
- Based on the participant's history and initial results, further testing should be considered in consultation with the Lilly-designated medical monitor, including tests for hepatitis D virus (HDV), cytomegalovirus (CMV), Epstein-Barr virus (EBV), acetaminophen levels, acetaminophen protein adducts, urine toxicology screen, Wilson's disease, blood alcohol levels, urinary ethyl glucuronide, and blood phosphatidylethanol.
- Based on the circumstances and the investigator's assessment of the participant's clinical condition, the investigator should consider referring the participant for a hepatologist or gastroenterologist consultation, and additional tests including magnetic resonance cholangiopancreatography (MRCP), endoscopic retrograde cholangiopancreatography (ERCP), cardiac echocardiogram, or a liver biopsy.
- Clinical and laboratory monitoring should continue at a frequency of 1 to 3 times weekly until levels normalize or return to approximate baseline values.

- All the medical information and tests results related to the hepatic monitoring and comprehensive hepatic evaluation should be collected and recorded in a hepatic safety CRF.

8.2.6.3. Study Intervention Interruption or Discontinuation

If a participant develops any 1 of the following laboratory or clinical changes**, interrupt the study intervention and continue close monitoring and comprehensive hepatic evaluation** as described in Section 8.2.6.1 and 8.2.6.2.

**Participants with normal or near normal baseline (ALT, AST, or ALP <1.5x ULN)**	**Participants with elevated baseline (ALT, AST, or ALP ≥1.5x ULN)**
ALT or AST ≥3x ULN with hepatic signs or symptoms[a] **or**	ALT or AST ≥2x baseline or ≥250 U/L (whichever occurs first) with hepatic signs or symptoms[a] **or**
ALT or AST ≥5x ULN for more than 2 weeks **or**	ALT or AST ≥3x baseline or ≥250 U/L (whichever occurs first) for more than 2 weeks **or**
ALT or AST ≥8x ULN **or**	ALT or AST ≥4x baseline or ≥400 U/L (whichever occurs first) **or**
ALT or AST ≥3x ULN and TBL ≥2x ULN[b] or INR ≥1.5 **or**	ALT or AST ≥2x baseline or ≥250 U/L (whichever occurs first) and TBL ≥2x ULN[b] or INR ≥1.5 **or**
ALP ≥3x ULN **or**	ALP ≥3x baseline **or**
ALP ≥2.5x ULN and TBL ≥2x ULN[b] **or**	ALP ≥2.5x baseline and TBL ≥2x ULN[b] **or**
ALP ≥2.5x ULN with hepatic signs or symptoms[a]	ALP ≥2.5x baseline with hepatic signs or symptoms[a]

[a] Examples of hepatic signs or symptoms: severe fatigue, nausea, vomiting, right upper quadrant abdominal pain, fever, rash, and/or eosinophilia >5%.

[b] In participants with Gilbert's syndrome, the threshold for TBL may be higher.

Interruption or discontinuation of study drug should include these actions:

- While the participant is not receiving the study drug, clinical and laboratory monitoring should continue at a frequency of 1 to 3 times weekly until liver tests normalize or return to approximate baseline values.
- If the hepatic event continues past the anticipated end of the study (that is, data lock) the investigator should consult with the Lilly-designated medical monitor to determine the need for further data collection beyond the end date of the study (that is, data lock date).
- All the medical information and tests results related to the close hepatic monitoring and comprehensive hepatic evaluation should be collected and recorded in a hepatic safety CRF.
- Resumption of the study drug after interruption for a hepatic reason can be considered only in consultation with the Lilly-designated medical monitor and only if the liver test results returned to near baseline and if a self-limited non-study drug etiology is identified. Otherwise, the study intervention should be permanently discontinued.

8.2.7. Hypersensitivity Reactions

Many drugs, including oral agents and biologic agents, carry the risk of systemic hypersensitivity reactions. If such a reaction occurs, additional data should be provided to the sponsor in the designated CRFs.

Sites should have appropriately trained medical staff and appropriate medical equipment available when study participants are receiving study intervention. It is recommended that participants who experience a systemic hypersensitivity reaction be treated per national and international guidelines.

In the case of a suspected systemic hypersensitivity event, additional blood samples should be collected as described in Section 10.2.1. Laboratory results are provided to the sponsor via the central laboratory.

8.2.8. Suicidal Ideation and Behavior Risk Monitoring

Columbia-Suicide Severity Rating Scale (C-SSRS)

The C-SSRS captures the occurrence, severity, and frequency of suicidal ideation and behavior during the assessment period via a semi-structured interview by a trained rater.

For this study, the C-SSRS is adapted for the assessment of the ideation and behavior categories only. The Intensity of Ideation and Lethality of Behavior sections are removed.

Timing of collection and AE monitoring

Nonleading AE collection should occur prior to the collection of the C-SSRS.

Only report a suicide-related event discovered during collection of the C-SSRS on the AE form if it is an AE that leads to discontinuation or an SAE.

Follow standard procedures for reporting SAEs.

8.2.9. Depression Monitoring

Monitor participants receiving study intervention for depression or any other unusual changes in behavior, especially at the beginning and end of the course of treatment, or at the time of dose changes, either increases or decreases.

Instrument used for monitoring

Monitor depression using PHQ-9 instrument.

Patient Health Questionnaire-9 (PHQ-9)

The PHQ-9 (Spitzer et.al. 1999; Moriarty et.al. 2015) is a validated, participant-reported instrument that assesses the specific diagnostic symptoms that determine the presence of a clinical depressive disorder per the Diagnosis and Statistical Manual for Mental Disorders, 5th Edition (DSM-5).

The questionnaire assesses the previous 2 weeks.

The PHQ-9 assesses 9 diagnostic symptoms:

- mood
- anhedonia
- appetite change
- sleep disturbance
- psychomotor agitation or retardation

- loss of energy
- feelings of worthlessness or guilt
- diminished concentration, and
- suicidal thoughts or attempts.

Each question has 4 response options, with scores ranging from 0 to 3. Higher numbers indicate greater dysfunction.

This table describes the interpretation of results.

Interpretation of Depression	Total Score
Minimal to none	0-4
Mild	5-9
Moderate	10-14
Moderately severe	15-19
Severe	20-27

8.3. Adverse Events, Serious Adverse Events, and Product Complaints

The definitions of the following events can be found in Section 3:

- AEs
- SAEs, and
- PCs.

These events will be reported by the participant, or, when appropriate, by a caregiver, surrogate, or the participant's legally authorized representative.

The investigator and any qualified designees are responsible for detecting, documenting, and recording events that meet these definitions and remain responsible for following up events that are serious, considered related to the study intervention or study procedures, or that caused the participant to discontinue the study intervention or study (refer Section 7).

Care will be taken not to introduce bias when detecting events. Open-ended and non-leading verbal questioning of the participant is the preferred method to inquire about event occurrences.

After the initial report, the investigator is required to proactively follow each participant at subsequent visits or contacts. All SAEs and AEs of special interest and other safety topics (as defined in Section 8.3.3) will be followed until resolution, stabilization, the event is otherwise explained, or the participant is lost to follow-up (as defined in Section 7.3).

For product complaints, the investigator is responsible for ensuring that follow-up includes any supplemental investigations as indicated to elucidate the nature or causality. Further information on follow-up procedures is provided in Section 10.3.

8.3.1. Timing and Mechanism for Collecting Events

This table describes the timing, deadlines, and mechanism for collecting events.

Event	Collection Start	Collection Stop	Timing for Reporting to Sponsor or Designee	Mechanism for Reporting	Back-up Method of Reporting
**Adverse Event**					
AE	Signing of the ICF	Participation in study has ended	As soon as possible upon site awareness	AE CRF	N/A
**Serious Adverse Event**					
SAE and SAE updates – prior to start of study intervention **and** deemed reasonably possibly related to study procedures	Signing of the ICF	Start of intervention	Within 24 hours of awareness	SAE CRF	SAE paper form
SAE and SAE updates – after start of study intervention	Start of intervention	Participation in study has ended	Within 24 hours of awareness	SAE CRF	SAE paper form
SAE[a] – after participant's study participation has ended **and** the investigator becomes aware	After participant's study participation has ended	N/A	Promptly	SAE paper form	N/A
**Pregnancy**					
Pregnancy in participants and partners of participants	After the start of study intervention	At least 30 days after the last dose	Within 24 hours (see Section 8.3.2)	Pregnancy CRF	Pregnancy paper form
**Product Complaints**					
PC associated with an SAE or might have led to an SAE	Start of study intervention	End of study intervention	Within 24 hours of awareness	PC form	N/A

Event	Collection Start	Collection Stop	Timing for Reporting to Sponsor or Designee	Mechanism for Reporting	Back-up Method of Reporting
PC not associated with an SAE	Start of study intervention	End of study intervention	Within 1 business day of awareness	PC form	N/A
Updated PC information	—	—	As soon as possible upon site awareness	Originally completed PC form with all changes signed and dated by the investigator	N/A
PC (if investigator becomes aware)	Participation in study has ended	N/A	Promptly	PC form	

[a] SAEs should not be reported unless the investigator deems them to be possibly related to study treatment or study participation.

8.3.2. Collection of Pregnancy Information

8.3.2.1. Participants Who Become Pregnant

The investigator will collect pregnancy information on any participant who becomes pregnant while participating in this study. The initial information will be recorded on the appropriate form and submitted to the sponsor within 24 hours of learning of a participant's pregnancy.

The participant will be followed to determine the outcome of the pregnancy. The investigator will collect follow-up information on the participant and the neonate and the information will be forwarded to the sponsor. Generally, follow-up will not be required for longer than 6 to 8 weeks beyond the estimated delivery date. Any termination of pregnancy will be reported, regardless of gestational age, fetal status (presence or absence of anomalies) or indication for the procedure.

While pregnancy itself is not considered to be an AE or SAE, any pregnancy complication or elective termination of a pregnancy for medical reasons will be reported as an AE or SAE.

A spontaneous abortion (occurring at <20 weeks gestational age) or still birth (occurring at ≥20 weeks gestational age) is always considered to be an SAE and will be reported as such.

Any poststudy pregnancy related SAE considered reasonably related to the study intervention by the investigator will be reported to the sponsor as described in Section 8.3.1. While the investigator is not obligated to actively seek this information in former study participants, the investigator may learn of an SAE through spontaneous reporting.

Any participant who becomes pregnant while participating in the study will discontinue study intervention and be withdrawn from the study. If the participant is discontinued from the study, follow the standard discontinuation process and continue directly to the follow-up phase. The

follow-up on the pregnancy outcome should continue independent of intervention or study discontinuation.

8.3.2.2. Participants With Partners Who Become Pregnant

When to collect pregnancy information

In most circumstances, the investigator will attempt to collect pregnancy information from a participant's partner who becomes pregnant while the participant is in this study.

After learning about a pregnancy in the partner of a study participant, the investigator

- will obtain a consent to release information from the pregnant partner directly, and
- within 24 hours after obtaining this consent, will record pregnancy information on the appropriate form and submit it to the sponsor.

The partner will be followed to determine the outcome of the pregnancy. Information on the status of the mother and neonate will be forwarded to the sponsor. Generally, the follow-up will be no longer than 6 to 8 weeks after the estimated delivery date. Any termination of the pregnancy will be reported regardless of gestational age, fetal status (presence or absence of anomalies) or indication for the procedure.

When not to collect pregnancy information

It is not necessary to collect information about a pregnancy in the partner of a study participant in these circumstances

- the partner of the study participant was not exposed to the study intervention, or
- the participant did not contribute the sperm or ova that resulted in the pregnancy.

8.3.3. Adverse Events of Special Interest and Other Safety Topics

8.3.3.1. Major Adverse Cardiovascular Events

Nonfatal CV AEs and all deaths will be adjudicated by a committee of physicians external to Lilly with cardiology expertise. This committee will be blinded to treatment assignment. The nonfatal CV AEs to be adjudicated include

- myocardial infarction
- hospitalization for unstable angina
- hospitalization for heart failure
- coronary interventions, such as coronary artery bypass graft or percutaneous coronary intervention, and
- cerebrovascular events, including cerebrovascular accident (stroke) and transient ischemic attack.

Case adjudication and data entry

An independent CEC with cardiology expertise will adjudicate all suspected cases of major adverse CV events. The investigator must first report these events as an AE as described in Section 8.3.1 and then report them as an endpoint on the CRF with all required source documents provided for adjudication to the CEC (refer Section 10.1.5). Clinical event reporting begins after randomization.

8.3.3.2. Arrhythmias and Cardiac Conduction Disorders

Treatment-emergent cardiac arrhythmias and conduction disorders will be further evaluated. Participants who develop any event from these groups of disorders should undergo an ECG, which should be submitted to the central reading center. Additional diagnostic tests to determine exact diagnosis should be performed, as needed. The specific diagnosis will be recorded as an AE. Events that meet criteria for serious conditions as described in Section 10.3.2 must be reported as SAEs.

8.3.3.3. Hypotension, Orthostatic Hypotension, and Syncope

All events of hypotension or orthostatic hypotension and syncope should be evaluated and additional diagnostic tests performed as needed.

8.3.3.4. Hypoglycemia

Distribution of glucometers and study diaries

All participants who develop T2D during the study will be provided with glucometers.

Participants without diabetes may, at the investigator's discretion, be given glucometers to assist in the evaluation of reported symptoms consistent with hypoglycemia.

Participants receiving glucometers will be instructed to provide glucose readings to site personnel that meet the definition of hypoglycemia.

Participants will also be trained about the signs and symptoms of hypoglycemia and its treatment. Participants will be asked to contact site personnel if they experience any of these symptoms with or without accompanying glucose readings.

Responding to recurrent hypoglycemia in participants taking concomitant antihyperglycemic medication

If a participant develops recurrent unexplained hypoglycemia during the treatment period, the investigator should consider reducing the dose of or discontinuing any concomitant antihyperglycemic medication commonly associated with hypoglycemia, for example, sulfonylurea. Study intervention discontinuation for recurrent hypoglycemia should be considered only if these events continue despite complete discontinuation of concomitant medications.

Recording hypoglycemic episodes

Hypoglycemia may be identified by spontaneous reporting of symptoms from participants (whether confirmed or unconfirmed by simultaneous glucose values) or by BG samples collected during study visits.

All hypoglycemic episodes will be recorded as AEs. If a hypoglycemic event meets severe criteria (see definition below), it should be recorded as serious on the AE and SAE CRFs and reported to Lilly as an SAE.

To avoid duplicate reporting, all consecutive blood glucose values <70 mg/dL (3.9 mmol/L) occurring within a 1-hour period may be considered a single hypoglycemic event (Weinberg et al. 2010; Danne et al. 2013).

Hypoglycemia definitions and categories

Investigators should use the following classification of hypoglycemia. The plasma glucose values in this section refer to values determined by a laboratory or International Federation of Clinical Chemistry and Laboratory Medicine plasma-equivalent glucose meters and strips.

Level 1 hypoglycemia - Glucose <70 mg/dL (3.9 mmol/L) and ≥54 mg/dL (3.0 mmol/L)

Level 1 hypoglycemia should alert the participant to take action such as treatment with fast-acting carbohydrates. Providers should continue to counsel participants to treat hypoglycemia at this glucose alert value.

Level 2 hypoglycemia - Glucose <54 mg/dL (3.0 mmol/L)

Level 2 hypoglycemia is a glucose value of <54 mg/dL (3.0 mmol/L). This glucose threshold is clinically relevant regardless of the presence or absence of symptoms of hypoglycemia.

Level 3 hypoglycemia - Severe hypoglycemia (in adults)

A severe event characterized by altered mental and/or physical status requiring assistance for treatment of hypoglycemia. For example, participants had altered mental status, and could not assist in their own care, or were semiconscious or unconscious, or experienced coma with or without seizures, and the assistance of another person was needed to actively administer carbohydrate, glucagon, or other resuscitative actions. Glucose measurements may not be available during such an event, but neurological recovery attributable to the restoration of glucose concentration to normal is considered sufficient evidence that the event was induced by a low glucose concentration.

The determination of a hypoglycemic event as an episode of severe hypoglycemia, as defined above, is made by the investigator based on the medical need of the participant to have required assistance and is not predicated on the report of a participant simply having received assistance.

If a hypoglycemic event meets the criteria of severe hypoglycemia, the investigator must record the event as serious on the AE CRF and report it to the sponsor as an SAE.

Nocturnal hypoglycemia

Nocturnal hypoglycemia is a hypoglycemia event (including severe hypoglycemia) that **occurs at night** and presumably during sleep.

8.3.3.5. Severe Gastrointestinal Adverse Events

Orforglipron may cause severe GI AEs, such as nausea, vomiting, and diarrhea. Information about severe GI AEs, as well as antiemetic or antidiarrheal use, will be collected in the AE and

concomitant medications CRFs, respectively. For detailed information concerning the management of GI AEs, please refer to Sections 6.6.1 and 6.9.1.

8.3.3.6. Acute Renal Events

Renal safety will be assessed based on repeated renal functional assessment as well as assessment of AEs suggestive of acute renal failure or worsening of preexisting chronic renal failure. GI AEs have been reported with orforglipron, including nausea, diarrhea, and vomiting. This is consistent with other GLP-1 RA (Aroda and Ratner 2011). The events may lead to dehydration, which could cause a deterioration in renal function, including acute renal failure. Participants should be advised to notify investigators in case of severe nausea, frequent vomiting, or symptoms of dehydration.

8.3.3.7. Pancreatitis

Diagnosis of acute pancreatitis

Acute pancreatitis is an AE of interest in all studies with orforglipron, including this study. The diagnosis of acute pancreatitis requires 2 of the following 3 features (Banks and Freeman 2006; Koizumi et al. 2006):

- abdominal pain, characteristic of acute pancreatitis, that is, epigastric pain radiating to the back, often associated with nausea and vomiting
- serum pancreatic amylase and/or lipase ≥3x ULN, and
- characteristic findings of acute pancreatitis on CT scan or MRI.

If acute pancreatitis is suspected, the investigator should

- obtain appropriate laboratory tests, including pancreatic amylase and lipase
- perform imaging studies, such as abdominal CT scan with or without contrast, or abdominal MRI

  Note: Abdominal ultrasound may be used as an alternative method only if CT and MRI cannot be performed.
- evaluate for possible causes of acute pancreatitis, including alcohol use, gallstone or gall bladder disease, hypertriglyceridemia, and concomitant medications.

Discontinuation for acute pancreatitis

If acute pancreatitis is suspected by the investigator, the participant must temporarily discontinue use of the study intervention (Section 7.1.2). If acute pancreatitis is ruled out, the participant should resume study intervention based on investigator's clinical judgment.

If pancreatitis is confirmed by the adjudication committee, the study intervention must be permanently discontinued (Section 7.1), and the participant should be followed throughout the duration of the study. If the case is not confirmed, then the participant can restart the study intervention if the investigator deems as clinically appropriate as described in Section 7.1.2.

Case adjudication and data entry

An independent CEC will adjudicate all suspected cases of pancreatitis. This committee will be blinded to treatment assignment. The investigator must first report these events as an AE as described in Section 8.3.1 and then report them as an endpoint on the CRF with all required source documents provided for adjudication to the CEC (refer Section 10.1.5). Clinical event reporting begins after randomization.

Asymptomatic elevation of serum amylase and/or lipase

Serial measures of pancreatic enzymes have limited clinical value for predicting episodes of acute pancreatitis in asymptomatic participants (Nauck et al. 2017; Steinberg et al. 2017a, 2017b). Therefore, further diagnostic follow-up of cases of asymptomatic elevation of pancreatic enzymes (lipase and/or pancreatic amylase ≥3x ULN) is not mandated but may be performed based on the investigator's clinical judgment and assessment of the participant's overall clinical condition.

Cases of pancreatic hyperenzymemia with symptoms or asymptomatic cases of pancreatic hyperenzymemia that undergo additional diagnostic follow-up will be submitted for adjudication.

8.3.3.8. Thyroid Malignancies and C-Cell Hyperplasia

Participants who are diagnosed with MTC and/or MEN2 during the study will have study intervention stopped (Section 7.1) and should continue follow-up with an endocrinologist.

The assessment of thyroid safety during the trial will include reporting of any case of thyroid neoplasms (including MTC, papillary carcinoma, and others) and measurements of calcitonin. The purpose of calcitonin measurements is to assess the potential of orforglipron to affect thyroid C-cell function, which may indicate development of C-cell hyperplasia and neoplasms.

Calcitonin measurements

If an increased calcitonin value (see definitions below) is observed in a participant who has been administered a medication that is known to increase serum calcitonin, then this medication should be stopped, and calcitonin levels should be measured after an appropriate washout period.

For participants who require additional endocrine assessment because of increased calcitonin concentration as defined in this section, data from the follow-up assessment will be collected in the specific section of the CRF.

Calcitonin measurements in participants with eGFR ≥60 mL/min/1.73 m^2

A significant increase in calcitonin for participants with eGFR ≥60 mL/min/1.73 m^2 is defined below. If a participant's laboratory results meet these criteria, these clinically significant laboratory results should be recorded as an AE.

- *Serum calcitonin value ≥20 ng/L and <35 ng/L AND ≥50% increase from the screening value.* These participants will be requested to repeat the measurement within 1 month. If this repeat value is increasing (≥10% increase), the study intervention should be discontinued, and the participant should undergo additional endocrine assessment and longer-term follow-up by an endocrinologist to exclude AEs on the thyroid gland.

- *Serum calcitonin value ≥35 ng/L AND ≥50% over the screening value*. In these participants, study intervention should be discontinued, and the participant should be recommended to immediately undergo additional endocrine assessments and longer-term follow-up by an endocrinologist to exclude AEs on the thyroid gland.

Calcitonin measurement in participants with eGFR <60 mL/min/1.73 m^2

A significant increase in calcitonin for participants with eGFR <60 mL/min/1.73 m^2 is defined as a *serum calcitonin value ≥35 ng/L AND ≥50% over the screening value.* If a participant's laboratory results meet these criteria, these clinically significant laboratory results should be recorded as an AE.

In these participants, study intervention should be discontinued if the increased concentration of calcitonin is confirmed. The participant must be recommended to immediately undergo additional endocrine assessments and longer-term follow-up by an endocrinologist to exclude AEs on the thyroid gland.

8.3.3.9. Malignancies

All events of malignancy or other suspected events related to malignancy should be evaluated and additional diagnostic tests performed as needed.

8.3.3.10. Hepatic Disorders

All events of hepatic disorders or other suspected events related to hepatic disorders should be evaluated and additional diagnostic tests performed as needed. In cases of elevated liver markers, hepatic monitoring should be initiated as outlined in Section 8.2.6.

8.3.3.11. Gallbladder and Biliary Tract Disorders

All events of TE biliary colic, cholecystitis, cholelithiasis, or other suspected events related to acute gallbladder disease should be evaluated and additional diagnostic tests performed, as needed.

8.3.3.12. Hypersensitivity Reactions

Refer to Section 8.2.7.

8.3.3.13. Depression and Suicidal Ideation or Behavior Monitoring

Participants will be monitored for depression and suicidal ideation or behavior through AE collection and by using the C-SSRS and the PHQ-9 questionnaires. Scores of the questionnaires must be reviewed by the investigator at the time of each visit and appropriate actions as described in Sections 7.1 and 8.2.9 should be taken.

8.3.3.14. Abuse Potential

All events of abuse potential should be evaluated, and additional investigations performed as needed.

8.4. Pharmacokinetics

PK parameters are not evaluated in this study.

8.5. Pharmacodynamics

PD parameters are not evaluated in this study.

8.6. Genetics

Genetics are not evaluated in this study.

8.7. Biomarkers

Plasma and serum samples will be collected to enable exploratory nonpharmacogenetic biomarker research, where local regulations allow.

Biomarker research is performed on stored samples to address questions of relevance to

- drug disposition
- target engagement
- PD
- mechanism of action
- variability of participant response, including safety, and
- clinical outcomes.

Samples may be used for

- research on the drug target
- disease process
- variable response to orforglipron
- pathways associated with obesity, diabetes, and related clinical traits or complications, including nonalcoholic steatohepatitis
- mechanism of action of orforglipron, and
- research method or validating diagnostic tools or assay(s) related to obesity, diabetes, or related clinical traits or complications.

Samples will be collected according to the schedule described in the SoA.

Sample retention is described in Section 10.1.12.

8.8. Immunogenicity Assessments

Immunogenicity parameters are not evaluated in this study.

8.9. Medical Resource Utilization and Health Economics

Medical resource utilization and health economics parameters are not evaluated in this study.

9. Statistical Considerations

The SAP will be finalized prior to first unblinded data transfer, and it will include a more technical and detailed description of the statistical analyses described in this section. This section is a summary of the planned statistical analyses of the most important endpoints, including primary and key secondary endpoints.

9.1. Statistical Hypotheses

For each treatment in the SURMOUNT-5 study (tirzepatide or semaglutide), the null hypothesis corresponding to the primary objective of this study is as follows:

- **Null hypothesis ($H_{1,0\ (TZP)}$, $H_{1,0\ (SEMA)}$)**: Orforglipron 36 mg or MTD (24 mg or 36 mg) is not superior to placebo at Week 52 with respect to mean percent maintenance of BW reduction achieved during 72 weeks of treatment with tirzepatide 15 mg or MTD (10 mg or 15 mg) or semaglutide 2.4 mg or MTD (1.7 mg or 2.4 mg), respectively, in participants who have reached a BW plateau.

For each treatment in the SURMOUNT-5 study (tirzepatide or semaglutide), the null hypotheses corresponding to the key secondary objectives are as follows:

- **Null Hypothesis ($H_{2,0\ (TZP)}$, $H_{2,0\ (SEMA)}$)**: Orforglipron 36 mg or MTD (24 mg or 36 mg) is not superior to placebo at Week 52 with respect to mean percent change in BW from baseline prior to the initiation of tirzepatide or semaglutide treatment, respectively (Week 0 of SURMOUNT-5 study) in all participants.
- **Null Hypothesis ($H_{3,0\ (TZP)}$, $H_{3,0\ (SEMA)}$)**: Orforglipron 36 mg or MTD (24 mg or 36 mg) is not superior to placebo at Week 52 with respect to mean percent maintenance of BW reduction achieved during 72 weeks of treatment with tirzepatide 15 mg or MTD (10 mg or 15 mg) or semaglutide 2.4 mg or MTD (1.7 mg or 2.4 mg), respectively, in all participants.
- **Null Hypothesis ($H_{4,0\ (TZP)}$, $H_{4,0\ (SEMA)}$)**: Orforglipron 36 mg or MTD (24 mg or 36 mg) is not superior to placebo at Week 52 with respect to the percentage of participants maintaining ≥80% of the BW reduction achieved during 72 weeks of treatment with tirzepatide 15 mg or MTD (10 mg or 15 mg) or semaglutide 2.4 mg or MTD (1.7 mg or 2.4 mg), respectively, in participants who reached a BW plateau.

9.1.1. Multiplicity Adjustment

Multiplicity adjusted analyses will be performed on the primary and key secondary objectives to control the overall family-wise error rate. Each treatment group in the SURMOUNT-5 study (tirzepatide or semaglutide) will be tested separately at a 2-sided alpha level of 0.05. The graphical multiple testing procedure described in Bretz et al. (2009, 2011) will be used. This approach is a closed testing procedure; hence, it strongly controls the family-wise error rate across all hypotheses (Alosh et al. 2014).

More details of the final graphical testing scheme will be prespecified in the statistical analysis plan prior to database lock. There will be no adjustment for multiple comparisons for any other analyses outside the primary and key secondary endpoints. No multiplicity adjustment is planned between estimands, as they are intended for different purposes.

9.2. Analyses Sets

For each treatment in the SURMOUNT-5 study (tirzepatide or semaglutide), the following tables define the Populations and the Analysis Datasets for the purposes of analyses based on the estimands defined in Section 3.

Population	Description
Screened population - Tirzepatide or Semaglutide, respectively	All participants from SURMOUNT-5 study treated with tirzepatide or semaglutide, respectively, who signed informed consent for this study.
Randomized population – Tirzepatide or Semaglutide, respectively	All participants from SURMOUNT-5 study who were treated with tirzepatide or semaglutide, respectively, and who are randomly assigned (orforglipron or placebo) to a treatment arm in this study.
Modified intent-to-treat population (mITT) for Efficacy Analyses – Tirzepatide or Semaglutide	All randomly assigned participants from SURMOUNT-5 study who were treated with tirzepatide or semaglutide, respectively, who are exposed to at least 1 dose of study intervention in this study. Participants who are inadvertently enrolled will be excluded. Participants will be analyzed according to the treatment they were randomly assigned to (orforglipron or placebo) regardless of the treatment actually received.
Modified intent-to-treat population (mITT) for Safety Analyses – Tirzepatide or Semaglutide	All randomly assigned participants from SURMOUNT-5 study treated with tirzepatide or semaglutide, respectively, who are exposed to at least 1 dose of study intervention in this study. Participants will be analyzed according to the treatment they were randomly assigned to (orforglipron or placebo) regardless of the treatment actually received.

Analysis Datasets	Description
Efficacy Analysis Set (EAS) – Tirzepatide or Semaglutide, respectively: This analysis set will be used to estimate the efficacy estimand	Data obtained during Study Periods I, II, and III from the mITT Population - Tirzepatide or Semaglutide, respectively, for efficacy analyses population, excluding data after permanent discontinuation of treatment, initiation of other AOMs, GLP-1 RAs, GIP/GLP-1 RAs, DPP-4 inhibitors, or rescue orforglipron, or having bariatric surgery or other weight-loss procedures.
Full Analysis Set (FAS) – Tirzepatide or Semaglutide, respectively: This analysis set will be used to estimate the modified treatment-regimen estimand	Data obtained during Study Periods I, II, and III from the mITT Population – Tirzepatide or Semaglutide, respectively, for efficacy analyses population regardless of adherence to study treatment and regardless of initiation of other AOMs, GLP-1 RAs, GIP/GLP-1 RAs , or DPP-4 inhibitors. Data obtained after rescue orforglipron or

	having bariatric surgery or other weight-loss procedures will be excluded.
Safety Analysis Set (SS) Excluding Rescue – Tirzepatide or Semaglutide, respectively: This analysis will be used to assess the safety of study treatment	Data obtained during Study Periods I, II, and III from the mITT for Safety Analyses Population – Tirzepatide or Semaglutide, respectively, regardless of adherence to study treatment and regardless of initiation of other AOMs, GLP-1 RAs, GIP/GLP-1 RAs (excluding tirzepatide or semaglutide), or DPP-4 inhibitor. Data obtained after rescue will be excluded.
Safety Analysis Set (SS) Excluding Rescue and other AOMs – Tirzepatide or Semaglutide, respectively: This analysis will be used to assess the safety of study treatment	Data obtained during Study Periods I, II, and III from the mITT for Safety Analyses Population– Tirzepatide or Semaglutide, respectively, regardless of adherence to study treatment. Data obtained after initiation of other AOMs, GLP-1 RAs, GIP/GLP-1 RAs , DPP-4 inhibitor, or rescue orforglipron will be excluded.
Safety Analysis Set (SS) After Rescue – Tirzepatide or Semaglutide, respectively: This analysis will be used to assess the safety of study treatment after participants require rescue orforglipron	Data obtained during Study Periods I, II, and III from the mITT for Safety Analyses Population: Tirzepatide or Semaglutide, respectively, regardless of adherence to study treatment and regardless of initiation of other AOMs, GLP-1 RAs, GIP/GLP-1 RAs (excluding tirzepatide or semaglutide), or DPP-4 inhibitor after participants start rescue orforglipron.

9.3. Statistical Analyses

9.3.1. General Considerations

Statistical analysis of this study will be the responsibility of Lilly or its designee. Any change to the data analysis methods described in the protocol will require an amendment ONLY if it changes a principal feature of the protocol. Any other change to the data analysis methods described in the protocol, and the justification for making the change, will be described in the SAP or CSR. Additional exploratory analyses of the data will be conducted as deemed appropriate.

Participants who have not reached the BW plateau by the end of the 72-week treatment with tirzepatide or semaglutide from SURMOUNT-5 study may also be eligible for randomization for this study if meeting all other randomization criteria but will not be included in the primary endpoint analysis. Data from these participants will be included in secondary analyses to better understand the effect of orforglipron on BW.

All tests of treatment effects will be conducted at a 2-sided alpha level of 0.05/1-sided alpha level of 0.025, unless otherwise stated, and all confidence intervals will be given at a 2-sided (95%) level. In statistical summaries and analyses, all data will be analyzed by randomized treatment assignment and will be performed separately for each treatment in the SURMOUNT-5 study. Participants will be analyzed according to the treatment they were randomly assigned to, regardless of the treatment actually received.

For each treatment in the SURMOUNT-5 study, efficacy analyses will use the corresponding efficacy analysis set to evaluate the efficacy estimand and the corresponding full analysis set to evaluate the modified treatment-regimen estimand. Safety will be assessed using the corresponding SS Excluding Rescue. Selected safety analyses may be conducted in the corresponding SS Excluding Rescue and other AOMs and in the corresponding SS After Rescue (refer Section 9.2).

Unless specified otherwise, baseline is defined as the last non-missing measurement at or before the first dose date of this study. Baseline for SURMOUNT-5 study is defined as the last non-missing measurement recorded on or before the first dose date, unless otherwise specified.

Summary statistics for continuous measures may include sample size, mean, SD, median, minimum, and maximum.

The Kaplan-Meier method will be used for estimation of cumulative event-free survival rates over time, and Cox proportional hazards regression analysis will be used to compare hazard rates among treatments.

Summary statistics for categorical measures, including categorized continuous measures, will include sample size, frequency, and percentages. Fisher's exact test will be used to examine the treatment difference in categorical outcomes. Logistic regression may be used to examine the treatment difference in binary efficacy outcomes. The negative binomial regression model will be used for the treatment comparison of discrete count measures if deemed appropriate.

Other statistical methods may be used, as appropriate, and details will be documented in the SAP.

Handling of missing, unused, and spurious data is addressed prospectively in the overall statistical methods described in the protocol and in the SAP, where appropriate. Adjustments to the planned analyses will be described in the final CSR.

9.3.2. Primary Endpoint/Estimands Analysis

For each treatment in the SURMOUNT-5 study (tirzepatide or semaglutide), the primary endpoint for this study is percent maintenance of BW reduction achieved during the 72 weeks of treatment with tirzepatide or semaglutide, respectively, for those who have achieved a BW plateau. For each treatment in the SURMOUNT-5 (SM-5) study, this endpoint will be used to evaluate the primary objective of the study for both the modified treatment-regimen and the efficacy estimands (Section 3). This endpoint is defined as

$$100 * \frac{BW - SM5\ Baseline\ BW}{Baseline\ BW - SM5\ Baseline\ BW}$$

For each treatment in the SURMOUNT-5 study, the null hypothesis corresponding to the primary objective is specified in Section 9.1.

For each treatment in the SURMOUNT-5 study, the primary objective based on the efficacy estimand defined in Section 3 will be evaluated using the corresponding EAS dataset (Section 9.2). Missing data at Week 52 for participants who take rescue orforglipron will be imputed with their highest BW measurement taken after randomization and prior to starting rescue. No additional imputation will be performed. For each treatment in the SURMOUNT-5 study, the primary analysis model for maintenance of BW reduction over time will be an

MMRM. The response variable of MMRM will be percent maintenance of BW reduction achieved during the 72 weeks of treatment with tirzepatide or semaglutide, respectively, at Week 52 visit.

The independent variables of the analysis model are treatment group (orforglipron 36 mg or MTD [24 mg or 36 mg], and placebo), visit, treatment-by-visit interaction, and sex, and percent weight loss achieved at Week 72 of SURMOUNT-5 study (<20%, ≥20%), and baseline BW as a covariate. An unstructured covariance structure will model relationship of within-patient errors.

If the analysis fails to converge, the following variance-covariance matrices will be used (in order) until convergence is achieved:

- heterogenous Toeplitz
- heterogenous first order autoregressive
- heterogeneous compound symmetry
- Toeplitz
- first order autoregressive, and
- compound symmetry.

The Kenward-Roger approximation will be used to estimate denominator degrees of freedom.

For each treatment in the SURMOUNT-5 study, the primary efficacy analysis based on the modified treatment-regimen estimand defined in Section 3 will be conducted using the corresponding full analysis set. This assessment will analyze percent maintenance at Week 52 of BW reduction achieved during the 72 weeks of treatment with tirzepatide or semaglutide, respectively, using an analysis of covariance (ANCOVA). The ANCOVA model will include terms of treatment, sex, percent weight loss achieved at Week 72 of SURMOUNT-5 study (<20%, ≥20%), and baseline BW as a covariate.

For the purpose of the treatment-regimen estimand, missing BW values at the 52-week visit in participants who had bariatric surgery or another weight-loss procedure or took rescue orforglipron will be imputed with the highest BW measurement collected after randomization and before the participant had surgery or took rescue. Missing BW values at the 52-week visit in participants who discontinued study intervention early and did not have bariatric surgery or another weight loss procedure and did not take rescue will be imputed based on observed data in the same treatment group from participants who had their efficacy assessed after ED of study intervention after randomization. This analysis will be conducted with multiple imputations, and statistical inference over multiple imputations will be guided by the method proposed by Rubin (1987).

9.3.3. Secondary Endpoints/Estimands Analysis

The endpoints corresponding to the secondary study objective subject to type 1 error rate control are specified in Section 3 under “Key Secondary” (Controlled for type 1 error) endpoints.

The null hypotheses corresponding to the key secondary objectives can be found in Section 9.1.

The key secondary objectives will be evaluated based on the modified treatment-regimen and the efficacy estimands, similar to the primary objective.

The assessment of key secondary objectives for continuous variables will be evaluated by an MMRM for the efficacy estimand and an ANCOVA for the modified treatment regimen estimand as described in Section 9.3.2.

For the assessment of the proportion of participants maintaining ≥80% of the BW reduction achieved during 72 weeks of tirzepatide or semaglutide, respectively, a logistic regression model with treatment group and strata as fixed effects and the continuous baseline value as a covariate will be used to examine the treatment difference with missing endpoints imputed. The unconditional treatment group effect will be assessed by risk difference and relative risk using the marginal standardization method, where the treatment group-specific risk will be derived from the counterfactual risks for each participant that are predicted with the fitted logistic model (FDA 2023; Ye et al. 2023). The estimated treatment group-specific risk, risk difference, relative risk, p-value, and 95% CI will be presented.

The missing value in body weight will be imputed first and then the corresponding binary variable will be derived. The details about imputation under modified treatment regimen estimand is described in Section 9.3.2. Under the efficacy estimand, data collected after ICEs will be excluded. Then missing body weight for participants who take rescue orforglipron will be imputed with their highest BW measurement taken after randomization and prior to starting rescue. Other missing data will be imputed through multiple imputation using all nonmissing data (including data imputed for participants initiated rescue) from the same treatment group under the missing-at-random assumption.

Additional details will be provided in the SAP.

9.3.4. Exploratory Endpoint Analysis

Endpoints for exploratory objectives are described in Section 3 and will be evaluated based on the efficacy estimand. Additional details will be provided in the SAP.

9.3.5. Safety Analyses

For each treatment in the SURMOUNT-5 study, safety assessments will be conducted using the corresponding SS Before Rescue and SS After Rescue (refer Section 9.2) irrespective of adherence to study intervention and initiation of other AOMs or GLP-1 RA or GIP/GLP-1 RA or DPP-4 inhibitor, unless indicated otherwise. Only descriptive statistics (that is, no treatment comparisons) will be provided for the corresponding SS After Rescue since all participants will be taking orforglipron.

AEs will be coded from the actual term using the Medical Dictionary for Regulatory Activities and reported with preferred terms and system organ class. Selected notable AEs of interest may be reported using high-level terms or Standardized Medical Dictionary for Regulatory Activities Queries. Summary statistics will be provided for incidence of TEAEs, SAEs, and study discontinuation due to AEs, study intervention discontinuation due to AEs, or deaths from the time of first dose through the end of the study.

Counts and proportions of participants experiencing AEs will be reported for each treatment group, and Fisher's exact test will be used to compare the treatment groups.

9.3.5.1. Adverse Events of Special Interest and Other Safety Topics

This section includes areas of interest whether due to observed safety findings, potential findings based on drug class, and agreed upon consultation with regulatory agencies for the reasons previously mentioned (refer Section 8.3.3). Summaries and analyses for incidence of AESIs and other safety topics will be provided by treatment. The details of analysis of AESI and other safety topics will be provided in the SAP.

9.3.5.2. Gastrointestinal Events

Summaries and analyses for incidence and severity of nausea, vomiting, constipation, and diarrhea will be provided by treatment group.

9.3.5.3. Central Laboratory Measures, and Vital Signs

Values and change from baseline to postbaseline values of central laboratory measures and vital signs will be summarized at each scheduled visit. The analysis model to make comparisons between treatment arms relative to continuous change from baseline values assessed over time will be an MMRM, with terms: treatment, visit, treatment-by-visit interaction, stratifying factors, and baseline measurement as covariates. An unstructured covariance structure will model relationship of within-participant errors.

9.3.6. Other Analyses

Details of the subgroup analyses will be shown in the SAP. For the primary endpoint, the following subgroup variables will be considered, but not limited to

- age group: <65 years, ≥65 years
- sex: individual AFAB, individual AMAB
- baseline BMI: <35, ≥35 kg/m^2
- race: white vs black vs other
- ethnicity: hispanic vs not hispanic, and
- percent BW reduction prior to randomization (<20%, ≥20%).

9.3.6.1. Suicide Ideation and Behavior

Analysis of C-SSRS Data.

Suicide-related thoughts and behaviors will be summarized based on responses to the C-SSRS consistent with the C-SSRS Scoring and Data Analysis Guide (C-SSRS WWW).

9.3.6.2. Depression

In addition to the summary of TEAEs, suicidal ideation and behavior will be assessed by C-SSRS, and depression-related symptoms will be assessed using PHQ-9. The analysis details will be provided in the SAP.

9.4. Interim Analysis

No interim analyses are planned for this study. If an unplanned interim analysis is deemed necessary for reasons other than a safety concern, the protocol must be amended. Final analysis will be performed when all participants complete the study.

9.5. Sample Size Determination

For each treatment group in the SURMOUNT-5 study, a sample size of 150 participants with a 3:2 randomization ratio (90/60 in orforglipron and placebo, respectively) is needed to ensure that 118 participants (70/48 in orforglipron and placebo, respectively) reach BW plateau. This sample size yields approximately 90% power to detect a 10% treatment difference for the primary endpoint of percent maintenance of BW reduction achieved during the 72 weeks of treatment in SURMOUNT-5, assuming a 20% discontinuation rate, and a common standard deviation of 14% using a 2-group t-test with a 5% 2-sided significant level.

All participants completing the SURMOUNT-5 study who meet the entry criteria for this study including completing the SURMOUNT-5 study on study treatment, completing Visits 2, 17, and 20 of SURMOUNT-5 study, and achieving at least a 5% weight reduction during the 72 weeks of treatment with tirzepatide or semaglutide, will be offered to enroll in this study. Therefore, the actual number of enrolled participants may be higher than planned.

10. Supporting Documentation and Operational Considerations

10.1. Appendix 1: Regulatory, Ethical, and Study Oversight Considerations

10.1.1. Regulatory and Ethical Considerations

This study will be conducted in accordance with the protocol and with the following:

- Consensus ethical principles derived from international guidelines including the Declaration of Helsinki and CIOMS International Ethical Guidelines
- Applicable ICH GCP Guidelines
- Applicable laws and regulations.

The protocol, protocol amendments, ICF, IB, and other relevant documents, for example, advertisements, must be submitted to an IRB/IEC by the investigator and reviewed and approved by the IRB/IEC before the study is initiated.

Any amendments to the protocol will require IRB/IEC approval before implementation of changes made to the study design, except for changes necessary to eliminate an immediate hazard to study participants.

Protocols and any substantial amendments to the protocol will require health authority approval prior to initiation except for changes necessary to eliminate an immediate hazard to study participants.

The investigator will be responsible for the following

- providing written summaries of the status of the study to the IRB/IEC annually or more frequently in accordance with the requirements, policies, and procedures established by the IRB/IEC
- notifying the IRB/IEC of SAEs or other significant safety findings as required by IRB/IEC procedures
- providing oversight of study conduct for participants under their responsibility and adherence to requirements of 21 code of federal regulations , ICH guidelines, the IRB/IEC, European regulation 536/2014 for clinical studies (if applicable), and all other applicable local regulations, and
- reporting to the sponsor or designee significant issues related to participant safety, participant rights, or data integrity.

Investigator sites are compensated for participation in the study as detailed in the clinical trial agreement.

10.1.2. Financial Disclosure

Investigators and sub-investigators will provide the sponsor with sufficient, accurate financial information as requested to allow the sponsor to submit complete and accurate financial certification or disclosure statements to the appropriate regulatory authorities. Investigators are responsible for providing information on financial interests during the course of the study and for 1 year after completion of the study.

10.1.3. Informed Consent Process

The investigator or the investigator's representative will explain the nature of the study, including the risks and benefits, to the potential participant or the potential participant's legally authorized representative and answer all questions regarding the study.

Potential participants must be informed that their participation is voluntary. Participants (or their legally authorized representatives) will be required to sign a statement of informed consent that meets the requirements of 21 CFR 50, local regulations, ICH guidelines, privacy and data protection requirements, where applicable, and the IRB/IEC or study center.

The medical record must include a statement that written informed consent was obtained before the participant was entered in the study and the date the written consent was obtained. The authorized person obtaining the informed consent must also sign the ICF.

Revised consents must be appropriately obtained using the correct approved ICFs for applicable study participants in accordance with sponsor and ethical review board consenting guidance.

A copy of the ICF(s) must be provided to the participant or the participant's legally authorized representative and is kept on file.

10.1.4. Data Protection

Participants will be assigned a unique identifier by the sponsor to protect the participant's personal data. Any participant information, such as records, datasets or tissue samples that are transferred to the sponsor will contain the identifier only. Participant names or any information which would make the participant identifiable will not be transferred.

The participant must be informed that the participant's personal study-related data will be used by the sponsor in accordance with local data protection law. The level of disclosure must also be explained to the participant who will be required to give consent for their data to be used as described in the informed consent. This is done by the site personnel through the informed consent process.

The participant must be informed through the informed consent by the site personnel that their medical records may be examined by Clinical Quality Assurance auditors or other authorized personnel appointed by the sponsor, by appropriate IRB/IEC members, and by inspectors from regulatory authorities.

The sponsor has processes in place to ensure information security, data integrity, and data protection. These processes address management of data transfer, and prevention and management of unauthorized access, disclosure, dissemination, alteration or loss of information

or personal data. These processes include appropriate contingency plan(s) for appropriate and timely response in the event of a data security breach.

The transfer of personal data is subject to appropriate safeguards through contractual agreements and processes. The sponsor's processes are compliant with local privacy laws and relevant legislations including the General Data Protection Regulation.

10.1.5. Committees Structure

10.1.5.1. Clinical Endpoint Committee

An independent CEC with membership external to the sponsor will be responsible for event adjudication in a blinded fashion.

Prospective adjudication of major adverse CV events (including all deaths) and pancreatic AEs will be performed for this study. Sections 8.3.3.1 and 8.3.3.7 outline additional information on CV and pancreatic adjudication committees.

10.1.6. Dissemination of Clinical Study Data

Reports

The sponsor will disclose a summary of study information, including tabular study results, on publicly available websites where required by local law or regulation.

The summary of results will be posted within the time frame specified by local law or regulation. If the study remains ongoing in some countries and a statistical analysis of an incomplete dataset would result in analyses lacking scientific rigor (for example, underpowered) or compromise the integrity of the overall analyses (for example, trial not yet unblinded), the summary of results will be submitted within 1 year after the end of the study globally or as soon as available, whichever is earlier.

Data

The sponsor provides access to all individual participant data collected during the trial, after anonymization, with the exception of PK or genetic data.

Data are available to request 6 months after the indication studied has been approved in the US and EU and after primary publication acceptance, whichever is later. No expiration date of data requests is currently set once data are made available.

Access is provided after a proposal has been approved by an independent review committee identified for this purpose and after receipt of a signed data-sharing agreement.

Data and documents, including the study protocol, SAP, CSR, and blank or annotated CRFs, will be provided in a secure data-sharing environment for up to 2 years per proposal.

For details on submitting a request, see the instructions provided at www.vivli.org.

10.1.7. Data Quality Assurance

Investigator responsibilities

All participant data relating to the study will be recorded on printed or electronic CRFs unless transmitted to the sponsor or designee electronically (for example, laboratory data). The investigator is responsible for verifying that data entries are accurate and correct by physically or electronically signing the CRF.

The investigator must maintain accurate documentation (source data) that supports the information entered in the CRF. This includes laboratory tests, medical records, and clinical notes.

The investigator must review and confirm that data entries are accurate and complete throughout the duration of the study, by physically or electronically signing the CRF, as instructed by the sponsor. All completed CRFs must be signed prior to archival.

The investigator must permit study-related monitoring, audits, IRB/IEC review, and regulatory agency inspections and provide direct or remote access to source documents.

Data monitoring and management

Quality tolerance limits will be predefined to identify systematic issues that can impact participant safety and/or reliability of study results. These predefined parameters will be monitored during the study and important excursions from the quality tolerance limits and remedial actions taken will be summarized in the CSR.

Monitoring details describing strategy, for example, risk-based initiatives in operations and quality such as risk management and mitigation strategies and analytical risk-based monitoring, methods, responsibilities and requirements, including handling of noncompliance issues and monitoring techniques are provided in the Monitoring Plan.

The sponsor or designee is responsible for the data management of this study including quality checking of the data.

The sponsor assumes accountability for actions delegated to other individuals, for example, contract research organizations.

The sponsor or designee will perform monitoring to confirm that data transcribed into the CRF by authorized site personnel are accurate, complete, and verifiable from source documents; that the safety and rights of participants are being protected; and that the study is being conducted in accordance with the currently approved protocol and any other study agreements, ICH GCP, and all applicable regulatory requirements.

Records retention and audits

Records and documents, including signed ICFs, pertaining to the conduct of this study must be retained by the investigator for the time period outlined in the clinical trial agreement unless local regulations or institutional policies require a longer retention period. No records may be destroyed during the retention period without the written approval of the sponsor. No records may be transferred to another location or party without written notification to the sponsor.

In addition, the sponsor or its representatives will periodically check a sample of the participant data recorded against source documents at the study site. The study may be audited by the sponsor or its representatives, or regulatory agencies at any time. Investigators will be given notice before an audit occurs.

Data capture system

The investigator is responsible for ensuring the accuracy, completeness, legibility, and timeliness of the data reported to the sponsor.

Electronic data capture system

An EDC system will be used in this study for the collection of CRF data. The investigator maintains a separate source for the data entered by the investigator or designee into the sponsor-provided EDC system. The investigator is responsible for the identification of any data to be considered source and for the confirmation that data reported are accurate and complete by signing the CRF.

Clinical outcome assessments

The clinical outcome assessments data (participant-focused outcome instrument) and other data will be collected by the authorized study personnel, via a paper source document and will be transcribed by the authorized study personnel into the EDC system.

Additionally, when electronic Clinical Outcome Assessment (eCOA) data (participant-focused outcome instrument) is directly recorded by the participant, into an instrument (for example, hand held smart phone or tablet), the eCOA data will serve as the source documentation and the investigator does not maintain a separate written or electronic record of these data.

Data storage and access

Data collected via the sponsor-provided data capture system(s) will be stored at a third-party (at third parties). The investigator will have continuous access to the data during the study and until decommissioning of the data capture system(s). Prior to decommissioning, the investigator will receive or access an archival copy of pertinent data for retention.

Data managed by a central vendor, such as laboratory test data, will be stored electronically in the central vendor's database system and reports will be provided to the investigator for review and retention. Data will subsequently be transferred from the central vendor to the sponsor data warehouse.

Data from complaint forms submitted to the sponsor will be encoded and stored in the global product complaint management system.

10.1.8. Source Documents

Source documents provide evidence for the existence of the participant and substantiate the integrity of the data collected. Source documents are filed at the investigator's site.

Data reported on or entered in the CRF and are transcribed from source documents must be consistent with the source documents or the discrepancies must be explained. The investigator may need to request previous medical records or transfer records, depending on the study. Also, current medical records must be available.

Definition of what constitutes source data can be found in the site confirmation of source data.

10.1.9. Study and Site Start and Closure

Study start

The study start date is the date on which the clinical study will be open for recruitment of participants.

First act of recruitment

The first act of recruitment is the date on which the clinical study will be open for recruitment of participants.

Study or site termination

The sponsor or sponsor's designee reserves the right to close the study site or terminate the study at any time for any reason at the sole discretion of the sponsor. Study sites will be closed upon study completion. A study site is considered closed when all required documents and study supplies have been collected and a study site closure visit has been performed.

The investigator may initiate study site closure at any time, provided there is reasonable cause and sufficient notice is given in advance of the intended termination.

Reasons for the early closure of a study site by the sponsor or investigator may include but are not limited to

- for study termination due to discontinuation of further study intervention development
- for site termination due to
    - failure of the investigator to comply with the protocol, the requirements of the IRB/IEC or local health authorities, the sponsor's procedures, or GCP guidelines
    - inadequate recruitment, evaluated after a reasonable amount of time of participants by the investigator, or
    - total number of participants included earlier than expected.

If the study is prematurely terminated or suspended, the sponsor shall promptly inform the investigators, the IECs/IRBs, the regulatory authorities, and any contract research organization(s) used in the study of the reason for termination or suspension, as specified by the applicable regulatory requirements. The investigator shall promptly inform the participant and should assure appropriate participant therapy and/or follow-up.

10.1.10. Publication Policy

In accordance with the sponsor's publication policy, the results of this study will be submitted for publication by a peer-reviewed journal.

10.1.11. Investigator Information

Researchers with appropriate education, training, and experience, as determined by the sponsor, will participate as investigators in this clinical trial.

10.1.12. Sample Retention

Sample retention enables use of new technologies, response to regulatory questions, and investigation of variable response that may not be observed until later in the development of orforglipron or after orforglipron become(s) commercially available.

Sample Type	Custodian	Retention Period After Last Participant Visit
Exploratory biomarkers	Sponsor or designee	7 years

10.2. Appendix 2: Clinical Laboratory Tests

The tests detailed in the table below will be performed by the central laboratory.

Local laboratory results are only required in the event that the central laboratory results are not available in time for either study intervention administration and/or response evaluation. If a local sample is required, it is important that the sample for central analysis is obtained at the same time. Additionally, if the local laboratory results are used to make either a study intervention decision or response evaluation, the results must be recorded.

In circumstances where the sponsor approves local laboratory testing in lieu of central laboratory testing (in the table below), the local laboratory must be qualified in accordance with applicable local regulations.

Protocol-specific requirements for inclusion or exclusion of participants are detailed in Section 5 of the protocol.

Additional tests may be performed at any time during the study as determined necessary by the investigator or required by local regulations.

Investigators must document their review of the laboratory safety results.

Laboratory/analyte results that could unblind the study will not be reported to investigative sites or other blinded personnel until the study has been unblinded.

Clinical Laboratory Tests	Comments
**Hematology**	Assayed by Lilly-designated laboratory.
Hemoglobin	
Hematocrit	
Erythrocyte count (RBCs - red blood cells)	
Mean cell volume	
Mean cell hemoglobin	
Mean cell hemoglobin concentration	
Leukocytes (WBCs - white blood cells)	
Differential	
Absolutes Count of:	
Neutrophils	
Lymphocytes	
Monocytes	
Eosinophils	
Basophils	
Platelets	
**Clinical chemistry**	Assayed by Lilly-designated laboratory.
Sodium	
Potassium	
Chloride	
Bicarbonate	
Total bilirubin	
Direct bilirubin	

Clinical Laboratory Tests	Comments
Alkaline phosphatase (ALP)	
Alanine aminotransferase (ALT)	
Aspartate aminotransferase (AST)	
Gamma-glutamyl transferase (GGT)	
Blood urea nitrogen (BUN)	
Creatinine	
Creatine kinase (CK)	
Uric acid	
Total protein	
Albumin	
Calcium	
Phosphorus	
Glucose	
**Lipid panel**	Assayed by Lilly-designated laboratory.
Total Cholesterol	
Triglycerides	
Low-density lipoprotein cholesterol (LDL-C)	Generated by Lilly-designated laboratory.<br>Direct measurement will be performed if triglycerides exceed maximum value for calculation.
Very Low-density lipoprotein cholesterol (VLDL-C)	Generated by Lilly-designated laboratory.
High-density lipoprotein cholesterol (HDL-C)	Generated by Lilly-designated laboratory.
Non-High density lipoprotein cholesterol (non-HDL)	Generated by Lilly-designated laboratory.
**Hepatitis serology**	
Hepatitis C Virus (HCV) testing:	
HCV antibody	
HCV RNA	Performed only for participants who test positive for anti-HCV.
Hepatitis B Virus (HBV) testing:	
HBV DNA	Performed only for participants who test positive for anti-HBc.
Hepatitis B core antibody (HBcAb)	
Hepatitis B surface antigen (HBsAg)	
Hepatitis B surface antibody (anti-HBs)	
**Hormones (female)**	
Serum pregnancy	Assayed by Lilly-designated laboratory.
Urine pregnancy	Assayed and evaluated locally.
Follicle-stimulating hormone (FSH)	Assayed by Lilly-designated laboratory.
**Calculations**	Generated by Lilly-designated laboratory.
eGFR (CKD-EPI) calculated using creatinine	Results will not be provided to the investigative sites.
eGFR (CKD-EPI) calculated using cystatin C	
eGFR (CKD-EPI) calculated using creatinine and cystatin C	Results will not be provided to the investigative sites.
Urinary albumin/creatinine ratio (UACR)	

Clinical Laboratory Tests	Comments
**Additional testing**	Assayed by Lilly-designated laboratory.
HbA1c	
Insulin	Results will not be provided to the investigative sites.
C-peptide	Results will not be provided to the investigative sites.
C-reactive protein, high sensitivity (hsCRP)	Results will not be provided to investigative sites.
Cystatin C	
Calcitonin	
Pancreatic amylase	
Lipase	
**Exploratory biomarker storage samples**	Assayed by Lilly-designated laboratory.<br>Results will not be provided to the investigative sites.
Serum	
Plasma (EDTA)	

10.2.1. Laboratory Samples to be Obtained at the Time of a Systemic Hypersensitivity Event

Purpose of collecting samples after a systemic hypersensitivity event

The samples listed in this appendix are not collected for acute study participant management. The sponsor will use the laboratory tests results from these samples to characterize hypersensitivity events across the clinical development program.

When to collect samples after a systemic hypersensitivity event occurs

Collect the samples listed below if a systemic hypersensitivity event is suspected. The timing should be as designated in the table, assuming the participant has been stabilized.

Obtain follow-up predose samples at the next regularly scheduled laboratory sample collection (ideally prior to the next dose after the event) to assess post-event return-to-baseline values.

Timing	Laboratory Test [a]
Collect from 30 minutes to 4 hours after the start of the event.<br>• Note: The optimal collection time is from 1 to 2 hours after the start of event.	total tryptase

[a] All samples for hypersensitivity testing will be assayed by Lilly-designated laboratory. Results will not be provided to the study site. If samples are not collected or are collected outside the specified time period, this will not be considered a protocol deviation.

What information to record

Record the date and time when the samples are collected.

Allowed additional testing for participant management

The investigator may perform additional tests locally, if clinically indicated, for acute study participant management.

10.3. Appendix 3: Adverse Events and Serious Adverse Events: Definitions and Procedures for Recording, Evaluating, Follow-up, and Reporting

10.3.1. Definition of AE

An AE is any untoward medical occurrence in a participant administered a pharmaceutical product and which does not necessarily have a causal relationship with the study intervention. An AE can therefore be any unfavorable and unintended sign (including an abnormal laboratory finding), symptom, or disease (new or exacerbated) temporally associated with the use of a medicinal (investigational) product, or investigational combination product, whether or not related to the medicinal (investigational) product or investigational combination product.

Events meeting the AE definition

- Any abnormal laboratory test results (hematology, clinical chemistry, or urinalysis) or other safety assessments, for example, ECG, radiological scans, and vital signs measurements, including those that worsen from baseline, considered clinically significant in the medical and scientific judgment of the investigator, that is, not related to progression of underlying disease.
- Exacerbation of a chronic or intermittent preexisting condition including either an increase in frequency and/or intensity of the condition.
- New condition detected or diagnosed after study intervention administration even though it may have been present before the start of the study.
- Signs, symptoms, or the clinical sequelae of a suspected drug-drug interaction.
- Medication error, misuse, or abuse of IMP, including signs, symptoms, or clinical sequelae.
- Lack of efficacy or failure of expected pharmacological action per se will not be reported as an AE or SAE. Such instances will be captured in the efficacy assessments. However, the signs, symptoms, and/or clinical sequelae resulting from lack of efficacy will be reported as AE or SAE if they fulfill the definition of an AE or SAE.

Events NOT meeting the AE definition

- Any clinically significant abnormal laboratory findings or other abnormal safety assessments that are associated with the underlying disease, unless judged by the investigator to be more severe than expected for the participant's condition.
- The disease or disorder being studied or expected progression, signs, or symptoms of the disease or disorder being studied, unless more severe than expected for the participant's condition.
- Situations in which an untoward medical occurrence did not occur (social and/or convenience admission to a hospital).
- Anticipated day-to-day fluctuations of preexisting disease(s) or condition(s) present or detected at the start of the study that do not worsen.

10.3.2. Definition of SAE

An SAE is defined as any untoward medical occurrence that, at any dose, meets 1 or more of the criteria listed:

- Results in death
- Is life-threatening
    - The term *life-threatening* in the definition of *serious* refers to an event in which the participant was at risk of death at the time of the event. It does not refer to an event, which hypothetically might have caused death, if it were more severe.
- Requires inpatient hospitalization or prolongation of existing hospitalization
    - In general, hospitalization signifies that the participant has been admitted to hospital or emergency ward (usually involving at least an overnight stay) for observation and/or treatment that would not have been appropriate in the physician's office or outpatient setting. Complications that occur during hospitalization are AEs. If a complication prolongs hospitalization or fulfills any other serious criteria, the event is serious. When in doubt as to whether hospitalization occurred or was necessary, the AE should be considered serious.
    - Hospitalization for elective treatment of a preexisting condition that did not worsen from baseline is not considered an AE.
- Results in persistent disability/incapacity
    - The term disability means a substantial disruption of a person's ability to conduct normal life functions.
    - This definition is not intended to include experiences of relatively minor medical significance, such as uncomplicated headache, nausea, vomiting, diarrhea, influenza, and accidental trauma, for example, sprained ankle, which may interfere with or prevent everyday life functions but do not constitute a substantial disruption.
- Is a congenital anomaly/birth defect
    - Abnormal pregnancy outcomes, for example, spontaneous abortion, fetal death, stillbirth, congenital anomalies, and ectopic pregnancy, are considered SAEs.
- Other situations:
    - Medical or scientific judgment should be exercised by the investigator in deciding whether SAE reporting is appropriate in other situations such as important medical events that may not be immediately life-threatening or result in death or hospitalization but may jeopardize the participant or may require medical or surgical intervention to prevent one of the other outcomes listed in the above definition. These events should usually be considered serious.
    - Examples of such events include invasive or malignant cancers, intensive treatment in an emergency room or at home for allergic bronchospasm, blood

dyscrasias or convulsions that do not result in hospitalization, or development of drug dependency or drug abuse.

10.3.3. Definition of Product Complaints

Product complaint

A PC is any written, electronic, or oral communication that alleges deficiencies related to the identity, quality, durability, reliability, safety, effectiveness or performance of a study intervention. When the ability to use the study intervention safely is impacted, the following are also PCs:

- deficiencies in labeling information, and
- use errors for device or drug-device combination products due to ergonomic design elements of the product.

PCs related to study interventions used in clinical trials are collected to ensure the safety of participants, monitor quality, and to facilitate process and product improvements.

If the participant identifies a PC or a problem with the study intervention, investigators will instruct participants to contact the site as soon as possible so that the situation can be assessed.

An event may meet the definition of both a PC and an AE/SAE. In such cases, it should be reported as both a PC and as an AE/SAE.

Device deficiencies are product complaints.

10.3.4. Recording and Follow-Up of AE and/or SAE and Product Complaints

AE, SAE, and product complaint recording

When an AE/SAE/PC occurs, it is the responsibility of the investigator to review all documentation (for example, hospital progress notes, laboratory reports, and diagnostics reports) related to the event.

The investigator will then record all relevant AE/SAE/PC information in the participant's medical records, in accordance with the investigator's normal clinical practice. AE/SAE information is reported on the appropriate CRF page and product complaint information is reported on the Product Complaint Form.

Note: An event may meet the definition of both a PC and an AE/SAE. In such cases, it should be reported as both a PC and as an AE/SAE.

It is **not** acceptable for the investigator to send photocopies of the participant's medical records to the sponsor or designee in lieu of completion of the CRF page for AE/SAE and the Product Complaint Form for PCs.

There may be instances when copies of medical records for certain cases are requested by the sponsor or designee. In this case, all participant identifiers, with the exception of the participant number, will be redacted on the copies of the medical records before submission to the sponsor or designee.

The investigator will attempt to establish a diagnosis of the event based on signs, symptoms, and/or other clinical information. Whenever possible, the diagnosis (not the individual signs/symptoms) will be documented as the AE/SAE.

Assessment of intensity

The investigator will make an assessment of intensity for each AE and SAE reported during the study and assign it to one of the following categories:

- Mild: A type of AE that is usually transient and may require only minimal treatment or therapeutic intervention. The event does not generally interfere with usual activities of daily living.
- Moderate: A type of AE that is usually alleviated with additional specific therapeutic intervention. The event interferes with usual activities of daily living, causing discomfort but poses no significant or permanent risk of harm to the research participant.
- Severe: A type of AE that interrupts usual activities of daily living, or significantly affects clinical status, or may require intensive therapeutic intervention. An AE that is assessed as severe should not be confused with an SAE. Severe is a category utilized for rating the intensity of an event, and both AEs and SAEs can be assessed as severe.

  An event is defined as "serious" when it meets at least 1 of the predefined outcomes as described in the definition of an SAE, NOT when it is rated as severe.

Assessment of causality

The investigator is obligated to assess the relationship between study intervention and each occurrence of each AE/SAE. The investigator will use clinical judgment to determine the relationship.

A "reasonable possibility" of a relationship conveys that there are facts, evidence, and/or arguments to suggest a causal relationship, rather than a relationship cannot be ruled out.

Alternative causes, such as underlying disease(s), concomitant therapy, and other risk factors, as well as the temporal relationship of the event to study intervention administration will be considered and investigated.

The investigator will also consult the IB in their assessment.

The investigator **must** review and provide an assessment of causality for each AE/SAE and document this in the medical notes.

There may be situations in which an SAE has occurred and the investigator has minimal information to include in the initial report to the sponsor or designee. However, it is very important that the investigator always make an assessment of causality for every event before the initial transmission of the SAE data to the sponsor or designee.

The investigator may change their opinion of causality in light of follow-up information and send an SAE follow-up report with the updated causality assessment.

The causality assessment is one of the criteria used when determining regulatory reporting requirements.

Follow-up of AEs and SAEs

- The investigator is obligated to perform or arrange for the conduct of supplemental measurements and/or evaluations as medically indicated or as requested by the sponsor or designee to elucidate the nature and/or causality of the AE or SAE as fully as possible. This may include additional laboratory tests or investigations, histopathological examinations, or consultation with other health care professionals.
- If a participant dies during participation in the study or during a recognized follow-up period, the investigator will provide the sponsor or designee with a copy of any postmortem findings including histopathology.

10.3.5. Reporting of SAEs

SAE reporting via an electronic data collection tool

The primary mechanism for reporting an SAE will be the electronic data collection tool.

If the electronic system is unavailable, then the site will use the SAE paper form (see next section) to report the event within 24 hours.

The site will enter the SAE data into the electronic system as soon as it becomes available.

After the study is completed at a given site, the electronic data collection tool will be taken off-line to prevent the entry of new data or changes to existing data.

If a site receives a report of a new SAE from a study participant or receives updated data on a previously reported SAE after the electronic data collection tool has been taken off-line, then the site can report this information on an SAE paper form (see next section) or to the medical monitor by telephone.

Contacts for SAE reporting can be found in SAE form.

SAE reporting via paper form

Facsimile transmission of the SAE paper form is the preferred method to transmit this information to the medical monitor.

Initial notification via telephone does not replace the need for the investigator to complete and sign the SAE CRF pages within the designated reporting time frames.

Contacts for SAE reporting can be found in SAE form.

10.3.6. Regulatory Reporting Requirements

SAE regulatory reporting

Prompt notification by the investigator to the sponsor of a SAE is essential so that legal obligations and ethical responsibilities toward the safety of participants and the safety of a study intervention under clinical investigation are met.

The sponsor has a legal responsibility to notify both the local regulatory authority and other regulatory agencies about the safety of a study intervention under clinical investigation. The sponsor will evaluate the reported SAEs, including confirmation of relatedness and assessment

of expectedness. The sponsor will comply with country-specific regulatory requirements relating to safety reporting to the regulatory authority, IRB/ IEC, and investigators.

An investigator who receives an investigator safety report describing a SAE or other specific safety information (for example, summary or listing of SAEs) from the sponsor will review and then file it along with the IB and will notify the IRB/IEC, if appropriate according to local requirements.

10.4. Appendix 4: Contraceptive and Barrier Guidance

10.4.1. Definitions

Word/Phrase	Definition
Individuals assigned female at birth (AFAB)	Individuals assigned the female sex based on external genitalia and/or genetic or medical information. In addition, if these individuals are of reproductive potential, they are potentially capable of gestating a fetus, and thus are capable of exposing an egg, embryo, or fetus to study intervention or drug effects.
Individuals assigned male at birth (AMAB)	Individuals assigned the male sex based on external genitalia and/or genetic or medical information. In addition, if these individuals are of reproductive potential, they are not capable of gestating a fetus, but are capable of exposing a fetus to study drug or drug effects via their semen. Individuals AMAB are considered to be not of reproductive potential if they have had orchiectomy (orchidectomy) with or without penectomy, confirmed by operative note.
Individuals of childbearing potential (IOCBP) [a]	Adult individuals AFAB are considered IOCBP unless they are INOCBP.<br>Note: Adolescent or adult individuals AFAB who are receiving hormone therapy as part of gender transition are considered IOCBP unless they meet the conditions outlined below for INOCBP.<br>Individuals AFAB who underwent endometrial ablation are considered IOCBP as it is not a form of surgical sterilization or contraception.
Individuals not of childbearing potential (INOCBP) [b]	Individuals AFAB are considered INOCBP if they are not capable of producing ova or embryo, and/or are not capable of potentially gestating a fetus. Such individuals include those who<br>• have a congenital anomaly such as Müllerian agenesis, resulting in confirmed infertility<br>• are infertile due to surgical sterilization, or<br>• are menopausal.<br>Acceptable surgical sterilization methods are total hysterectomy, bilateral salpingo-oophorectomy, bilateral salpingectomy, or bilateral oophorectomy.

Word/Phrase	Definition
Menopausal state [c]	The menopausal state is defined as an individual:<br>• at any age at least 6 weeks post-surgical bilateral oophorectomy with or without hysterectomy, confirmed by operative note; or<br>• aged at least 40 years and up to 55 years with an intact uterus, not on hormone therapy[c], who has had cessation of menses for at least 12 consecutive months without an alternative medical cause, AND with a follicle-stimulating hormone ≥40 mIU/mL; or<br>• 55 years or older not on hormone therapy, who has had at least 12 months of spontaneous amenorrhea, or<br>• aged at least 55 years with a diagnosis of menopause prior to starting hormone replacement therapy.

a IOCBP is inclusive of the concept of women of childbearing potential (WOCBP or WCBP), a term often used in literature and regulatory guidance documents.

b INOCBP is inclusive of the concept of women not of childbearing potential (WNOBCP).

c The individual **should not** be taking medications during amenorrhea such as oral contraceptives, hormone replacement therapy (HRT), gonadotropin-releasing hormone, anti-estrogens, selective estrogen receptor modulators, or chemotherapy that could induce transient amenorrhea. Individuals on HRT and those whose menopausal status cannot be confirmed will be required to comply with the protocol contraception requirements if they wish to continue HRT during the study. Otherwise they must discontinue HRT to allow confirmation of menopausal status before study enrollment.

10.4.2. Contraception Guidance

Individuals AFAB

IOCBP who are completely abstinent as their preferred and usual lifestyle, or exclusively engage in sexual relations with other individual(s) who are AFAB as their preferred and usual lifestyle, must follow the rules in this table.

Must…	Must not…
agree to either remain abstinent or exclusively engage in sexual relations with other individual(s) who are AFAB, and not plan a pregnancy during the study	• use periodic abstinence methods<br>○ calendar<br>○ ovulation<br>○ symptothermal, or<br>○ post-ovulation<br>• declare abstinence just for the duration of a trial, or<br>• use the withdrawal method

IOCBP who are NOT completely abstinent as their preferred and usual lifestyle, or who do NOT exclusively engage in sexual relations with other individual(s) who are AFAB as their preferred and usual lifestyle, must follow the rules in this table.

**Must…**
Agree to use 2 methods of effective contraception, where at least 1 method must be highly effective. These methods of contraception must be used during the study and for at least 30 days after the last dose of the study intervention.<br>Note: Individuals AFAB who had bilateral tubal ligation should use second form of contraception.

Examples of different forms of contraception:

**Methods**	**Examples**
Highly effective contraception (less than 1% failure rate)	• fallopian tubal sterilization methods other than bilateral salpingectomy (laparoscopic bipolar electrocoagulation, plastic ring application on the uterine tubes, fallopian tube ligation, hysteroscopic sterilization) Note: Bilateral salpingectomy is indicative of permanent sterilization. Please see the INOCBP definition above<br>• combination oral contraceptive pill<br>• progestin-only contraceptive pill (mini-pill)<br>• implanted contraceptives<br>• injected contraceptives<br>• contraceptive patch (only for individuals <198 pounds or 90 kg)<br>• total abstinence<br>• sexual relationships exclusively between individuals who are assigned the same sex at birth<br>• vasectomy – for individuals AMAB in clinical trials and for the partner of an individual AFAB (if only sexual partner)<br>• fallopian tube implants (if confirmed by hysterosalpingogram)<br>• vaginal ring containing combination hormone medication, or<br>• intrauterine devices
Effective contraception	• penile condom with spermicide<br>• vaginal condom with spermicide<br>• diaphragm with spermicide<br>• cervical sponge with spermicide, or<br>• cervical cap with spermicide<br>Note: Penile and vaginal condoms should not be used in combination.
Ineffective forms of contraception whether	• spermicide alone<br>• periodic abstinence

Methods	Examples
used alone or in any combination	• fertility awareness (calendar method, temperature method, cervical mucus, or symptothermal)<br>• withdrawal<br>• postcoital douche, or<br>• lactational amenorrhea

Individuals AMAB

Individuals AMAB may participate in this trial.

For individuals AMAB, no contraception is required except in compliance with specific local government study requirements.

10.5. Appendix 5: Liver Safety: Suggested Actions and Follow-up Assessments

Hepatic evaluation testing

Refer Section 8.2.6 for guidance on appropriate test selection.

The Lilly-designated central laboratory should complete the analysis of all selected testing except for testing listed in the investigator-designated local laboratory table. The central laboratory will report results if a validated test or calculation is available.

Local testing may be performed *in addition to central testing* when necessary for immediate participant management. The local laboratory must be qualified in accordance with applicable local regulations. If testing is not available in certain regions based on local requirements, consult with Lilly-designated medical monitor.

**Tests assayed by Lilly-designated central laboratory**	
**Hepatic Hematology Panel**	**Hepatitis A virus (HAV) testing:**
Hemoglobin	HAV total antibody [a]
Hematocrit	HAV IgM antibody
Erythrocytes (RBCs - red blood cells)	**Hepatitis B virus (HBV) testing:**
Leukocytes (WBCs - white blood cells)	Hepatitis B surface antigen (HBsAg)
Differential:	Hepatitis B surface antibody (anti-HBs)
Neutrophils	Hepatitis B core total antibody (anti-HBc)
Lymphocytes	Hepatitis B core IgM antibody
Monocytes	HBV DNA [b]
Basophils	**Hepatitis C virus (HCV) testing:**
Eosinophils	HCV total antibody [a]
Platelets	HCV RNA [b]
Cell morphology (RBC and WBC)	**Hepatitis D virus (HDV) testing [c]:**
**Hepatic Clinical Chemistry Panel**	HDV total antibody [a]
Total bilirubin	HDV IgM antibody
Direct bilirubin	HDV RNA [b]
Alkaline phosphatase (ALP)	**Hepatitis E virus (HEV) testing:**
Alanine aminotransferase (ALT)	HEV IgG antibody
Aspartate aminotransferase (AST)	HEV IgM antibody
Gamma-glutamyl transferase (GGT)	HEV RNA [b]
Creatine kinase (CK)	**Anti-nuclear antibody (ANA)**
**Hepatic Coagulation Panel**	**Anti-smooth muscle antibody (ASMA) or anti-actin antibody**
Prothrombin time, INR (PT-INR)	**Immunoglobulin IgA (quantitative)**
**Urine Chemistry**	**Immunoglobulin IgG (quantitative)**
Drug screen	**Immunoglobulin IgM (quantitative)**
**Haptoglobin**	

Tests assayed by investigator-designated local laboratory	
**Acetaminophen**	**Cytomegalovirus (CMV) testing:**
**Acetaminophen protein adducts** [d]	CMV antibody
**Alkaline phosphatase isoenzymes**	CMV DNA [b]
**Ceruloplasmin**	**Herpes simplex virus (HSV) testing:**
**Copper**	HSV (Type 1 and 2) antibody
**Ethyl alcohol (ethanol, EtOH)**	HSV (Type 1 and 2) DNA [b]
**Phosphatidylethanol (PEth)**	Liver kidney microsomal type 1 (LKM-1) antibody
**Urine Chemistry**	**Microbiology Culture:**
Ethyl glucuronide (EtG)	Blood
**Epstein-Barr virus (EBV) testing:**	Urine
EBV antibody	
EBV DNA [b]	

a If laboratory does not offer total antibody testing, IgG and/ or IgM are acceptable substitutes.
b Reflex/confirmation dependent on regulatory requirements, testing availability, or both.
c If HDV testing is not available, HBV testing may be sufficient. If HBV testing is positive, consult with the Lilly-designated medical monitor.
d Availability of acetaminophen protein adducts testing is limited, so testing may be performed at central laboratories, if needed.

10.6. Appendix 6: Measurement of Height, Weight, Waist, and Hip Circumference

The following information has been adapted from standardized physical measurement protocols for the WHO's STEPwise approach to Surveillance (STEPS) (WHO 2017).

Height

Step 1. Ask the participant to remove their footwear and any headgear (light headgear worn for religious reasons can remain, but this should be worn by the participant at every clinic visit when their height is measured).

Step 2. Ask the participant to stand on the calibrated height measuring board (stadiometer) or against a wall with their feet together and their knees straight with their heels against the backboard, the stadiometer, or the wall.

Step 3. Ask the participant to look straight ahead without tilting their head up.

Step 4. Ask the participant to breathe in and stand tall. Measure and record the participant's height in **centimeters to 1 decimal place**.

Weight

- Body weight measurements should be done in a consistent manner using a calibrated electronic scale capable of measuring weight in **kilograms to 1 decimal place**.
- All weights for a given participant should be measured using the same scale, whenever possible, at approximately the same time in the morning after evacuation of bladder contents.
- Body weight must be measured in fasting state. If the participant is not fasting, the participant should be called in for a new visit within the visit window to have the fasting body weight measured.

Step 1. Ask the participant to empty their pockets, remove their footwear, outerwear (coat, jacket, etc.), and any headgear (light headgear worn for religious reasons can remain, but this should be worn by the participant at every clinic visit when weight is measured).

Step 2. Make sure the scale is placed on a firm, flat, even surface (not on carpet, on a sloping surface, or a rough, uneven surface).

Step 3. Ask the participant to step onto the scale with 1 foot on each side of the scale.

Step 4. Ask the participant to stand still with arms by sides and then record weight in kilograms to the nearest one-tenth kilogram.

Waist circumference

- Waist circumference should be measured in the horizontal plane and at the midpoint between the lower margin of the last palpable rib and the top of the iliac crest.
- Measurements should be taken at the end of a normal expiration using a non-stretchable measuring tape. The tape should lie flat against the skin without compressing the soft tissue.

- The waist circumference should be measured twice, rounded to the nearest 0.5 cm. The measuring tape should be removed between the 2 measurements. Both measurements will be recorded in the CRF. If the difference between the 2 measurements exceeds 1 cm, this set of measurements should be discarded and the 2 measurements repeated.

Step 1: Ask the participant to wear light clothing (if available, patient gowns garments could also be used). The same approach should be used for each time waist circumference is measured in the study

Step 2*:* Ask the participant to stand with their feet close together, arms at their side, body weight evenly distributed.

Step 3*:* Ask the participant to relax and measure the participant's waist circumference.

Hip circumference

Locate the correct area of the hips - the widest point around the buttocks. The hip measurement should include the buttocks and the hips, and round to the nearest 0.5 cm.

10.7. Appendix 7: Prohibited Medications or Medications with Special Use Restrictions

10.7.1. Excluded/Prohibited or Restricted Use Medications

Medications within the following categories are strictly prohibited during the study. The lists below provide examples of each category of medication, but the examples are not exhaustive. As stated in Section 6.9.2, participants who have to initiate certain prohibited concomitant medications during this study will be discontinued from the study (Section 7.1).

10.7.1.1. Anti-Obesity Medications

Anti-Obesity medications within 180 days of Visit 1 or any time during the study are prohibited.

- liraglutide
- orlistat
- sibutramine
- phenylpropanolamine
- mazindol
- phentermine
- lorcaserin
- phentermine/topiramate
- naltrexone/bupropion
- ingested material that transiently occupies space in the stomach, for example, Plenity®
- over-the-counter medications, for example, allī®, and
- other incretin-based therapies.

10.7.1.2. Weight Gain Medications

Participants are excluded if they have initiated or changed dose for the following medications, which may cause weight gain, within 12 months prior to Visit 1. Common examples of these medications are the following:

- imipramine
- amitriptyline
- mirtazapine
- paroxetine
- phenelzine
- chlorpromazine
- thioridazine
- clozapine
- olanzapine
- quetiapine

- valproic acid (and its derivatives)
- lithium

Initiation of these medications during the study is strongly discouraged, and alternative therapies which do not lead to weight gain should be considered instead.

10.7.1.3. Antihyperglycemic Medications

Participants are excluded if taking antihyperglycemic medications within 90 days prior to Visit 1, or between Visit 1 and Visit 2, regardless of indication for use. Examples of exclusionary medications are the following:

- metformin
- canagliflozin
- dapagliflozin
- empagliflozin
- dulaglutide
- liraglutide
- exenatide
- tirzepatide
- sitagliptin
- saxagliptin
- linagliptin
- alogliptin

For participants with a confirmed diagnosis of T2D during the study, GLP-1 receptor agonists, GIP/GLP-1 receptor agonists and DPP-4 inhibitor are prohibited throughout the study. The following medications are examples:

- dulaglutide
- liraglutide
- exenatide
- tirzepatide
- sitagliptin
- saxagliptin
- linagliptin
- alogliptin

10.7.1.4. Strong CYP3A Inhibitors or Inducers, P-gp or BCRP Substrates with a Narrow Therapeutic Index, and OATP Inhibitors

Participants cannot be taking strong CYP3A inhibitors or inducers (exception: topical or inhaled formulations), drugs that are sensitive P-gp/BCRP substrates with a narrow therapeutic index, or strong OATP inhibitors within 2 weeks prior to randomization at Visit 2 or at any time while

taking study intervention. To be eligible for randomization into this study, those drugs need to be washed out for at least 2 weeks prior to Visit 2 and the participant should be on a stable dose of alternative medications for at least 2 weeks prior to randomization at Visit 2.

Non-exhaustive lists of examples of these medications are provided in this table.

**Drug class**	**Drug name**
Strong CYP3A4 inhibitors or inducers	
Anti-hepatitis C	• boceprevir<br>• danoprevir and ritonavir<br>• paritaprevir and ritonavir and ombitasvir and/or dasabuvir<br>• telaprevir
Antiretroviral	• cobicistat<br>• elvitegravir and ritonavir<br>• indinavir and ritonavir<br>• lopinavir and ritonavir<br>• nelfinavir<br>• ritonavir<br>• saquinavir and ritonavir<br>• tipranavir and ritonavir
Antifungal	• itraconazole<br>• ketoconazole<br>• posaconazole<br>• voriconazole
Antibiotic	• clarithromycin<br>• rifampin<br>• telithromycin<br>• troleandomycin
Other	• apalutamide<br>• carbamazepine<br>• enzalutamide<br>• grapefruit juice<br>• mitotane<br>• nefazodone<br>• phenytoin<br>• St. John's wort
Strong OATP inhibitors	
Anti-hepatitis C	• faldaprevir<br>• glecaprevir/pibrentasvir<br>• sofosbuvir/velpatasvir/voxilaprevir<br>• telaprevir

Antiretroviral	• darunavir/ritonavir<br>• elvitegravir/cobicistat/emtricitabine/tenofovir DF<br>• lopinavir/ritonavir<br>• tipranavir/ritonavir
Other	• cyclosporine<br>• rifampin
P-gp substrates with narrow therapeutic index	
Antirejection	• cyclosporine<br>• everolimus<br>• sirolimus<br>• tacrolimus
Other	• colchicine<br>• dabigatran etexilate<br>• digoxin<br>• pimozide<br>• quinidine<br>• quinine
BCRP substrates with narrow therapeutic index	
	• prazosin

[a] Participants taking these medications (ketoconazole, itraconazole, voriconazole, or posaconazole) should, if appropriate, switch to at least 2 weeks prior to randomization at Visit 2:

- miconazole, or
- clotrimazole.

[b] For participants taking clarithromycin or telithromycin, azithromycin may be substituted at least 2 weeks prior to randomization at Visit 2.

10.7.2. Medications with Special Use Restrictions

10.7.2.1. Moderate CYP3A Inhibitors and Inducers

If participants assigned to orforglipron are taking moderate CYP3A inhibitors or inducers, investigators should consider alternative medications whenever possible. Examples of these medications include

- cimetidine
- ciprofloxacin
- clotrimazole
- diltiazem
- erythromycin
- fluconazole, and
- verapamil.

10.7.2.2. Drugs Affected by Increase in Gastric pH

Drugs that may be affected by an increase in gastric pH should be separated from orforglipron administration by at least 2 to 4 hours. Examples include

Drug class	Drug name
Anti-hepatitis C	• ledipasvir + sofosbuvir (Harvoni®)<br>• sofosbuvir + velpatasvir (Epclusa®)
Antiretroviral	• atazanavir<br>• delavirdine mesylate<br>• emtricitabine + rilpivirine + tenofovir + disoproxil fumarate (Complera®)<br>• fosamprenavir<br>• nelfinavir<br>• raltegravir<br>• rilpivirine
Bisphosphonates	
Ferrous sulfate	
Levothyroxine	
Simvastatin	
Tyrosine kinase inhibitors	• acalabrutinib<br>• bosutinib<br>• dasatinib<br>• erlotinib<br>• gefitinib<br>• lapatinib<br>• nilotinib<br>• pazopanib
Other narrow therapeutic index substrates with potential pH-dependent solubility or stability	

10.8. Appendix 8: Definition and Management of Diabetes

Definition and management of incident diabetes

Definition of incident diabetes

Incident diabetes is defined when any 1 of the following occur after randomization (ADA 2023):

- unequivocal hyperglycemia (random glucose ≥200 mg/dL) with signs or symptoms of hyperglycemia
- any 2 of the following criteria are observed at the same visit, or 1 abnormal value is observed and subsequently confirmed:
  - HbA1c ≥6.5% (≥48 mmol/mol)
  - FSG ≥126 mg/dL (≥7.0 mmol/L)
- initiation of any medication for the treatment of diabetes

Confirmation of diabetes diagnosis

In the event 1 abnormal value is observed (HbA1c ≥6.5% [48 mmol/mol]) OR FSG ≥126 mg/dL (7.0 mmol/L) after randomization, the abnormal test should be repeated within 4 weeks to confirm diagnosis of diabetes and to ensure that diabetes management is initiated without delay.

The diagnosis of diabetes is confirmed if any of the following occur:

- HbA1c ≥6.5% (≥48 mmol/mol) is observed at 2 measurements any time during the study.
- FSG value ≥126 mg/dL (≥7.0 mmol/L) is observed at a consecutive FSG measurement (either at a scheduled or an unscheduled visit) following an isolated FSG value ≥126 mg/dL (≥7.0 mmol/L).
  - If diabetes diagnosis has not been confirmed at the consecutive FSG measurement, another FSG result ≥126 mg/dL (≥7.0 mmol/L) observed during the study will be considered as a new finding requiring confirmation.

Note: Once diabetes has been confirmed, repeating any future abnormal test within 4 weeks is no longer required.

Recording of incident diabetes events

- Diabetes diagnosis and the onset date as assessed by investigator will be recorded in the AE CRF.
- If the diagnosis of diabetes is based on laboratory results, the date of the first abnormal HbA1c or glucose value within the diabetes range should be indicated as the date of diagnosis, unless, in the investigator's opinion, a different date is more appropriate. If the diagnosis is based on initiation of any medication for the treatment of diabetes, the investigator should indicate the most probable date of diagnosis in the CRFs.

Management of incident diabetes

Participants who develop diabetes during the study will be

- provided and trained to use a glucometer
- educated on the signs and symptoms of hypoglycemia and its treatment, and
- instructed to report hypoglycemic episodes

Participants will be referred to their usual care provider and provided with a letter showing the study results indicative of diabetes. The decision to further evaluate, to initiate antihyperglycemic therapy, and the choice of antihyperglycemic medication will be at the discretion of the participant's usual care provider, with the exception of use of DPP-4 inhibitors and GLP-1 receptor agonists or other incretin-based therapies (for example, tirzepatide), which are prohibited during the study. Monitoring for hypoglycemia includes capture of events as defined in Section 8.3.3.4.

10.9. Appendix 9: Provisions for Changes in Study Conduct During Exceptional Circumstances

Implementation of this appendix

The changes to procedures described in this appendix are temporary measures intended to be used only during specific time periods as directed by the sponsor in partnership with the investigator.

In an exceptional circumstance, the sponsor's procedures ensure that relevant parties are kept informed of changes to the design and conduct of the study.

Exceptional circumstances

Exceptional circumstances are rare events that may cause disruptions to the conduct of the study. Examples include pandemics or natural disasters. These disruptions may limit the ability of the investigators, participants, or both to attend on-site visits or to conduct planned study procedures.

Implementing changes under exceptional circumstances

In an exceptional circumstance, after receiving the sponsor's written approval, sites may implement changes if permitted by local regulations.

After approval by local Ethical Review Boards, regulatory bodies and any other relevant local authorities, implementation of these exceptional circumstance changes will not typically require additional notification to these groups, unless they have specific requirements in which notification is required (for example, upon implementation and suspension of changes). All approvals and notifications must be retained in the study records.

If the sponsor grants written approval for changes in study conduct, the sponsor will also provide additional written guidance, if needed.

Considerations for making a change

The prevailing consideration for making a change is ensuring the safety of study participants. Additional important considerations for making a change are compliance with GCP, enabling participants to continue safely in the study and maintaining the integrity of the study.

Informed consent

Additional consent from the participant will be obtained, if required, for:

- participation in remote visits, as defined in Section "Remote Visits,"
- a change in the method of study intervention administration,
- dispensation of additional study intervention during an extended treatment period,
- alternate delivery of study intervention and ancillary supplies, and
- provision of their personal or medical information required prior to implementation of these activities.

Changes in study conduct during exceptional circumstances

Changes in study conduct not described in this appendix, or not consistent with applicable local regulations, are not allowed.

The following changes in study conduct will not be considered protocol deviations.

Remote visits

Types of remote visits

Telemedicine - Telephone or technology-assisted virtual visits, or both, are acceptable to complete appropriate assessments. Assessments to be completed in this manner include, but are not limited to,

- collection of AEs
- administer C-SSRS since last assessed
- review diary
- review diet and physical activity goals and
- concomitant medications.

Mobile healthcare - Healthcare visits may be performed by a mobile healthcare provider at locations other than the study site when participants cannot travel to the site due to an exceptional circumstance if written approval is provided by the sponsor. Procedures performed at such visits include, but are not limited to,

- concomitant medications
- vital signs (BP and Pulse Rate)
- body weight
- patient-reported outcomes
- collection of blood samples
- physical assessments, and
- collection of health information.

Data capture

In source documents and the CRF, the study site should capture the visit method, with a specific explanation for any data missing because of missed in-person site visits.

Safety reporting

Regardless of the type of remote visits implemented, the protocol requirements regarding the reporting of AEs, SAEs, and PCs remain unchanged.

Return to on-site visits

Every effort should be made to enable participants to return to on-site visits as soon as reasonably possible, while ensuring the safety of both the participants and the site staff.

Local laboratory testing option

Local laboratory testing may be conducted in lieu of central laboratory testing. However, central laboratory testing must be retained for: Visits 1, 2, 15 ED and Visit 801. The local laboratory must be qualified in accordance with applicable local regulations.

Study intervention and ancillary supplies (including participant diaries)

When a participant is unable to go to the site to receive study supplies during normal on-site visits, the site should work with the sponsor to determine appropriate actions. These actions may include

- asking the participant to go to the site and receive study supplies from site staff without completion of a full study visit,
- asking the participant's designee to go to the site and receive study supplies on a participant's behalf, and
- arranging delivery of study supplies.

These requirements must be met before action is taken:

- Alternate delivery of study intervention should be performed in a manner that does not compromise treatment blinding and ensures product integrity. The existing protocol requirements for product accountability remain unchanged, including verification of participant's receipt of study supplies.
- When delivering supplies to a location other than the study site (for example, participant's home), the investigator, sponsor, or both should ensure oversight of the shipping process to ensure accountability and product quality (that is, storage conditions maintained and intact packaging upon receipt).
- Instructions may be provided to the participant or designee on the final disposition of any unused or completed study supplies.

Adjustments to visit windows

Whenever possible and safe to do so, as determined by the investigator's discretion, participants should complete the usual SoA. To maximize the possibility that these visits can be conducted as on-site visits, the windows for visits may be adjusted, upon further guidance from the sponsor. This minimizes missing data and preserves the intended conduct of the study.

This table describes the allowed adjustments to visit windows.

Visit Number	Tolerance
Visit 2 through Visit 15	Within 10 days before or after the intended date per the SoA.
Visit 801	up to 28 days after the intended date per the SoA.

For participants whose visits have extended windows, additional study intervention may need to be provided to avoid interruption and maintain overall integrity of the study.

Documentation

Changes to study conduct will be documented

Sites will identify and document the details of how participants, visit types, and conducted activities were affected by exceptional circumstances. Dispensing/shipment records of study

intervention and relevant communications, including delegation, should be filed with site study records.

Source documents at alternate locations

Source documents generated at a location other than the study site should be part of the investigator's source documentation and should be transferred to the site in a secure and timely manner.

10.10. Appendix 10: Abbreviations and Definitions

Term	Definition
**abuse**	Use of a study intervention for recreational purposes or to maintain an addiction or dependence
**AE**	adverse event
**AESI**	Adverse Events of Special Interest
**AFAB**	Assigned female at birth
**ALP**	alkaline phosphatase
**ALT**	alanine aminotransferase
**AMAB**	Assigned male at birth
**ANCOVA**	analysis of covariance
**AOM**	anti-obesity medication
**AST**	aspartate aminotransferase
**BCRP**	breast cancer resistance protein
**BG**	blood glucose
**blinding/masking**	A single-blind study is one in which the investigator and/or the investigator's staff are aware of the treatment but the participant is not, or vice versa, or when the sponsor is aware of the treatment but the investigator and/the investigator's staff and the participant are not.<br>A double-blind study is one in which neither the participant nor any of the investigator or sponsor staff who are involved in the treatment or clinical evaluation of the subjects are aware of the treatment received.
**BMI**	body mass index
**BP**	blood pressure
**BW**	body weight
**CEC**	Clinical endpoint committee
**C-SSRS**	Columbia-Suicide Severity Rating Scale
**CIOMS**	Council for International Organizations of Medical Sciences
**CK**	creatine kinase

**complaint**	A complaint is any written, electronic, or oral communication that alleges deficiencies related to the identity, quality, purity, durability, reliability, safety or effectiveness, or performance of a drug or drug delivery system.
**compliance**	Adherence to all study-related, good clinical practice (GCP), and applicable regulatory requirements.
**CONSORT**	Consolidated Standards of Reporting Trials
**CRF**	case report form; a printed, optical, or electronic document designed to record all of the protocol-required information to be reported to the sponsor for each trial participant.
**CMV**	cytomegalovirus
**CSR**	clinical study report
**CT**	computed tomography
**CV**	cardiovascular
**CYP3A**	cytochrome P450, family 3, subfamily A
**D. Bi**	direct bilirubin
**DPP-4**	dipeptidyl peptidase-4
**EBV**	Epstein-Barr virus
**ECG**	Electrocardiogram
**ED**	Early discontinuation
**EDC**	Electronic data capture
**eGFR**	Estimated glomerular filtration rate
**enroll**	The act of assigning a participant to a treatment. Participants who are enrolled in the study are those who have been assigned to a treatment.
**enter**	Participants entered into a study are those who sign the informed consent form directly or through their legally acceptable representatives.
**ERCP**	endoscopic retrograde cholangiopancreatography
**FSG**	fasting serum glucose
**GCP**	good clinical practice
**GDPR**	General Data Protection Regulation
**GGT**	gamma-glutamyltransferase
**GI**	gastrointestinal

**GIP**	Gastric Inhibitory Polypeptide
**GLP-1**	Glucagon-like Peptide-1
**GLP-1 RAs**	Glucagon-like Peptide-1 Receptor Agonists
**HbA1c**	glycated hemoglobin
**HDV**	hepatitis D virus
**IB**	Investigator's Brochure
**ICF**	informed consent form
**ICH**	International Council for Harmonisation
**IEC**	Independent ethics committee
**IMP**	Investigational Medicinal Product (see also "investigational product")<br>A medicinal product which is being tested or used as a reference, including as a placebo, in a clinical trial.
**INR**	international normalized ratio
**informed consent**	A process by which a participant voluntarily confirms their willingness to participate in a particular study, after having been informed of all aspects of the study that are relevant to the participant's decision to participate. Informed consent is documented by means of a written, signed and dated informed consent form.
**interim analysis**	An interim analysis is an analysis of clinical study data, separated into treatment groups, that is conducted before the final reporting database is created/locked.
**investigational product**	A pharmaceutical form of an active ingredient or placebo being tested or used as a reference in a clinical trial, including products already on the market when used or assembled (formulated or packaged) in a way different from the authorized form, or marketed products used for an unauthorized indication, or marketed products used to gain further information about the authorized form. See also "IMP."
**IRB**	Institutional review board
**IWRS**	interactive web-response system

**medication error**	Errors in the prescribing, dispensing, or administration of a study intervention, regardless of whether or not the medication is administered to the participant or the error leads to an AE. Medication error generally involve a failure to uphold one or more of the five "rights" of medication use: the right participant, the right drug, the right dose, right route, at the right time.<br>In addition to the core five rights, the following may also represent medication errors:<br>• dose omission associated with an AE or a product complaint<br>• dispensing or use of expired medication<br>• use of medication past the recommended in-use date<br>• dispensing or use of an improperly stored medication<br>• use of an adulterated dosage form or administration technique inconsistent with the medication's labeling (for example, Summary of Product Characteristics, IB, local label, protocol), or<br>• shared use of cartridges, prefilled pens, or both.
**MEN-2**	multiple endocrine neoplasia-2
**misuse**	Use of a study intervention for self-treatment that either is inconsistent with the prescribed dosing regimen, indication, or both, or is obtained without a prescription
**MMRM**	Mixed Model Repeated Measures
**MRCP**	magnetic resonance cholangiopancreatography
**MRI**	magnetic resonance imaging
**MTC**	medullary thyroid carcinoma
**MTD**	maximum tolerated dose
**OATP**	organic-anion-transporting polypeptides
**participant**	Equivalent to CDISC term "subject": an individual who participates in a clinical trial, either as recipient of an investigational medicinal product or as a control
**PC**	product complaint
**P-gp**	P-glycoprotein
**PHQ-9**	Patient health questionnaire-9
**PK/PD**	pharmacokinetics/pharmacodynamics
**PPS**	per-protocol set: The set of data generated by the subset of participant who sufficiently complied with the protocol to ensure that these data would be likely to exhibit the effects of treatment, according to the underlying scientific model.
**PRO/ePRO**	patient-reported outcomes/electronic patient-reported outcomes
**PT**	prothrombin time

**QD**	once daily
**SAE**	serious adverse event
**SAP**	statistical analysis plan
**screen**	The act of determining if an individual meets minimum requirements to become part of a pool of potential candidates for participation in a clinical study.
**SD**	Standard deviation
**SGLT-2i**	Sodium-glucose co-transporter-2 inhibitor
**SoA**	Schedule of Activities
**SS**	Safety analysis set
**T1D**	type 1 diabetes
**T2D**	type 2 diabetes
**TBL**	total bilirubin level
**TEAE**	Treatment-emergent adverse event: An untoward medical occurrence that emerges during a defined treatment period, having been absent pretreatment, or worsens relative to the pretreatment state, and does not necessarily have to have a causal relationship with this treatment.
**ULN**	upper limit of normal

11. References

[ADA] American Diabetes Association. Introduction and methodology: *Standards of Care in Diabetes*. *Diab Care*. 2023;46(1):S1-S4. https://doi.org/10.2337/dc23-Sint

[ADA-EASD] American Diabetes Association. Management of hyperglycemia in Type 2 Diabetes: ADA-EASD Consensus Report 2022. Presented at the 58th EASD Annual Meeting on September 23, 2022. Accessed May 5, 2024. https://professional.diabetes.org/content-page/management-hyperglycemia-type-2-diabetes-ada-easd-consensus-report-2022

Allison DB, Downey M, Atkinson RL, et al. Obesity as a disease: a white paper on evidence and arguments commissioned by the Council of the Obesity Society. *Obesity (Silver Spring).* 2008;16(6):1161-1177. https://doi.org/10.1038/oby.2008.231

[AMA] American Medical Association House of Delegates: Recognition of obesity as a disease. Resolution: 420 (A-13). 2013. Accessed February 3, 2023. https://media.npr.org/documents/2013/jun/ama-resolution-obesity.pdf.

Alosh M, Bretz F, Huque M. Advanced multiplicity adjustment methods in clinical trials. *Stat Med*. 2014;33(4):693-713. https://doi.org/10.1002/sim.5974

Ard J, Fitch A, Fruh S, Herman L. Weight loss and maintenance related to the mechanism of action of glucagon-like peptide 1 receptor agonists. *Adv Ther*. 2021;38(6):2821-2839. http://doi.org/10.1007/s12325-021-01710-0

Aroda VR, Ratner R. The safety and tolerability of GLP-1 receptor agonists in the treatment of type 2 diabetes: a review. *Diabetes Metab Res Rev.* 2011;27(6):528-542. https://doi.org/10.1002/dmrr.1202

Aronne J, Sattar N, Horn DB, et al. Continued treatment with tirzepatide for maintenance of weight reduction in adults with obesity: the SURMOUNT-4 randomized clinical trial. *JAMA*. 2024;331(1):38-48. http://doi.org/10.1001/jama.2023.24945

Baggio LL, Drucker DJ. Biology of incretins: GLP-1 and GIP. *Gastroenterol.* 2007;132(6):2131-2157. https://doi.org/10.1053/j.gastro.2007.03.054

Banks PA, Freeman ML; Practice Parameters Committee of the American College of Gastroenterology. Practice guidelines in acute pancreatitis. *Am J Gastroenterol*. 2006;101(10):2379-2400. https://doi.org/10.1111/j.1572-0241.2006.00856.x

Berger SE, Huggins GS, McCaffery JM, et al. Change in cardiometabolic risk factors associated with magnitude of weight regain 3 years after a 1-year intensive lifestyle intervention in type 2 diabetes mellitus: The Look AHEAD Trial. *J Am Heart Assoc*. 2019;8(20):e010951. http://doi.org/10.1161/JAHA.118.010951

Bretz F, Maurer W, Brannath W, Posch M. A graphical approach to sequentially rejective multiple test procedures. *Stat Med*. 2009;28(4):586-604. https:/doi.org/10.1002.sim.3495

Bretz F, Posch M, Glimm E, et al. Graphical approaches for multiple comparison procedures using weighted Bonferroni, Simes, or parametric tests. *Biom J*. 2011;53(6):894-913. https://doi.org/10.1002/bimj.201000239

[CSAPH] Council on Science and Public Health. Report of the Council on Science and Public Health. Is obesity a disease? (Resolution 115-A-12). Council on Science and Public Health.

Report number: 3-A-13, 2013. Accessed January 31, 2023. https://www.ama-assn.org/sites/ama-assn.org/files/corp/media-browser/public/about-ama/councils/Council%20Reports/council-on-science-public-health/a13csaph3.pdf

[C-SSRS] The Columbia Lighthouse Project. The Columbia Protocol for Research. Columbia-Suicide Severity Rating Scale Scoring and Data Analysis Guide. Version 2.0. Published February 2013. Accessed October 12, 2023. http://cssrs.columbia.edu/wp-content/uploads/ScoringandDataAnalysisGuide-for-Clinical-Trials-1.pdf.

Dalton M, Finlayson G, Hill A, Blundell J. Preliminary validation and principal components analysis of the Control of Eating Questionnaire (CoEQ) for the experience of food craving. *Eur J Clin Nutr.* 2015;69(12):1313-1317. https://doi.org/10.1038/ejcn.2015.57

Danne T, Philotheou A, Goldman D, et al. A randomized trial comparing the rate of hypoglycemia – assessed using continuous glucose monitoring – in 125 preschool children with type 1 diabetes treated with insulin glargine or NPH insulin (the PRESCHOOL study). *Pediatr Diabetes.* 2013;14(8):593-601. https://doi/10.1111/pedi.12051

[EMA] European Medicines Agency. Committee for Medicinal Products for Human Use (CHMP). Guideline on clinical evaluation of medicinal products used in weight management. Published June 23, 2016. Accessed May 24, 2024. https://www.ema.europa.eu/en/documents/scientific-guideline/guideline-clinical-evaluation-medicinal-products-used-weight-management-revision-1_en.pdf.

[FAO/WHO/UNU] Food and Agriculture Organization of the United Nations/World Health Organization/United Nations University. Human energy requirements: Report of a joint FAO/WHO/UNO expert consultation. Published October 2004. Accessed May 24, 2024. http://www.fao.org/3/y5686e/y5686e00.htm

[FDA] Food and Drug Administration. Guidance for Industry. Developing products for weight management. Published February 2007. Accessed May 24, 2024. https://www.fda.gov/media/71252/download

Garvey W, Batterham RL, Bhatta M, et al. Two-year effects of semaglutide in adults with overweight or obesity: the STEP 5 trial. *Nat Med*. 2022;28(10): 2083-2091. https://doi.org/10.1038/s41591-022-02026-4

Garvey WT, Mechanick JI, Brett EM, et al. American association of clinical endocrinologists and American college of endocrinology comprehensive clinical practice guidelines for medical care of patients with obesity. *Endocr Pract*. 2016;22(suppl 3):1-203. http://doi.org/10.4158/EP161365

Jensen MD, Ryan DH, Donato KA, et al.; Expert Panel Members. Executive summary: guidelines (2013) for the management of overweight and obesity in adults: a report of the American College of Cardiology/American Heart Association Task Force on Practice Guidelines and the Obesity Society published by the Obesity Society and American College of Cardiology/American Heart Association Task Force on Practice Guidelines. Based on a systematic review from the Obesity Expert Panel, 2013. *Obesity (Silver Spring)*. 2014;22:(suppl 2):S5-S39. https://doi.org/10.1002/oby.20821

King WC, Hinerman AS, Belle SH, et al. Comparison of the performance of common measures of weight regain after bariatric surgery for association with clinical outcomes. *JAMA*. 2018;320(15):1560-1569. https://doi.org/10.1001/jama.2018.14433

Koizumi M, Takada T, Kawarada Y, et al. JPN guidelines for the management of acute pancreatitis: diagnostic criteria for acute pancreatitis. *J Hepatobiliary Pancreat Surg*. 2006;13(1):25-32. https://doi.org/10.1007/s00534-005-1048-2

Kronsteiner-Gicevic S, Tello M, Lincoln LE et al. Validation of the Rapid Prime Diet Quality Score Screener (rPDQS), A Brief Dietary Assessment Tool With Simple Traffic Light Scoring. J Acad Nutr Diet. 2023 Nov;123(11):1541-1554.e7. https://doi.org/10.1016/j.jand.2023.05.023

Look AHEAD Research Group; Wing RR, Bolin P, Brancati FL, et al. Cardiovascular effects of intensive lifestyle intervention in type 2 diabetes. *N Engl J Med*. 2013;369(2):145-154. https://doi.org/10.1056/NEJMoa1212914. Erratum in: *N Engl J Med*. 2014;370(19):1866. https://doi.org/10.1056/NEJMoa1212914

Maruish ME, ed. *User's Manual for the SF-36v2 Health Survey*. 3rd ed. Quality Metric Incorporated; 2011.

Moriarty AS, Gilbody S, McMillan D, Manea L. Screening and case finding for major depressive disorder using the Patient Health Questionnaire (PHQ-9): a meta-analysis. *Gen Hosp Psychiatry*. 2015;37(6):567-576. https://doi.org/10.1016/j.genhosppsych.2015.06.012

Nauck MA, Meier JJ, Schmidt WE. Incretin-based glucose-lowering medications and the risk of acute pancreatitis and/or pancreatic cancer: reassuring data from cardio-vascular outcome trials. *Diabetes Obes Metab.* 2017;19(9):1327-1328. https://doi.org/10.1111/dom.12981

Pastors JG, Warshaw H, Daly A, et al. The evidence for the effectiveness of medical nutrition therapy in diabetes management. *Diabetes Care.* 2002;25(3):608-613. https://doi.org/10.2337/diacare.25.3.608

Rothberg AE, McEwen LN, Kraftson AT, et al. Impact of weight loss on waist circumference and the components of the metabolic syndrome. *BMJ Open Diabetes Res Care*. 2017;5(1):e000341. https://doi.org/10.1136/bmjdrc-2016-000341

Rubin DB. Multiple imputation for nonresponse in surveys. New York: John Wiley & Sons Inc.; 1987.

Rubino D, Abrahamsson N, Davies M, et al. Effect of continued weekly subcutaneous semaglutide vs placebo on weight loss maintenance in adults with overweight or obesity: the STEP 4 randomized clinical trial. *JAMA*. 2021;325(14):1414-1425. https://doi.org/10.1001/jama.2021.3224

Saxenda [package insert]. Plainsboro, NJ: Novo Nordisk, Inc.; 2023.

Skow MA, Bergmann NC, Knop FK. Diabetes and obesity treatment based on dual incretin receptor activation: "twincretins". *Diabetes Obes Metab.* 2016;18(9):847-854. https://doi.org/10.1111/dom.12685

Spitzer RL, Kroenke K, Williams JB. Validation and utility of a self-report version of PRIME-MD: the PHQ primary care study. Primary Care Evaluation of Mental Disorders. Patient Health Questionnaire. *JAMA*. 1999;282(18):1737-44. https://doi.org/10.1001/jama.282.18.1737

Steinberg WM, Buse JB, Ghorbani MLM, et al. Amylase, lipase, and acute pancreatitis in people with type 2 diabetes treated with liraglutide: results from the LEADER randomized trial. *Diabetes Care.* 2017a;40(7):966-972. https://doi.org/10.2337/dc16-2747 [Erratum in: *Diabetes Care*. 2018;41(7):1538.]

Steinberg WM, Rosenstock J, Wadden TA, et al. Impact of liraglutide on amylase, lipase, and acute pancreatitis in participants with overweight/obesity and normoglycemia, prediabetes, or type 2 diabetes: secondary analyses of pooled data from the SCALE clinical development program. *Diabetes Care*. 2017b;40(7):839-848. https://doi.org/10.2337/dc16-2684

Stevens J, Truesdale KP, McClain JE, Cai J. The definition of weight maintenance. *Int J. Obes (Lond)*. 2006;30(3):391-399. http://doi.org/10.1038/sj.ijo.0803175

Tomlinson B, Hu M, Zhang Y, et al. An overview of new GLP-1 receptor agonists for type 2 diabetes. *Expert Opin Investig Drugs*. 2016;25(2):145-158. https://doi.org/10.1517/13543784.2016.1123249

UKPDS Group. UK Prospective Diabetes Study 7: response of fasting plasma glucose to diet therapy in newly presenting type II diabetic patients. *Metabolism.* 1990;39(9):905–912. https://doi.org/10.1016/0026-0495(90)90299-R

Wadden TA, Hollander P, Klein S, et al. Weight maintenance and additional weight loss with liraglutide after low-calorie-diet-induced weight loss: the SCALE Maintenance randomized study. *Int J Obes (Lond)*. 2013;37(11):1443-1451. https://doi.org/10.1038/ijo.2013.120

Wegovy [package insert]. Plainsboro, NJ: Novo Nordisk Inc.; 2023.

Weinberg ME, Bacchetti P, Rushakoff RJ. Frequently repeated glucose measurements overestimate the incidence of inpatient hypoglycemia and severe hyperglycemia. *J Diabetes Sci Technol.* 2010;4(3):577-582. https://doi.org/10.1177/193229681000400311

Wilding John PH, Batterham RL, Davies M, et al. Weight regain and cardiometabolic effects after withdrawal of semaglutide: the STEP 1 trial extension. *Diabetes Obes Metab* . 2022;24(8):1553-1564.

Wing RR, Lang W, Wadden TA, et al.; Look AHEAD Research Group. Benefits of modest weight loss in improving cardiovascular risk factors in overweight and obese individuals with type 2 diabetes. *Diabetes Care*. 2011;34(7):1481-1486. https://doi.org/10.2337/dc10-2415

Wharton S, Blevins T, Connery L, et al.; GZGI Investigators. Daily Oral GLP-1 Receptor Agonist Orforglipron for Adults with Obesity. *N Engl J Med*. 2023;389(10):877-888. https://doi.org/10.1056/NEJMoa2302392

[WHO] World Health Organization. WHO STEPS surveillance manual: the WHO STEPwise approach to noncommunicable disease risk factor surveillance. Updated January 26, 2017. Accessed January 05, 2022. https: //cdn.who.int/media/docs/default-source/ncds/ncd-surveillance/steps/steps-manual.pdf?sfvrsn=c281673d_7

Zepbound [package insert]. Indianapolis, IN: Eli Lilly and Company; 2023.

Signature Page for VV-CLIN-151791 v2.0

Approval	PPD<br>Statistician<br>10-Nov-2025 14:56:35 GMT+0000

Approval	PPD<br>Medical Director<br>10-Nov-2025 16:15:26 GMT+0000

Signature Page for VV-CLIN-151791 v2.0

Title Page

Protocol Title: A Phase 3b, Randomized, Double-Blind, Placebo-Controlled Study to Evaluate the Efficacy and Safety of Orforglipron Once Daily Versus Placebo for Maintenance of Body Weight Reduction in Participants Who Have Obesity or Overweight with Weight-Related Comorbidities (ATTAIN- MAINTAIN)

Protocol Number: J2A-MC-GZPN

Compound Number: LY3502970

Short Title: Efficacy and Safety of Orforglipron Compared with Placebo for Maintenance of Body Weight Reduction in Participants with Obesity or Overweight with Weight-Related Comorbidities

Acronym: ATTAIN-MAINTAIN

Sponsor Name: Eli Lilly and Company

Legal Registered Address: Indianapolis, Indiana, USA 46285

Regulatory Agency Identifier Number(s)

Registry	ID
IND	156143

Confidential Information

The information contained in this document is confidential and the information contained within it may not be reproduced or otherwise disseminated without the approval of Eli Lilly and Company or its subsidiaries

Note to Regulatory Authorities: This document may contain protected personal data and/or commercially confidential information exempt from public disclosure. Eli Lilly and Company requests consultation regarding release/redaction prior to any public release. In the United States, this document is subject to Freedom of Information Act (FOIA) Exemption 4 and may not be reproduced or otherwise disseminated without the written approval of Eli Lilly and Company or its subsidiaries.

Document ID: VV-CLIN-156239

Table of Contents

Title Page 1
Table of Contents 2
Version history 6
1. Introduction 7
1.1. Objectives, Endpoints, and Estimands 8
1.2. Study Design 15
2. Statistical Hypotheses 18
2.1. Multiplicity Adjustment 18
3. Analysis Sets 21
4. Statistical Analyses 24
4.1. General Considerations 24
4.1.1. Baseline Definition 25
4.1.2. Analysis Methods 27
4.2. Participant Dispositions 33
4.3. Primary Endpoints/Estimands Analysis 34
4.3.1. Definition of Endpoint 34
4.3.2. Main Analytical Approach 34
4.3.3. Supplementary Analyses 34
4.4. Secondary Endpoints/Estimands Analysis 34
4.4.1. Key Secondary Endpoints 34
4.4.2. Additional Secondary Endpoint 35
4.5. Exploratory Endpoints Analysis 35
4.5.1. Exploratory Analysis Specified in the Protocol 35
4.5.2. Other Exploratory Analysis Not Specified in Protocol 37
4.6. Safety Analyses 38
4.6.1. Extent of Exposure 38
4.6.2. Adverse Events 39
4.6.3. Special Safety Topics 41
4.6.4. Clinical Laboratory Evaluation 48
4.6.5. Vital Signs and Physical Characteristics 48
4.6.6. Electrocardiograms 48
4.7. Other Analyses 48
4.7.1. Health Outcomes 48
4.7.2. Subgroup Analyses 52
4.8. Interim Analysis 52
4.9. Changes to Protocol-Planned Analyses 52

5. Sample Size Determination 53
6. Supporting Documentation 54
6.1. Appendix 1: Demographic and Baseline Characteristics 54
6.2. Appendix 2: Historical Illnesses and Preexisting Conditions 56
6.3. Appendix 3: Treatment Compliance 56
6.4. Appendix 4: Prior/Concomitant Medications 56
6.5. Appendix 5: Important Protocol Deviations 58
6.6. Appendix 6: Clinical Trial Registry Analyses 58
7. References 60

Table of Contents

Tables **Page**

Table GZPN.1.1. Visit Schedule 17

Table GZPN.3.1. Description of Analysis Population and Analysis Datasets 21

Table GZPN.4.1. Baseline and Postbaseline Definitions for Safety Outcomes 26

Table GZPN.4.2. Imputation Procedure 30

Table GZPN.4.3. Listings and Summary Tables Related to Dispositions....34

Table GZPN.4.4. Summary Tables Related to Adverse Events 40

Table GZPN.4.5. Description and Analyses of Safety Topics of Interest 41

Table GZPN.4.6. Summary Tables Related to Clinical Laboratory Evaluations 48

Table GZPN.4.7. Summary Tables Related to Vital Signs 48

Table GZPN.6.1. Demographics and Baseline Characteristics with Variables for Subgroup Analysis....55

Table of Contents

Figure **Page**

Figure GZPN.1.1. Illustration of study design for clinical protocol J2A-MC-GZPN. 16

Figure GZPN.2.1. Graphical testing scheme for the tirzepatide group......................................19

Figure GZPN.2.2. Graphical testing scheme for the semaglutide group. 20

Version history

This Statistical Analysis Plan (SAP) for Study J2A-MC-GZPN (GZPN) is based on the GZGU(a) Harmonized Clinical Protocol dated 10 Nov 2025.

SAP Version History Summary

SAP Version	Approval Date	Change	Rationale
1	See date on Page 1	Not Applicable	Original version

1. Introduction

This SAP describes the pre-specified statistical analyses for Study GZPN. These analyses apply to efficacy and safety data.

Changes to the protocol-planned analyses are described in Section 4.9.

1.1. Objectives, Endpoints, and Estimands

Participants treated with tirzepatide in SURMOUNT-5 Study

Objectives	Endpoints
**Primary**	
To demonstrate that orforglipron 36 mg or MTD (24 mg or 36 mg) is superior to placebo at Week 52 for the mean percent maintenance of BW reduction achieved with 72 weeks of treatment with tirzepatide 15 mg or MTD (10 mg or 15 mg) in participants who have reached a BW plateau[a]	Percent maintenance of BW reduction achieved during the 72 weeks of tirzepatide treatment
**Key Secondary**	
To demonstrate that orforglipron 36 mg or MTD (24 mg or 36 mg) is superior to placebo at Week 52 for the mean percent change in BW from SURMOUNT-5 baseline in all participants	Percent change in BW from SURMOUNT-5 baseline prior to the initiation of tirzepatide treatment
To demonstrate that orforglipron 36 mg or MTD (24 mg or 36 mg) is superior to placebo at Week 52 for the mean percent maintenance of BW reduction achieved with 72 weeks of treatment with tirzepatide 15 mg or MTD (10 mg or 15 mg) in all participants	Percent maintenance of BW reduction achieved during the 72 weeks of tirzepatide treatment
To demonstrate that orforglipron 36 mg or MTD (24 mg or 36 mg) is superior to placebo at Week 52 for maintenance of BW reduction achieved with 72 weeks treatment of tirzepatide 15 mg or MTD (10 mg or 15 mg) in participants who have reached a BW plateau[a]	Assessment (yes/no) of maintaining ≥80% of the BW reduction achieved during 72 weeks of tirzepatide treatment
**Additional Secondary**	
To demonstrate that orforglipron 36 mg or MTD (24 mg or 36 mg) is superior to placebo at Week 52 for maintenance of BW reduction achieved with 72 weeks treatment of tirzepatide 15 mg or MTD (10 mg or 15 mg) in participants who have reached a BW plateau[a]	Assessment (yes/no) of maintaining ≥15% BW reduction from SURMOUNT-5 baseline for participants who have already lost ≥15% BW after 72-week of tirzepatide treatment
To compare orforglipron 36 mg or MTD (24 mg or 36 mg) to placebo at Week 52 in participants who have reached a BW plateau[a] for BW endpoints	• From randomization to Week 52<br>○ change in BW (kg)<br>○ percent change in BW<br>○ change in waist circumference (cm)
To compare orforglipron 36 mg or MTD (24 mg or 36 mg) to placebo at Week 24 in participants prior to receiving rescue orforglipron who have reached a BW plateau[a] for mean percent maintenance of BW reduction achieved during the 72 weeks of tirzepatide treatment	Percent maintenance of BW reduction achieved during the 72 weeks of tirzepatide treatment

Objectives	Endpoints
To describe the safety of orforglipron 36 mg or MTD (24 mg or 36 mg) in all participants enrolled in the study	• Summary of safety data, including number and incidence of<br>o SAEs<br>o TEAEs<br>o discontinuations due to AEs
**Exploratory**	
To evaluate orforglipron 36 mg and/or MTD (24 mg or 36 mg) to placebo at Week 52 in participants who have reached a BW plateau[a] before randomization for change in:	Change from before randomization at Week 52
• lipid parameters	• total cholesterol<br>• HDL-cholesterol<br>• LDL-cholesterol<br>• VLDL-cholesterol<br>• non-HDL cholesterol<br>• triglycerides
• blood pressure parameters	systolic blood pressure (mmHg).
• glycemic parameters	• fasting glucose (mg/dL)<br>• fasting insulin, and<br>• HbA1c (%)
• rescue AOM use	Use of rescue orforglipron for weight regain ≥50% of the BW reduction achieved with tirzepatide treatment
• BW endpoints	• Change in BMI (kg/m^2)<br>• Assessment (yes/no) of maintenance of BW reduction ≥20% BW reduction from SURMOUNT-5 baseline for participants who have already lost ≥20% with tirzepatide treatment
• patient-reported outcomes	• SF-36v2 acute form domain and summary scores<br>• CoEQ scores<br>• Proportion of participants with improved categorical shift in:<br>o PGIS-Physical Function Weight<br>o PGIS-Food Craving<br>• Proportion of participants with improvements in:<br>o PGIC-Physical Function Weight<br>o PGIC-Food Craving<br>• rPDQS score

Objectives	Endpoints
**Exploratory – Not specified in protocol**To compare orforglipron 36 mg or MTD (24 mg or 36 mg) to placebo at Week 52 in participants who have reached a BW plateau[a] for BW endpointsAssessment (yes/no) of continuing to lose weight after 72 weeks of tirzepatide treatment from randomizationTo compare orforglipron 36 mg or MTD (24 mg or 36 mg) to placebo at Week 52 in participants who have reached a BW plateau[a] for BW endpoints	Proportion of participants achieving body weight change thresholds values after randomization<br>• <5%

To compare orforglipron 36 mg or MTD (24 mg or 36 mg) to placebo for delaying progression to prediabetes or T2D at 52 weeks.Time to onset of prediabetes or T2D [b] Abbreviations: AE = adverse event; BMI = body mass index; BW = body weight; CoEQ = Control of Eating Questionnaire-NRS; HbA1c = hemoglobin A1c; HDL = high-density lipoprotein; LDL = low-density lipoprotein; MTD = maximum tolerated dose; PGIC = Patient Global Impression of Change; PGIS = Patient Global Impression of Severity; rPDQS = rapid Prime Diet Quality Score; SAE = serious adverse event; SF-36v2 = Short Form-36 version 2 Health Survey acute form; SURMOUNT-5 = Study I8F-MC-GPHJ (GPHJ); TEAE = treatment-emergent adverse event; VLDL = very-low-density lipoprotein; T2D = type 2 diabetes.

[a] BW plateau is defined as <5% BW change between Visit 17 (Week 60) and Visit 20 (Week 72) of SURMOUNT-5 study.

[b] The diagnosis of prediabetes and T2D will be consistent with ADA recommendations (ADA 2022).

Participants treated with semaglutide in SURMOUNT-5 Study

Objectives	Endpoints
**Primary**	
To demonstrate that orforglipron 36 mg or MTD (24 mg or 36 mg) is superior to placebo at Week 52 for the mean percent maintenance of BW reduction achieved with 72 weeks of treatment with semaglutide 2.4 mg or MTD (1.7 mg or 2.4 mg) in participants who have reached a BW plateau[a]	Percent maintenance of BW reduction achieved during the 72 weeks of semaglutide treatment
**Key Secondary**	
To demonstrate that orforglipron 36 mg or MTD (24 mg or 36 mg) is superior to placebo at Week 52 for the mean percent change in BW from SURMOUNT-5 baseline in all participants	Percent change in BW from SURMOUNT-5 baseline prior to the initiation of semaglutide treatment
To demonstrate that orforglipron 36 mg or MTD (24 mg or 36 mg) is superior to placebo at Week 52 for the mean percent maintenance of BW reduction achieved with 72 weeks of treatment with semaglutide 2.4 mg or MTD (1.7 mg or 2.4 mg) in all participants	Percent maintenance of BW reduction achieved during the 72 weeks of semaglutide treatment
To demonstrate that orforglipron 36 mg or MTD (24 mg or 36 mg) is superior to placebo at Week 52 for maintenance of BW reduction achieved with 72 weeks of treatment with semaglutide 2.4 mg or MTD (1.7 mg or 2.4 mg) in participants who have reached a BW plateau[a]	Assessment (yes/no) of maintaining ≥80% of the BW reduction achieved during 72 weeks of semaglutide treatment

Objectives	Endpoints
**Additional Secondary**	
To demonstrate that orforglipron 36 mg or MTD (24 mg or 36 mg) is superior to placebo at Week 52 for maintenance of BW reduction achieved with 72 weeks treatment of semaglutide 2.4 mg or MTD (1.7 mg or 2.4 mg) in participants who have reached a BW plateau[a]	Assessment (yes/no) of maintaining ≥15% BW reduction from SURMOUNT-5 baseline for participants who have already lost ≥15% BW after 72 weeks of semaglutide treatment
To compare orforglipron 36 mg or MTD (24 mg or 36 mg) to placebo at Week 52 in participants who have reached a BW plateau[a] for BW endpoints	• From randomization to Week 52:<br>o change in BW (kg)<br>o percent change in BW<br>o change in waist circumference (cm)
To compare orforglipron 36 mg or MTD (24 mg or 36 mg) to placebo at Week 24 in participants prior to receiving rescue orforglipron who have reached a BW plateau[a] for mean percent maintenance of BW reduction achieved during the 72 weeks of semaglutide treatment	Percent maintenance of BW reduction achieved during the 72 weeks of semaglutide treatment
To describe the safety of orforglipron 36 mg or MTD (24 mg or 36 mg) in all participants enrolled in the study	• Summary of safety data, including number and incidence of<br>o SAEs<br>o TEAEs<br>o discontinuations due to AEs
**Exploratory**	
To compare orforglipron 36 mg and/or MTD (24 mg or 36 mg) to placebo at Week 52 in participants who have reached a BW plateau[a] before randomization for change in:	Change from before randomization at Week 52
• lipid parameters	• total cholesterol<br>• HDL-cholesterol<br>• LDL-cholesterol<br>• VLDL-cholesterol<br>• non-HDL cholesterol<br>• triglycerides
• blood pressure parameters	systolic blood pressure (mmHg)
• glycemic parameters	• fasting glucose (mg/dL)<br>• fasting insulin<br>• HbA1c (%)
• rescue AOM use	Use of rescue orforglipron for weight regain ≥50% of the BW reduction achieved with semaglutide treatment.

Objectives	Endpoints
• BW endpoints	• Change in BMI (kg/m^2).<br>• Assessment (yes/no) of maintenance of BW reduction ≥20% BW reduction from SURMOUNT-5 baseline for participants who have already lost ≥20% with semaglutide treatment
• patient-reported outcomes	• SF-36v2 acute form domain and summary scores<br>• CoEQ scores<br>• Proportion of participants with improved categorical shift in:<br>o PGIS-Physical Function Weight<br>o PGIS-Food Craving<br>• Proportion of participants with improvements in:<br>o PGIC-Physical Function Weight<br>o PGIC-Food Craving<br>• rPDQS score
**Exploratory – Not specified in protocol**	
To compare orforglipron 36 mg or MTD (24 mg or 36 mg) to placebo at Week 52 in participants who have reached a BW plateau[a] for BW endpoints	Assessment (yes/no) of continuing to lose weight after 72 weeks of tirzepatide treatment from randomization
To compare orforglipron 36 mg or MTD (24 mg or 36 mg) to placebo at Week 52 in participants who have reached a BW plateau [a] for BW endpoints	Proportion of participants achieving body weight change thresholds values after randomization<br>• <5%
To compare orforglipron 36 mg or MTD (24 mg or 36 mg) to placebo for delay progression to prediabetes or T2D at 52 weeks.	• Time to onset of prediabetes or T2D [b]

Abbreviations: AE = adverse event; BMI = body mass index; BW = body weight; CoEQ = Control of Eating Questionnaire-NRS; HbA1c = hemoglobin A1c; HDL = high-density lipoprotein; LDL = low-density lipoprotein; MTD = maximum tolerated dose; PGIC = Patient Global Impression of Change; PGIS = Patient Global Impression of Severity; rPDQS = rapid Prime Diet Quality Score; SAE = serious adverse event; SF-36v2 = Short Form-36 version 2 Health Survey acute form; SURMOUNT-5 = Study I8F-MC-GPHJ (GPHJ); TEAE = treatment-emergent adverse event; VLDL = very-low-density lipoprotein; T2D = type 2 diabetes.

a BW plateau is defined as <5% BW change between Visit 17 (Week 60) and Visit 20 (Week 72) of SURMOUNT-5 study.

b The diagnosis of prediabetes and T2D will be consistent with ADA recommendations (ADA 2022).

Estimands

There will be 2 main estimands planned in the study, modified treatment regimen estimand and efficacy estimand. Unless otherwise specified or requested by a regulatory agency, modified treatment regimen estimand will be the primary estimand, with the efficacy estimand considered supportive. Both the treatment-regimen and efficacy estimands will be evaluated for the primary and key secondary objectives. In addition, supplementary estimands may be considered for the primary endpoint.

Modified treatment-regimen estimand

This estimand aims at reflecting how participants with obesity or overweight with at least 1 weight-related comorbid condition are treated in clinical practice and takes into account both tolerability and efficacy.

For each treatment in the SURMOUNT-5 study: (tirzepatide or semaglutide), this modified treatment-regimen estimand answers the following question of interest for the primary objective: *What is the treatment difference at Week 52 for orforglipron versus placebo in mean percent maintenance of BW reduction achieved during 72 weeks of treatment with tirzepatide or semaglutide, respectively, as an adjunct to a reduced-calorie diet and increased physical activity in participants with obesity or overweight with at least 1 weight-related comorbid condition, regardless of treatment discontinuation for any reasons and regardless of initiation of other AOMs, GLP-1 RAs, GIP/GLP-1 RAs, or DPP-4 inhibitors. This estimand also assumes that participants who had bariatric surgery or another weight-loss procedure or took rescue orforglipron would not have received any additional improvement from their randomized study treatment?*

The modified treatment-regimen estimand is described by the following attributes:

- **Population** - Participants who meet the eligibility criteria. Further details can be found in Section 5 of the protocol.
- **Endpoints** - Mean percent maintenance of BW reduction achieved during the 72 weeks of treatment with tirzepatide or semaglutide, respectively.
- **Treatment condition -** The randomized treatment as an adjunct to a reduced-calorie diet and increased physical activity regardless of adherence to treatment with or without other AOMs, GLP-1 RAs, GIP/GLP-1 RA, or DPP-4 inhibitors. Further details on study treatment and concomitant therapy can be found in Section 6 of the protocol.
- **Intercurrent events** - "treatment discontinuation for any reason" and "initiation of other AOMs, GLP-1 RAs, GIP/GLP-1 RA, or DPP-4 inhibitor" are addressed by the treatment condition. Bariatric surgery or other weight-loss procedures or rescue orforglipron will be addressed by the hypothetical strategy. It will be assumed that participants who undergo bariatric surgery, weight-loss procedures, or rescue orforglipron would not have received any additional improvement from their randomized treatment.
- **Population-level summary and treatment effect of interest** - Difference in the mean of the endpoint between treatment conditions at Week 52.

Efficacy estimand

For each treatment in the SURMOUNT-5 study: (tirzepatide or semaglutide), the efficacy estimand answers the following question of interest for the primary objective:

What is the treatment difference at Week 52 for orforglipron versus placebo in mean percent maintenance of body weight (BW) reduction achieved during 72 weeks of treatment with

tirzepatide or semaglutide, respectively, as an adjunct to a reduced-calorie diet and increased physical activity in participants with obesity or overweight with at least 1 weight-related comorbid condition, assuming that participants had stayed on treatment, had not taken other AOMs, GLP-1 RAs, GIP/GLP-1 RAs, or DPP-4 inhibitors, had not had bariatric surgery or other weight management procedures, and assuming that participants who took rescue orforglipron would not have received any additional improvement from their randomized study treatment?

The efficacy estimand is described by the following attributes:

- **Population –** Participants who meet the eligibility criteria . Further details can be found in Section 5 of the protocol.
- **Endpoints** – Mean percent maintenance of BW reduction achieved during the 72 weeks of treatment with tirzepatide or semaglutide, respectively.
- **Treatment condition –** The randomized treatment as an adjunct to a reduced-calorie diet and increased physical activity. Further details on study treatment can be found in Section 6 of the protocol.
- **Intercurrent events –** "Treatment discontinuation for any reason," "Initiation of other AOMs, GLP-1 RAs, GIP/GLP-1 RAs or DPP-4 inhibitors," "having bariatric surgery or other weight management procedures," or "taking rescue *orforglipron*" will be addressed using the hypothetical strategy:
  - had participants stayed on treatment
  - had participants not taken other AOMs, GLP-1 RAs, GIP/GLP-1 RAs, or DPP-4 inhibitors and had not had bariatric surgery or other weight-loss procedure, and
  - assuming that participants who took rescue orforglipron would not have received any additional improvement from their randomized treatment.
- **Population-level summary and treatment effect of interest** – Difference in mean of the endpoint between treatment conditions at Week 52.

Supplementary treatment regimen estimand

This estimand focuses on the treatment effect for orforglipron versus placebo regardless of adherence to study treatment, or initiation of other AOMs, GLP-1 RAs, GIP/GLP-1 RA, or DPP-4 inhibitors, or having bariatric surgery or weight-loss procedures, and regardless of taking rescue orforglipron.

For each treatment in the SURMOUNT-5 study: (tirzepatide or semaglutide), the supplementary treatment-regimen estimand answers the following question of interest for the primary objective: *What is the treatment difference at Week 52 for orforglipron versus placebo in mean percent maintenance of BW reduction achieved during 72 weeks of treatment with tirzepatide or semaglutide, respectively, as an adjunct to a reduced-calorie diet and increased physical activity*

in participants with obesity or overweight with at least 1 weight-related comorbid condition, regardless of treatment discontinuation for any reasons, regardless of initiation of other AOMs, GLP-1 RAs, GIP/GLP-1 RAs, or DPP-4 inhibitors, regardless of initiation of bariatric surgery or another weight-loss procedure, and regardless of the initiation of rescue orforglipron?

The modified treatment-regimen estimand is described by the following attributes:

- **Population** - Participants who meet the eligibility criteria Further details can be found in Section 5 of the protocol.
- **Endpoints** - Mean percent maintenance of BW reduction achieved during the 72 weeks of treatment with tirzepatide or semaglutide, respectively.
- **Treatment condition -** The randomized treatment as an adjunct to a reduced-calorie diet and increased physical activity regardless of adherence to treatment with or without other AOMs, GLP-1 RAs, GIP/GLP-1 RA, DPP-4 inhibitors, bariatric surgery, weight-loss procedures, or rescue orforglipron.
- **Intercurrent events** - All ICEs are handled by the treatment policy estimand and are included in the treatment condition.
- **Population-level summary and treatment effect of interest** - Difference in the mean of the endpoint between treatment conditions at Week 52.

1.2. Study Design

Study GZPN is a Phase 3b, multicenter, randomized, double-blind, placebo controlled study that will investigate efficacy and safety of orforglipron 36 mg or MTD [24 mg or 36 mg] compared with placebo, in achieving maintenance of body weight reduction from the 72 weeks of tirzepatide or semaglutide treatment in participants who have obesity or overweight with weight-related coexisting conditions and previously participated in SURMOUNT-5 study.

For each treatment in the SURMOUNT-5 study: (tirzepatide or semaglutide), the study will consist of 3 periods: an up to 2-week screening period; a 52-week double-blind treatment period (including a 8-week dose escalation period) and a 2-week safety follow-up period. The study participants will be randomly assigned in a 3:2 ratio (orforglipron 36 mg or MTD [24 mg or 36 mg] and placebo at the end of the screening period.

Schema

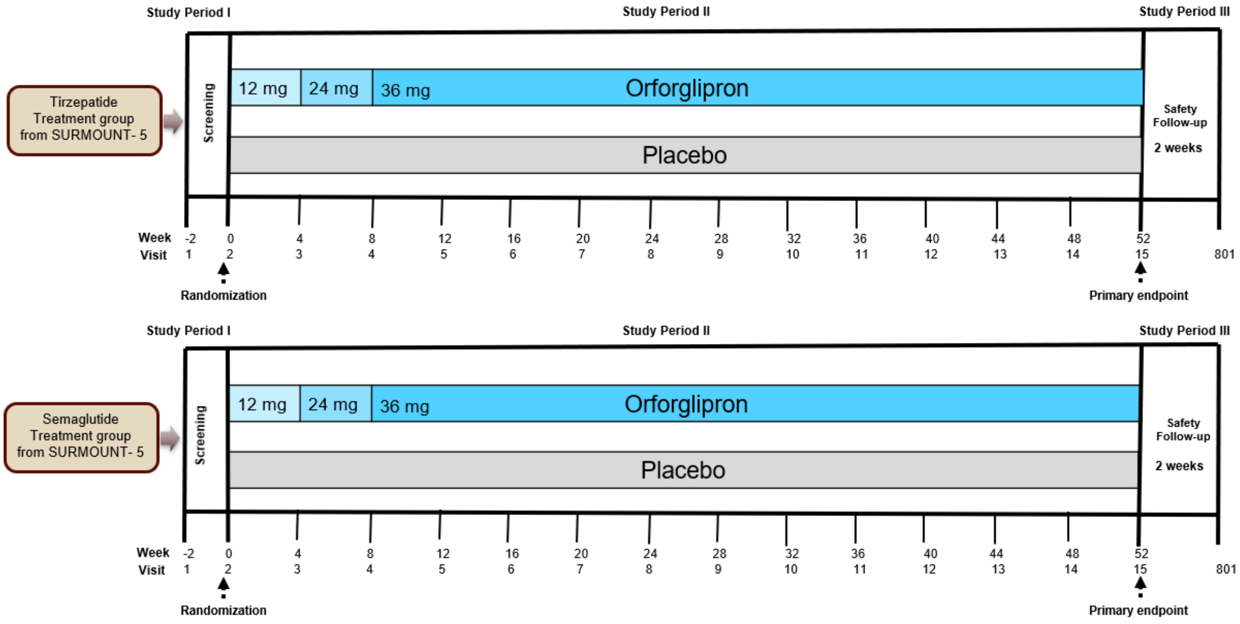

Figure GZPN.1.1. Illustration of study design for clinical protocol J2A-MC-GZPN.

The visit frequency is listed in Table GZPN.1.1.

Table GZPN.1.1. Visit Schedule

	Visit Number	Weeks from Visit 2 (Randomization)
**Screening period**	1	-14 to -7 days
**Treatment period**	2	0
	3	4
	4	8
	5T	12
	6	16
	7T	20
	8	24
	9T	28
	10T	32
	11	36
	12T	40
	13T	44
	14	48
	15	52
	ED	
**Safety follow-up period**	801	2 weeks post end-of-treatment period

Abbreviation: ED = early discontinuation. T = telephone visit.

All participants will be centrally randomized in a 3:2 ratio using an IWRS.

Potential bias will be reduced by central randomization, and the randomization will be stratified by the following factors:

- achieving plateau at Week 72 of SURMOUNT-5 study (yes/no)
- sex (individual AFAB, individual AMAB), and
- percent weight loss at Week 72 of SURMOUNT-5 study (<20% versus ≥20%).

Participants are considered to have achieved plateau if percent change in weight from Week 60 to Week 72 from SURMOUNT-5 study is less than 5%.

2. Statistical Hypotheses

For each treatment in the *SURMOUNT-5 study (tirzepatide or semaglutide), the null hypothesis corresponding to the primary objective of this study is as follows:*

- **Null hypothesis** ***($H_{1,0\ (TZP)}$, $H_{1,0\ (SEMA)}$)***: *Orforglipron 36 mg or MTD (24 mg or 36 mg) is not superior to placebo at Week 52 with respect to mean percent maintenance of BW reduction achieved during 72 weeks of treatment with tirzepatide 15 mg or MTD (10 mg or 15 mg) or semaglutide 2.4 mg or MTD (1.7 mg or 2.4 mg), respectively, in participants who have reached a BW plateau. For each treatment in the SURMOUNT-5 study (tirzepatide or semaglutide), the null hypotheses corresponding to the key secondary objective is as follows:*
- **Null Hypothesis** ***($H_{2,0\ (TZP)}$, $H_{2,0\ (SEMA)}$)***: *Orforglipron 36 mg or MTD (24 mg or 36 mg) is not superior to placebo at Week 52 with respect to mean percent change in BW from baseline prior to the initiation of tirzepatide or semaglutide treatment, respectively (Week 0 of SURMOUNT-5 study) in all participants.*
- **Null Hypothesis (**$H_{3,0\ (TZP)}$, $H_{3,0\ (SEMA)}$**)**: *Orforglipron 36 mg or MTD (24 mg or 36 mg) is not superior to placebo at Week 52 with respect to mean percent maintenance of BW reduction achieved during 72 weeks of treatment with tirzepatide 15 mg or MTD (10 mg or 15 mg) or semaglutide 2.4 mg or MTD (1.7 mg or 2.4 mg), respectively, in all participants.*
- **Null Hypothesis (**$H_{4,0\ (TZP)}$, $H_{4,0\ (SEMA)}$**)**: Orforglipron 36 mg or MTD (24 mg or 36 mg) is not superior to placebo at Week 52 with respect to the percentage of participants maintaining ≥80% of the BW reduction achieved during 72 weeks of treatment with tirzepatide 15 mg or MTD (10 mg or 15 mg) or semaglutide 2.4 mg or MTD (1.7 mg or 2.4 mg), respectively, in participants who reached a BW plateau.

2.1. Multiplicity Adjustment

Multiplicity adjusted analyses will be performed on the primary and key secondary objectives to control the overall Type-I error rate. The tirzepatide and semaglutide treatment groups in the SURMOUNT-5 study are treated as independent studies and will be analyzed separately. Each will be tested at a two-sided significance level of 0.05. , a 2-sided alpha level of 0.05 will be used. The graphical multiple testing procedure described in Bretz et al. (2009, 2011) will be used. This approach is a closed testing procedure; hence, it strongly controls the Type-I error rate across all hypotheses (Alosh et al. 2014).

Figure GZPN.2.1 and Figure GZPN.2.2 illustrate the final graphical testing scheme for tirzepatide and semaglutide treatment groups in the SURMOUNT-5 study, including testing order, interrelationships, Type-I error allocation for the primary and key secondary objectives, and the associated propagation. The modified treatment regimen estimand will be the primary estimand, with the efficacy estimand considered supportive. Since these estimands are intended for distinct purposes, no multiplicity adjustment will be made for conducting separate

analyses on the same objectives. Unless otherwise specified, there will be no adjustment for multiple comparisons for any other analyses outside the primary and key secondary objectives.

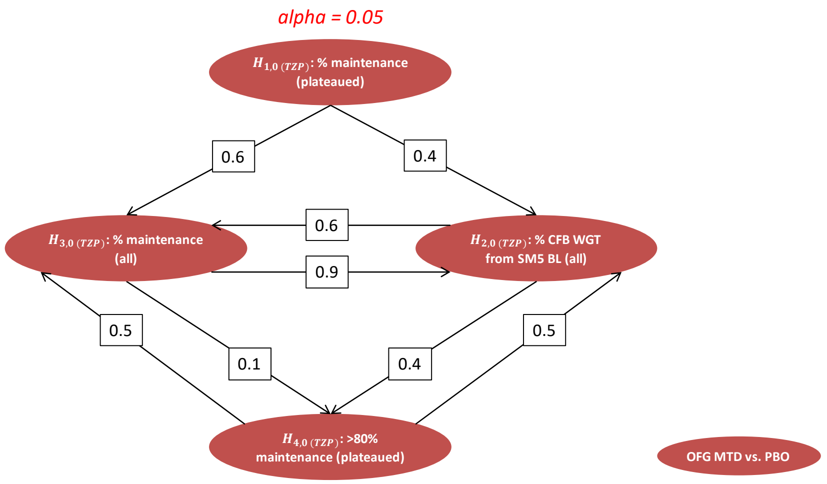

Abbreviations: BL = baseline; CFB = change from baseline; MTD = maximum tolerated dose; OFG = orforglipron; PBO = placebo; SM5 = SURMOUNT-5 study; TZP = tirzepatide.

Figure GZPN.2.1. Graphical testing scheme for the tirzepatide group.

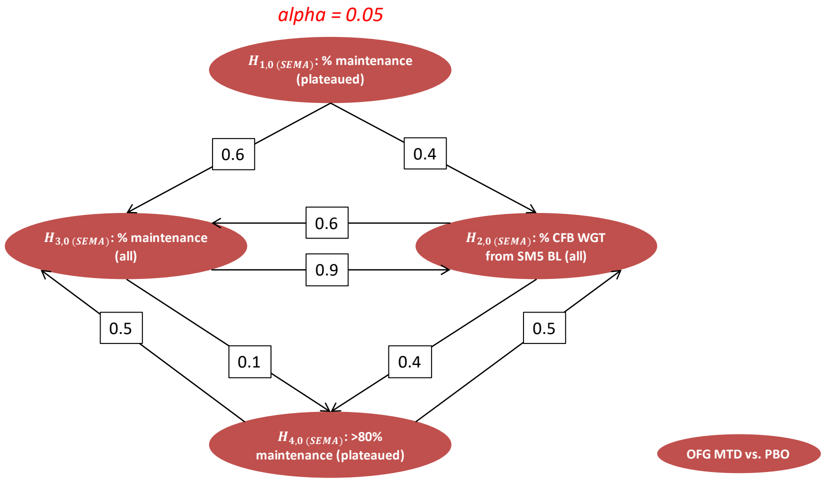

Abbreviations: BL = baseline; CFB = change from baseline; MTD = maximum tolerated dose; OFG = orforglipron; PBO = placebo; SEMA = semaglutide; SM5 = SURMOUNT-5 study.

Figure GZPN.2.2. Graphical testing scheme for the semaglutide group.

3. Analysis Sets

The tables define the analysis populations and datasets for the purposes of analysis based on the estimands defined in Section 1.1.

Table GZPN.3.1. Description of Analysis Population and Analysis Datasets

Participant Analysis Set	Description
Screened population - Tirzepatide or Semaglutide, respectively	Participants from SURMOUNT-5 (SM5) study treated with tirzepatide or semaglutide, respectively, who signed informed consent for this study.
Randomized population – Tirzepatide or Semaglutide, respectively	Participants from SURMOUNT-5 study who were treated with tirzepatide or semaglutide, respectively, and who are randomly assigned to a treatment arm (orforglipron or placebo) in this study.
Modified intent-to-treat population (mITT) – Tirzepatide or Semaglutide, respectively	Randomly assigned participants from SURMOUNT-5 study who were treated with tirzepatide or semaglutide, respectively, who are exposed to at least 1 dose of study intervention in this study. Participants who are inadvertently enrolled will be excluded.
Modified intent-to-treat population (mITT) with BW plateau – Tirzepatide or Semaglutide, respectively	Randomly assigned participants from SURMOUNT-5 study who were in BW plateau and were treated with tirzepatide or semaglutide, respectively, who are exposed to at least 1 dose of study intervention in this study. Participants who are inadvertently enrolled will be excluded.
Safety participants – Tirzepatide or Semaglutide, respectively	Randomly assigned participants from SURMOUNT-5 study who were treated with tirzepatide or semaglutide, respectively, who are exposed to at least 1 dose of study intervention. Participants will be analyzed according to the treatment they were randomly assigned to regardless of the treatment actually received.

Abbreviations: BW = body weight; OFG = orforglipron; MTD = maximum tolerated dose; PBO = placebo.

[a] Refers to the informed consent for the study.

The following analysis data sets are defined to estimate the estimands defined in the protocol and to address safety.

Analysis Datasets	Description
Full Analysis Set (FAS) – Tirzepatide or Semaglutide, respectively: This analysis set will be used to estimate the modified treatment-regimen estimand	Data obtained during Study Period II from the mITT Population - Tirzepatide or Semaglutide, respectively, for efficacy analyses population regardless of adherence to study treatment and regardless of initiation of other AOMs, GLP-1 RAs, GIP/GLP-1 RAs, or DPP-4 inhibitors. Data obtained after rescue orforglipron or having bariatric surgery or other weight-loss procedures will be excluded.
Full Analysis Set (FAS2) - Tirzepatide or Semaglutide, respectively: This analysis set will be used to estimate the supplementary treatment-regimen estimand	Data obtained during Study Period II from the mITT Population - Tirzepatide or Semaglutide, respectively, for efficacy analyses population regardless of adherence to study treatment and regardless of initiation of other AOMs, GLP-1 RAs, GIP/GLP-1 RAs, or DPP-4 inhibitors, regardless of initiation of rescue orforglipron or having bariatric surgery or other weight-loss procedures.
Efficacy Analysis Set (EAS) – Tirzepatide or Semaglutide, respectively: This analysis set will be used to estimate the efficacy estimand	Data obtained during Study Period II from the mITT Population - Tirzepatide or Semaglutide, respectively, for efficacy analyses population, excluding data after permanent discontinuation of treatment, initiation of other AOMs, GLP-1 RAs, GIP/GLP-1 RAs, DPP-4 inhibitors, or rescue orforglipron, or having bariatric surgery or other weight-loss procedures.
Full Analysis Set for weight plateau (FAS - WP) – Tirzepatide or Semaglutide, respectively: This analysis set will be used to estimate the modified treatment-regimen estimand	Data obtained during Study Period II from the mITT population with BW Plateau – Tirzepatide or Semaglutide, respectively, for efficacy analyses population regardless of adherence to study treatment and regardless of initiation of other AOMs, GLP-1 RAs, GIP/GLP-1 RAs, or DPP-4 inhibitors. Data obtained after rescue orforglipron or having bariatric surgery or other weight-loss procedures will be excluded.
Full Analysis Set for weight plateau (FAS2 - WP) – Tirzepatide or Semaglutide, respectively: This analysis set will be used to estimate the supplementary treatment-regimen estimand	Data obtained during Study Periods II from the mITT with BW Plateau – Tirzepatide or Semaglutide, respectively, for efficacy analyses population regardless of adherence to study treatment and regardless of initiation of other AOMs, GLP-1 RAs, GIP/GLP-1 RAs, or DPP-4 inhibitors, regardless of initiation of rescue orforglipron or having bariatric surgery or other weight-loss procedures.
Efficacy Analysis Set for weight plateau (EAS - WP) - Tirzepatide or Semaglutide, respectively: This analysis set will be used to estimate the efficacy estimand	Data obtained during Study Period II from the mITT Population with BW Plateau - Tirzepatide or Semaglutide, respectively, for efficacy analyses population, excluding data after permanent discontinuation of treatment, initiation of other AOMs, GLP-1 RAs, GIP/GLP-1 RAs, DPP-4 inhibitors, or rescue orforglipron, or having bariatric surgery or other weight-loss procedures.

Analysis Datasets	Description
Safety Analysis Set (SS) Before Rescue – Tirzepatide or Semaglutide, respectively: This analysis will be used to assess the safety of study treatment prior to the initiation of Rescue Orforglipron	Data obtained during Study Periods II, and III from the Safety Population– Tirzepatide or Semaglutide, respectively, regardless of adherence to study treatment and regardless of initiation of other AOMs, GLP-1 RAs, GIP/GLP-1 Ras, or DPP-4 inhibitor. Data obtained after rescue will be excluded.
Safety Analysis Set (SS) orforglipron - Tirzepatide or Semaglutide, respectively: This analysis will be used to assess the safety of orforglipron, including after participants require rescue	Data obtained during Study Periods II, and III from the Safety Population in both arms while exposed to orforglipron: Tirzepatide or Semaglutide, respectively, regardless of adherence to study treatment and regardless of initiation of other AOMs, GLP-1 RAs, GIP/GLP RAs, or DPP-4 inhibitor. Data for participants randomized to orforglipron will be included after first dose and data for participants randomized to placebo will be included after initiation of rescue orforglipron.
Safety Analysis Set (SS) Before Rescue and initiation of AOMs– Tirzepatide or Semaglutide, respectively: This analysis will be used to assess the safety of study treatment	Data obtained during Study Periods II, and III from the Safety Population– Tirzepatide or Semaglutide, respectively, regardless of adherence to study treatment. Data obtained after rescue and initiation of other AOMs, GLP-1 RAs, GIP/GLP-1 RAs, or DPP-4 inhibitor will be excluded.
Safety Analysis Set (SS) Before Week 24 (Visit 8)– Tirzepatide or Semaglutide, respectively: This analysis will be used to assess the safety of study treatment	Data obtained during Study Periods II, and III from the Safety Population– Tirzepatide or Semaglutide, respectively, regardless of adherence to study treatment and regardless of initiation of other AOMs, GLP-1 RAs, GIP/GLP Ras, or DPP-4 inhibitor, data obtained after Week 24 (Visit 8) will be excluded.

4. Statistical Analyses

4.1. General Considerations

Statistical analysis of this study will be the responsibility of Lilly or its designee. The statistical analyses will be performed using R 4.3.2 or higher.

Any change to the data analysis methods described in the protocol will require an amendment ONLY if it changes a principal feature of the protocol. Any other change to the data analysis methods described in the protocol, and the justification for making the change, will be described in the SAP or Clinical Study Report (CSR). Some analyses and summaries described in this analysis plan may not be conducted if not warranted by data (for example, few events to justify conducting an analysis). Listings of events will be provided in such situations. Additional exploratory analyses of the data will be conducted as deemed appropriate without further changes made to the protocol or SAP, even after final database lock.

All tests of treatment effects will be conducted at a 2-sided alpha level of 0.05, unless otherwise stated, and all confidence intervals will be given at a 2-sided (95%) level. In statistical summaries and analyses, all data will be analyzed by randomized treatment assignment and will be performed separately for each treatment in the SURMOUNT-5 study. Participants will be analyzed according to the treatment they were randomly assigned to, regardless of the treatment actually received.

For each treatment in the SURMOUNT-5 study, efficacy analyses will use the full analysis set (FAS or FAS - WP) to evaluate the modified treatment-regimen estimand, and the efficacy analysis (EAS or EAS - WP) set to evaluate the efficacy estimand. Safety analyses will be assessed using the Safety Analysis Set (SS) Before Rescue. Selected safety analyses may be conducted in the Safety Analysis Set (SS) Before Rescue and initiation of AOMs, Safety Analysis Set (SS) orforglipron Only and/or in the Safety Analysis Set (SS) Before Week 24 (Visit 8).

End of study participation for a participant will be the earliest of date of death, date of withdrawal from further participation in the study, and date of last study visit. For patients considered to be lost to follow-up, end of study participation will be the date of lost to follow-up reported by the investigator. Patient data included in the database after the last date of study participation (date of death, date of ED or date of last study visit) will be excluded from statistical analysis. Listings of such data may be provided.

A participant is considered to have completed the study if the participant has completed all periods of the study including the last visit or the last scheduled procedure shown in the SoA.

For continuous measures, summary statistics may include sample size, mean, SD, median, minimum, and maximum for the actual value, the change from baseline, and if applicable, the percent change from baseline measurements as well. Model-based estimated means and standard errors derived from the analysis models will also be displayed for the actual value, and the change or percent change from baseline measurements. For analysis of log-transformed parameters, model estimated means and standard errors on the original scale will be derived

through back-transformation using the delta method from the model-based estimated means and standard errors on the natural log-scale. Treatment comparisons will be displayed showing the model-based estimated means, the 95% CIs, and the p-values for the treatment differences. All baseline measures will be analyzed using an analysis of variance (ANOVA) model that has treatment group as the fixed effect.

For categorical measures, summary statistics will include sample size, frequency, and percentages. For safety categorical measures, risk difference and its 95% CI will be provided wherever applicable. If applicable, Fisher's exact test or Pearson's chi-squared test will be used for treatment comparisons.

For time-to-event measures, participants without an event will be censored, and time-to-event will be the number of days between the start date and the date of the participant's end of follow-up + 1 day (depending on the estimand definition). For participants experiencing the event, "time-to-first-event" will be the time (in days) from start date to the first occurrence of the event + 1 day. For safety and study intervention discontinuation analyses, the date of first dose of study intervention will be used as the start date. For other analyses (e.g. study discontinuation), the randomization date will be used as the start date (in rare cases where randomization occurred prior to Visit 2 date, Visit 2 date will be used instead).

Not all analyses described in this SAP will necessarily be included in the Clinical Study Report (CSR). Any analysis described in this SAP and not provided in the CSR would be available upon request.

4.1.1. Baseline Definition

Unless otherwise specified, the baseline for efficacy assessments is defined as the last available non-missing measurement prior to or at the first dose of study intervention; in most cases, this will be the measurement recorded at Week 0 (Visit 2). If there are no doses of study intervention administered, the baseline will be defined as the last available non-missing measurement on or prior to randomization. In cases where the measurement is taken on the same day (where the time is not collected or not reliable) as the first dose, this measurement will be used as the baseline value for data analysis. For patient-reported outcome measures data obtained at Visit 2, regardless of the timing relative to first dose, will serve as the baseline.

For safety analyses, the definition of baseline and postbaseline can be found in Table GZPN.4.1.

Table GZPN.4.1. Baseline and Postbaseline Definitions for Safety Outcomes

Analysis Set	Analysis Type	Baseline Period	Postbaseline Period
SS – Before Rescue<br>SS – Prior to Rescue and AOM	Treatment-Emergent Adverse Events	Starts from informed consent date and ends prior to the first dose (typically at Week 0).	Starts after the first dose of study intervention and ends at the earliest of end of the follow-up period, or the last date defined in each safety analysis set (i.e. earliest of day prior to rescue start date or prohibited medication start date, or the end of the follow-up period or the date of study withdrawal).
SS – Before Rescue<br>SS – Prior to Rescue and AOM	Treatment-Emergent Abnormal Labs, and Vital Signs.	Starts from informed consent date and ends prior to the first dose (typically at Week 0).<br>All scheduled and unscheduled measurements will be included.	Starts after the first dose and ends at the earliest of end of the follow-up period or the last date defined in each safety analysis set.<br>All scheduled and unscheduled measurements will be included.
SS – Before Rescue<br>SS – Prior to Rescue and AOM	Change from baseline to each postbaseline week and topostbaseline for laboratory values, vital signs	Starts from informed consent date and ends prior to the first dose (typically at Week 0).	Starts after the first dose and ends at the earliest of end of the follow-up period or the last date defined in each safety analysis set.<br>Only scheduled visits and early termination visits that fall on the scheduled visits will be included.

Analysis Set	Analysis Type	Baseline Period	Postbaseline Period
SS - orforglipron	Treatment-Emergent Adverse Events	Starts from informed consent date and ends prior to the first dose of orforglipron<br>An AE would be considered as baseline AE if 1) started prior to first dose of drug and was ongoing during the baseline period or 2) started after first dose of PBO drug and was still ongoing at the time of initiation of rescue. Note: for PBO arm, AEs that start after the first dose of PBO and ends prior to the first dose of OFG will not be considered as baseline AEs	For OFG arm, starts after the first dose and ends at the end of the follow-up period or the date of study withdrawal.<br>For PBO arm, starts after the first dose of Rescue and ends at the end of the follow-up period or the date of study withdrawal.<br>All scheduled and unscheduled measurements will be included.
SS - orforglipron	Treatment-Emergent Abnormal Labs, and Vital Signs..	Starts from informed consent date and ends prior to the first dose of orforglipron.<br>Note: for PBO arm, abnormal values between the first dose of PBO and the first dose of OFG will not be considered as baseline abnormality	For OFG arm, starts after the first dose and ends at the end of the follow-up period or the date of study withdrawal.<br>For PBO arm, starts after the first dose of Rescue and ends at the end of the follow-up period or the date of study withdrawal.

4.1.2. Analysis Methods

The analysis methods are consistent with the desired estimands and the FDA guidance on "Adjusting for Covariates in Randomized Clinical Trials for Drugs and Biological Products Guidance Document" (FDA 2023).

4.1.2.1. Analysis Methods for Modified Treatment Regimen Estimand

4.1.2.1.1. Method for Continuous Variables

The ANCOVA model will be used to analyze continuous measurements at Week 24 or Week 52 and will be guided by the modified treatment regimen estimand. A single strata variable will be constructed with 6 joint levels of, including whether achieving plateau at Week 72 of SURMOUNT-5 study (yes/no), sex (individual AFAB, individual AMAB), and percent weight loss at Week 72 of SURMOUNT-5 study (<20% versus ≥20%). Note, this strata variable will only have 4 joint levels in FAS - WP and FAS2 - WP. The model will include

- treatment group as a factor variable
- strata as a factor variable

- baseline value (usually of the dependent variable)
- interaction between strata variable and treatment group, and
- interaction between baseline value and treatment group.

Unless otherwise specified, baseline value is the baseline of the dependent variable. The estimated treatment group effect and comparison between orforglipron 36 mg or MTD (24 mg or 36 mg) versus placebo will be reported together with variability estimated using the robust inference (Ye et al. 2022; FDA 2023). The associated 2-sided 95% confidence interval and corresponding p-values will also be reported. If the model fails to converge, all the interaction terms will be removed before the model fitting. The addition of interaction terms is not intended to estimate the heterogeneity effect but to provide robustness and efficiency for the estimate of treatment comparisons on the unconditional effect. The final inference will be derived using Rubin's Rule which combines estimates from multiple imputed datasets.

Some parameters may be log-transformed before fitting the ANCOVA as specified in Sections 4.4 through 4.6, with the associated log-transformed baseline value as a covariate. In these cases, model-based estimated means and 95% CIs for each treatment group and treatment difference will be back-transformed and presented as mean percent change from baseline and as relative treatment difference to placebo treatment groups in percent change.

4.1.2.1.2. Method for Binary Variables

The binary outcomes will be analysed with the following procedure:

- For modified treatment estimand, use the same imputation strategy as in Section 4.1.2.1.3 on the underlying continuous endpoint that determines the binary value.
- Transform the observed and imputed continuous value to the binary value, for example, convert the continuous body weight values at Week 52 visit to whether the percent maintenance or the corresponding percent change from baseline meets a certain threshold (Ma et al. 2022).
- Fit a logistic regression model to the data with the following terms:
  - treatment group as a factor variable
  - strata as a factor variable
  - baseline value (usually of the dependent variable)
  - interaction between treatment group and strata, and
  - interaction between treatment group and baseline value.
- Provide estimates and inferences of unconditional treatment effects defined by the risk difference and/or relative risk based on the delta-method using the formula provided (Ye et al. 2023).
- Derive the final inference using Rubin's rule which combines estimates from multiple imputed datasets.

The statistical testing for risk differences will be used as the primary approach for treatment comparisons.

4.1.2.1.3. Multiple Imputation Strategy for Modified Treatment Regimen Estimand

Participants who discontinue study intervention (that is, discontinue study treatment) will be encouraged to continue in the study for the treatment period and follow-up period (note, the case report forms [CRFs] use “phase”, which is interchangeable with the “period” in SAP). In this section, study discontinuation refers to treatment phase discontinuation captured in CRF.

The imputation procedure for creating a single imputed dataset under modified treatment regimen is detailed in Table GZPN.4.2.

In principle, missing data at Week 52 for participants who take rescue orforglipron (Scenario 3) will be imputed with their worst measurement taken after randomization and up to the rescue start date (WOCF). Missing data due to permanent discontinuation of the study intervention (Scenario 5) will be imputed by treatment group using retrieved dropouts (MI-RD), namely using multiple imputation based on data retrieved from participants who permanently discontinued the study intervention but continued in the study with non-missing measurements from the same treatment group (Scenario 4). If there are not enough retrieved dropouts to provide a reliable imputation model (that is, the model implemented does not converge), data after WOCF imputation from participants in Scenario 3 will be combined with retrieved dropout participants to impute.

Table GZPN.4.2. Imputation Procedure

Scenario<br>(Study Intervention/Treatment Period Discontinuation; Missingness Status at Endpoint)	Methods to Handle Missing Values at Endpoint
1. No study intervention discontinuation; no initiation of rescue; no missing value	N/A
2. No study intervention discontinuation; no initiation of rescue; with missing value	Use observed data, including baseline and all postbaseline visits, across all time points from Scenario 1 and 2 to impute under the MAR assumption by treatment group. There should be very few such cases in a clinical trial. No additional covariates will be included in the imputation model.
3. Initiate rescue orforglipron; regardless of with or without missing value	Endpoint visit will be imputed with the worst value occurred at or prior to the first dose date of rescue.
4. Study intervention discontinuation prior to start of rescue; no study discontinuation; no missing value	N/A
5. Study discontinuation resulting in study intervention discontinuation prior to start of rescue orforglipron; with missing value	Missing values at the endpoint visit will be imputed by treatment through MCMC (predictive mean matching method), using the baseline and the endpoint visit data from Scenario 4 (MI-RD). If there are not enough participants in Scenario 4 for model convergence, then participants from Scenario 3 will be added for imputation

Abbreviations: MAR = missing at random; N/A = not applicable.

4.1.2.1.4. Multiple imputation Strategy for Supplementary Treatment Regimen Estimand

For Supplementary Treatment Regimen Estimand, data obtained after rescue orforglipron or having bariatric surgery or other weight-loss procedures will not be excluded (FAS2 and FAS2 – WP). All missing data at primary time point will be imputed by treatment group using retrieved dropouts (MI-RD), namely using multiple imputation based on data retrieved from participants who permanently discontinued the study intervention but continued in the study with non-missing measurements from the same treatment group, if there are not enough retrieved dropouts to provide a reliable imputation model, an alternative multiple imputation method (Placebo Washout) will be used.

4.1.2.2. Analysis Methods for Efficacy Estimand

4.1.2.2.1. Method for Continuous Variables

For each treatment in the SURMOUNT-5 study, the primary objective based on the efficacy estimand defined in Section 3 will be evaluated using the corresponding EAS or EAS - WP dataset. Missing data at after the start of rescue orforglipron will be imputed using WOCF. No additional imputation will be performed.

For the assessment of continuous efficacy measures guided by the efficacy estimand, a maximum likelihood-based MMRM (Wang and Du 2024) analysis will be used. All the longitudinal

observations at each scheduled postbaseline visit will be included in the analysis. The MMRM model will include the following terms:

- treatment group as a factor variable
- visit as a factor variable
- strata as a factor variable
- baseline value (usually of the dependent variable)
- interaction between treatment group, strata variable and visit, and
- interaction between treatment group, baseline value and visit.

The estimated treatment group effect and comparison between orforglipron 36 mg or MTD (24 mg or 36 mg) and placebo at the scheduled visits will be reported together with the variability estimated using the robust inference as provided in Wang and Du (2024). The sandwich estimator (Diggle et al. 1994) for the variance-covariance matrix will be used. The addition of 3-way interaction terms is not intended to estimate the heterogeneity effect but to provide robustness and efficiency for the estimate of treatment comparisons on the unconditional effect. The associated 2-sided 95% confidence interval and corresponding p-values will also be reported. An unstructured covariance matrix by each treatment group will be used to model the within-participant errors, assuming heteroscedasticity and the measurements for different participants are independent. If the model fails to converge, the model will be simplified to include the following terms, removing 3-way interactions and the heteroscedasticity assumption:

- treatment group as a factor variable
- visit as a factor variable
- strata as a factor variable
- baseline value (of the dependent variable)
- interaction between treatment group and visit
- interaction between strata variable and visit, and
- interaction between baseline value and visit.

If the model still fails to converge, the following covariance structures will be tested in order for the simplified model:

- heterogeneous Toeplitz
- heterogeneous autoregressive
- heterogeneous compound symmetry
- homogeneous Toeplitz
- homogeneous autoregressive, and
- homogeneous compound symmetry.

The first covariance structure that converges will be used.

For some variables, both the postbaseline response variables and baseline variable will be log-transformed before fitting the MMRM. The treatment group estimates will be the percent change from baseline while the treatment contrasts will be the relative change in orforglipron 36 mg or MTD (24 mg or 36 mg) compared to the placebo (%) over the scheduled visits.

4.1.2.2.2. Method for Binary Variables

The binary outcomes will be analysed with the following procedure:

- For efficacy estimand, the underlying continuous endpoint that determines the binary value will be imputed by first imputing the data at final timepoint collected after rescue using the worst value prior to or at the day of rescue, then the remaining missing value will be imputed through multiple imputation using all non-missing data (including data imputed by worst value) from the same treatment group under the missing at random (MAR) assumption.
- At the visit of interest, transform the observed and imputed continuous value to the binary value, for example, convert the continuous body weight values to whether the percent maintenance or the corresponding percent change from baseline meets a certain threshold.
- Fit a logistic regression model to the data with the following terms:
    - treatment group as a factor variable
    - strata as a factor variable
    - baseline value (of the dependent variable)
    - interaction between treatment group and strata, and
    - interaction between treatment group and baseline value.
- Provide for the visit of interest the estimates and inferences of unconditional treatment effects defined by the risk difference and/or relative risk based on the delta-method using the formula provided (Ye et al. 2023).
- Derive the final inference using Rubin's rule which combines estimates from multiple imputed datasets.

The statistical testing for risk difference will be used as the primary approach for treatment comparisons.

4.1.2.3. Analysis Methods for Safety

For categorical safety measures, treatment group differences of percentages will be conducted using Fisher's exact test or Pearson's chi-squared test if applicable, unless otherwise specified. Risk difference and the 95% CI will be provided.

For selected continuous safety measures, unless otherwise specified, treatment group differences of mean change or percent mean change from baseline at all scheduled visits will be assessed via an MMRM using maximum likelihood, which will include the following terms:

- treatment group as a factor variable
- visit as a factor variable
- baseline value (of the dependent variable)
- interaction between visit and treatment group

If the data does not warrant the MMRM model, then an ANCOVA model with treatment group as a fixed effect and the continuous baseline value as a covariate will be used.

No explicit imputation will be conducted for safety measures. Some parameters (such as urinary albumin to creatinine ratio [UACR], p-amylase, and lipase) may be log-transformed before fitting the MMRM as specified in Section 4.1.2.3. In these cases, model-based estimated means and 95% confidence intervals for each treatment group and treatment difference will be back-transformed and presented as mean percent change from baseline and as relative treatment difference of orforglipron treatment groups compared to placebo in percent change.

The Kaplan–Meier (KM) product limit method will be used to estimate the cumulative event-free survival rates over time for the time-to-event analyses. An unstratified Cox proportional hazards regression analysis will be used to compare hazard rates among treatments. An unstratified log-rank test will be used to calculate p-values. Time-to-event for the specific safety event of interest will be calculated from the date of first dose.

A logistic regression model will be used for hypoglycemia incidence for treatment comparisons for the overall postbaseline period. The model will include the fixed effects of treatment. Given the expected small number of hypoglycemic events in the study population, the rate of events will be analyzed using an empirical method for the overall postbaseline period. If there are no observed hypoglycemia events, no analysis will be conducted.

4.2. Participant Dispositions

The participant dispositions for the screening period, study intervention, the treatment period, and/or the follow-up period will be collected in the CRFs with the corresponding primary reason.

The study completion status is defined as at the end of the 52-week treatment period (when the primary endpoint is ascertained and the primary database is locked): completers will be considered as those who complete the treatment period (Week 52) and the follow-up visit, otherwise, participants will be considered as non-completers.

The planned listings and summary tables for dispositions are provided in Table GZPN.4.3. No inferential analysis will be performed.

Table GZPN.4.3. Listings and Summary Tables Related to Dispositions

Analysis	Population/Period
Summary of disposition (prior to randomization)	Entered participants
Patient allocation by region, country, and center/site	Entered participants
Summary of study and study intervention disposition	Randomized participants/TP + FP (TP for study treatment disposition)
Kaplan-Meier plot of time to study discontinuation	Randomized participants/TP + FP
Kaplan-Meier plot of time to study intervention discontinuation	mITT participants/TP
Kaplan-Meier plot of time to study intervention discontinuation due to AEs	mITT participants/TP
Listing of randomization	Randomized participants
Listing of randomized participants who were discontinued from the study intervention due to inadvertent enrollment	Randomized participants
Listing of study and study intervention disposition	Randomized participants

Abbreviations: FP = follow-up period; TP = treatment period.

4.3. Primary Endpoints/Estimands Analysis

4.3.1. Definition of Endpoint

For each treatment in the SURMOUNT-5 study (tirzepatide or semaglutide), the primary endpoint for this study is percent maintenance of BW reduction achieved during the 72 weeks of treatment with tirzepatide or semaglutide at Week 52, respectively, for those who have achieved a BW plateau. This endpoint will be used to evaluate the primary objective of the study for the modified treatment-regimen estimand and the efficacy estimand, as well as the supplementary estimands. (Section 1.1). This endpoint is defined as

$$100 * \frac{BW - SM5\ Baseline\ BW}{Baseline\ BW - SM5\ Baseline\ BW}$$

4.3.2. Main Analytical Approach

For each treatment in the SURMOUNT-5 study, the analytical approaches are specified for the modified treatment regimen estimand in Section 4.1.2.1.1 (ANCOVA) and the efficacy estimand in Section 4.1.2.2.1 (MMRM). SURMOUNT-5 baseline weight will be used as the baseline covariate. Orforglipron 36 mg or MTD will be declared superior to placebo in percent maintenance of BW reduction if the p-value is less than the alpha level allocated to the hypothesis according to the graphical approach (Section 2.1).

4.3.3. Supplementary Analyses

The supplemental analysis is specified in Section 4.1.2.1.4 (Supplementary Treatment-regimen Estimand).

4.4. Secondary Endpoints/Estimands Analysis

4.4.1. Key Secondary Endpoints

The key secondary study objectives are listed in Section 1.1.

4.4.1.1. Definition of Endpoints

The definition of the endpoints are:

- percent change in BW from SURMOUNT-5 baseline prior to the initiation of tirzepatide or semaglutide treatment at Week 52
- percent maintenance of BW reduction achieved during the 72 weeks of treatment with tirzepatide or semaglutide at Week 52 (defined in Section 4.3.1)
- incidence of participants maintaining ≥80% of the BW reduction achieved during 72 weeks of tirzepatide or semaglutide treatment at Week 52

4.4.1.2. Main Analytical Approach

For each treatment in the SURMOUNT-5 study, the analytical approaches are specified for the modified treatment regimen estimand in Section 4.1.2.1.1 (ANCOVA) and the efficacy estimand in Section 4.1.2.2.1 (MMRM). SURMOUNT-5 baseline weight will be used as the baseline covariate for percent maintenance of BW reduction analysis.

For each hypothesis, orforglipron 36 mg or MTD will be declared superior to placebo if the p-value is less than the alpha level allocated to the hypothesis according to the graphical approach (Section 2.1).

4.4.2. Additional Secondary Endpoint

The following additional secondary efficacy endpoints will be guided by the efficacy estimand (Section 4.1.2.2) and will be evaluated using the EAS-WP analysis set.:

- change from randomization in BW (kg) at Week 52
- percent change from randomization in BW at Week 52
- change from randomization in waist circumference (cm) at Week 52
- percent maintenance of BW reduction achieved during the 72 weeks of treatment with tirzepatide or semaglutide in SURMOUNT-5 at Week 24
- incidence of participants maintaining ≥15% BW reduction from SURMOUNT-5 baseline for participants who have already lost ≥15% BW after 72-week of tirzepatide or semaglutide treatment at Week 52

4.5. Exploratory Endpoints Analysis

4.5.1. Exploratory Analysis Specified in the Protocol

The following exploratory endpoints will be guided by the efficacy estimand (Section 4.1.2.2) and will be evaluated using the EAS-WP analysis set

Measure	Relative to the efficacy measure:	Analysis Conducted	Additional Information
Lipid parameters	• total cholesterol (mg/dL),<br>• HDL-cholesterol (mg/dL)<br>• LDL-cholesterol (mg/dL)<br>• VLDL-cholesterol (mg/dL)<br>• non-HDL cholesterol (mg/dL)<br>• triglycerides (mg/dL)	MMRM specified in Section 4.1.2.2.1	Data will be log-transformed prior to fitting the MMRM model.
blood pressure parameters	systolic blood pressure (mmHg).	MMRM specified in Section 4.1.2.2.1	
glycemic parameters	fasting glucose (mg/dL)<br>fasting insulin (pmol/L)<br>HbA1c (%)	MMRM specified in Section 4.1.2.2.1	Data will be log-transformed for fasting insulin prior to fitting the MMRM model.
rescue AOM use	Use of rescue orforglipron (yes/no) for weight regain ≥50% of the BW reduction achieved with tirzepatide or semaglutide treatment	Logistic model in Section 4.1.2.2.2	
BW endpoints	Change in BMI (kg/m^2)	MMRM specified in Section 4.1.2.2.1	
	Assessment (yes/no) of maintenance of BW reduction ≥20% BW reduction from SURMOUNT-5 baseline for participants who have already lost ≥20% with tirzepatide or semaglutide treatment	MMRM specified in Section 4.1.2.2.1	
Patient-reported outcomes	• SF-36v2 acute form domain and summary scores<br>• CoEQ scores<br>• rPDQS score	MMRM specified in Section 4.1.2.2.1	
	• Proportion of participants with improved categorical shift in:<br>○ PGIS-Physical Function Weight<br>○ PGIS-Food Craving<br>• Proportion of participants with improvements in:<br>○ PGIC-Physical Function Weight<br>○ PGIC-Food Craving	For each question, the proportion of participants with improvements from baseline will be summarized. Shift analysis from baseline to Week 52 will also be performed	

Abbreviations: AE = adverse event; BMI = body mass index; BW = body weight; CoEQ = Control of Eating Questionnaire-NRS; HbA1c = hemoglobin A1c; HDL = high-density lipoprotein; LDL = low-density lipoprotein; MTD = maximum tolerated dose; PGIC = Patient Global Impression of Change; PGIS = Patient Global Impression of Severity; rPDQS = rapid Prime Diet Quality Score; SAE = serious adverse event; SF-36v2 = Short Form-36 version 2 Health Survey acute form; SURMOUNT-5 = Study I8F-MC-GPHJ (GPHJ); TEAE = treatment-emergent adverse event; VLDL = very-low-density lipoprotein.

[a] BW plateau is defined as <5% BW change between Visit 17 (Week 60) and Visit 20 (Week 72) of SURMOUNT-5 study.

4.5.2. Other Exploratory Analysis Not Specified in Protocol

Additional sensitivity analysis will be conducted using Efficacy Analysis Set for weight plateau (EAS-WP), excluding data after prolonged dose interruption for mean percent maintenance of BW reduction achieved during the 72 weeks of treatment with tirzepatide or semaglutide under Efficacy Estimand . Prolonged dose interruption is defined as Dose Interruption >= 30 days. Analysis methods will follow Section 4.1.2.2.1.

Another sensitivity analysis will be conducted using Efficacy Analysis Set (EAS), excluding data after prolonged dose interruption for mean percent change in BW from prior to the initiation of tirzepatide or semaglutide, respectively, of orforglipron 36 mg or MTD [24 mg or 36 mg] compared with the placebo at Week 52 under Efficacy Estimand. Analysis methods will follow Section 4.1.2.2.1. In addition, below analyses will be performed.

**Measure**	**Relative to the efficacy measure:**	**Analysis Conducted**	**Additional Information**
To compare orforglipron 36 mg or MTD (24 mg or 36 mg) to placebo at Week 52 in participants who have reached a BW plateau[a] for BW endpoints	Assessment (yes/no) of continuing to lose weight after 72 weeks of tirzepatide or semaglutide treatment from randomization	Logistic regression specified in Section 4.1.2.2.2	
To compare orforglipron 36 mg or MTD (24 mg or 36 mg) to placebo for delaying progression to prediabetes or T2D at 52 weeks in all participants.	Time to onset of prediabetes or T2D	Analysis method specified in Section 4.1.2.3	
To compare orforglipron 36 mg or MTD (24 mg or 36 mg) to placebo at Week 52 in participants who have reached a BW plateaua for BW endpoints	Proportion of participants achieving body weight change thresholds values after randomization<br>• <5%	Logistic regression specified in Section 4.1.2.2.2	

[a] BW plateau is defined as <5% BW change between Visit 17 (Week 60) and Visit 20 (Week 72) of SURMOUNT-5 study; T2D = type 2 diabetes.

4.6. Safety Analyses

The planned safety analyses are consistent with compound level safety standards, which are based on various sources, including company standards, internal and external subject matter experts, publications from cross-industry initiatives (for example, PHUSE 2013, 2015, 2017, 2018, 2022), and publications from regulatory agencies (for example, EMA 2014, CDER/BIRSS 2022, 2023). Descriptions of the safety analyses are provided in this SAP, but some details are found in the compound level safety standards.

For each treatment in the SURMOUNT-5 study, safety assessments will be conducted using the Safety Analysis Set (SS) Before Rescue. Selected safety analyses may be conducted with Safety Analysis Set (SS) Before Rescue and initiation of AOMs, the Safety Analysis Set (SS) Orforglipron Only and/or Safety Analysis Set (SS) Before Week 24 (Visit 8). Only descriptive statistics (that is, no treatment comparisons) will be provided for the Safety Analysis Set (SS) Orforglipron Only, since all participants will be taking orforglipron.

AEs will be coded from the actual term using the Medical Dictionary for Regulatory Activities and reported with preferred terms and system organ class. Selected notable AEs of interest may be reported using high-level terms or Standardized Medical Dictionary for Regulatory Activities Queries.

The analytical approaches for safety analyses are specified in Section 4.1.2.3. Summary tables with risk difference will be sorted by decreasing order in risk difference.Not all displays will necessarily be created as a “static” display. Some may be incorporated into interactive display tools instead of or in addition to a static display.

4.6.1. Extent of Exposure

Duration of exposure to study treatment will be summarized by treatment group for safety participants under Safety Analysis Set (SS) Before Rescue. Descriptive statistics for participant weeks (or days) of exposure and total participant years in exposure will be provided. Overall exposure will be summarized in total participant-year (PY) of exposure, derived in the following manner:

total PY of exposure = sum of duration of exposure in days (for all participants in treatment group) / 365.25

In addition, the frequency and percentages of participants falling into the following exposure ranges for study and study intervention may be summarized by planned treatment group:

- 0 weeks
- >0 to <4 weeks
- ≥4 to <8 weeks
- ≥8 to <12 weeks

- ≥12 to <16 weeks
- ≥16 to <20 weeks
- ≥20 to <24 weeks
- ≥24 to <32 weeks
- ≥32 to <40 weeks
- ≥40 to <52 weeks, and
- ≥52 weeks.

Participants with dose interruptions/modifications will be summarized with reasons by treatment group.

No p-values will be reported in these summaries as they are intended to describe the study populations rather than test hypotheses about them.

4.6.2. Adverse Events

The planned summaries for AEs are provided in Table GZPN.4.4 and are described more fully in compound-level safety standards.

Table GZPN.4.4. Summary Tables Related to Adverse Events

**Analysis**	**Method**	**Additional analysis sets other than SS Before Rescue**
Overview of AEs, including<br>• TEAE<br>• SAE<br>• death, and<br>• permanent discontinuation from study intervention due to an AE.	Fisher's exact	SS Orforglipron SS Prior to Rescue and Initiation of AOMs for SAE<br>SS Before Week 24 (Visit 8) for SAE
TEAEs by PT within SOC	Fisher's exact	SS Orforglipron<br>SS Before Week 24 (Visit 8)
TEAEs by PT	Fisher's exact	SS Orforglipron
Maximum Severity TEAEs by PT within SOC		SS Orforglipron
TEAEs with incidence ≥5% by PT	Fisher's exact	SS Orforglipron
SAEs by PT within SOC	Fisher's exact	SS Orforglipron,<br>SS Prior to Rescue and Initiation of AOMs for SAE
Primary AEs leading to permanent discontinuation of study intervention by PT within SOC	Fisher's exact for SS Before Rescue	SS Orforglipron
Primary AEs leading to permanent discontinuation of study by PT within SOC	Fisher's exact for SS Before Rescue	SS Orforglipron
AEs leading to study intervention interruption by PT within SOC	Fisher's exact for SS Before Rescue	SS Orforglipron
AEs leading to study intervention reduction by PT within SOC	Fisher's exact for SS Before Rescue	SS Orforglipron
Listing of SAEs		SS Orforglipron
Listing of primary AEs leading to permanent discontinuation of study intervention		SS Orforglipron
Listing of primary AEs leading to permanent discontinuation of study		SS Orforglipron
Listing of deaths		
Listing AEs related to suspected overdosing		SS Orforglipron
Listing of participants with at least 1 notable event		

Abbreviations: AE =Adverse Event; FP = follow-up period; TP = treatment period; TEAE = Treatment Emergent Adverse Event; SAE = Serious Adverse Event; PT = Preferred Term; SOC = System Organ Class.

4.6.3. Special Safety Topics

This section includes safety topics of interest whether due to observed safety findings, potential findings based on drug class, or safety topics anticipated to be requested by a regulatory agency for any reason. In general, safety topics of interest will be identified by 1 or more standardized Medical Dictionary for Regulatory Activities (MedDRA) query(ies) (SMQs), system organ class (SOC), high-level term, FDA Medical Query, or by a Lilly defined MedDRA Preferred Term (PT) listing based upon the review of the most current MedDRA version, or by relevant laboratory changes. Searching criteria are detailed in the compound level safety standards.

The planned analyses for safety topics of interest are provided in Table GZPN.4.5 and are described more fully in the compound level safety standards.

Table GZPN.4.5. Description and Analyses of Safety Topics of Interest

Special Safety Topic	Short Description	Analysis	Analysis Set	Method
Major Adverse Cardiovascular Events	Death and nonfatal CV AEs will be adjudicated by a committee of physicians external to Lilly with cardiology expertise: Clinical Endpoint Committee (CEC).	Positively adjudicated MACE by category/subcategory and PT (if necessary)<br>MACE reported by investigators may be summarized	SS before rescue	
		Listing of all MACE reported by investigator (whether or not positively adjudicated)	SS before rescue	
Arrhythmias and Cardiac Conduction Disorders	The treatment-emergent (TE) arrhythmias and cardiac conduction disorders events will be derived using the MedDRA PTs contained in certain SMQs.	TE arrhythmias and cardiac conduction disorders by PT nested within SMQ and HLT[a]	SS before rescue	Fisher's exact
Hypotension, Orthostatic Hypotension, and Syncope	The TE hypotension, orthostatic hypotension, and syncope events will be derived using MedDRA PTs.	TE hypotension, orthostatic hypotension, and syncope by PT[a]	SS before rescue	Fisher's exact

Special Safety Topic	Short Description	Analysis	Analysis Set	Method
Hypoglycemia[b]	The 2023 American Diabetes Association position statement on glycemic targets (El Sayad et al. 2023) will be used to define:<br>• Level 1 hypoglycemia<br>• Level 2 hypoglycemia<br>• Level 3 hypoglycemia (severe/serious hypoglycemia)<br>Nocturnal hypoglycemia events (including severe hypoglycemia) **occur at night** and presumably during sleep.	Incidence (percent of patients experiencing 1 or more episode) of level 2 or level 3 hypoglycemia<br><br>Rate (episodes/patient/year) of level 2 or level 3 hypoglycemia	SS Before Rescue	Empirical method for the overall postbaseline period
		Listing of level 2 or level 3 hypoglycemia events	SS Before Rescue	
Gastrointestinal (GI) Adverse Events[c]	GI AEs using Gastrointestinal disorders SOC and Abdominal Pain (narrow FMQ) will be captured.	Study intervention discontinuation due to TE gastrointestinal events	SS Before Rescue	Fisher's exact
			SS orforglipron	
		TE abdominal pain by FMQ narrow PT	SS Before Rescue	Fisher's exact
			SS orforglipron	
		Severe or serious TE gastrointestinal events by PT	SS Before Rescue	Fisher's exact
			SS orforglipron	
		TE nausea, vomiting, diarrhea, constipation, and NVD by maximum severity	SS Before Rescue	Fisher's exact
			SS orforglipron	
		Prevalence and incidence over time for TE nausea, vomiting, diarrhea, constipation, and NVD	SS Before Rescue SS	
			SS orforglipron	

Special Safety Topic	Short Description	Analysis	Analysis Set	Method
		Plot of time to the onset of TE nausea, vomiting, diarrhea, constipation, and NVD	SS Before Rescue	KM
			SS orforglipron	KM
		Plot of prevalence and incidence over time for TE nausea, vomiting, diarrhea, constipation, and NVD by maximum severity	SS Before Rescue	
			SS orforglipron	
Renal Safety	Laboratory measures related to renal safety will be analyzed.<br>Renal events including acute renal failure and chronic renal failure exacerbation will be captured using SMQs.<br>Dehydration events will be captured using SMQ.	Shift of min-to-min for eGFR estimated by the CKD-EPI Cystatin C equation	SS Before Rescue	
		Shift of max-to-max for UACR	SS Before Rescue	
		MMRM analyses for eGFR estimated by the CKD-EPI Cystatin C equation	SS Before Rescue	MMRM
		MMRM analyses for UACR (log transformation)	SS Before Rescue	MMRM
		TE renal events by PT nested within SMQ [a]	SS Before Rescue	Fisher's exact
		Listing of TE dehydration events by PT [a]	SS Before Rescue	
Pancreatitis	The pancreatic enzyme data (p-amylase and lipase) will be observed through laboratory testing.<br><br>All suspected cases of acute or chronic pancreatitis and AEs of severe or serious abdominal pain of unknown etiology will be sent for adjudication by an independent clinical endpoint committee.	Shift of maximum-to-maximum for pancreatic enzyme	SS Before Rescue	
			SS Before Rescue and Initiation of AOMs	
			SS orforglipron	
		MMRM analysis for pancreatic enzymes (p-amylase and lipase) with a log transformation (postbaseline/baseline)	SS Before Rescue	MMRM
		Investigator-reported events and subsequently confirmed adjudicated events, respectively	SS Before Rescue	Fisher's exact
			Safety Analysis Set (SS) Before Rescue	Fisher's exact
			SS orforglipron	
		TE Pancreatic events by "Acute pancreatitis" SMQ and "Chronic pancreatitis" PT	SS Before Rescue	
			SS Before Rescue and Initiation of AOMs	
			SS orforglipron	

Approved on 21 Nov 2025 GMT

Special Safety Topic	Short Description	Analysis	Analysis Set	Method
		Listing of adjudicated and investigator-reported pancreatic events	SS Before Rescue	
			SS Before Rescue and Initiation of AOMs	
			SS orforglipron	
Thyroid Malignancies and C-Cell Hyperplasia	TE thyroid malignancies and C-cell hyperplasia will be identified using MedDRA HLT and PT.<br>The purpose of calcitonin measurements is to assess the potential effect of orforglipron on thyroid C-cell function.	TE thyroid C-cell hyperplasia and malignancies by PT	SS Before Rescue	Fisher's exact
		Shift of maximum-to-maximum for calcitonin value in the thresholds	SS Before Rescue	
		Listing of participants who meet protocol defined discontinuation criteria based on calcitonin:<br>• with eGFR <60 mL/min/1.73 m^2 at baseline, serum calcitonin value ≥35 ng/L AND ≥50% increase from the baseline value[a]<br>• with eGFR ≥60 mL/min/1.73 m^2 at baseline, serum calcitonin value ≥20 and <35 ng/L AND ≥50% increase from the baseline value and ≥10% increase on retest[a]<br>with eGFR ≥60 mL/min/1.73 m^2 at baseline, serum calcitonin value<br>≥35 ng/L AND ≥50% increase from the baseline value[a]	SS Before Rescue	
		MMRM analysis for calcitonin (log transformation)	SS Before Rescue	
Malignancies	The malignancy events will be derived using the MedDRA PTs contained certain SMQs.	TE malignancy by PT nested within SMQ[a]	SS Before Rescue	Fisher's exact

Special Safety Topic	Short Description	Analysis	Analysis Set	Method
Hepatic Safety	Hepatic labs include ALT, AST, ALP, TBL, DBL, and GGT.<br><br>When criteria are met for hepatic evaluations, investigators will conduct close monitoring of hepatic symptoms and liver tests, perform a comprehensive evaluation for alternative causes of abnormal liver tests, and complete follow-up hepatic safety eCRFs.	Abnormal postbaseline categories for hepatic safety parameters: ALT, AST, ALP, TBL, DBL, and GGT	SS Before Rescue	
			SS Before Rescue and Initiation of AOMs	
			SS orforglipron	
		Treatment-emergent potentially drug-related hepatic disorders by PT nested within SMQ	SS Before Rescue	Fisher's exact
			SS Before Rescue and Initiation of AOMs	Fisher's exact
			SS orforglipron	
		Hepatocellular drug-induced liver injury screening plot (maximum TBL vs maximum ALT or AST)	SS Before Rescue	
			SS Before Rescue and Initiation of AOMs	
			SS orforglipron	
		Hepatocellular drug-induced liver injury screening table	SS Before Rescue	
			SS Before Rescue and Initiation of AOMs	
			SS orforglipron	
		Cholestatic drug-induced liver injury screening table	SS Before Rescue	
			SS Before Rescue and Initiation of AOMs	
			SS orforglipron	
		Cholestatic drug-induced liver injury screening plot (maximum TBL vs maximum ALP)	SS Before Rescue	
			SS Before Rescue and Initiation of AOMs	
			SS orforglipron	
		Listing of participants with ALT or AST ≥3X ULN	SS Before Rescue	
			SS Before Rescue and Initiation of AOMs	
			SS orforglipron	
		Listing of participants with ALP or TBL ≥2X ULN	SS Before Rescue	
			SS Before Rescue and Initiation of AOMs	
			SS orforglipron	
		Shift of maximum-to-maximum for ALT, AST, ALP, TBL	SS Before Rescue	
			SS Before Rescue and Initiation of AOMs	
			SS orforglipron	

Special Safety Topic	Short Description	Analysis	Analysis Set	Method
		Participant profiles for participants meeting criteria for a comprehensive hepatic evaluation (as defined in the protocol).	SS Before Rescue	
			SS Before Rescue and Initiation of AOMs	
			SS orforglipron	
		Hepatic Laboratory Parameters (ALT, AST, ALP, TBL, DBL, and GGT) with a log transformation (postbaseline/baseline)	SS Before Rescue	MMRM
Gallbladder and Biliary Tract Disorders	All events of TE gallbladder and biliary tract disorders will be identified by using certain SMQs.	TE gallbladder and biliary tract disorders by PT nested within SMQ [a]	SS Before Rescue	Fisher's exact
Hypersensitivity Reactions	All events of TE allergic reaction and hypersensitivities will be identified by using certain SMQs.	TE allergic reaction and hypersensitivities by PT nested within SMQ [a]	SS Before Rescue	Fisher's exact
Depression, Suicidal Ideation, and Behavior	AEs will be searched using MedDRA PTs that satisfy the search criteria.	TE major depressive disorder, suicidal ideation, or behavior events by PT nested within FMQ or SMQ [a]	SS Before Rescue	Fisher's exact
	Suicide-related thoughts and behaviors will be collected based on the C-SSRS.	Summary of C-SSRS categories and composite measures	SS Before Rescue	
		Listing of C-SSRS categories and composite measures		

Special Safety Topic	Short Description	Analysis	Analysis Set	Method
	Patient health questionnaire-9 (PHQ-9) will be collected to assesses the specific diagnostic symptoms that determine the presence of a clinical depressive disorder. The PHQ-9 total scores will be categorized as none (not depressed), mild, moderate, moderately severe, and severe	Shift of each baseline category (maximum value) versus each postbaseline category (maximum value)	SS Before Rescue	
		Summary of categories based on the maximum values during baseline and postbaseline:<br>• any increase in depression category (that is, worsening of depression)<br>• increase from Minimal to none or Mild depression to Moderate, Moderately severe, or Severe depression<br>increase from Mild or Moderate depression to Moderately severe or Severe depression	SS Before Rescue	
Abuse Potential	AEs will be searched using a modified abuse potential FMQ.	TE abuse potential events by PT [a]	SS Before Rescue	Fisher's exact
AEs possibly related to loss of lean muscle mass	AEs will be identified using MedDRA HLTs and PTs	TEAEs possibly related to loss of lean muscle mass by PT within Event Category	SS Before Rescue	Fisher's exact
Malnutrition	AEs will be identified using MedDRA HLTs and PTs	TE malnutrition by PT within Event Category	SS Before Rescue	Fisher's exact
Excessive weight loss	AEs will be identified using MedDRA PTs BMI <18.5 kg/m2 will be analyzed	Severe or serious TE excessive weight loss by PT or BMI $<18.5 kg/m^2$	SS Before Rescue	Fisher's exact
		Listing of participants with BMI $<18.5\ kg/m^2$	SS Before Rescue	

Abbreviations: AE = adverse event; ALP = serum alkaline phosphatase; ALT = alanine aminotransferase; AST = aspartate aminotransferase; BMI = body mass index; CEC = clinical endpoint committee; CKD-EPI = Chronic Kidney Disease Epidemiology; C-SSRS = Columbia-Suicide Severity rating scale; CV = cardiovascular; eCRF = electronic case report form; eGFR = estimated glomerular filtration rate; DBL = direct bilirubin; FMQ = FDA Medical Query; FP = follow-up period; GGT = gamma glutamyl transferase; GI = gastrointestinal; HLT = high-level term; MACE = major adverse cardiovascular event; MedDRA = Medical Dictionary for Regulatory Activities; MMRM = mixed model repeated measure; PT = Preferred Term; SMQ = standardized MedDRA query; SOC = system organ class; TEAE = treatment-emergent adverse event; TBL = total bilirubin; TP = treatment period; UACR = urinary albumin-to-creatinine ratio; vs = versus.

[a] For these tables, if the number of events is less than 10, a listing will be provided instead.
[b] Glucose values collected on the same date as an OGTT assessment are not included
[c] Additionally, all GI events will be analyzed.
Note: Listings and participant profiles may be provided through interactive display tools instead of a static display.

4.6.4. Clinical Laboratory Evaluation

The planned summaries for clinical laboratory evaluations are provided in Table GZPN.4.6 and are described more fully in compound-level safety standards.

Table GZPN.4.6. Summary Tables Related to Clinical Laboratory Evaluations

Analysis	Method	Analysis Set
Box plots and mean/SD (or 95% CI) for observed values by visit	Descriptive statistics	SS Before Rescue
Box plots and mean/SD (or 95% CI) for change from baseline values by visit	Descriptive statistics	SS Before Rescue
Summary for participants with elevated or low values meeting specified levels	Descriptive statistics	SS Before Rescue
Listing of abnormal laboratory findings		SS Before Rescue

Abbreviations: CI = confidence intervals; FP = follow-up period; SD = standard deviation; TP = treatment period.

4.6.5. Vital Signs and Physical Characteristics

Triplicate vital signs will be collected at each visit, thus the mean of these measurements will be used for the vital signs analyses. The planned summaries for vital signs (SBP, diastolic blood pressure, and pulse rate) are provided in Table GZPN.4.7, and are described more fully in the compound-level safety standards.

Table GZPN.4.7. Summary Tables Related to Vital Signs

Analysis	Method	Analysis Set
Box plots and mean/SD (or 95% CI) for observed values by visit	Descriptive statistics	SS Before Rescue
Box plots and mean/SD (or 95% CI) for change from baseline values by visit	Descriptive statistics	SS Before Rescue
Analysis of blood pressure and pulse rate for change from baseline	MMRM	SS Before Rescue
Summary for participants meeting specific blood pressure and pulse rate levels	Descriptive statistics	SS Before Rescue
Shift of maximum-to-maximum for pulse rate	Descriptive statistics	SS Before Rescue

Abbreviations: CI = confidence intervals; FP = follow-up period; MMRM = mixed model repeated measures; SD = standard deviation; TP = treatment period.

4.6.6. Electrocardiograms

A summary of adverse event reported by electrocardiograms (ECGs) will be provided.

4.7. Other Analyses

4.7.1. Health Outcomes

The patient-reported outcome questionnaires will be completed by the participants at baseline and postbaseline. Guided by the modified treatment-regimen estimand, the analysis will be

conducted using an ANCOVA model as described in Section 4.1.2.1.1 using full analysis set (FAS - WP). Due to difficulty applying WOCF imputation for many patient-reported outcomes, all missing values at Week 52, including for those that initiate rescue, will be imputed using the last observation carry forward (LOCF). PGIC (Physical Function Weight and Food Craving) will be summarized using the full analysis set (FAS - WP). PGIS (Physical Function Weight and Food Craving) will be summarized in shift table from baseline to postbaselineat at each postbaseline visit. In addition, all patient-reported outcome questionnaires may be analyzed under the efficacy estimand using an ANCOVA model with missing data in efficacy analysis set (EAS - WP) be imputed using LOCF.

4.7.1.1. Short-Form-36 Health Survey Version 2, Acute Form

Per copyright owner, the QualityMetric Health Outcomes™ Scoring (PRO_CoRe V2.0) Software will be used to derive the following domain and component scores:

- Mental Component Score (MCS)
- Physical Component Score (PCS)
- Physical Functioning domain (PF)
- Role-Physical domain (RP)
- Bodily Pain domain (BP)
- General Health domain (GH)
- Vitality domain (VT)
- Social Functioning domain (SF)
- Role-Emotional domain (RE), and
- Mental Health domain (MH).

Descriptive summaries by treatment group at each scheduled visit at which the SF-36 is administered will be presented for each domain. The change from baseline to Week 52 for each domain and component scores will be analyzed using an ANCOVA specified in Section 4.1.2.1.1.

4.7.1.2. Control of Eating Questionnaire

The CoEQ (Dalton et al. 2015) is a 19-item, participant-completed questionnaire that assesses the intensity of food cravings, food types craved, appetite, and mood over the past 7 days. The 11-point numeric rating scale ranges from 0 (not at all) to 10 (extremely) for each of the 19 items.

Seventeen of the 19 items are grouped into the 4 domains of

- craving control
- positive mood
- craving for savory foods, and

- craving for sweet foods.

The remaining 2 items, which assess the degree of hunger and fullness, are scored individually.

Higher scores represent higher levels of the concept measured in each domain or individual item (for the 2 standalone items).

Descriptive summaries by treatment group at each schedule visit at which the CoEQ is administered will be presented for each item. The change from baseline to Week 52 for each domain will be analyzed using an ANCOVA specified in Section 4.1.2.1.1.

4.7.1.3. Patient Global Impression of Severity for Physical Function Weight (PGIS-Physical Function Weight)

The PGIS-Physical Function due to Weight scale is designed to assess the participants' overall perception of their condition. This is a single global item that asks participants to rate how their weight limited their ability to perform physical activities in the past 7 days on a 5-point scale ranging from "not at all limited" to "extremely limited".

A shift table from baseline to postbaseline of the response will be created at each postbaseline visit.

4.7.1.4. Patient Global Impression of Severity for Food Craving (PGIS-Food Craving)

The PGIS Food Craving item is a patient-reported, single-item measure that assesses participants' overall perception of their food craving in the past 7 days on a 5-point scale:

- much higher than usual
- somewhat higher than usual
- same as usual
- somewhat lower than usual, and
- much lower than usual.

A shift table from baseline to postbaseline of the response will be created at each postbaseline visit.

4.7.1.5. Patient Global Impression of Change for Physical Function Weight (PGIC-Physical Function Weight)

The PGIC-Physical Function due to Weight scale is designed to assess the participants' overall perception of the efficacy of treatment. This is a single global item that asks participants to rate the overall change in their ability to perform physical activities due to their weight since starting the study medication. The responses are based on a 5-point scale ranging from "much better" to "much worse".

The counts and percentages of participants for PGIC of change for physical function weight categories at each postbaseline time point will be summarized by nominal visit and by treatment.

4.7.1.6. Patient Global Impression of Change for Food Craving (PGIC-Food Craving)

PGIC Food Craving item is a patient-reported, single-item measure that assesses participants' change in their food craving since they started taking the study medication. The item is rated on a 5-point scale:

- increased a lot
- increased a little
- no change
- decreased a little, and
- decreased a lot.

The counts and percentages of participants for PGIC of change for food craving categories at each postbaseline time point will be summarized by nominal visit and by treatment.

4.7.1.7. Rapid Prime Diet Quality Score (rPDQS)

The rPDQS is a brief diet quality screener that identifies clinically relevant patterns of food intake. The rPDQS starts with the following sentence: "Thinking back over the past month, how often have you eaten each of the following foods?" This question is followed by 6 food group items with protective health associations (for example, fish, whole grains, beans, vegetables, fruits, and peanut butter/nuts) and 7 food group items with adverse health associations (for example, processed meats, beef/pork/lamb, full-fat dairy, fast food, sugary beverages, white bread, pasta, sweets, and desserts). The responses to these 13 items are included in a dietary score.

The rPDQS questions are ordered with

1. protein sources (for example, meat, fish, and dairy),
2. carbohydrate sources (for example, fast food, sugary drinks, breads, sweets, fruits, and vegetables), and
3. fat sources (for example, nuts).

For each question, respondents select from 5 possible answers ranging from "less than once per week" to "twice per day or more". The scoring approach allocates 0 to 4 points for consumption of each of the 6 food groups whose intake is encouraged (with a reverse scoring for the 7 food groups to limit) based on the original frequency of consumption (range, 0 to 52; higher score being healthier). The total rPDQS strongly correlated with the Healthy Eating Index (HEI)- 2015 diet quality metric estimated from 24-hour diet recalls, suggesting that the screener provides an informative assessment of an individual's dietary intake (Kronsteiner-Gicevic et al. 2023).

Descriptive summaries by treatment group at each scheduled visit at which the rPDQS is administered will be presented for each item. The change from baseline to Week 52 for each domain will be analyzed using an ANCOVA specified in Section 4.1.2.1.1.

4.7.2. Subgroup Analyses

Subgroup analyses of the primary endpoint, percent maintenance of BW reduction achieved during the 72 weeks of treatment with tirzepatide or semaglutide for those who have achieved a BW plateau will be made based on the modified treatment regimen using the FAS-WP to assess consistency of the intervention effect. The variables for subgroup analysis are specified in Appendix 1.

The ANCOVA model specified in Section 4.1.2.1.1 will be fitted separately within each category of subgroup. The model-based estimated mean, estimated treatment difference, SE, and 95% CI will be presented. The model-based estimated means and variance-covariance estimates from these separate models will be used to test the treatment-by-subgroup interaction at the significance level of 0.10.

If any category within the subgroup is <5% of the total population, only descriptive statistics will be provided for that category (that is, there will be no inferential testing within the subgroup category).

4.8. Interim Analysis

No interim analyses are planned for this study. If an unplanned interim analysis is deemed necessary for reasons other than a safety concern, the protocol must be amended. Final analysis will be performed when all participants complete the study.

4.9. Changes to Protocol-Planned Analyses

The ANCOVA and the MMRM models specified in Sections 4.1.2.1 and 4.1.2.2 in the SAP have been updated compared to the models specified in Section 9.3.2 in the protocol to be consistent with the most recent recommendations for ANCOVA (Ye et al. 2022; FDA 2023) and MMRM analyses (Wang and Du 2024).

5. Sample Size Determination

For each treatment group in the SURMOUNT-5 study, a sample size of 150 participants with a 3:2 randomization ratio (90/60 in orforglipron and placebo, respectively) is needed to ensure that 118 participants (70/48 in orforglipron and placebo, respectively) reach BW plateau. This sample size yields approximately 90% power to detect a 10% treatment difference for the primary endpoint of percent maintenance of BW reduction achieved during the 72 weeks of treatment in SURMOUNT-5, assuming a 20% discontinuation rate, and a common standard deviation of 14% using a 2-group t-test with a 5% 2-sided significant level.

All participants completing the SURMOUNT-5 study who meet the entry criteria for this study including completing the SURMOUNT-5 study on study treatment, completing Visits 2, 17, and 20 of SURMOUNT-5 study, and achieving at least a 5% weight reduction during the 72 weeks of treatment with tirzepatide or semaglutide, will be offered to enroll in this study. Therefore, the actual number of enrolled participants may be higher than planned.

6. Supporting Documentation

6.1. Appendix 1: Demographic and Baseline Characteristics

A listing of participant demographics for all randomized participants will be provided. All demographic and baseline clinical characteristics will be summarized by study treatment for participants in the randomized populations.

Table GZPN.6.1 describes the specific variables and how they will be summarized. The last column specifies variables used for the subgroup analysis described in Section 4.7.1.

Table GZPN.6.1. Demographics and Baseline Characteristics with Variables for Subgroup Analysis

Variable	Quantitative Summary	Categorical Summary	Subgroup Analysis[a]
Demographics			
Age[b]	Yes	<65, ≥65 years	X
		<75, ≥75 years	
		<65, ≥65 and <75, ≥75 and <85, ≥85 years	
Sex	No	Male, Female	X
Race	No	American Indian/Alaska Native, Asian, Black/African American, Native Hawaiian or other Pacific Islander, White, or Multiple	X
Height (cm)	Yes		
Baseline waist circumference (cm)	Yes		
Baseline body weight (kg)	Yes		
Baseline BMI	Yes	<30, ≥30 to <35, ≥35 to <40, ≥40 kg/m^2	X for : <30, ≥30 kg/m^2
Caffeine use	No	Never, Current, Former	
Alcohol use	No	Never, Current, Former	
Tobacco use	No	Never, Current, Former	
percent BW reduction prior to randomization	Yes	(<20%, ≥20%).	X
Baseline Disease Characteristics			
Baseline systolic blood pressure (mmHg)	Yes		
Baseline diastolic blood pressure (mmHg)	Yes		
Baseline pulse rate (beats/min)	Yes		
Baseline eGFR CKD-EPI Cystatin-C (mL/min/1.73m^2)	Yes	<60, ≥60 mL/min/1.73m^2	
Baseline eGFR CKD-EPI Creatinine (mL/min/1.73m^2)	Yes	<60, ≥60 mL/min/1.73m^2	
Baseline UACR (g/kg)	Yes	<30, ≥30 and ≤300, >300 g/kg	
Baseline triglycerides (mg/dL)	Yes		
Baseline total cholesterol (mg/dL)	Yes		
Baseline VLDL-cholesterol (mg/dL)	Yes		
Baseline non-HDL-cholesterol (mg/dL)	Yes		
Baseline LDL-cholesterol (mg/dL)	Yes		
Baseline HDL-cholesterol (mg/dL)	Yes		
Baseline fasting insulin (pmol/L)	Yes		

Variable	Quantitative Summary	Categorical Summary	Subgroup Analysis[a]
Baseline HbA1c (%)	Yes	<5.7, ≥5.7 and ≤6.4, ≥6.5	
Baseline HbA1c (mmol/mol)	Yes		
Baseline fasting glucose (mg/dL)	Yes		
Baseline fasting glucose (mmol/L)	Yes		
Duration of obesity (years)	Yes		
Prediabetes[c]	No	Yes, No	

Abbreviations: eGFR = estimated glomerular filtration rate; HDL = high-density lipoprotein; LDL = low-density lipoprotein; UACR = urine albumin-to-creatinine ratio; VLDL = very low-density lipoprotein.

a Subgroup analyses are defined in Section 4.7.1 with more details.

b Age in years will be calculated as length of the time interval from the imputed date of birth (July 1 in the year of birth collected in the eCRF) to the informed consent date.

c Prediabetes status at baseline is determined from the laboratory data in LabsConnect.

6.2. Appendix 2: Historical Illnesses and Preexisting Conditions

The count and percentages of participants with historical illnesses and preexisting conditions will be summarized by treatment group using the MedDRA PTs nested within SOC. The SOC will be in alphabetical order. Events will be ordered by decreasing frequency. Conditions (that is, PTs) will be ordered by decreasing frequency within SOC. This will be summarized for all randomized participants. Historical illnesses are illnesses that end prior to informed consent and preexisting conditions are conditions that are still ongoing at informed consent. No statistical comparisons between treatment groups will be performed.

6.3. Appendix 3: Treatment Compliance

Treatment compliance for each visit interval is defined as taking at least 75% of the required doses of study drug. Similarly, a participant will be considered significantly noncompliant if he or she is judged by the investigator to have intentionally or repeatedly taken more than the prescribed amount of medication (more than 125%). Compliance over the study period will be calculated using the number of doses administered (regardless of the actual dose in mg administered) divided by the total number of doses expected to be administered ×100 over the study period. Treatment compliance will be summarized descriptively in the study period by treatment using the safety population.

6.4. Appendix 4: Prior/Concomitant Medications

Medications that start before or at the last date of treatment period or follow-up period and are ongoing or ended during the treatment period or follow-up period will be classified as concomitant medication. Medications that start and end before the first dose date of the study intervention will be classified as prior therapy.

Baseline is defined as the corresponding medication taken on the day before the date of the first dose of study intervention.

If there are no doses of study intervention, randomization date will be used instead of the first dose date.

The planned summaries for concomitant medications are provided in Table GZPN 6.x.

Additionally, medications of interest (as defined below) will be summarized by treatment group.

Concomitant medications of interest include the following: Use of the following medications after baseline:

- antidiarrheal medication
- antiemetic medication.

The following list of standardized generic names (CMDECOD) will be used to define the intercurrent event of initiation of prohibited medication:

Drug	CMDECOD	CMDECOD code
AOMs		
Orlistat	ORLISTAT	01215601001
Sibutramine	SIBUTRAMINE	01356801001
Phenylpropanolamine	PHENYLPROPANOLAMINE	00103801001
Mazindol	MAZINDOL	00309701001
Phentermine	PHENTERMINE	00131701001
Lorcaserin	LORCASERIN	07224601001
Phentermine and Topiramate	PHENTERMINE;TOPIRAMATE	12942601001
Bupropion and Naltrexone	BUPROPION;NALTREXONE	12725401001
Plenity	CELLULOSE MICROCRYSTALLINE;CITRIC ACID	15738901002
Benzphetamine	BENZFETAMINE	00554001005
Diethylpropion	AMFEPRAMONE	00052701006
GLP-1 RAs, GIP/GLP-1 RAs:		
Liraglutide	LIRAGLUTIDE	05745801001
Dulaglutide	DULAGLUTIDE	07237701001
Exenatide	EXENATIDE	03598701001
Semaglutide	SEMAGLUTIDE	06507301001
Tirzepatide	TIRZEPATIDE	15438301001
DPP-4 Inhibitors:		
Sitagliptin	SITAGLIPTIN	05710001001
Saxagliptin	SAXAGLIPTIN	05722201001
Linagliptin	LINAGLIPTIN	06301101001
Alogliptin	ALOGLIPTIN	06373001001

The following list of standardized codes (MHDECOD) will be used to define the intercurrent event of having bariatric surgery or other weight loss procedures:

Standardized Code
Bariatric gastric balloon insertion
Endoscopic sleeve gastroplasty
Metabolic surgery
Gastric banding

6.5. Appendix 5: Important Protocol Deviations

Important protocol deviations are defined in the Trial Issues Management Plan. A listing and a summary of important protocol deviations by treatment group will be provided at the end of study.

6.6. Appendix 6: Clinical Trial Registry Analyses

Additional analyses will be performed for the purpose of fulfilling the Clinical Trial Registry (CTR) requirements.

Analyses provided for the CTR requirements include the following:

- Summary of adverse events, provided as a dataset which will be converted to an XML file. Both Serious Adverse Events and 'Other' Non-Serious Adverse Events are summarized: by treatment group, by MedDRA preferred term.
    - An adverse event is considered 'Serious' whether or not it is a treatment emergent adverse event (TEAE).
    - An adverse event is considered in the 'Other' category if it is both a TEAE and is not serious. For each Serious AE and 'Other' AE, for each term and treatment group, the following are provided:
        - the number of participants at risk of an event
        - the number of participants who experienced each event term
        - the number of events experienced.
- For each Serious AE, these additional terms are provided for EudraCT:
    - the total number of occurrences causally related to treatment
    - the total number of deaths
    - the total number of deaths causally related to treatment.
- Consistent with www.ClinicalTrials.gov requirements, 'Other' AEs that occur in fewer than 5% of patients/subjects in every treatment group may be excluded if a 5% threshold is chosen. Allowable thresholds include 0% (all events), 1%, 2%, 3%, 4% and 5%.
- AE reporting is consistent with other document disclosures for example, the CSR, manuscripts, and so forth.

Demographic table including the following age ranges required by EudraCT: adults (18-64 years), 65-85 years, and 85 years and over.

7. References

American Diabetes Association Professional Practice Committee. 6. Glycemic Goals and Hypoglycemia: Standards of Care in Diabetes-2025. *Diabetes Care*. 2025;48(Suppl 1):S128-S145. https://doi.org/10.2337/dc25-s006

Andersen SW, Millen BA. On the practical application of mixed effects models for repeated measures to clinical trial data. *Pharm Stat*. 2013;12(1):7-16. https://doi.org/10.1002/pst.1548

Alosh, M, Bretz, F, Huque M. Advanced multiplicity adjustment methods in clinical trials. *Stat Med.* 2014;33(4):693-713. https://doi.org/10.1002/sim.5974

Bretz F, Maurer W, Brannath W, Posch M. A graphical approach to sequentially rejective multiple test procedures. *Stat Med*. 2009;28(4):586-604. https://doi.org/10.1002/sim.3495

Bretz, F, Posch M, Glimm E, et al. Graphical approaches for multiple comparison procedures using weighted Bonferroni, Simes, or parametric tests. *Biom J*. 2011;53(6):894-913. https://doi.org/10.1002/bimj.201000239

Cappelleri JC, Bushmakin AG, Gerber RA, et al. Evaluating the Power of Food Scale in obese subjects and a general sample of individuals: development and measurement properties. *Int J Obes (Lond)*. 2009;33(8):913-922. https://doi.org/10.1038/ijo.2009.107

[CDER/BIRRS] Center for Drug Evaluation and Research (CDER) and Biomedical Informatics and Regulatory Review Science (BIRRS) Team. Advancing premarket safety analytics workshop meet material_FDA-DM FMQs. Published September 06, 2022a. Accessed November 20, 2023. https://www.regulations.gov/document/FDA-2022-N-1961-0001

[CDER/BIRRS] Center for Drug Evaluation and Research (CDER) and Biomedical Informatics and Regulatory Review Science (BIRRS) Team. Standard Safety Tables and Figures: Integrated Guide. Published August 2022b. Accessed November 20, 2023. https://www.regulations.gov/document/FDA-2022-N-1961-0046

Chapman R. Expected a posteriori scoring in PROMIS®. *J Patient Rep Outcomes*. 2022;6(1):59. https://doi.org/10.1186/s41687-022-00464-9

Diggle PJ, Liang KY, Zeger SL. *Analysis of Longitudinal Data*. Clarendon Press; 1994.

Du Y, Li J, Raha S, Qu Y. A unified Bayesian framework for bias adjustment in multiple comparisons from clinical trials. *Stat Med*. 2024;43(15):2928-2943. https://doi.org/10.1002/sim.10064

[EMA] European Medicines Agency. Guidance document for the content of the <Co->Rapporteur day 80 critical assessment report. EMA/269176/2014. Published 2014. Accessed May 3, 2023. https://www.ema.europa.eu/en/documents/regulatory-procedural-guideline/day-80-assessment-report-clinical-guidance_en.pdf

EuroQol Research Foundation. EQ-5D-5L User Guide, Version 3.0. Updated September 2019. Accessed July 29, 2022. https://euroqol.org/publications/user-guides

[FDA] Center for Drug Evaluation and Research (CDER) and Center for Biologics Evaluation and Research (CBER). Adjusting for covariates in randomized clinical trials for drugs and biological products: Guidance for industry. Published May 2023. Accessed July 24, 2023. https://www.fda.gov/regulatory-information/search-fda-guidance-documents/adjusting-covariates-randomized-clinical-trials-drugs-and-biological-products

Flint A, Raben A, Blundell JE, Astrup A. Reproducibility, power and validity of visual analogue scales in assessment of appetite sensations in single test meal studies. *Int J Obes Relat Metab Disord.* 2000;24(1):38-48. https://doi.org/10.1038/sj.ijo.0801083.

[ICH] International Council for Harmonisation. ICH harmonised guideline. Addendum on estimands and sensitivity analysis in clinical trials to the guideline on statistical principles for clinical trials. E9(R1). Adopted on November 20, 2019. Accessed November 07, 2022. https://database.ich.org/sites/default/files/E9-R1_Step4_Guideline_2019_1203.pdf

Inker LA, Schmid CH, Tighiouart H, et al.; CKD-EPI Investigators. Estimating glomerular filtration rate from serum creatinine and cystatin C. *N Engl J Med*. 2012;367(1):20-29. https://doi.org/10.1056/NEJMoa1114248.

[JASSO] Japan Society for the Study of Obesity. Guideline for the management of obesity disease [in Japanese]. 2022. Tokyo: Life Science Publishing. 2022.

Kolotkin RL, Ervin CM, Meincke HH, et al. Development of a clinical trials version of the Impact of Weight on Quality of Life-Lite questionnaire (IWQOL-Lite Clinical Trials Version): results from two qualitative studies. *Clin Obes*. 2017;7(5):290-299. https://doi.org/10.1111/cob.12197

Kolotkin RL, Williams VSL, Ervin CM, et al. Validation of a new measure of quality of life in obesity trials: Impact of Weight on Quality of Life-Lite Clinical Trials Version. *Clin Obes*. 2019;9(3):e12310. https://doi.org/10.1111/cob.12310

Lowe MR, Butryn ML, Didie ER, et al. The Power of Food Scale. A new measure of the psychological influence of the food environment. *Appetite.* 2009;53(1):114-118. https://doi.org/10.1016/j.appet.2009.05.016

Ma C, Shen X, Qu Y, Du Y. Analysis of an incomplete binary outcome dichotomized from an underlying continuous variable in clinical trials. *Pharm Stat*. 2022;21(5):907-918. https://doi.org/10.1002/pst.2204

Maruish ME, editor. User's Manual for the SF36v2 Health Survey. 3rd ed. Lincoln, RI: Quality Metric Incorporated; 2011

PHUSE. Analysis and displays associated with adverse events: focus on adverse events in Phase 2-4 clinical trials and integrated summary documents. Published February 03, 2017. Accessed May 30, 2023. https://phuse.s3.eu-central-1.amazonaws.com/Deliverables/Standard+Analyses+and+Code+Sharing/Analyses+and+Displays+Associated+with+Adverse+Events+Focus+on+Adverse+Events+in+Phase+2-4+Clinical+Trials+and+Integrated+Summary.pdf

PHUSE. Analysis and displays associated with demographics, disposition, and medications. Published March 02, 2018. Accessed May 30, 2023. https://phuse.s3.eu-central-1.amazonaws.com/Deliverables/Standard+Analyses+and+Code+Sharing/Analyses+%26+Displays+Associated+with+Demographics,+Disposition+and+Medication+in+Phase+2-4+Clinical+Trials+and+Integrated+Summary+Documents.pdf

PHUSE. Analyses and displays associated with laboratory analyte measurements in Phase 2-4 clinical trials and integrated submission documents – update to recommendations. Published August 19, 2022. Accessed May 30, 2023. https://phuse.s3.eu-central-1.amazonaws.com/Deliverables/Safety+Analytics/WP068.pdf

PHUSE. Analyses and displays associated with measures of central tendency – focus on vital sign, electrocardiogram, and laboratory analyte measurements in Phase 2-4 clinical trials and integrated submission documents. Published October 10, 2013. Accessed May 30, 2023. https://phuse.s3.eu-central-1.amazonaws.com/Deliverables/Standard+Analyses+and+Code+Sharing/Analyses+%26+Displays+Associated+with+Measures+of+Central+Tendency-+Focus+on+Vital+Sign,+Electrocardiogram+%26+Laboratory+Analyte+Measurements+in+Phase+2-4+Clinical+Trials+and+Integrated+Submissions.pdf

PHUSE. Analyses and displays associated with outliers or shifts from normal to abnormal: focus on vital signs, electrocardiogram, and laboratory analyte measurements in Phase 2-4 clinical trials and integrated summary documents. Published September 10, 2015. Accessed May 30, 2023. https://phuse.s3.eu-central-1.amazonaws.com/Deliverables/Standard+Analyses+and+Code+Sharing/Analyses+%26+Displays+Associated+with+Outliers+or+Shifts+from+Normal+To+Abnormal+Focus+on+Vital+Signes+%26+Electrocardiogram+%26+Laboratory+Analyte+Measurements+in+Phase+2-4+Clinical+Trials+and+Integrated+Summary.pdf

Röver C, Bender R, Dias S, et al. On weakly informative prior distributions for the heterogeneity parameter in Bayesian random-effects meta-analysis. *Res Synth Methods*. 2021;12(4):448-474. https://doi.org/10.1002/jrsm.1475.

Sadoul BC, Schuring EAH, Mela DJ, Peters HPF. The relationship between appetite scores and subsequent energy intake: an analysis based on 23 randomized controlled studies. *Appetite*. 2014;83:153-159. https://doi.org/10.1016/j.appet.2014.08.016

Ullrich J, Ernst B, Wilms B, et al. Roux-en Y gastric bypass surgery reduces hedonic hunger and improves dietary habits in severely obese subjects. *Obes Surg*. 2013:23(1):50-55. https://doi.org/10.1007/s11695-012-0754-5

van Can J, Sloth B, Jensen CB, et al. Effects of the once-daily GLP-1 analog liraglutide on gastric emptying, glycemic parameters, appetite and energy metabolism in obese, non-diabetic adults. *Int J Obes (Lond)*. 2014;38(6):784-793. https://doi.org/10.1038/ijo.2013.162

Wang B, Du Y. Improving the mixed model for repeated measures to robustly increase precision in randomized trials. *Int J Biostat*. 2023:20(2):585-598. https://doi.org/10.1515/ijb-2022-0101

Ye T, Bannick M, Yi Y, Shao J. Robust variance estimation for covariate-adjusted unconditional treatment effect in randomized clinical trials with binary outcomes. *Stat Theory Relat Fields*. 2023;7(2):159-163. https://doi.org/10.1080/24754269.2023.2205802

Ye T, Shao J, Yi Y, Zhao Q. Toward better practice of covariate adjustment in analyzing randomized clinical trials. *J Am Stat Assoc*. 2022;118(544):2370-2382. https://doi.org/10.1080/01621459.2022.2049278

Signature Page for VV-CLIN-156239 v1.0

Approval	PPD<br>Statistician<br>21-Nov-2025 17:16:46 GMT+0000

Signature Page for VV-CLIN-156239 v1.0

Approved on 21 Nov 2025 GMT
