# Supplementary material for: Orforglipron for maintenance of body weight reduction: the double-blind, randomized phase 3b ATTAIN-MAINTAIN trial
Source: Nat Med. 2026 May 13;32(7):2679–87. doi: 10.1038/s41591-026-04386-7 (PMC13375559; doi:10.1038/s41591-026-04386-7)
Supplement: Supplementary file 2 — Reporting Summary [file 41591_2026_4386_MOESM2_ESM.pdf]

nature portfolio

Corresponding author(s): Louis J. Aronne

Last updated by author(s): Mar 23, 2026

Reporting Summary

Nature Portfolio wishes to improve the reproducibility of the work that we publish. This form provides structure for consistency and transparency in reporting. For further information on Nature Portfolio policies, see our Editorial Policies and the Editorial Policy Checklist.

Statistics

For all statistical analyses, confirm that the following items are present in the figure legend, table legend, main text, or Methods section.

n/a	Confirmed	
☐	☒	The exact sample size (*n*) for each experimental group/condition, given as a discrete number and unit of measurement
☒	☐	A statement on whether measurements were taken from distinct samples or whether the same sample was measured repeatedly
☐	☒	The statistical test(s) used AND whether they are one- or two-sided<br>*Only common tests should be described solely by name; describe more complex techniques in the Methods section.*
☐	☒	A description of all covariates tested
☐	☒	A description of any assumptions or corrections, such as tests of normality and adjustment for multiple comparisons
☐	☒	A full description of the statistical parameters including central tendency (e.g. means) or other basic estimates (e.g. regression coefficient) AND variation (e.g. standard deviation) or associated estimates of uncertainty (e.g. confidence intervals)
☐	☒	For null hypothesis testing, the test statistic (e.g. *F*, *t*, *r*) with confidence intervals, effect sizes, degrees of freedom and *P* value noted<br>*Give P values as exact values whenever suitable.*
☒	☐	For Bayesian analysis, information on the choice of priors and Markov chain Monte Carlo settings
☒	☐	For hierarchical and complex designs, identification of the appropriate level for tests and full reporting of outcomes
☒	☐	Estimates of effect sizes (e.g. Cohen's *d*, Pearson's *r*), indicating how they were calculated

Our web collection on statistics for biologists contains articles on many of the points above.

Software and code

Policy information about availability of computer code

Data collection	No novel code was used for data collection
Data analysis	Statistical analyses were computed using R version 4.4.2 and SDTM datasets were produced using SAS EG 7.15/version 9.4

For manuscripts utilizing custom algorithms or software that are central to the research but not yet described in published literature, software must be made available to editors and reviewers. We strongly encourage code deposition in a community repository (e.g. GitHub). See the Nature Portfolio guidelines for submitting code & software for further information.

Data

Policy information about availability of data

All manuscripts must include a data availability statement. This statement should provide the following information, where applicable:

- Accession codes, unique identifiers, or web links for publicly available datasets
- A description of any restrictions on data availability
- For clinical datasets or third party data, please ensure that the statement adheres to our policy

Lilly provides access to all individual participant data collected during the trial, after anonymization, with the exception of pharmacokinetic or genetic data. Data are available to request 6 months after the indication studied has been approved in the US and EU and after primary publication acceptance, whichever is later. No expiration date of data requests is currently set once data are made available. Access is provided after a proposal has been approved by an independent review committee identified for this purpose and after receipt of a signed data sharing agreement. Data and documents, including the study protocol, statistical analysis

plan, clinical study report, blank or annotated case report forms, will be provided in a secure data sharing environment. For details on submitting a request, see the instructions provided at www.vivli.org.

Research involving human participants, their data, or biological material

Policy information about studies with human participants or human data. See also policy information about sex, gender (identity/presentation), and sexual orientation and race, ethnicity and racism.

Reporting on sex and gender	Only sex assigned at birth was collected for all participants. Participants were screened and recruited irrespective of sex.
Reporting on race, ethnicity, or other socially relevant groupings	Participants were screened and recruited irrespective of their race/ethnicity. Data presented in Table 1
Population characteristics	Presented in Table 1
Recruitment	Took place at 29 sites in United States
Ethics oversight	The trial was conducted in compliance with the Declaration of Helsinki and the Good Clinical Practice guidelines of the International Council for Harmonization. The trial protocol was approved at each trial site's institutional review board or independent ethics committee. A blind, independent, external committee adjudicated all deaths, pancreatic adverse events, and cardiovascular events. All participants provided written informed consent.

Note that full information on the approval of the study protocol must also be provided in the manuscript.

Field-specific reporting

Please select the one below that is the best fit for your research. If you are not sure, read the appropriate sections before making your selection.

☒ Life sciences ☐ Behavioural & social sciences ☐ Ecological, evolutionary & environmental sciences

For a reference copy of the document with all sections, see nature.com/documents/nr-reporting-summary-flat.pdf

Life sciences study design

All studies must disclose on these points even when the disclosure is negative.

Sample size	For each treatment group in the SURMOUNT-5 study, a sample size of 150 participants with a 3:2 randomization ratio (90/60 in orforglipron and placebo, respectively) was determined to ensure that 118 participants (70/48 in orforglipron and placebo, respectively) reach BW plateau. This sample size yielded approximately 90% power to detect a 10% treatment difference for the primary endpoint of percent maintenance of BW reduction achieved during the 72 weeks of treatment in SURMOUNT-5, assuming a 20% discontinuation rate, and a common standard deviation of 14% using a 2-group t-test with a 5% 2-sided significant level.
Data exclusions	In this study, all randomly assigned participants from SURMOUNT-5 study who were treated with tirzepatide or semaglutide, respectively, took at least 1 dose of study intervention in this study. Participants who were inadvertently enrolled were excluded. Two estimands, (modified treatment-regimen estimand and efficacy estimand), were used to assess treatment efficacy from different perspectives and accounted for intercurrent events. The modified treatment-regimen estimand evaluated the treatment effect regardless of treatment discontinuation or initiation of prohibited medications. This estimand also assumes that participants who had bariatric surgery or another weight loss procedure or took rescue orforglipron would not have received any additional improvement from their randomized study treatment. The efficacy estimand evaluated the treatment effect assuming that participants had stayed on treatment, had not taken prohibited medications, had not had bariatric surgery or any other weight management procedures and assuming that participants who took rescue orforglipron would not have received any additional improvement from their randomized study treatment. For the efficacy estimand, observed data after early treatment discontinuation, initiation of prohibited medications, initiation of rescue or having bariatric surgery or other weight loss procedures was not used in the analysis. For the modified treatment-regimen estimand, observed data after initiation of rescue or having bariatric surgery or other weight loss procedures was not used in the analysis.
Replication	Results were consistent between the protocol defined estimands. This study is part of a broader ATTAIN global program
Randomization	Randomization was stratified by achievement of plateau at week 72 in SURMOUNT-5, sex, and percent weight loss at week 72 of SURMOUNT-5 (<20%, ≥20%). Plateau was defined as a less than 5% change in weight during weeks 60 to 72 in the SURMOUNT-5 study.
Blinding	Study investigators, site staff, clinical monitors, and participants were blinded to the study intervention until study completion.

Reporting for specific materials, systems and methods

We require information from authors about some types of materials, experimental systems and methods used in many studies. Here, indicate whether each material, system or method listed is relevant to your study. If you are not sure if a list item applies to your research, read the appropriate section before selecting a response.

Materials & experimental systems

n/a	Involved in the study
☒	☐ Antibodies
☒	☐ Eukaryotic cell lines
☒	☐ Palaeontology and archaeology
☒	☐ Animals and other organisms
☐	☒ Clinical data
☒	☐ Dual use research of concern
☒	☐ Plants

Methods

n/a	Involved in the study
☒	☐ ChIP-seq
☒	☐ Flow cytometry
☒	☐ MRI-based neuroimaging

Clinical data

Policy information about clinical studies

All manuscripts should comply with the ICMJE guidelines for publication of clinical research and a completed CONSORT checklist must be included with all submissions.

Clinical trial registration: ClinicalTrials.gov registration : NCT06584916

Study protocol: Presented in Supplementary appendix

Data collection: Data was collected between 13 September 2024 (first participant visit) to 21 November 2025 (last participant visit), in 29 sites in the US

Outcomes: Primary and secondary objectives and endpoints were pre-specified in the protocol and statistical analysis plan. The primary endpoint was the percent maintenance of body weight reduction in SURMOUNT-5 for participants who achieved a body weight plateau. The key secondary endpoint in plateau participants included the assessment (yes/no) of maintaining at least 80% of body weight reduction. For all participants, the key secondary endpoints included the percent change in body weight from SURMOUNT-5 baseline and percent maintenance of body weight reduction. Additional secondary endpoints include evaluation of maintenance of at least 15% body weight reduction from SURMOUNT-5 baseline for participants who had already lost at least 15% body weight reduction after 72 weeks of tirzepatide or semaglutide treatment, change and percent change in body weight (in kg) from baseline to week 52, and change in waist circumference (in cm) from baseline to week 52.

Plants

Seed stocks: *Report on the source of all seed stocks or other plant material used. If applicable, state the seed stock centre and catalogue number. If plant specimens were collected from the field, describe the collection location, date and sampling procedures.*

Novel plant genotypes: *Describe the methods by which all novel plant genotypes were produced. This includes those generated by transgenic approaches, gene editing, chemical/radiation-based mutagenesis and hybridization. For transgenic lines, describe the transformation method, the number of independent lines analyzed and the generation upon which experiments were performed. For gene-edited lines, describe the editor used, the endogenous sequence targeted for editing, the targeting guide RNA sequence (if applicable) and how the editor was applied.*

Authentication: *Describe any authentication procedures for each seed stock used or novel genotype generated. Describe any experiments used to assess the effect of a mutation and, where applicable, how potential secondary effects (e.g. second site T-DNA insertions, mosiacism, off-target gene editing) were examined.*
