# Supplementary figures and images for: Orforglipron for maintenance of body weight reduction: the double-blind, randomized phase 3b ATTAIN-MAINTAIN trial
Source: Nat Med. 2026 May 13;32(7):2679–87. doi: 10.1038/s41591-026-04386-7 (PMC13375559; doi:10.1038/s41591-026-04386-7)

Cohort 1

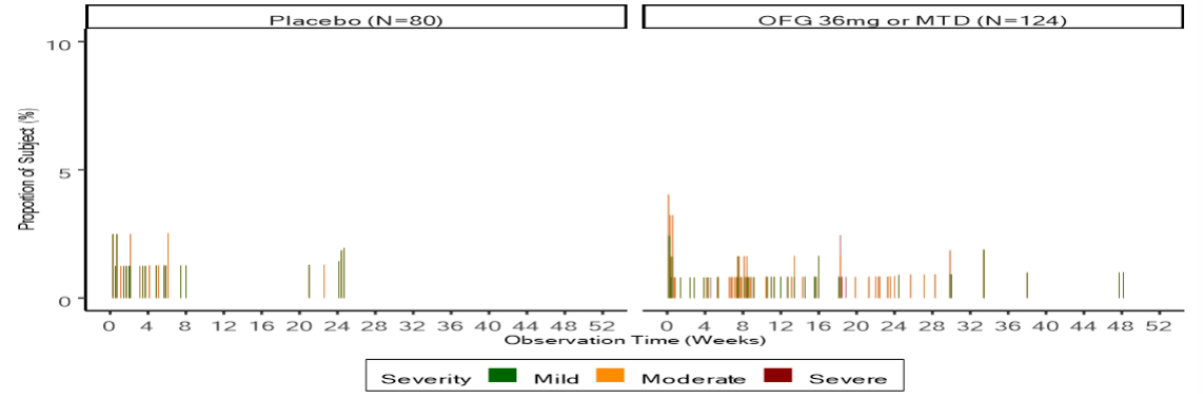

Cohort 2

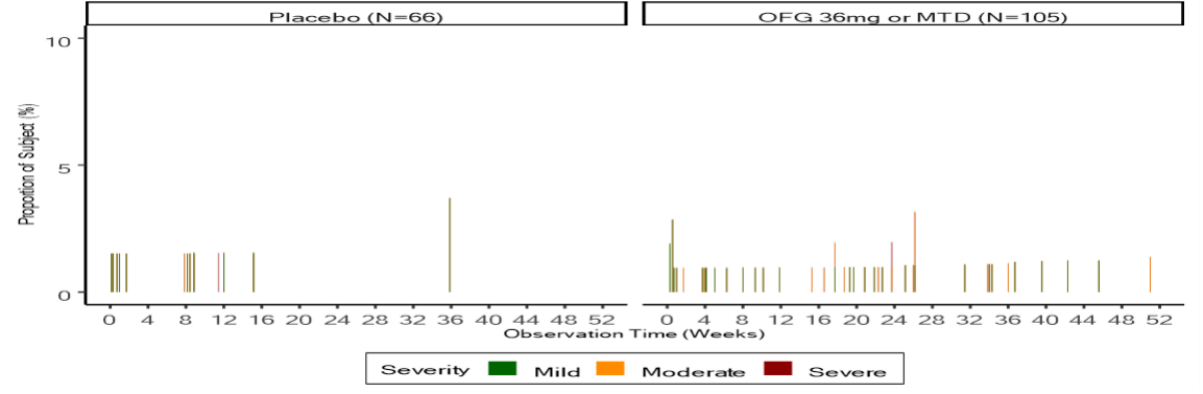

Supplement: Supplementary file 9 — Sensitivity analysis. [file 41591_2026_4386_MOESM9_ESM.pdf]
